# Supplementary figures and images for: Diverse ATPase Proteins in Mobilomes Constitute a Large Potential Sink for Prokaryotic Host ATP
Source: Front Microbiol. 2021 Jul 8;12:691847. doi: 10.3389/fmicb.2021.691847 (PMC8297831; doi:10.3389/fmicb.2021.691847)

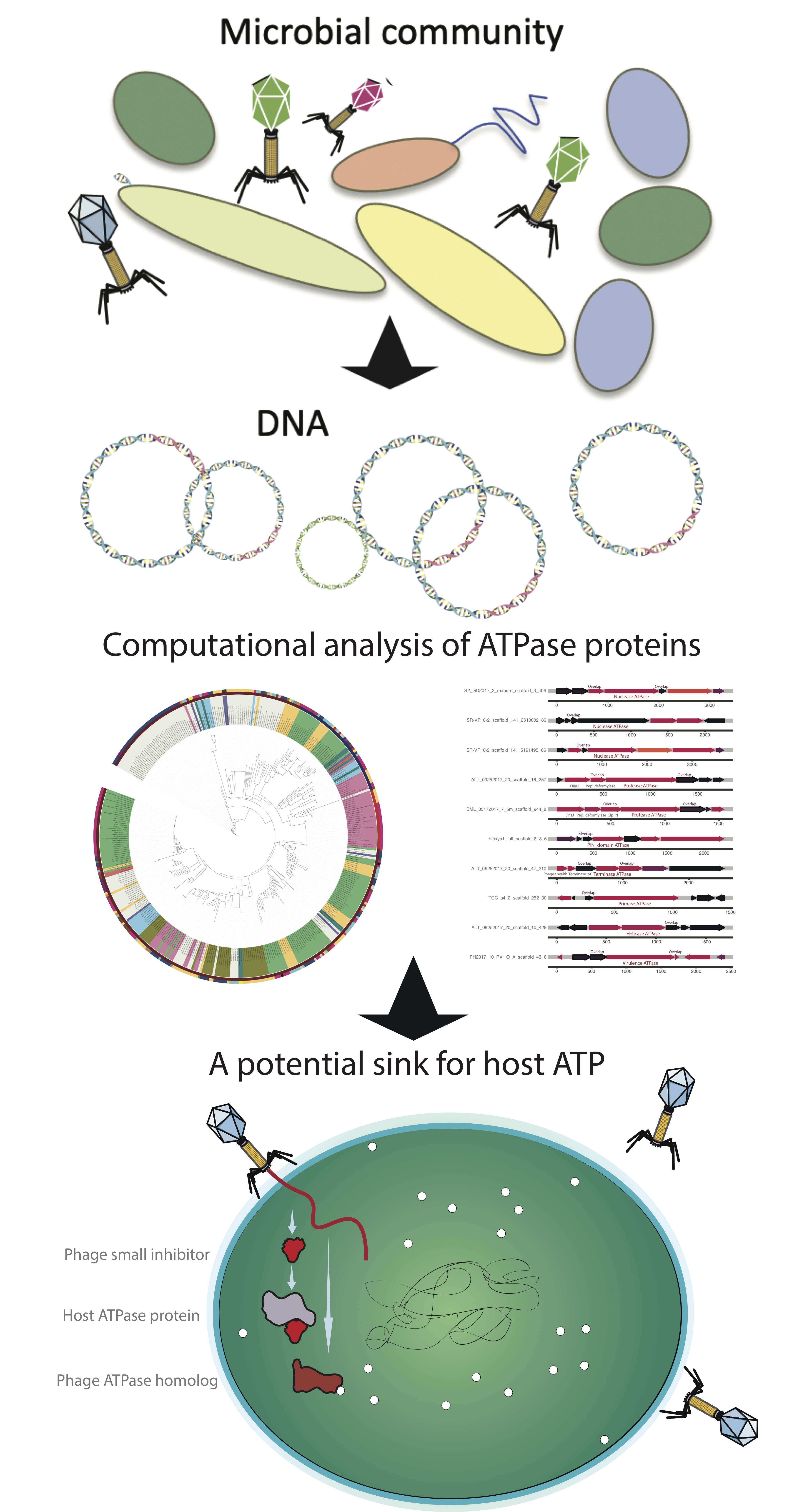

Supplement: Supplementary file 1 [file Image_1.JPEG]
