# Supplementary material for: Diverse ATPase Proteins in Mobilomes Constitute a Large Potential Sink for Prokaryotic Host ATP
Source: Front Microbiol. 2021 Jul 8;12:691847. doi: 10.3389/fmicb.2021.691847 (PMC8297831; doi:10.3389/fmicb.2021.691847)
Supplement: Supplementary file 2 [file Data_Sheet_1.docx]

**Figure S1.** ATPase proteins predicted from the huge phage dataset (A) Number of ATPase proteins against genome length fits a linear regression with significant p-value (2e-16). (B) Normal Q-Q plot shows the two distributions are linearly related and normally distributed as the points lie on the line y=x.

**A**

**B**

**Figure S2.** Tree-based exploration of neighborhoods and domains of ATP proteins with nuclease domains in the mobilome metagenome.

**
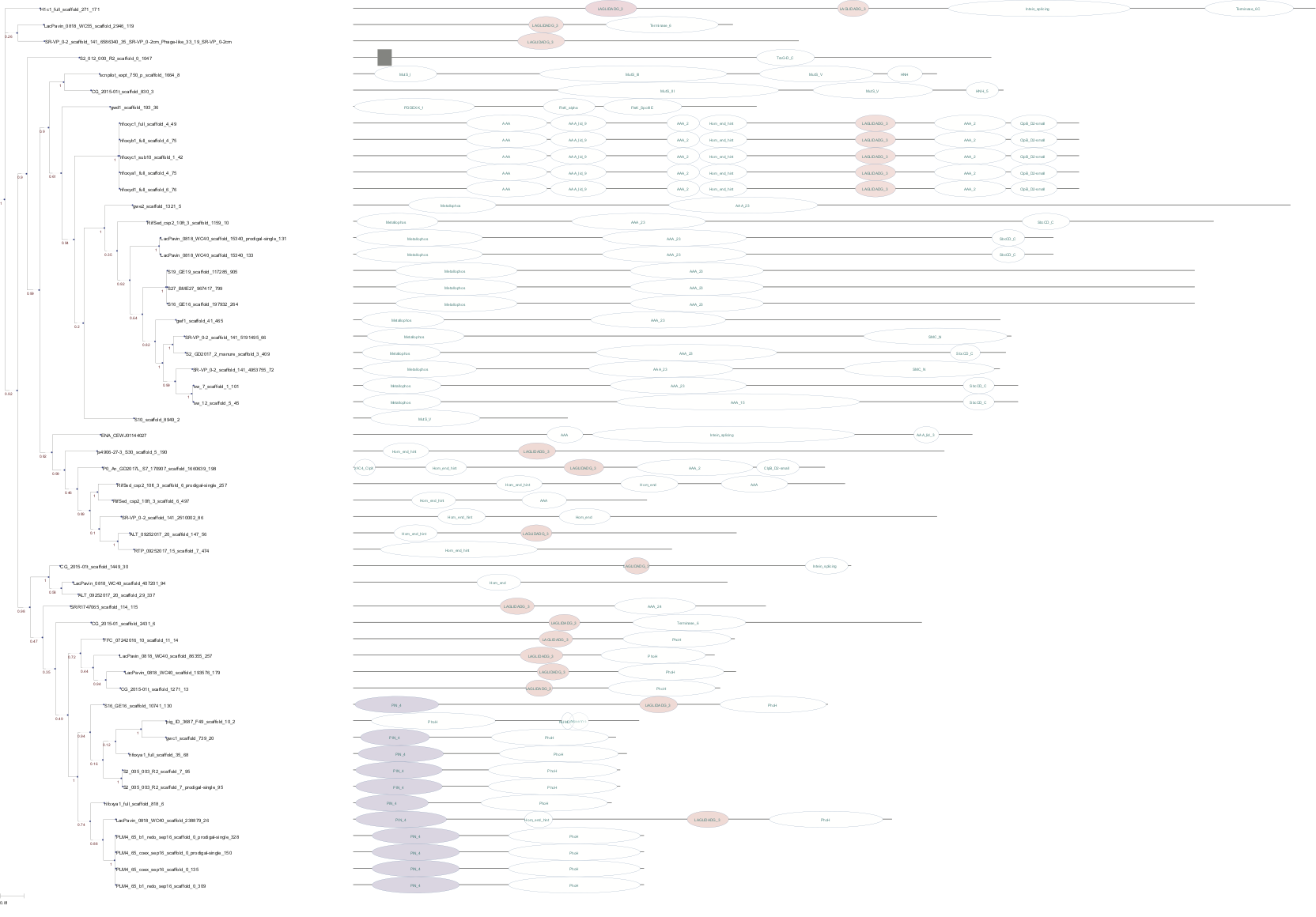
**

**Figure S3.** Multiple sequence alignment of the ATPase domains of all the composite ATPase proteins reveals the conserved regions of the ATPase family (Walker A motif and Walker B motif), as well as other regions (Sensor-1 and Sensor-2). Each ATPase domain is labelled with the biological or enzymatic activity of the functional domain that is fused together as a composite protein.

**
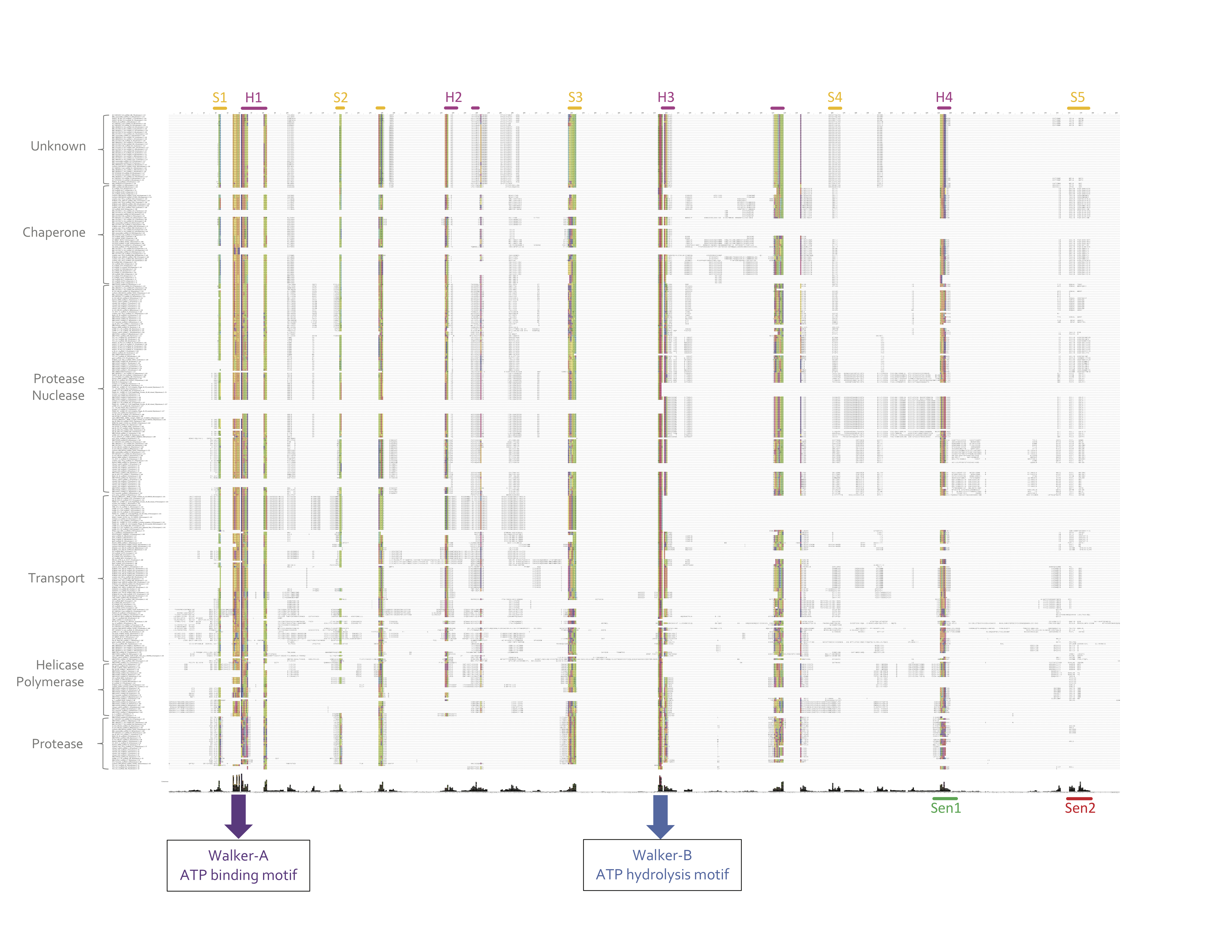
**

**Figure S4.** Lengths of co-localized proteins to composite ATPase proteins in the mobilome metagenome.

**
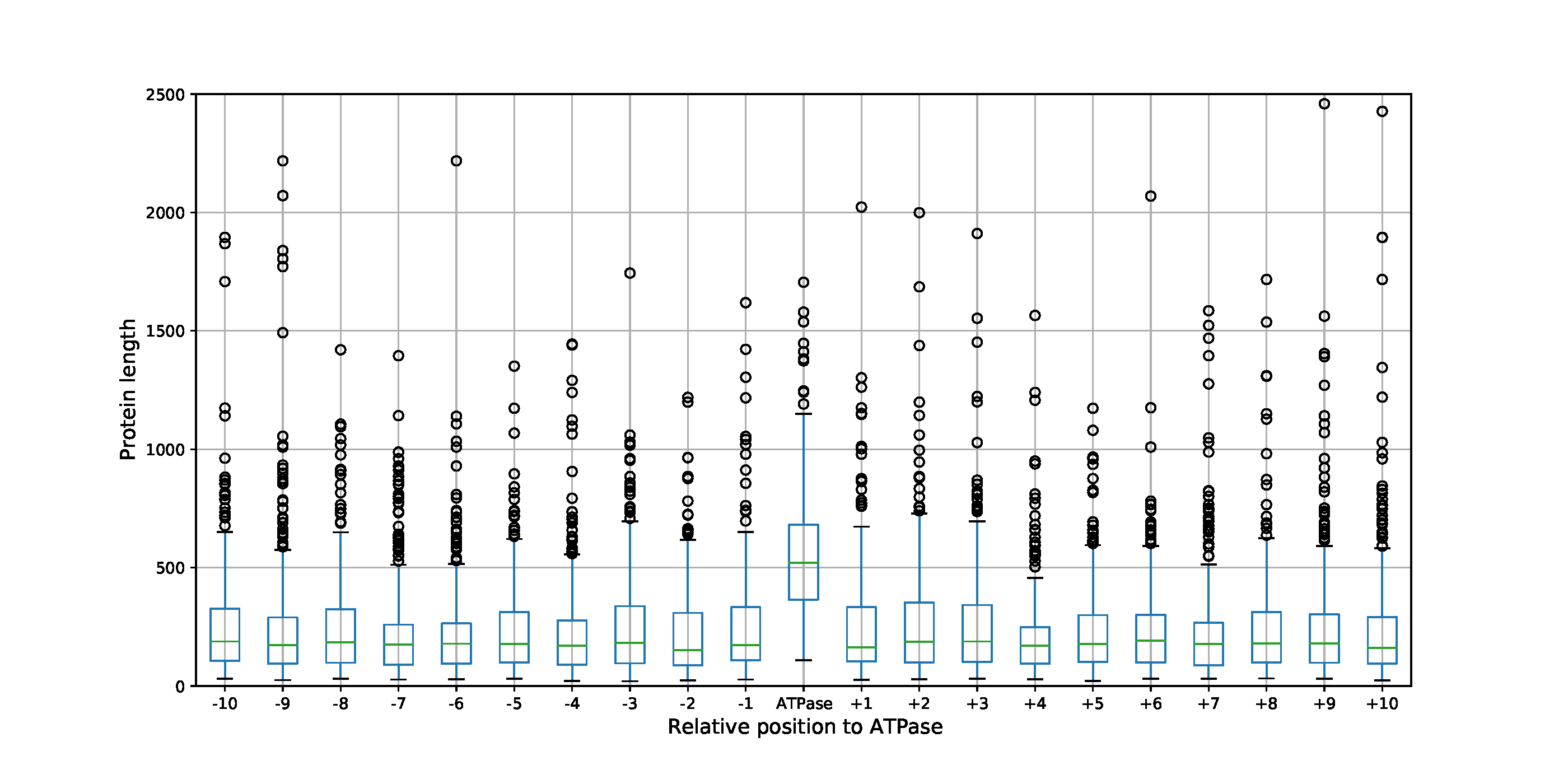
**

**Figure S5.** (A) Heatmap of genomic neighborhood of composite ATPase proteins, with the x-axis showing the proteins localized ±3 of each composite ATPase protein in the middle. The color bar represents the number of blast hits in the ggKbase database (with the threshold e-value of 1e-30) arranged by protein function (B) Heatmap of genomic neighborhood of composite ATPase proteins, with the x-axis showing the proteins localized ±3 of each composite ATPase protein in the middle. The color bar represents the number of blast hits in the ggKbase database (with the threshold e-value of 1e-30) clustered by similarity.

**A** **B**

**
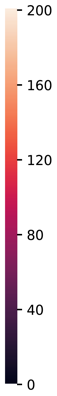
**

**Figure S6.** Phage-encoded small protein inhibiting the ATPase domain of the host protein, with a coupled protein homolog (adapted from *Hood et al. 2017*).

**Figure S7.** Boxplot of protein size by mobilome category, with the x-axis showing the mobilome category and the number of proteins in each category, and the y-axis showing the size of proteins.

**A**

**
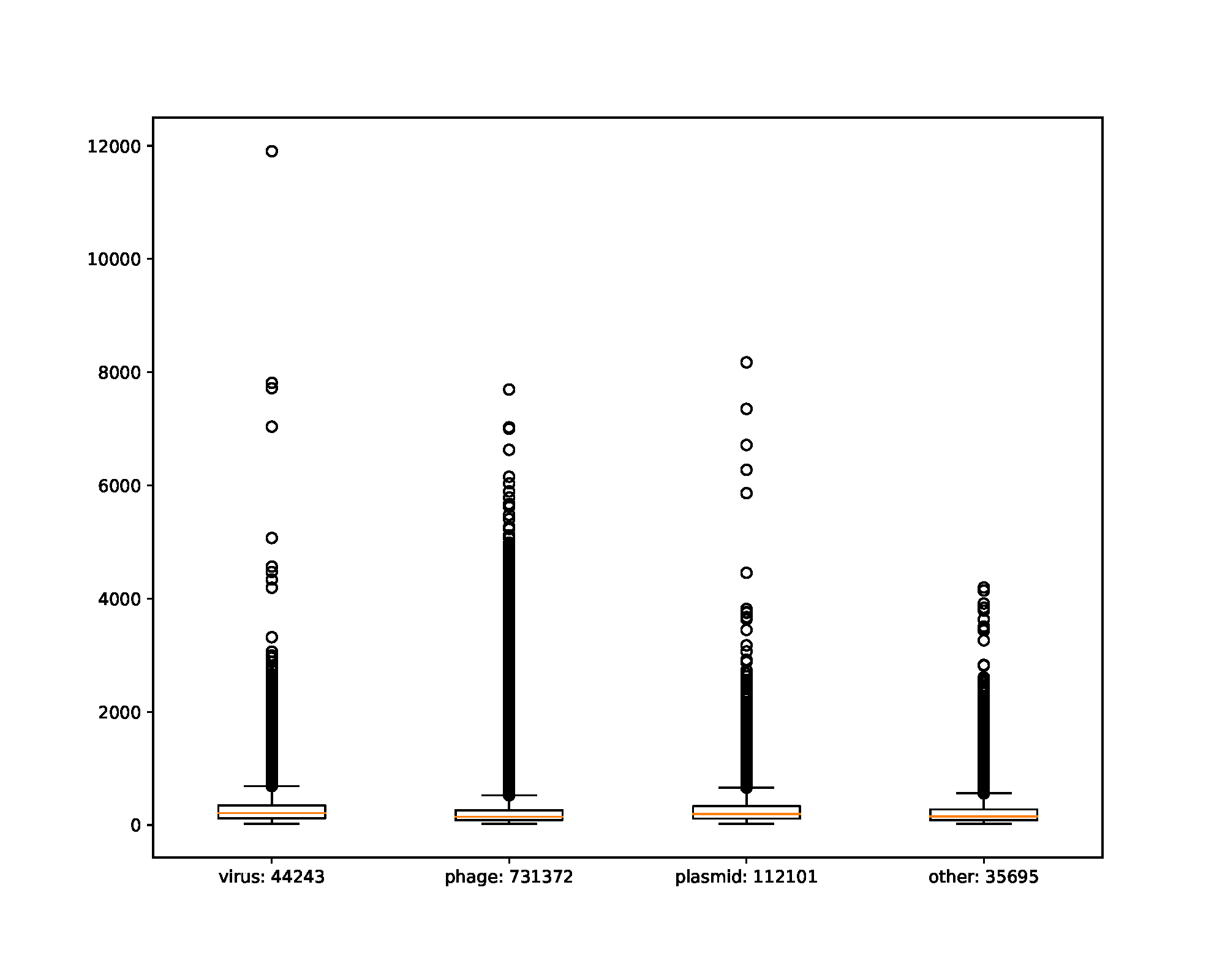
**

**Figure S8.** Boxplot of ATPase lengths by category in the mobilome metagenome.

**
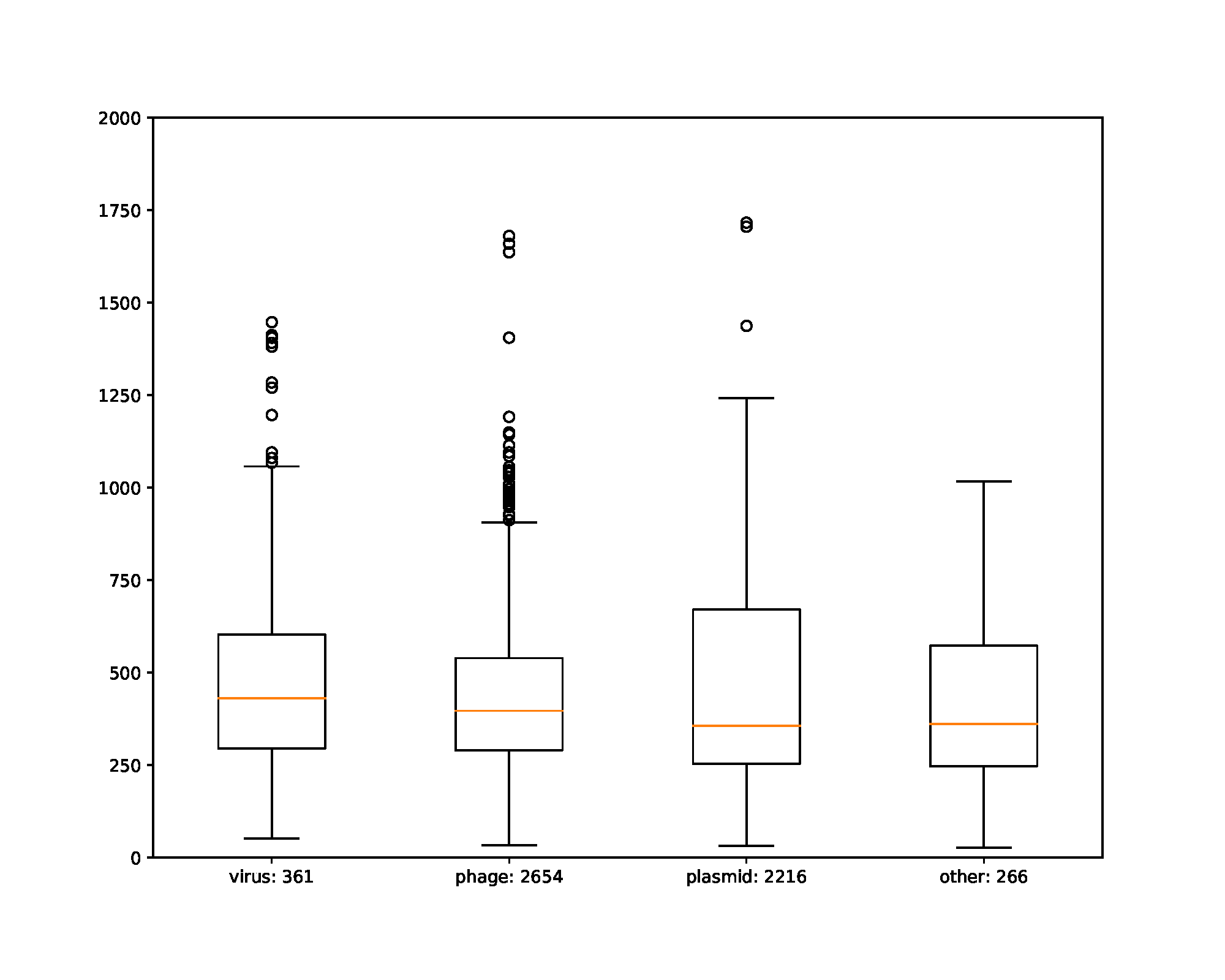
**

**Table S1.** Conservation of genomic context in composite ATPase proteins co-localized with small proteins

| **query sequence id** | subject sequence id | percentage of identical matches | alignment length | number of mismatches | number of gap openings | start of alignment in query | end of alignment in query | start of alignment in subject | end of alignment in subject | e value | bit score |
| --- | --- | --- | --- | --- | --- | --- | --- | --- | --- | --- | --- |

| **S2_GD2017_2_manure_scaffold_3_409|GD2017-2_manure_QB3_180125_Potentially_complete_Phage_48_40|GD2017-2_manure_QB3_180125** | S30_BME30_294872_48|BM_2017_Coates_4_Potentially_Complete_Phage_48-32|BM_2017_Coates_4 | 100.000 | 1071 | 0 | 0 | 1 | 1071 | 1 | 1071 | 0.0 | 2209 |
| --- | --- | --- | --- | --- | --- | --- | --- | --- | --- | --- | --- |
| **S2_GD2017_2_manure_scaffold_3_409|GD2017-2_manure_QB3_180125_Potentially_complete_Phage_48_40|GD2017-2_manure_QB3_180125** | S28_BME28_138683_397|BM_2017_Strous_8_UNK|BM_2017_Strous_8 | 100.000 | 1071 | 0 | 0 | 1 | 1071 | 1 | 1071 | 0.0 | 2209 |
| **S2_GD2017_2_manure_scaffold_3_409|GD2017-2_manure_QB3_180125_Potentially_complete_Phage_48_40|GD2017-2_manure_QB3_180125** | S27_BME27_629333_prodigal-single_185|BM_PHAGE_48_13|BM_2017_Strous_6 | 100.000 | 1071 | 0 | 0 | 1 | 1071 | 1 | 1071 | 0.0 | 2209 |
| **S2_GD2017_2_manure_scaffold_3_409|GD2017-2_manure_QB3_180125_Potentially_complete_Phage_48_40|GD2017-2_manure_QB3_180125** | S27_BME27_629333_175|BM_2017_Strous_6_Phage_48_13|BM_2017_Strous_6 | 100.000 | 1071 | 0 | 0 | 1 | 1071 | 1 | 1071 | 0.0 | 2209 |
| **S2_GD2017_2_manure_scaffold_3_409|GD2017-2_manure_QB3_180125_Potentially_complete_Phage_48_40|GD2017-2_manure_QB3_180125** | S22_GE22_scaffold_285059_prodigal-single_514|GD_PHAGE_COMPLETE_48_49|E_GD2017-2_anammox-7_S22_GE22_Biohub_180515 | 100.000 | 1071 | 0 | 0 | 1 | 1071 | 1 | 1071 | 0.0 | 2209 |
| **S2_GD2017_2_manure_scaffold_3_409|GD2017-2_manure_QB3_180125_Potentially_complete_Phage_48_40|GD2017-2_manure_QB3_180125** | S22_GE22_scaffold_285059_437|E_GD2017-2_anammox-7_S22_GE22_Biohub_170515_Potentially_Complete_48_49|E_GD2017-2_anammox-7_S22_GE22_Biohub_180515 | 100.000 | 1071 | 0 | 0 | 1 | 1071 | 1 | 1071 | 0.0 | 2209 |
| **S2_GD2017_2_manure_scaffold_3_409|GD2017-2_manure_QB3_180125_Potentially_complete_Phage_48_40|GD2017-2_manure_QB3_180125** | S20_GE20_scaffold_396645_13|E_GD2017-2_strous-5_S20_GE20_Biohub_170515_UNK|E_GD2017-2_strous-5_S20_GE20_Biohub_180515 | 100.000 | 1071 | 0 | 0 | 1 | 1071 | 1 | 1071 | 0.0 | 2209 |
| **S2_GD2017_2_manure_scaffold_3_409|GD2017-2_manure_QB3_180125_Potentially_complete_Phage_48_40|GD2017-2_manure_QB3_180125** | S15_GE15_scaffold_320047_243|E_GD2017-2_urea-2_S15_GE15_Biohub_170515_UNK|E_GD2017-2_urea-2_S15_GE15_Biohub_180515 | 100.000 | 1071 | 0 | 0 | 1 | 1071 | 1 | 1071 | 0.0 | 2209 |
| **S2_GD2017_2_manure_scaffold_3_409|GD2017-2_manure_QB3_180125_Potentially_complete_Phage_48_40|GD2017-2_manure_QB3_180125** | S14_GE14_scaffold_311130_13|E_GD2017-2_urea-2_S14_GE14_Biohub_170515_UNK|E_GD2017-2_urea-2_S14_GE14_Biohub_180515 | 100.000 | 1071 | 0 | 0 | 1 | 1071 | 1 | 1071 | 0.0 | 2209 |
| **S2_GD2017_2_manure_scaffold_3_409|GD2017-2_manure_QB3_180125_Potentially_complete_Phage_48_40|GD2017-2_manure_QB3_180125** | P0_An_pond3_S8_coassembly_k141_2723617_434|P0_An_pond3_S8_coassembly_UNK|E_GD2017-1_P0_An_pond3_S8_Biohub_coassembly | 100.000 | 1071 | 0 | 0 | 1 | 1071 | 1 | 1071 | 0.0 | 2209 |
| **S2_GD2017_2_manure_scaffold_3_409|GD2017-2_manure_QB3_180125_Potentially_complete_Phage_48_40|GD2017-2_manure_QB3_180125** | P0_An_GD2017L_S7_coassembly_k141_1013362_prodigal-single_80|GD_PHAGE_48_49|E_P0_An_GD2017L_S7_coassembly | 100.000 | 1071 | 0 | 0 | 1 | 1071 | 1 | 1071 | 0.0 | 2209 |
| **S2_GD2017_2_manure_scaffold_3_409|GD2017-2_manure_QB3_180125_Potentially_complete_Phage_48_40|GD2017-2_manure_QB3_180125** | P0_An_GD2017L_S7_coassembly_k141_1013362_74|P0_An_GD2017L_S7_coassembly_Phage_48_49|E_P0_An_GD2017L_S7_coassembly | 100.000 | 1071 | 0 | 0 | 1 | 1071 | 1 | 1071 | 0.0 | 2209 |
| **S2_GD2017_2_manure_scaffold_3_409|GD2017-2_manure_QB3_180125_Potentially_complete_Phage_48_40|GD2017-2_manure_QB3_180125** | S19_GE19_scaffold_2259_prodigal-single_281|GD_PHAGE_48_10|E_GD2017-2_strous-5-pellet_S19_GE19_Biohub_180515 | 100.000 | 1071 | 0 | 0 | 1 | 1071 | 1 | 1071 | 0.0 | 2209 |
| **S2_GD2017_2_manure_scaffold_3_409|GD2017-2_manure_QB3_180125_Potentially_complete_Phage_48_40|GD2017-2_manure_QB3_180125** | S19_GE19_scaffold_2259_257|E_GD2017-2_strous-5-pellet_S19_GE19_Biohub_170515_Phage_48_10|E_GD2017-2_strous-5-pellet_S19_GE19_Biohub_180515 | 100.000 | 1071 | 0 | 0 | 1 | 1071 | 1 | 1071 | 0.0 | 2209 |
| **S2_GD2017_2_manure_scaffold_3_409|GD2017-2_manure_QB3_180125_Potentially_complete_Phage_48_40|GD2017-2_manure_QB3_180125** | S18_GE18_scaffold_616449_288|E_GD2017-2_strous-5-prefilter_S18_GE18_Biohub_170515_UNK|E_GD2017-2_strous-5-prefilter_S18_GE18_Biohub_180515 | 100.000 | 1071 | 0 | 0 | 1 | 1071 | 1 | 1071 | 0.0 | 2209 |
| **S2_GD2017_2_manure_scaffold_3_409|GD2017-2_manure_QB3_180125_Potentially_complete_Phage_48_40|GD2017-2_manure_QB3_180125** | S16_GE16_scaffold_390213_390|E_GD2017-2_urea-3_S16_GE16_Biohub_170515_UNK|E_GD2017-2_urea-3_S16_GE16_Biohub_180515 | 100.000 | 1071 | 0 | 0 | 1 | 1071 | 1 | 1071 | 0.0 | 2209 |
| **S2_GD2017_2_manure_scaffold_3_409|GD2017-2_manure_QB3_180125_Potentially_complete_Phage_48_40|GD2017-2_manure_QB3_180125** | P0_An_pond3_S8_170907_scaffold_1005865_41|E_GD2017-1_P0_AN_POND3_S8_BIOHUB_170907_UNK|E_GD2017-1_P0_An_pond3_S8_Biohub_170907 | 100.000 | 1071 | 0 | 0 | 1 | 1071 | 1 | 1071 | 0.0 | 2209 |
| **S2_GD2017_2_manure_scaffold_3_409|GD2017-2_manure_QB3_180125_Potentially_complete_Phage_48_40|GD2017-2_manure_QB3_180125** | P0_An_GD2017L_S7_170907_scaffold_359857_24|E_GD2017-1_P0_An_GD2017L_S7_Biohub_170907_UNK|E_GD2017-1_P0_An_GD2017L_S7_Biohub_170907 | 100.000 | 1071 | 0 | 0 | 1 | 1071 | 1 | 1071 | 0.0 | 2209 |
| **S2_GD2017_2_manure_scaffold_3_409|GD2017-2_manure_QB3_180125_Potentially_complete_Phage_48_40|GD2017-2_manure_QB3_180125** | S2_GD2017_2_manure_scaffold_3_prodigal-single_482|GD_PHAGE_COMPLETE_48_40|GD2017-2_manure_QB3_180125 | 100.000 | 1071 | 0 | 0 | 1 | 1071 | 1 | 1071 | 0.0 | 2209 |
| **S2_GD2017_2_manure_scaffold_3_409|GD2017-2_manure_QB3_180125_Potentially_complete_Phage_48_40|GD2017-2_manure_QB3_180125** | S2_GD2017_2_manure_scaffold_3_409|GD2017-2_manure_QB3_180125_Potentially_complete_Phage_48_40|GD2017-2_manure_QB3_180125 | 100.000 | 1071 | 0 | 0 | 1 | 1071 | 1 | 1071 | 0.0 | 2209 |
| **S2_GD2017_2_manure_scaffold_3_409|GD2017-2_manure_QB3_180125_Potentially_complete_Phage_48_40|GD2017-2_manure_QB3_180125** | GD18-4_manure_scaffold_876276_8|GD2018-4_manure_QB3_180703_UNK|GD2018-4_manure_QB3_180703 | 100.000 | 1048 | 0 | 0 | 1 | 1048 | 1 | 1048 | 0.0 | 2164 |
| **S2_GD2017_2_manure_scaffold_3_409|GD2017-2_manure_QB3_180125_Potentially_complete_Phage_48_40|GD2017-2_manure_QB3_180125** | SR-VP_0-2_scaffold_141_2432070_17|SR-VP_0-2cm_UNK|SR-VP_0-2cm | 78.151 | 1071 | 232 | 1 | 1 | 1071 | 1 | 1069 | 0.0 | 1761 |
| **S2_GD2017_2_manure_scaffold_3_409|GD2017-2_manure_QB3_180125_Potentially_complete_Phage_48_40|GD2017-2_manure_QB3_180125** | SR-VP_0-2_scaffold_141_7948322_1|SR-VP_0-2cm_UNK|SR-VP_0-2cm | 77.328 | 988 | 223 | 1 | 85 | 1071 | 2 | 989 | 0.0 | 1617 |
| **S2_GD2017_2_manure_scaffold_3_409|GD2017-2_manure_QB3_180125_Potentially_complete_Phage_48_40|GD2017-2_manure_QB3_180125** | L3a1_full_idba_ud_scaffold_6_227|L3a1_UNK|L3a1 | 71.989 | 1071 | 298 | 1 | 1 | 1071 | 1 | 1069 | 0.0 | 1614 |
| **S2_GD2017_2_manure_scaffold_3_409|GD2017-2_manure_QB3_180125_Potentially_complete_Phage_48_40|GD2017-2_manure_QB3_180125** | S33_3P_scaffold_263290_4|Genasci_Feb2018_S33_3P_UNK|Genasci_Feb2018_S33_3P | 100.000 | 764 | 0 | 0 | 308 | 1071 | 1 | 764 | 0.0 | 1559 |
| **S2_GD2017_2_manure_scaffold_3_409|GD2017-2_manure_QB3_180125_Potentially_complete_Phage_48_40|GD2017-2_manure_QB3_180125** | SR-VP_4-6_scaffold_141_847411_1|SR-VP_4-6cm_Biohub_180515_UNK|SR-VP_4-6cm_Biohub_180515 | 75.742 | 944 | 227 | 1 | 1 | 944 | 1 | 942 | 0.0 | 1512 |
| **S2_GD2017_2_manure_scaffold_3_409|GD2017-2_manure_QB3_180125_Potentially_complete_Phage_48_40|GD2017-2_manure_QB3_180125** | L3a2_full_idba_ud_scaffold_8334_1|L3a2_MaxBin2_EukRep_ggKbase_unknown_002|L3a2 | 72.038 | 844 | 236 | 0 | 192 | 1035 | 1 | 844 | 0.0 | 1286 |
| **S2_GD2017_2_manure_scaffold_3_409|GD2017-2_manure_QB3_180125_Potentially_complete_Phage_48_40|GD2017-2_manure_QB3_180125** | PAFVLPS_2018_scaffold_33_42|circular_33|viral-cat | 53.759 | 1064 | 489 | 3 | 11 | 1071 | 10 | 1073 | 0.0 | 1210 |
| **S2_GD2017_2_manure_scaffold_3_409|GD2017-2_manure_QB3_180125_Potentially_complete_Phage_48_40|GD2017-2_manure_QB3_180125** | PAFVLPS_2018_scaffold_18_356|viral-cat_UNK|viral-cat | 53.731 | 1072 | 490 | 5 | 4 | 1071 | 5 | 1074 | 0.0 | 1200 |
| **S2_GD2017_2_manure_scaffold_3_409|GD2017-2_manure_QB3_180125_Potentially_complete_Phage_48_40|GD2017-2_manure_QB3_180125** | PAFVLPS_2018_J30_2_scaffold_1899_3|soil-virus-P14-J30-2018_UNK|soil-virus-P14-J30-2018 | 53.731 | 1072 | 490 | 5 | 4 | 1071 | 5 | 1074 | 0.0 | 1200 |
| **S2_GD2017_2_manure_scaffold_3_409|GD2017-2_manure_QB3_180125_Potentially_complete_Phage_48_40|GD2017-2_manure_QB3_180125** | MISA_07112018_0_1um_scaffold_3654_1|MISA_07112018_0_1um_UNK|MISA_07112018_0_1um | 55.741 | 1019 | 448 | 3 | 34 | 1049 | 1 | 1019 | 0.0 | 1191 |
| **S2_GD2017_2_manure_scaffold_3_409|GD2017-2_manure_QB3_180125_Potentially_complete_Phage_48_40|GD2017-2_manure_QB3_180125** | PAFVLPS_2018_scaffold_128948_1|viral-cat_UNK|viral-cat | 53.046 | 1067 | 495 | 5 | 11 | 1071 | 10 | 1076 | 0.0 | 1187 |
| **S2_GD2017_2_manure_scaffold_3_409|GD2017-2_manure_QB3_180125_Potentially_complete_Phage_48_40|GD2017-2_manure_QB3_180125** | S15_GE15_scaffold_39268_58|E_GD2017-2_urea-2_S15_GE15_Biohub_170515_UNK|E_GD2017-2_urea-2_S15_GE15_Biohub_180515 | 52.627 | 1066 | 499 | 5 | 12 | 1071 | 11 | 1076 | 0.0 | 1179 |
| **S2_GD2017_2_manure_scaffold_3_409|GD2017-2_manure_QB3_180125_Potentially_complete_Phage_48_40|GD2017-2_manure_QB3_180125** | S16_GE16_scaffold_5545_prodigal-single_28|GD_PHAGE_46_9|E_GD2017-2_urea-3_S16_GE16_Biohub_180515 | 52.627 | 1066 | 499 | 5 | 12 | 1071 | 11 | 1076 | 0.0 | 1179 |
| **S2_GD2017_2_manure_scaffold_3_409|GD2017-2_manure_QB3_180125_Potentially_complete_Phage_48_40|GD2017-2_manure_QB3_180125** | S16_GE16_scaffold_5545_27|E_GD2017-2_urea-3_S16_GE16_Biohub_170515_Phage-like_46_9|E_GD2017-2_urea-3_S16_GE16_Biohub_180515 | 52.627 | 1066 | 499 | 5 | 12 | 1071 | 11 | 1076 | 0.0 | 1179 |
| **S2_GD2017_2_manure_scaffold_3_409|GD2017-2_manure_QB3_180125_Potentially_complete_Phage_48_40|GD2017-2_manure_QB3_180125** | SR-VP_0-2_scaffold_141_5191495_prodigal-single_73|SR-VP_PHAGE_38_25|SR-VP_0-2cm | 49.200 | 1063 | 537 | 2 | 11 | 1071 | 19 | 1080 | 0.0 | 1083 |
| **S2_GD2017_2_manure_scaffold_3_409|GD2017-2_manure_QB3_180125_Potentially_complete_Phage_48_40|GD2017-2_manure_QB3_180125** | SR-VP_0-2_scaffold_141_5191495_66|SR-VP_0-2cm_Phage_38_25|SR-VP_0-2cm | 49.200 | 1063 | 537 | 2 | 11 | 1071 | 19 | 1080 | 0.0 | 1083 |
| **S2_GD2017_2_manure_scaffold_3_409|GD2017-2_manure_QB3_180125_Potentially_complete_Phage_48_40|GD2017-2_manure_QB3_180125** | LacPavin_0419_WC70S_scaffold_945204_4|LacPavin_0419_WC70S_UNK|LacPavin_0419_WC70S | 46.654 | 1061 | 561 | 4 | 11 | 1069 | 9 | 1066 | 0.0 | 1001 |
| **S2_GD2017_2_manure_scaffold_3_409|GD2017-2_manure_QB3_180125_Potentially_complete_Phage_48_40|GD2017-2_manure_QB3_180125** | LacPavin_0818_WC55_scaffold_113784_prodigal-single_368|LP_PHAGE_COMPLETE_34_34|LacPavin_0818_WC55 | 46.654 | 1061 | 561 | 4 | 11 | 1069 | 9 | 1066 | 0.0 | 1001 |
| **S2_GD2017_2_manure_scaffold_3_409|GD2017-2_manure_QB3_180125_Potentially_complete_Phage_48_40|GD2017-2_manure_QB3_180125** | LacPavin_0818_WC55_scaffold_113784_357|LacPavin_0818_WC55_Potentially_Complete_Phage_34_34|LacPavin_0818_WC55 | 46.654 | 1061 | 561 | 4 | 11 | 1069 | 9 | 1066 | 0.0 | 1001 |
| **S2_GD2017_2_manure_scaffold_3_409|GD2017-2_manure_QB3_180125_Potentially_complete_Phage_48_40|GD2017-2_manure_QB3_180125** | LacPavin_0818_WC45_scaffold_80267_117|LacPavin_0818_WC45_UNK|LacPavin_0818_WC45 | 46.654 | 1061 | 561 | 4 | 11 | 1069 | 9 | 1066 | 0.0 | 1001 |
| **S2_GD2017_2_manure_scaffold_3_409|GD2017-2_manure_QB3_180125_Potentially_complete_Phage_48_40|GD2017-2_manure_QB3_180125** | LacPavin_0718_WC55_scaffold_0_33|LacPavin_0718_WC55_Phage_34_15|LacPavin_0718_WC55 | 46.654 | 1061 | 561 | 4 | 11 | 1069 | 9 | 1066 | 0.0 | 1001 |
| **S2_GD2017_2_manure_scaffold_3_409|GD2017-2_manure_QB3_180125_Potentially_complete_Phage_48_40|GD2017-2_manure_QB3_180125** | LacPavin_0718_WC45_scaffold_3_117|LacPavin_0718_WC45_UNK|LacPavin_0718_WC45 | 46.654 | 1061 | 561 | 4 | 11 | 1069 | 9 | 1066 | 0.0 | 1001 |
| **S2_GD2017_2_manure_scaffold_3_409|GD2017-2_manure_QB3_180125_Potentially_complete_Phage_48_40|GD2017-2_manure_QB3_180125** | ERMZT366_2_scaffold_1600_1|ERMZT366_2_UNK|ERMZT366_2 | 46.569 | 1020 | 537 | 5 | 10 | 1023 | 7 | 1024 | 0.0 | 977 |
| **S2_GD2017_2_manure_scaffold_3_409|GD2017-2_manure_QB3_180125_Potentially_complete_Phage_48_40|GD2017-2_manure_QB3_180125** | AB_072018_0_1um_scaffold_612_15|AB_072018_0_1um_UNK|AB_072018_0_1um | 44.863 | 1061 | 574 | 5 | 11 | 1069 | 9 | 1060 | 0.0 | 972 |
| **S2_GD2017_2_manure_scaffold_3_409|GD2017-2_manure_QB3_180125_Potentially_complete_Phage_48_40|GD2017-2_manure_QB3_180125** | AB_082018_0_1um_scaffold_2553_3|AB_082018_0_1um_UNK|AB_082018_0_1um | 44.769 | 1061 | 575 | 5 | 11 | 1069 | 9 | 1060 | 0.0 | 969 |
| **S2_GD2017_2_manure_scaffold_3_409|GD2017-2_manure_QB3_180125_Potentially_complete_Phage_48_40|GD2017-2_manure_QB3_180125** | AB_092018_0_1um_scaffold_3669_7|AB_092018_0_1um_UNK|AB_092018_0_1um | 44.769 | 1061 | 575 | 5 | 11 | 1069 | 9 | 1060 | 0.0 | 969 |
| **S2_GD2017_2_manure_scaffold_3_409|GD2017-2_manure_QB3_180125_Potentially_complete_Phage_48_40|GD2017-2_manure_QB3_180125** | FFC_04162018_0_1um_scaffold_1491_2|FFC_04162018_0_1um_UNK|FFC_04162018_0_1um | 44.655 | 1057 | 574 | 5 | 11 | 1065 | 9 | 1056 | 0.0 | 964 |
| **S2_GD2017_2_manure_scaffold_3_409|GD2017-2_manure_QB3_180125_Potentially_complete_Phage_48_40|GD2017-2_manure_QB3_180125** | FFC_04162018_0_1um_scaffold_562_4|FFC_04162018_0_1um_UNK|FFC_04162018_0_1um | 45.292 | 1062 | 566 | 7 | 11 | 1069 | 9 | 1058 | 0.0 | 961 |
| **S2_GD2017_2_manure_scaffold_3_409|GD2017-2_manure_QB3_180125_Potentially_complete_Phage_48_40|GD2017-2_manure_QB3_180125** | BML_coassembly_scaffold_632_30|BML_coassembly_UNK|BML_coassembly | 44.539 | 1062 | 581 | 5 | 11 | 1069 | 10 | 1066 | 0.0 | 948 |
| **S2_GD2017_2_manure_scaffold_3_409|GD2017-2_manure_QB3_180125_Potentially_complete_Phage_48_40|GD2017-2_manure_QB3_180125** | SR-VP_0-2_scaffold_141_6448357_9|SR-VP_0-2cm_UNK|SR-VP_0-2cm | 43.425 | 1057 | 594 | 3 | 16 | 1069 | 14 | 1069 | 0.0 | 935 |
| **S2_GD2017_2_manure_scaffold_3_409|GD2017-2_manure_QB3_180125_Potentially_complete_Phage_48_40|GD2017-2_manure_QB3_180125** | BC_09192017_0_5m_scaffold_204_33|BC_09192017_0_5m_UNK|BC_09192017_0_5m | 44.977 | 1065 | 564 | 9 | 13 | 1069 | 10 | 1060 | 0.0 | 930 |
| **S2_GD2017_2_manure_scaffold_3_409|GD2017-2_manure_QB3_180125_Potentially_complete_Phage_48_40|GD2017-2_manure_QB3_180125** | LacPavin_0419_WC53_scaffold_182236_12|LacPavin_0419_WC53_UNK|LacPavin_0419_WC53 | 44.026 | 1063 | 579 | 9 | 13 | 1069 | 10 | 1062 | 0.0 | 923 |
| **S2_GD2017_2_manure_scaffold_3_409|GD2017-2_manure_QB3_180125_Potentially_complete_Phage_48_40|GD2017-2_manure_QB3_180125** | LacPavin_0818_WC40_scaffold_227017_5|LacPavin_0818_WC40_UNK|LacPavin_0818_WC40 | 44.026 | 1063 | 579 | 9 | 13 | 1069 | 10 | 1062 | 0.0 | 923 |
| **S2_GD2017_2_manure_scaffold_3_409|GD2017-2_manure_QB3_180125_Potentially_complete_Phage_48_40|GD2017-2_manure_QB3_180125** | P0_An_pond3_S8_coassembly_k141_276347_2|P0_An_pond3_S8_coassembly_UNK|E_GD2017-1_P0_An_pond3_S8_Biohub_coassembly | 43.274 | 1063 | 585 | 7 | 13 | 1068 | 15 | 1066 | 0.0 | 921 |
| **S2_GD2017_2_manure_scaffold_3_409|GD2017-2_manure_QB3_180125_Potentially_complete_Phage_48_40|GD2017-2_manure_QB3_180125** | P0_An_GD2017L_S7_coassembly_k141_961924_7|P0_An_GD2017L_S7_coassembly_UNK|E_P0_An_GD2017L_S7_coassembly | 43.274 | 1063 | 585 | 7 | 13 | 1068 | 15 | 1066 | 0.0 | 921 |
| **S2_GD2017_2_manure_scaffold_3_409|GD2017-2_manure_QB3_180125_Potentially_complete_Phage_48_40|GD2017-2_manure_QB3_180125** | P0_An_pond3_S8_170907_scaffold_875447_2|E_GD2017-1_P0_AN_POND3_S8_BIOHUB_170907_UNK|E_GD2017-1_P0_An_pond3_S8_Biohub_170907 | 43.274 | 1063 | 585 | 7 | 13 | 1068 | 15 | 1066 | 0.0 | 921 |
| **S2_GD2017_2_manure_scaffold_3_409|GD2017-2_manure_QB3_180125_Potentially_complete_Phage_48_40|GD2017-2_manure_QB3_180125** | P0_An_GD2017L_S7_170907_scaffold_2243732_2|E_GD2017-1_P0_An_GD2017L_S7_Biohub_170907_UNK|E_GD2017-1_P0_An_GD2017L_S7_Biohub_170907 | 43.274 | 1063 | 585 | 7 | 13 | 1068 | 15 | 1066 | 0.0 | 921 |
| **S2_GD2017_2_manure_scaffold_3_409|GD2017-2_manure_QB3_180125_Potentially_complete_Phage_48_40|GD2017-2_manure_QB3_180125** | LacPavin_0419_WC70S_scaffold_29687_53|LacPavin_0419_WC70S_UNK|LacPavin_0419_WC70S | 43.650 | 1063 | 588 | 6 | 12 | 1071 | 9 | 1063 | 0.0 | 916 |
| **S2_GD2017_2_manure_scaffold_3_409|GD2017-2_manure_QB3_180125_Potentially_complete_Phage_48_40|GD2017-2_manure_QB3_180125** | LacPavin_0419_WC53_scaffold_459219_54|LacPavin_0419_WC53_UNK|LacPavin_0419_WC53 | 43.650 | 1063 | 588 | 6 | 12 | 1071 | 9 | 1063 | 0.0 | 916 |
| **S2_GD2017_2_manure_scaffold_3_409|GD2017-2_manure_QB3_180125_Potentially_complete_Phage_48_40|GD2017-2_manure_QB3_180125** | LacPavin_0818_WC40_scaffold_701884_6|LacPavin_0818_WC40_UNK|LacPavin_0818_WC40 | 43.650 | 1063 | 588 | 6 | 12 | 1071 | 9 | 1063 | 0.0 | 916 |
| **S2_GD2017_2_manure_scaffold_3_409|GD2017-2_manure_QB3_180125_Potentially_complete_Phage_48_40|GD2017-2_manure_QB3_180125** | LacPavin_0818_WC40_scaffold_164700_3|LacPavin_0818_WC40_UNK|LacPavin_0818_WC40 | 44.477 | 1014 | 552 | 5 | 58 | 1069 | 21 | 1025 | 0.0 | 909 |
| **S2_GD2017_2_manure_scaffold_3_409|GD2017-2_manure_QB3_180125_Potentially_complete_Phage_48_40|GD2017-2_manure_QB3_180125** | SR-VP_2-4_scaffold_141_5320225_1|SR-VP_2-4cm_UNK|SR-VP_2-4cm | 68.354 | 632 | 200 | 0 | 267 | 898 | 1 | 632 | 0.0 | 901 |
| **S2_GD2017_2_manure_scaffold_3_409|GD2017-2_manure_QB3_180125_Potentially_complete_Phage_48_40|GD2017-2_manure_QB3_180125** | 3300005805_____Ga0079957_1000004_111|imgvr_subset_UNK|imgvr_subset | 43.535 | 1075 | 567 | 12 | 13 | 1069 | 10 | 1062 | 0.0 | 900 |
| **S2_GD2017_2_manure_scaffold_3_409|GD2017-2_manure_QB3_180125_Potentially_complete_Phage_48_40|GD2017-2_manure_QB3_180125** | SR-VP_4-6_scaffold_141_6084653_1|SR-VP_4-6cm_Biohub_180515_UNK|SR-VP_4-6cm_Biohub_180515 | 84.124 | 485 | 77 | 0 | 55 | 539 | 1 | 485 | 0.0 | 887 |
| **S2_GD2017_2_manure_scaffold_3_409|GD2017-2_manure_QB3_180125_Potentially_complete_Phage_48_40|GD2017-2_manure_QB3_180125** | PAFVLPS_2018_scaffold_386_6|viral-cat_UNK|viral-cat | 42.239 | 1063 | 601 | 6 | 12 | 1069 | 9 | 1063 | 0.0 | 866 |
| **S2_GD2017_2_manure_scaffold_3_409|GD2017-2_manure_QB3_180125_Potentially_complete_Phage_48_40|GD2017-2_manure_QB3_180125** | FFC_07242016_10_scaffold_7883_1|FFC_07242016_10_UNK|FFC_07242016_10 | 72.408 | 569 | 157 | 0 | 273 | 841 | 1 | 569 | 0.0 | 865 |
| **S2_GD2017_2_manure_scaffold_3_409|GD2017-2_manure_QB3_180125_Potentially_complete_Phage_48_40|GD2017-2_manure_QB3_180125** | LacPavin_0419_WC53_scaffold_515305_1|LacPavin_0419_WC53_UNK|LacPavin_0419_WC53 | 43.198 | 963 | 536 | 5 | 109 | 1069 | 1 | 954 | 0.0 | 829 |
| **S2_GD2017_2_manure_scaffold_3_409|GD2017-2_manure_QB3_180125_Potentially_complete_Phage_48_40|GD2017-2_manure_QB3_180125** | PLM4_65_b1_redo_sep16_scaffold_45075_3|PLM4_65cm_b1_redo_sep2016_UNK|PLM4_65cm_b1_redo_sep2016 | 42.797 | 944 | 536 | 3 | 13 | 954 | 11 | 952 | 0.0 | 805 |
| **S2_GD2017_2_manure_scaffold_3_409|GD2017-2_manure_QB3_180125_Potentially_complete_Phage_48_40|GD2017-2_manure_QB3_180125** | LacPavin_0419_WC70S_scaffold_436490_10|LacPavin_0419_WC70S_UNK|LacPavin_0419_WC70S | 41.271 | 991 | 560 | 11 | 89 | 1071 | 414 | 1390 | 0.0 | 772 |
| **S2_GD2017_2_manure_scaffold_3_409|GD2017-2_manure_QB3_180125_Potentially_complete_Phage_48_40|GD2017-2_manure_QB3_180125** | SR-VP_0-2_scaffold_141_4769078_1|SR-VP_0-2cm_UNK|SR-VP_0-2cm | 39.312 | 1076 | 610 | 15 | 17 | 1071 | 5 | 1058 | 0.0 | 770 |
| **S2_GD2017_2_manure_scaffold_3_409|GD2017-2_manure_QB3_180125_Potentially_complete_Phage_48_40|GD2017-2_manure_QB3_180125** | SR-VP_4-6_scaffold_141_3196289_1|SR-VP_4-6cm_Biohub_180515_UNK|SR-VP_4-6cm_Biohub_180515 | 71.456 | 529 | 150 | 1 | 544 | 1071 | 1 | 529 | 0.0 | 769 |
| **S2_GD2017_2_manure_scaffold_3_409|GD2017-2_manure_QB3_180125_Potentially_complete_Phage_48_40|GD2017-2_manure_QB3_180125** | SR-VP_4-6_scaffold_141_4681429_2|SR-VP_4-6cm_Biohub_180515_UNK|SR-VP_4-6cm_Biohub_180515 | 38.555 | 1066 | 630 | 13 | 15 | 1071 | 11 | 1060 | 0.0 | 760 |
| **S2_GD2017_2_manure_scaffold_3_409|GD2017-2_manure_QB3_180125_Potentially_complete_Phage_48_40|GD2017-2_manure_QB3_180125** | FFC_092018_0_1um_scaffold_17_166|FFC_092018_0_1um_partial_phage_31_10|FFC_092018_0_1um | 39.624 | 1065 | 620 | 12 | 11 | 1069 | 1 | 1048 | 0.0 | 759 |
| **S2_GD2017_2_manure_scaffold_3_409|GD2017-2_manure_QB3_180125_Potentially_complete_Phage_48_40|GD2017-2_manure_QB3_180125** | L3m1_full_idba_ud_scaffold_1665_1|L3m1_UNK|L3m1 | 41.195 | 937 | 546 | 3 | 137 | 1071 | 2 | 935 | 0.0 | 757 |
| **S2_GD2017_2_manure_scaffold_3_409|GD2017-2_manure_QB3_180125_Potentially_complete_Phage_48_40|GD2017-2_manure_QB3_180125** | Salt_Pond_R1_A_D2_MG_scaffold_231_6|JGI_Salt_Pond_R1_A_D2_MG_UNK|JGI_Salt_Pond_R1_A_D2_MG | 37.899 | 1066 | 631 | 12 | 17 | 1067 | 5 | 1054 | 0.0 | 754 |
| **S2_GD2017_2_manure_scaffold_3_409|GD2017-2_manure_QB3_180125_Potentially_complete_Phage_48_40|GD2017-2_manure_QB3_180125** | Salt_Pond_R1_C_D1_MG_scaffold_2500_2|JGI_Salt_Pond_R1_C_D1_MG_UNK|JGI_Salt_Pond_R1_C_D1_MG | 37.899 | 1066 | 631 | 12 | 17 | 1067 | 5 | 1054 | 0.0 | 753 |
| **S2_GD2017_2_manure_scaffold_3_409|GD2017-2_manure_QB3_180125_Potentially_complete_Phage_48_40|GD2017-2_manure_QB3_180125** | Salt_Pond_R1_A_D1_MG_scaffold_1858_5|Salt_Pond_R1_A_D1_MG_UNK|JGI_Salt_Pond_R1_A_D1_MG | 37.899 | 1066 | 631 | 12 | 17 | 1067 | 5 | 1054 | 0.0 | 753 |
| **S2_GD2017_2_manure_scaffold_3_409|GD2017-2_manure_QB3_180125_Potentially_complete_Phage_48_40|GD2017-2_manure_QB3_180125** | BML_08182015_1_5m_scaffold_13_140|BML_08182015_1_5m_UNK|BML_08182015_1_5m | 38.354 | 1069 | 626 | 14 | 10 | 1069 | 10 | 1054 | 0.0 | 746 |
| **S2_GD2017_2_manure_scaffold_3_409|GD2017-2_manure_QB3_180125_Potentially_complete_Phage_48_40|GD2017-2_manure_QB3_180125** | BML_coassembly_scaffold_125_135|BML_coassembly_UNK|BML_coassembly | 38.354 | 1069 | 626 | 14 | 10 | 1069 | 10 | 1054 | 0.0 | 746 |
| **S2_GD2017_2_manure_scaffold_3_409|GD2017-2_manure_QB3_180125_Potentially_complete_Phage_48_40|GD2017-2_manure_QB3_180125** | LacPavin_0818_WC55_scaffold_68513_31|LacPavin_0818_WC55_UNK|LacPavin_0818_WC55 | 38.433 | 1072 | 619 | 17 | 13 | 1069 | 2 | 1047 | 0.0 | 744 |
| **S2_GD2017_2_manure_scaffold_3_409|GD2017-2_manure_QB3_180125_Potentially_complete_Phage_48_40|GD2017-2_manure_QB3_180125** | LacPavin_0718_WC55_scaffold_501_23|LacPavin_0718_WC55_UNK|LacPavin_0718_WC55 | 38.433 | 1072 | 619 | 17 | 13 | 1069 | 2 | 1047 | 0.0 | 744 |
| **S2_GD2017_2_manure_scaffold_3_409|GD2017-2_manure_QB3_180125_Potentially_complete_Phage_48_40|GD2017-2_manure_QB3_180125** | LacPavin_0818_WC45_scaffold_283915_26|LacPavin_0818_WC45_UNK|LacPavin_0818_WC45 | 38.433 | 1072 | 619 | 17 | 13 | 1069 | 2 | 1047 | 0.0 | 744 |
| **S2_GD2017_2_manure_scaffold_3_409|GD2017-2_manure_QB3_180125_Potentially_complete_Phage_48_40|GD2017-2_manure_QB3_180125** | BC_09192017_0_5m_scaffold_7473_3|BC_09192017_0_5m_UNK|BC_09192017_0_5m | 42.659 | 865 | 489 | 3 | 205 | 1069 | 1 | 858 | 0.0 | 739 |
| **S2_GD2017_2_manure_scaffold_3_409|GD2017-2_manure_QB3_180125_Potentially_complete_Phage_48_40|GD2017-2_manure_QB3_180125** | OR_07232018_dam_0_1um_scaffold_14_256|OR_07232018_dam_0_1um_partial_phage_30_17|OR_07232018_dam_0_1um | 39.151 | 1083 | 603 | 15 | 11 | 1071 | 1 | 1049 | 0.0 | 738 |
| **S2_GD2017_2_manure_scaffold_3_409|GD2017-2_manure_QB3_180125_Potentially_complete_Phage_48_40|GD2017-2_manure_QB3_180125** | BC_09192017_0_5m_scaffold_2747_7|BC_09192017_0_5m_UNK|BC_09192017_0_5m | 41.481 | 945 | 529 | 13 | 135 | 1069 | 77 | 1007 | 0.0 | 738 |
| **S2_GD2017_2_manure_scaffold_3_409|GD2017-2_manure_QB3_180125_Potentially_complete_Phage_48_40|GD2017-2_manure_QB3_180125** | P0_An_pond3_S8_coassembly_k141_4231480_1|P0_An_pond3_S8_coassembly_UNK|E_GD2017-1_P0_An_pond3_S8_Biohub_coassembly | 54.677 | 620 | 274 | 1 | 11 | 630 | 10 | 622 | 0.0 | 737 |
| **S2_GD2017_2_manure_scaffold_3_409|GD2017-2_manure_QB3_180125_Potentially_complete_Phage_48_40|GD2017-2_manure_QB3_180125** | AB_082018_0_1um_scaffold_2922_2|AB_082018_0_1um_UNK|AB_082018_0_1um | 38.426 | 1080 | 600 | 20 | 17 | 1069 | 7 | 1048 | 0.0 | 736 |
| **S2_GD2017_2_manure_scaffold_3_409|GD2017-2_manure_QB3_180125_Potentially_complete_Phage_48_40|GD2017-2_manure_QB3_180125** | AB_072018_0_1um_scaffold_4422_4|AB_072018_0_1um_UNK|AB_072018_0_1um | 38.404 | 1078 | 599 | 20 | 19 | 1069 | 1 | 1040 | 0.0 | 734 |
| **S2_GD2017_2_manure_scaffold_3_409|GD2017-2_manure_QB3_180125_Potentially_complete_Phage_48_40|GD2017-2_manure_QB3_180125** | ALT_082018_0_1um_scaffold_7999_4|ALT_082018_0_1um_UNK|ALT_082018_0_1um | 38.082 | 1074 | 622 | 14 | 12 | 1069 | 3 | 1049 | 0.0 | 734 |
| **S2_GD2017_2_manure_scaffold_3_409|GD2017-2_manure_QB3_180125_Potentially_complete_Phage_48_40|GD2017-2_manure_QB3_180125** | LacPavin_0818_WC40_scaffold_811308_1|LacPavin_0818_WC40_UNK|LacPavin_0818_WC40 | 46.745 | 768 | 398 | 5 | 11 | 776 | 9 | 767 | 0.0 | 734 |
| **S2_GD2017_2_manure_scaffold_3_409|GD2017-2_manure_QB3_180125_Potentially_complete_Phage_48_40|GD2017-2_manure_QB3_180125** | GS605_0p1_scaffold_1053_23|lsdeep_GS605_0p1_UNK|lsdeep_GS605_0p1 | 38.396 | 1060 | 629 | 13 | 13 | 1069 | 12 | 1050 | 0.0 | 732 |
| **S2_GD2017_2_manure_scaffold_3_409|GD2017-2_manure_QB3_180125_Potentially_complete_Phage_48_40|GD2017-2_manure_QB3_180125** | FFC_04162018_0_1um_scaffold_557_3|FFC_04162018_0_1um_UNK|FFC_04162018_0_1um | 38.296 | 1068 | 626 | 18 | 13 | 1069 | 2 | 1047 | 0.0 | 731 |
| **S2_GD2017_2_manure_scaffold_3_409|GD2017-2_manure_QB3_180125_Potentially_complete_Phage_48_40|GD2017-2_manure_QB3_180125** | LacPavin_0818_WC55_scaffold_896135_3|LacPavin_0818_WC55_UNK|LacPavin_0818_WC55 | 37.372 | 1073 | 633 | 15 | 11 | 1069 | 1 | 1048 | 0.0 | 723 |
| **S2_GD2017_2_manure_scaffold_3_409|GD2017-2_manure_QB3_180125_Potentially_complete_Phage_48_40|GD2017-2_manure_QB3_180125** | S15_GE15_scaffold_591272_2|E_GD2017-2_urea-2_S15_GE15_Biohub_170515_UNK|E_GD2017-2_urea-2_S15_GE15_Biohub_180515 | 57.491 | 574 | 244 | 0 | 11 | 584 | 10 | 583 | 0.0 | 723 |
| **S2_GD2017_2_manure_scaffold_3_409|GD2017-2_manure_QB3_180125_Potentially_complete_Phage_48_40|GD2017-2_manure_QB3_180125** | LacPavin_0818_WC40_scaffold_309612_50|LacPavin_0818_WC40_UNK|LacPavin_0818_WC40 | 37.373 | 1081 | 617 | 15 | 11 | 1067 | 12 | 1056 | 0.0 | 721 |
| **S2_GD2017_2_manure_scaffold_3_409|GD2017-2_manure_QB3_180125_Potentially_complete_Phage_48_40|GD2017-2_manure_QB3_180125** | SR-VP_0-2_scaffold_141_2719384_2|SR-VP_0-2cm_UNK|SR-VP_0-2cm | 76.688 | 459 | 107 | 0 | 613 | 1071 | 3 | 461 | 0.0 | 721 |
| **S2_GD2017_2_manure_scaffold_3_409|GD2017-2_manure_QB3_180125_Potentially_complete_Phage_48_40|GD2017-2_manure_QB3_180125** | GS605_0p1_scaffold_738_22|lsdeep_GS605_0p1_UNK|lsdeep_GS605_0p1 | 38.665 | 1094 | 582 | 23 | 13 | 1071 | 13 | 1052 | 0.0 | 717 |
| **S2_GD2017_2_manure_scaffold_3_409|GD2017-2_manure_QB3_180125_Potentially_complete_Phage_48_40|GD2017-2_manure_QB3_180125** | Salt_Pond_R2_restored_H2O_MG_scaffold_42_100|JGI_Salt_Pond_R2_restored_H2O_MG_UNK|JGI_Salt_Pond_R2_restored_H2O_MG | 36.739 | 1067 | 649 | 12 | 11 | 1071 | 1 | 1047 | 0.0 | 717 |
| **S2_GD2017_2_manure_scaffold_3_409|GD2017-2_manure_QB3_180125_Potentially_complete_Phage_48_40|GD2017-2_manure_QB3_180125** | LacPavin_0419_WC53_scaffold_84377_3|LacPavin_0419_WC53_UNK|LacPavin_0419_WC53 | 38.015 | 1068 | 629 | 13 | 11 | 1069 | 8 | 1051 | 0.0 | 716 |
| **S2_GD2017_2_manure_scaffold_3_408|GD2017-2_manure_QB3_180125_Potentially_complete_Phage_48_40|GD2017-2_manure_QB3_180125** | GD18-4_manure_scaffold_876276_7|GD2018-4_manure_QB3_180703_UNK|GD2018-4_manure_QB3_180703 | 100.000 | 316 | 0 | 0 | 1 | 316 | 2 | 317 | 0.0 | 656 |
| **S2_GD2017_2_manure_scaffold_3_408|GD2017-2_manure_QB3_180125_Potentially_complete_Phage_48_40|GD2017-2_manure_QB3_180125** | S27_BME27_629333_prodigal-single_186|BM_PHAGE_48_13|BM_2017_Strous_6 | 100.000 | 316 | 0 | 0 | 1 | 316 | 2 | 317 | 0.0 | 656 |
| **S2_GD2017_2_manure_scaffold_3_408|GD2017-2_manure_QB3_180125_Potentially_complete_Phage_48_40|GD2017-2_manure_QB3_180125** | S22_GE22_scaffold_285059_prodigal-single_513|GD_PHAGE_COMPLETE_48_49|E_GD2017-2_anammox-7_S22_GE22_Biohub_180515 | 100.000 | 316 | 0 | 0 | 1 | 316 | 2 | 317 | 0.0 | 656 |
| **S2_GD2017_2_manure_scaffold_3_408|GD2017-2_manure_QB3_180125_Potentially_complete_Phage_48_40|GD2017-2_manure_QB3_180125** | P0_An_GD2017L_S7_coassembly_k141_1013362_prodigal-single_81|GD_PHAGE_48_49|E_P0_An_GD2017L_S7_coassembly | 100.000 | 316 | 0 | 0 | 1 | 316 | 2 | 317 | 0.0 | 656 |
| **S2_GD2017_2_manure_scaffold_3_408|GD2017-2_manure_QB3_180125_Potentially_complete_Phage_48_40|GD2017-2_manure_QB3_180125** | S19_GE19_scaffold_2259_prodigal-single_282|GD_PHAGE_48_10|E_GD2017-2_strous-5-pellet_S19_GE19_Biohub_180515 | 100.000 | 316 | 0 | 0 | 1 | 316 | 2 | 317 | 0.0 | 656 |
| **S2_GD2017_2_manure_scaffold_3_408|GD2017-2_manure_QB3_180125_Potentially_complete_Phage_48_40|GD2017-2_manure_QB3_180125** | S2_GD2017_2_manure_scaffold_3_prodigal-single_481|GD_PHAGE_COMPLETE_48_40|GD2017-2_manure_QB3_180125 | 100.000 | 316 | 0 | 0 | 1 | 316 | 2 | 317 | 0.0 | 656 |
| **S2_GD2017_2_manure_scaffold_3_408|GD2017-2_manure_QB3_180125_Potentially_complete_Phage_48_40|GD2017-2_manure_QB3_180125** | S30_BME30_294872_49|BM_2017_Coates_4_Potentially_Complete_Phage_48-32|BM_2017_Coates_4 | 100.000 | 316 | 0 | 0 | 1 | 316 | 1 | 316 | 0.0 | 655 |
| **S2_GD2017_2_manure_scaffold_3_408|GD2017-2_manure_QB3_180125_Potentially_complete_Phage_48_40|GD2017-2_manure_QB3_180125** | S28_BME28_138683_396|BM_2017_Strous_8_UNK|BM_2017_Strous_8 | 100.000 | 316 | 0 | 0 | 1 | 316 | 1 | 316 | 0.0 | 655 |
| **S2_GD2017_2_manure_scaffold_3_408|GD2017-2_manure_QB3_180125_Potentially_complete_Phage_48_40|GD2017-2_manure_QB3_180125** | S27_BME27_629333_176|BM_2017_Strous_6_Phage_48_13|BM_2017_Strous_6 | 100.000 | 316 | 0 | 0 | 1 | 316 | 1 | 316 | 0.0 | 655 |
| **S2_GD2017_2_manure_scaffold_3_408|GD2017-2_manure_QB3_180125_Potentially_complete_Phage_48_40|GD2017-2_manure_QB3_180125** | S22_GE22_scaffold_285059_436|E_GD2017-2_anammox-7_S22_GE22_Biohub_170515_Potentially_Complete_48_49|E_GD2017-2_anammox-7_S22_GE22_Biohub_180515 | 100.000 | 316 | 0 | 0 | 1 | 316 | 1 | 316 | 0.0 | 655 |
| **S2_GD2017_2_manure_scaffold_3_408|GD2017-2_manure_QB3_180125_Potentially_complete_Phage_48_40|GD2017-2_manure_QB3_180125** | S20_GE20_scaffold_396645_12|E_GD2017-2_strous-5_S20_GE20_Biohub_170515_UNK|E_GD2017-2_strous-5_S20_GE20_Biohub_180515 | 100.000 | 316 | 0 | 0 | 1 | 316 | 1 | 316 | 0.0 | 655 |
| **S2_GD2017_2_manure_scaffold_3_408|GD2017-2_manure_QB3_180125_Potentially_complete_Phage_48_40|GD2017-2_manure_QB3_180125** | S15_GE15_scaffold_320047_244|E_GD2017-2_urea-2_S15_GE15_Biohub_170515_UNK|E_GD2017-2_urea-2_S15_GE15_Biohub_180515 | 100.000 | 316 | 0 | 0 | 1 | 316 | 1 | 316 | 0.0 | 655 |
| **S2_GD2017_2_manure_scaffold_3_408|GD2017-2_manure_QB3_180125_Potentially_complete_Phage_48_40|GD2017-2_manure_QB3_180125** | S14_GE14_scaffold_311130_14|E_GD2017-2_urea-2_S14_GE14_Biohub_170515_UNK|E_GD2017-2_urea-2_S14_GE14_Biohub_180515 | 100.000 | 316 | 0 | 0 | 1 | 316 | 1 | 316 | 0.0 | 655 |
| **S2_GD2017_2_manure_scaffold_3_408|GD2017-2_manure_QB3_180125_Potentially_complete_Phage_48_40|GD2017-2_manure_QB3_180125** | P0_An_pond3_S8_coassembly_k141_2723617_435|P0_An_pond3_S8_coassembly_UNK|E_GD2017-1_P0_An_pond3_S8_Biohub_coassembly | 100.000 | 316 | 0 | 0 | 1 | 316 | 1 | 316 | 0.0 | 655 |
| **S2_GD2017_2_manure_scaffold_3_408|GD2017-2_manure_QB3_180125_Potentially_complete_Phage_48_40|GD2017-2_manure_QB3_180125** | P0_An_GD2017L_S7_coassembly_k141_1013362_75|P0_An_GD2017L_S7_coassembly_Phage_48_49|E_P0_An_GD2017L_S7_coassembly | 100.000 | 316 | 0 | 0 | 1 | 316 | 1 | 316 | 0.0 | 655 |
| **S2_GD2017_2_manure_scaffold_3_408|GD2017-2_manure_QB3_180125_Potentially_complete_Phage_48_40|GD2017-2_manure_QB3_180125** | S19_GE19_scaffold_2259_258|E_GD2017-2_strous-5-pellet_S19_GE19_Biohub_170515_Phage_48_10|E_GD2017-2_strous-5-pellet_S19_GE19_Biohub_180515 | 100.000 | 316 | 0 | 0 | 1 | 316 | 1 | 316 | 0.0 | 655 |
| **S2_GD2017_2_manure_scaffold_3_408|GD2017-2_manure_QB3_180125_Potentially_complete_Phage_48_40|GD2017-2_manure_QB3_180125** | S18_GE18_scaffold_616449_289|E_GD2017-2_strous-5-prefilter_S18_GE18_Biohub_170515_UNK|E_GD2017-2_strous-5-prefilter_S18_GE18_Biohub_180515 | 100.000 | 316 | 0 | 0 | 1 | 316 | 1 | 316 | 0.0 | 655 |
| **S2_GD2017_2_manure_scaffold_3_408|GD2017-2_manure_QB3_180125_Potentially_complete_Phage_48_40|GD2017-2_manure_QB3_180125** | S16_GE16_scaffold_390213_389|E_GD2017-2_urea-3_S16_GE16_Biohub_170515_UNK|E_GD2017-2_urea-3_S16_GE16_Biohub_180515 | 100.000 | 316 | 0 | 0 | 1 | 316 | 1 | 316 | 0.0 | 655 |
| **S2_GD2017_2_manure_scaffold_3_408|GD2017-2_manure_QB3_180125_Potentially_complete_Phage_48_40|GD2017-2_manure_QB3_180125** | P0_An_pond3_S8_170907_scaffold_1005865_40|E_GD2017-1_P0_AN_POND3_S8_BIOHUB_170907_UNK|E_GD2017-1_P0_An_pond3_S8_Biohub_170907 | 100.000 | 316 | 0 | 0 | 1 | 316 | 1 | 316 | 0.0 | 655 |
| **S2_GD2017_2_manure_scaffold_3_408|GD2017-2_manure_QB3_180125_Potentially_complete_Phage_48_40|GD2017-2_manure_QB3_180125** | P0_An_GD2017L_S7_170907_scaffold_359857_23|E_GD2017-1_P0_An_GD2017L_S7_Biohub_170907_UNK|E_GD2017-1_P0_An_GD2017L_S7_Biohub_170907 | 100.000 | 316 | 0 | 0 | 1 | 316 | 1 | 316 | 0.0 | 655 |
| **S2_GD2017_2_manure_scaffold_3_408|GD2017-2_manure_QB3_180125_Potentially_complete_Phage_48_40|GD2017-2_manure_QB3_180125** | S2_GD2017_2_manure_scaffold_3_408|GD2017-2_manure_QB3_180125_Potentially_complete_Phage_48_40|GD2017-2_manure_QB3_180125 | 100.000 | 316 | 0 | 0 | 1 | 316 | 1 | 316 | 0.0 | 655 |
| **S2_GD2017_2_manure_scaffold_3_408|GD2017-2_manure_QB3_180125_Potentially_complete_Phage_48_40|GD2017-2_manure_QB3_180125** | SR-VP_4-6_scaffold_141_1485620_4|SR-VP_4-6cm_Biohub_180515_UNK|SR-VP_4-6cm_Biohub_180515 | 65.190 | 316 | 110 | 0 | 1 | 316 | 2 | 317 | 1.65E-162 | 465 |
| **S2_GD2017_2_manure_scaffold_3_408|GD2017-2_manure_QB3_180125_Potentially_complete_Phage_48_40|GD2017-2_manure_QB3_180125** | SR-VP_0-2_scaffold_141_2432070_13|SR-VP_0-2cm_UNK|SR-VP_0-2cm | 65.190 | 316 | 110 | 0 | 1 | 316 | 2 | 317 | 1.65E-162 | 465 |
| **S2_GD2017_2_manure_scaffold_3_408|GD2017-2_manure_QB3_180125_Potentially_complete_Phage_48_40|GD2017-2_manure_QB3_180125** | ALT_082018_0_1um_scaffold_60661_1|ALT_082018_0_1um_UNK|ALT_082018_0_1um | 65.823 | 316 | 106 | 1 | 1 | 316 | 2 | 315 | 1.54E-159 | 458 |
| **S2_GD2017_2_manure_scaffold_3_408|GD2017-2_manure_QB3_180125_Potentially_complete_Phage_48_40|GD2017-2_manure_QB3_180125** | L3a2_full_idba_ud_scaffold_20020_5|L3a2_UNK|L3a2 | 64.984 | 317 | 110 | 1 | 1 | 316 | 2 | 318 | 7.46E-157 | 451 |
| **S2_GD2017_2_manure_scaffold_3_408|GD2017-2_manure_QB3_180125_Potentially_complete_Phage_48_40|GD2017-2_manure_QB3_180125** | L3a1_full_idba_ud_scaffold_6_225|L3a1_UNK|L3a1 | 64.984 | 317 | 110 | 1 | 1 | 316 | 2 | 318 | 7.46E-157 | 451 |
| **S2_GD2017_2_manure_scaffold_3_408|GD2017-2_manure_QB3_180125_Potentially_complete_Phage_48_40|GD2017-2_manure_QB3_180125** | SR-VP_2-4_scaffold_141_6534590_1|SR-VP_2-4cm_UNK|SR-VP_2-4cm | 60.784 | 306 | 112 | 3 | 1 | 304 | 2 | 301 | 3.7E-133 | 390 |
| **S2_GD2017_2_manure_scaffold_3_408|GD2017-2_manure_QB3_180125_Potentially_complete_Phage_48_40|GD2017-2_manure_QB3_180125** | ERMGT642_2_scaffold_16862_2|ERMGT642_2_UNK|ERMGT642_2 | 60.377 | 318 | 118 | 3 | 1 | 316 | 2 | 313 | 5.59E-127 | 375 |
| **S2_GD2017_2_manure_scaffold_3_408|GD2017-2_manure_QB3_180125_Potentially_complete_Phage_48_40|GD2017-2_manure_QB3_180125** | LacPavin_0419_WC70S_scaffold_727964_1|LacPavin_0419_WC70S_UNK|LacPavin_0419_WC70S | 66.359 | 217 | 73 | 0 | 1 | 217 | 2 | 218 | 2.11E-107 | 322 |
| **S2_GD2017_2_manure_scaffold_3_408|GD2017-2_manure_QB3_180125_Potentially_complete_Phage_48_40|GD2017-2_manure_QB3_180125** | S33_3P_scaffold_613888_1|Genasci_Feb2018_S33_3P_UNK|Genasci_Feb2018_S33_3P | 100.000 | 142 | 0 | 0 | 1 | 142 | 2 | 143 | 4.37E-98 | 295 |
| **S2_GD2017_2_manure_scaffold_3_408|GD2017-2_manure_QB3_180125_Potentially_complete_Phage_48_40|GD2017-2_manure_QB3_180125** | FFC_07242016_10_scaffold_11803_1|FFC_07242016_10_UNK|FFC_07242016_10 | 61.638 | 232 | 86 | 2 | 86 | 315 | 1 | 231 | 1.26E-93 | 288 |
| **S2_GD2017_2_manure_scaffold_3_408|GD2017-2_manure_QB3_180125_Potentially_complete_Phage_48_40|GD2017-2_manure_QB3_180125** | S15_GE15_scaffold_39268_60|E_GD2017-2_urea-2_S15_GE15_Biohub_170515_UNK|E_GD2017-2_urea-2_S15_GE15_Biohub_180515 | 45.794 | 321 | 159 | 4 | 1 | 313 | 2 | 315 | 1.49E-93 | 290 |
| **S2_GD2017_2_manure_scaffold_3_408|GD2017-2_manure_QB3_180125_Potentially_complete_Phage_48_40|GD2017-2_manure_QB3_180125** | P0_An_pond3_S8_coassembly_k141_1548338_2|P0_An_pond3_S8_coassembly_UNK|E_GD2017-1_P0_An_pond3_S8_Biohub_coassembly | 45.794 | 321 | 159 | 4 | 1 | 313 | 2 | 315 | 1.49E-93 | 290 |
| **S2_GD2017_2_manure_scaffold_3_408|GD2017-2_manure_QB3_180125_Potentially_complete_Phage_48_40|GD2017-2_manure_QB3_180125** | S16_GE16_scaffold_5545_prodigal-single_26|GD_PHAGE_46_9|E_GD2017-2_urea-3_S16_GE16_Biohub_180515 | 45.794 | 321 | 159 | 4 | 1 | 313 | 2 | 315 | 1.49E-93 | 290 |
| **S2_GD2017_2_manure_scaffold_3_408|GD2017-2_manure_QB3_180125_Potentially_complete_Phage_48_40|GD2017-2_manure_QB3_180125** | S16_GE16_scaffold_5545_25|E_GD2017-2_urea-3_S16_GE16_Biohub_170515_Phage-like_46_9|E_GD2017-2_urea-3_S16_GE16_Biohub_180515 | 45.794 | 321 | 159 | 4 | 1 | 313 | 2 | 315 | 1.49E-93 | 290 |
| **S2_GD2017_2_manure_scaffold_3_408|GD2017-2_manure_QB3_180125_Potentially_complete_Phage_48_40|GD2017-2_manure_QB3_180125** | PAFVLPS_2018_scaffold_33_41|circular_33|viral-cat | 44.728 | 313 | 165 | 4 | 1 | 313 | 2 | 306 | 2.41E-88 | 277 |
| **S2_GD2017_2_manure_scaffold_3_408|GD2017-2_manure_QB3_180125_Potentially_complete_Phage_48_40|GD2017-2_manure_QB3_180125** | SR-VP_0-2_scaffold_141_1614196_2|SR-VP_0-2cm_UNK|SR-VP_0-2cm | 47.937 | 315 | 155 | 6 | 5 | 314 | 6 | 316 | 1.04E-87 | 276 |
| **S2_GD2017_2_manure_scaffold_3_408|GD2017-2_manure_QB3_180125_Potentially_complete_Phage_48_40|GD2017-2_manure_QB3_180125** | PAFVLPS_2018_scaffold_595250_1|viral-cat_UNK|viral-cat | 54.585 | 229 | 98 | 2 | 1 | 229 | 2 | 224 | 2.83E-86 | 269 |
| **S2_GD2017_2_manure_scaffold_3_408|GD2017-2_manure_QB3_180125_Potentially_complete_Phage_48_40|GD2017-2_manure_QB3_180125** | ERMGT300_2_scaffold_18467_2|ERMGT300_2_UNK|ERMGT300_2 | 42.345 | 307 | 172 | 3 | 3 | 309 | 5 | 306 | 1.19E-82 | 263 |
| **S2_GD2017_2_manure_scaffold_3_408|GD2017-2_manure_QB3_180125_Potentially_complete_Phage_48_40|GD2017-2_manure_QB3_180125** | PAFVLPS_2018_J31_2_scaffold_3728_7|soil-virus-P15-J31-2018_UNK|soil-virus-P15-J31-2018 | 41.905 | 315 | 174 | 4 | 1 | 313 | 2 | 309 | 8.86E-82 | 260 |
| **S2_GD2017_2_manure_scaffold_3_408|GD2017-2_manure_QB3_180125_Potentially_complete_Phage_48_40|GD2017-2_manure_QB3_180125** | PAFVLPS_2018_scaffold_386_7|viral-cat_UNK|viral-cat | 42.006 | 319 | 179 | 4 | 1 | 316 | 2 | 317 | 3.82E-81 | 259 |
| **S2_GD2017_2_manure_scaffold_3_408|GD2017-2_manure_QB3_180125_Potentially_complete_Phage_48_40|GD2017-2_manure_QB3_180125** | SR-VP_4-6_scaffold_141_5507573_2|SR-VP_4-6cm_Biohub_180515_UNK|SR-VP_4-6cm_Biohub_180515 | 45.426 | 317 | 164 | 6 | 3 | 314 | 7 | 319 | 2.93E-80 | 257 |
| **S2_GD2017_2_manure_scaffold_3_408|GD2017-2_manure_QB3_180125_Potentially_complete_Phage_48_40|GD2017-2_manure_QB3_180125** | ERMZT366_2_scaffold_1600_2|ERMZT366_2_UNK|ERMZT366_2 | 43.137 | 306 | 168 | 3 | 5 | 310 | 7 | 306 | 1.08E-79 | 255 |
| **S2_GD2017_2_manure_scaffold_3_408|GD2017-2_manure_QB3_180125_Potentially_complete_Phage_48_40|GD2017-2_manure_QB3_180125** | PAFVLPS_2018_scaffold_178920_3|viral-cat_UNK|viral-cat | 42.089 | 316 | 167 | 8 | 3 | 313 | 4 | 308 | 3.55E-78 | 251 |
| **S2_GD2017_2_manure_scaffold_3_408|GD2017-2_manure_QB3_180125_Potentially_complete_Phage_48_40|GD2017-2_manure_QB3_180125** | PAFVLPS_2018_scaffold_7515_43|viral-cat_UNK|viral-cat | 41.009 | 317 | 180 | 4 | 3 | 316 | 4 | 316 | 6.98E-78 | 250 |
| **S2_GD2017_2_manure_scaffold_3_408|GD2017-2_manure_QB3_180125_Potentially_complete_Phage_48_40|GD2017-2_manure_QB3_180125** | BC_09192017_0_5m_scaffold_13069_5|BC_09192017_0_5m_UNK|BC_09192017_0_5m | 41.195 | 318 | 175 | 6 | 3 | 316 | 5 | 314 | 1E-77 | 250 |
| **S2_GD2017_2_manure_scaffold_3_408|GD2017-2_manure_QB3_180125_Potentially_complete_Phage_48_40|GD2017-2_manure_QB3_180125** | PAFVLPS_2018_scaffold_265627_3|viral-cat_UNK|viral-cat | 39.623 | 318 | 185 | 4 | 1 | 316 | 2 | 314 | 2.72E-77 | 249 |
| **S2_GD2017_2_manure_scaffold_3_408|GD2017-2_manure_QB3_180125_Potentially_complete_Phage_48_40|GD2017-2_manure_QB3_180125** | SR-VP_0-2_scaffold_141_5191495_prodigal-single_71|SR-VP_PHAGE_38_25|SR-VP_0-2cm | 40.379 | 317 | 177 | 5 | 3 | 316 | 4 | 311 | 2.94E-77 | 249 |
| **S2_GD2017_2_manure_scaffold_3_408|GD2017-2_manure_QB3_180125_Potentially_complete_Phage_48_40|GD2017-2_manure_QB3_180125** | SR-VP_0-2_scaffold_141_5191495_64|SR-VP_0-2cm_Phage_38_25|SR-VP_0-2cm | 40.379 | 317 | 177 | 5 | 3 | 316 | 4 | 311 | 2.94E-77 | 249 |
| **S2_GD2017_2_manure_scaffold_3_408|GD2017-2_manure_QB3_180125_Potentially_complete_Phage_48_40|GD2017-2_manure_QB3_180125** | SR-VP_0-2_scaffold_141_3175919_4|SR-VP_0-2cm_UNK|SR-VP_0-2cm | 41.066 | 319 | 177 | 5 | 3 | 316 | 4 | 316 | 5.3E-77 | 248 |
| **S2_GD2017_2_manure_scaffold_3_408|GD2017-2_manure_QB3_180125_Potentially_complete_Phage_48_40|GD2017-2_manure_QB3_180125** | FFC_04162018_0_1um_scaffold_562_5|FFC_04162018_0_1um_UNK|FFC_04162018_0_1um | 40.125 | 319 | 177 | 6 | 3 | 316 | 5 | 314 | 6.54E-77 | 248 |
| **S2_GD2017_2_manure_scaffold_3_408|GD2017-2_manure_QB3_180125_Potentially_complete_Phage_48_40|GD2017-2_manure_QB3_180125** | LacPavin_0419_WC70S_scaffold_436490_8|LacPavin_0419_WC70S_UNK|LacPavin_0419_WC70S | 40.635 | 315 | 175 | 6 | 3 | 316 | 4 | 307 | 6.97E-77 | 248 |
| **S2_GD2017_2_manure_scaffold_3_408|GD2017-2_manure_QB3_180125_Potentially_complete_Phage_48_40|GD2017-2_manure_QB3_180125** | GS605_0p1_scaffold_766_26|lsdeep_GS605_0p1_UNK|lsdeep_GS605_0p1 | 40.895 | 313 | 176 | 4 | 1 | 313 | 1 | 304 | 1.1E-76 | 247 |
| **S2_GD2017_2_manure_scaffold_3_408|GD2017-2_manure_QB3_180125_Potentially_complete_Phage_48_40|GD2017-2_manure_QB3_180125** | BML_07122017_9_5m_scaffold_15179_2|BML_07122017_9_5m_UNK|BML_07122017_9_5m | 41.159 | 328 | 166 | 6 | 3 | 316 | 4 | 318 | 2.69E-76 | 246 |
| **S2_GD2017_2_manure_scaffold_3_408|GD2017-2_manure_QB3_180125_Potentially_complete_Phage_48_40|GD2017-2_manure_QB3_180125** | BML_coassembly_scaffold_632_31|BML_coassembly_UNK|BML_coassembly | 41.159 | 328 | 166 | 6 | 3 | 316 | 4 | 318 | 2.69E-76 | 246 |
| **S2_GD2017_2_manure_scaffold_3_408|GD2017-2_manure_QB3_180125_Potentially_complete_Phage_48_40|GD2017-2_manure_QB3_180125** | FFC_082018_0_1um_scaffold_1367_4|FFC_082018_0_1um_UNK|FFC_082018_0_1um | 40.453 | 309 | 172 | 6 | 9 | 316 | 11 | 308 | 3.32E-76 | 246 |
| **S2_GD2017_2_manure_scaffold_3_408|GD2017-2_manure_QB3_180125_Potentially_complete_Phage_48_40|GD2017-2_manure_QB3_180125** | GS843_0p1_scaffold_644_7|lsdeep_GS843_0p1_UNK|lsdeep_GS843_0p1 | 40.895 | 313 | 176 | 4 | 1 | 313 | 1 | 304 | 1.13E-75 | 244 |
| **S2_GD2017_2_manure_scaffold_3_408|GD2017-2_manure_QB3_180125_Potentially_complete_Phage_48_40|GD2017-2_manure_QB3_180125** | H1a2_full_idba_ud_scaffold_13397_6|H1a2_UNK|H1a2 | 40.379 | 317 | 183 | 4 | 3 | 316 | 4 | 317 | 4.18E-75 | 243 |
| **S2_GD2017_2_manure_scaffold_3_408|GD2017-2_manure_QB3_180125_Potentially_complete_Phage_48_40|GD2017-2_manure_QB3_180125** | LacPavin_0419_WC70S_scaffold_53030_2|LacPavin_0419_WC70S_UNK|LacPavin_0419_WC70S | 39.441 | 322 | 172 | 7 | 3 | 313 | 4 | 313 | 7.8E-75 | 243 |
| **S2_GD2017_2_manure_scaffold_3_408|GD2017-2_manure_QB3_180125_Potentially_complete_Phage_48_40|GD2017-2_manure_QB3_180125** | AB_072018_0_1um_scaffold_191_18|AB_072018_0_1um_UNK|AB_072018_0_1um | 38.941 | 321 | 178 | 6 | 1 | 315 | 1 | 309 | 2.5E-74 | 241 |
| **S2_GD2017_2_manure_scaffold_3_408|GD2017-2_manure_QB3_180125_Potentially_complete_Phage_48_40|GD2017-2_manure_QB3_180125** | SR-VP_0-2_scaffold_141_6448357_11|SR-VP_0-2cm_UNK|SR-VP_0-2cm | 38.535 | 314 | 181 | 6 | 3 | 313 | 4 | 308 | 9.09E-74 | 240 |
| **S2_GD2017_2_manure_scaffold_3_408|GD2017-2_manure_QB3_180125_Potentially_complete_Phage_48_40|GD2017-2_manure_QB3_180125** | FFC_092018_0_1um_scaffold_9152_2|FFC_092018_0_1um_UNK|FFC_092018_0_1um | 38.390 | 323 | 180 | 6 | 1 | 316 | 3 | 313 | 2.04E-73 | 239 |
| **S2_GD2017_2_manure_scaffold_3_408|GD2017-2_manure_QB3_180125_Potentially_complete_Phage_48_40|GD2017-2_manure_QB3_180125** | LacPavin_0419_WC70S_scaffold_945204_3|LacPavin_0419_WC70S_UNK|LacPavin_0419_WC70S | 39.450 | 327 | 179 | 6 | 1 | 316 | 3 | 321 | 4.15E-73 | 238 |
| **S2_GD2017_2_manure_scaffold_3_408|GD2017-2_manure_QB3_180125_Potentially_complete_Phage_48_40|GD2017-2_manure_QB3_180125** | LacPavin_0818_WC55_scaffold_113784_prodigal-single_367|LP_PHAGE_COMPLETE_34_34|LacPavin_0818_WC55 | 39.450 | 327 | 179 | 6 | 1 | 316 | 3 | 321 | 4.15E-73 | 238 |
| **S2_GD2017_2_manure_scaffold_3_408|GD2017-2_manure_QB3_180125_Potentially_complete_Phage_48_40|GD2017-2_manure_QB3_180125** | LacPavin_0818_WC55_scaffold_113784_356|LacPavin_0818_WC55_Potentially_Complete_Phage_34_34|LacPavin_0818_WC55 | 39.450 | 327 | 179 | 6 | 1 | 316 | 3 | 321 | 4.15E-73 | 238 |
| **S2_GD2017_2_manure_scaffold_3_408|GD2017-2_manure_QB3_180125_Potentially_complete_Phage_48_40|GD2017-2_manure_QB3_180125** | LacPavin_0818_WC45_scaffold_80267_116|LacPavin_0818_WC45_UNK|LacPavin_0818_WC45 | 39.450 | 327 | 179 | 6 | 1 | 316 | 3 | 321 | 4.15E-73 | 238 |
| **S2_GD2017_2_manure_scaffold_3_408|GD2017-2_manure_QB3_180125_Potentially_complete_Phage_48_40|GD2017-2_manure_QB3_180125** | LacPavin_0718_WC55_scaffold_0_34|LacPavin_0718_WC55_Phage_34_15|LacPavin_0718_WC55 | 39.450 | 327 | 179 | 6 | 1 | 316 | 3 | 321 | 4.15E-73 | 238 |
| **S2_GD2017_2_manure_scaffold_3_408|GD2017-2_manure_QB3_180125_Potentially_complete_Phage_48_40|GD2017-2_manure_QB3_180125** | LacPavin_0718_WC45_scaffold_3_116|LacPavin_0718_WC45_UNK|LacPavin_0718_WC45 | 39.450 | 327 | 179 | 6 | 1 | 316 | 3 | 321 | 4.15E-73 | 238 |
| **S2_GD2017_2_manure_scaffold_3_408|GD2017-2_manure_QB3_180125_Potentially_complete_Phage_48_40|GD2017-2_manure_QB3_180125** | ALT_03122018_0_1um_scaffold_7667_2|ALT_03122018_0_1um_UNK|ALT_03122018_0_1um | 38.629 | 321 | 179 | 6 | 1 | 315 | 1 | 309 | 4.47E-73 | 238 |
| **S2_GD2017_2_manure_scaffold_3_408|GD2017-2_manure_QB3_180125_Potentially_complete_Phage_48_40|GD2017-2_manure_QB3_180125** | BML_08182015_8_5m_scaffold_1747_4|BML_08182015_8_5m_UNK|BML_08182015_8_5m | 39.308 | 318 | 181 | 7 | 1 | 315 | 1 | 309 | 5.73E-73 | 238 |
| **S2_GD2017_2_manure_scaffold_3_408|GD2017-2_manure_QB3_180125_Potentially_complete_Phage_48_40|GD2017-2_manure_QB3_180125** | BML_08182015_1_5m_scaffold_1135_22|BML_08182015_1_5m_UNK|BML_08182015_1_5m | 39.308 | 318 | 181 | 7 | 1 | 315 | 1 | 309 | 5.73E-73 | 238 |
| **S2_GD2017_2_manure_scaffold_3_408|GD2017-2_manure_QB3_180125_Potentially_complete_Phage_48_40|GD2017-2_manure_QB3_180125** | BML_coassembly_scaffold_23117_6|BML_coassembly_UNK|BML_coassembly | 39.308 | 318 | 181 | 7 | 1 | 315 | 1 | 309 | 5.73E-73 | 238 |
| **S2_GD2017_2_manure_scaffold_3_408|GD2017-2_manure_QB3_180125_Potentially_complete_Phage_48_40|GD2017-2_manure_QB3_180125** | PH2017_20_SFW_D_A_scaffold_10948_1|PH2017_20_SFW_D_A_full_UNK|PH2017_20_SFW_D_A_full | 38.941 | 321 | 178 | 6 | 1 | 315 | 1 | 309 | 2.25E-72 | 236 |
| **S2_GD2017_2_manure_scaffold_3_408|GD2017-2_manure_QB3_180125_Potentially_complete_Phage_48_40|GD2017-2_manure_QB3_180125** | PH2017_20_SFW_D_A_scaffold_9668_1|PH2017_20_SFW_D_A_filtered_BT2def_UNK|PH2017_20_SFW_D_A_filtered_BT2def | 38.941 | 321 | 178 | 6 | 1 | 315 | 1 | 309 | 2.25E-72 | 236 |
| **S2_GD2017_2_manure_scaffold_3_408|GD2017-2_manure_QB3_180125_Potentially_complete_Phage_48_40|GD2017-2_manure_QB3_180125** | PH2017_20_SFW_D_A_scaffold_10190_1|PH2017_20_SFW_D_A_filtered_UNK|PH2017_20_SFW_D_A_filtered | 38.941 | 321 | 178 | 6 | 1 | 315 | 1 | 309 | 2.25E-72 | 236 |
| **S2_GD2017_2_manure_scaffold_3_408|GD2017-2_manure_QB3_180125_Potentially_complete_Phage_48_40|GD2017-2_manure_QB3_180125** | LacPavin_0419_WC70S_scaffold_29687_52|LacPavin_0419_WC70S_UNK|LacPavin_0419_WC70S | 39.365 | 315 | 178 | 5 | 1 | 315 | 1 | 302 | 3.49E-72 | 236 |
| **S2_GD2017_2_manure_scaffold_3_408|GD2017-2_manure_QB3_180125_Potentially_complete_Phage_48_40|GD2017-2_manure_QB3_180125** | amazon_river_scaffold_24401_1|amazon_river_UNK|amazon_river | 41.901 | 284 | 154 | 6 | 9 | 291 | 11 | 284 | 4.21E-72 | 234 |
| **S2_GD2017_2_manure_scaffold_3_408|GD2017-2_manure_QB3_180125_Potentially_complete_Phage_48_40|GD2017-2_manure_QB3_180125** | AB_092018_0_1um_scaffold_3669_6|AB_092018_0_1um_UNK|AB_092018_0_1um | 38.700 | 323 | 183 | 5 | 1 | 316 | 3 | 317 | 1.19E-71 | 234 |
| **S2_GD2017_2_manure_scaffold_3_408|GD2017-2_manure_QB3_180125_Potentially_complete_Phage_48_40|GD2017-2_manure_QB3_180125** | AB_092018_0_1um_scaffold_69710_2|AB_092018_0_1um_UNK|AB_092018_0_1um | 38.170 | 317 | 185 | 5 | 1 | 315 | 1 | 308 | 1.51E-71 | 234 |
| **S2_GD2017_2_manure_scaffold_3_408|GD2017-2_manure_QB3_180125_Potentially_complete_Phage_48_40|GD2017-2_manure_QB3_180125** | LacPavin_0419_WC53_scaffold_459219_53|LacPavin_0419_WC53_UNK|LacPavin_0419_WC53 | 39.048 | 315 | 179 | 5 | 1 | 315 | 1 | 302 | 1.64E-71 | 234 |
| **S2_GD2017_2_manure_scaffold_3_408|GD2017-2_manure_QB3_180125_Potentially_complete_Phage_48_40|GD2017-2_manure_QB3_180125** | LacPavin_0818_WC40_scaffold_701884_7|LacPavin_0818_WC40_UNK|LacPavin_0818_WC40 | 39.048 | 315 | 179 | 5 | 1 | 315 | 1 | 302 | 1.64E-71 | 234 |
| **S2_GD2017_2_manure_scaffold_3_408|GD2017-2_manure_QB3_180125_Potentially_complete_Phage_48_40|GD2017-2_manure_QB3_180125** | FFC_04162018_0_1um_scaffold_1491_3|FFC_04162018_0_1um_UNK|FFC_04162018_0_1um | 38.700 | 323 | 183 | 5 | 1 | 316 | 3 | 317 | 1.66E-71 | 234 |
| **S2_GD2017_2_manure_scaffold_3_408|GD2017-2_manure_QB3_180125_Potentially_complete_Phage_48_40|GD2017-2_manure_QB3_180125** | AB_082018_0_1um_scaffold_2553_2|AB_082018_0_1um_UNK|AB_082018_0_1um | 38.700 | 323 | 183 | 5 | 1 | 316 | 3 | 317 | 1.87E-71 | 234 |
| **S2_GD2017_2_manure_scaffold_3_408|GD2017-2_manure_QB3_180125_Potentially_complete_Phage_48_40|GD2017-2_manure_QB3_180125** | SP8F_scaffold_1008_4|sea_ice_sp8_UNK|sea_ice_sp8 | 39.365 | 315 | 179 | 7 | 1 | 313 | 1 | 305 | 2.39E-71 | 233 |
| **S2_GD2017_2_manure_scaffold_3_408|GD2017-2_manure_QB3_180125_Potentially_complete_Phage_48_40|GD2017-2_manure_QB3_180125** | AB_072018_0_1um_scaffold_612_14|AB_072018_0_1um_UNK|AB_072018_0_1um | 38.700 | 323 | 183 | 5 | 1 | 316 | 3 | 317 | 5.02E-71 | 233 |
| **S2_GD2017_2_manure_scaffold_3_408|GD2017-2_manure_QB3_180125_Potentially_complete_Phage_48_40|GD2017-2_manure_QB3_180125** | LacPavin_0818_WC40_scaffold_152276_4|LacPavin_0818_WC40_UNK|LacPavin_0818_WC40 | 38.679 | 318 | 186 | 6 | 1 | 316 | 3 | 313 | 5.06E-71 | 233 |
| **S2_GD2017_2_manure_scaffold_3_408|GD2017-2_manure_QB3_180125_Potentially_complete_Phage_48_40|GD2017-2_manure_QB3_180125** | ERMGT157_2_scaffold_21646_2|ERMGT157_2_UNK|ERMGT157_2 | 39.873 | 316 | 177 | 7 | 3 | 316 | 4 | 308 | 5.46E-71 | 233 |
| **S2_GD2017_2_manure_scaffold_3_408|GD2017-2_manure_QB3_180125_Potentially_complete_Phage_48_40|GD2017-2_manure_QB3_180125** | BC_09192017_0_5m_scaffold_204_34|BC_09192017_0_5m_UNK|BC_09192017_0_5m | 40.645 | 310 | 173 | 4 | 6 | 315 | 3 | 301 | 7.45E-71 | 232 |
| **S2_GD2017_2_manure_scaffold_3_408|GD2017-2_manure_QB3_180125_Potentially_complete_Phage_48_40|GD2017-2_manure_QB3_180125** | AB_092018_0_1um_scaffold_21218_4|AB_092018_0_1um_UNK|AB_092018_0_1um | 39.482 | 309 | 170 | 7 | 1 | 304 | 1 | 297 | 7.68E-71 | 232 |
| **S2_GD2017_2_manure_scaffold_3_408|GD2017-2_manure_QB3_180125_Potentially_complete_Phage_48_40|GD2017-2_manure_QB3_180125** | BC_09192017_0_5m_scaffold_71308_1|BC_09192017_0_5m_UNK|BC_09192017_0_5m | 38.438 | 320 | 180 | 7 | 1 | 315 | 1 | 308 | 9.14E-71 | 232 |
| **S2_GD2017_2_manure_scaffold_3_408|GD2017-2_manure_QB3_180125_Potentially_complete_Phage_48_40|GD2017-2_manure_QB3_180125** | SR-VP_0-2_scaffold_141_7274537_3|SR-VP_0-2cm_UNK|SR-VP_0-2cm | 39.432 | 317 | 180 | 6 | 3 | 316 | 4 | 311 | 9.22E-71 | 232 |
| **S2_GD2017_2_manure_scaffold_3_408|GD2017-2_manure_QB3_180125_Potentially_complete_Phage_48_40|GD2017-2_manure_QB3_180125** | LacPavin_0419_WC53_scaffold_69987_2|LacPavin_0419_WC53_UNK|LacPavin_0419_WC53 | 38.462 | 325 | 181 | 6 | 1 | 316 | 3 | 317 | 1.46E-70 | 232 |
| **S2_GD2017_2_manure_scaffold_3_408|GD2017-2_manure_QB3_180125_Potentially_complete_Phage_48_40|GD2017-2_manure_QB3_180125** | FFC_082018_0_1um_scaffold_11518_2|FFC_082018_0_1um_UNK|FFC_082018_0_1um | 38.438 | 320 | 180 | 7 | 1 | 315 | 1 | 308 | 1.73E-70 | 231 |
| **S2_GD2017_2_manure_scaffold_3_408|GD2017-2_manure_QB3_180125_Potentially_complete_Phage_48_40|GD2017-2_manure_QB3_180125** | AB_092018_0_1um_scaffold_6932_6|AB_092018_0_1um_UNK|AB_092018_0_1um | 39.739 | 307 | 173 | 7 | 1 | 304 | 13 | 310 | 2.7E-70 | 231 |
| **S2_GD2017_2_manure_scaffold_3_408|GD2017-2_manure_QB3_180125_Potentially_complete_Phage_48_40|GD2017-2_manure_QB3_180125** | LacPavin_0818_WC40_scaffold_160089_2|LacPavin_0818_WC40_UNK|LacPavin_0818_WC40 | 39.185 | 319 | 182 | 8 | 1 | 313 | 1 | 313 | 3.13E-70 | 231 |
| **S2_GD2017_2_manure_scaffold_3_408|GD2017-2_manure_QB3_180125_Potentially_complete_Phage_48_40|GD2017-2_manure_QB3_180125** | LacPavin_0419_WC70S_scaffold_308887_22|LacPavin_0419_WC70S_UNK|LacPavin_0419_WC70S | 39.241 | 316 | 180 | 7 | 1 | 313 | 1 | 307 | 4.02E-70 | 231 |
| **S2_GD2017_2_manure_scaffold_3_408|GD2017-2_manure_QB3_180125_Potentially_complete_Phage_48_40|GD2017-2_manure_QB3_180125** | LacPavin_0818_WC40_scaffold_164700_2|LacPavin_0818_WC40_UNK|LacPavin_0818_WC40 | 38.080 | 323 | 185 | 5 | 1 | 316 | 3 | 317 | 4.19E-70 | 231 |
| **S2_GD2017_2_manure_scaffold_3_408|GD2017-2_manure_QB3_180125_Potentially_complete_Phage_48_40|GD2017-2_manure_QB3_180125** | Salt_Pond_SF2_B_H2O_MG_scaffold_415_25|JGI_Salt_Pond_SF2_B_H2O_MG_UNK|JGI_Salt_Pond_SF2_B_H2O_MG | 38.924 | 316 | 181 | 7 | 1 | 313 | 1 | 307 | 6.08E-70 | 230 |
| **S2_GD2017_2_manure_scaffold_3_408|GD2017-2_manure_QB3_180125_Potentially_complete_Phage_48_40|GD2017-2_manure_QB3_180125** | Salt_Pond_SF2_C_H2O_MG_scaffold_87051_1|JGI_Salt_Pond_SF2_C_H2O_MG_UNK|JGI_Salt_Pond_SF2_C_H2O_MG | 38.065 | 310 | 182 | 6 | 1 | 309 | 1 | 301 | 6.09E-70 | 230 |
| **S2_GD2017_2_manure_scaffold_3_408|GD2017-2_manure_QB3_180125_Potentially_complete_Phage_48_40|GD2017-2_manure_QB3_180125** | LacPavin_0818_WC40_scaffold_329966_1|LacPavin_0818_WC40_UNK|LacPavin_0818_WC40 | 38.966 | 290 | 171 | 3 | 23 | 310 | 15 | 300 | 9.7E-70 | 229 |
| **S2_GD2017_2_manure_scaffold_3_407|GD2017-2_manure_QB3_180125_Potentially_complete_Phage_48_40|GD2017-2_manure_QB3_180125** | GD18-4_manure_scaffold_876276_6|GD2018-4_manure_QB3_180703_UNK|GD2018-4_manure_QB3_180703 | 100.000 | 301 | 0 | 0 | 1 | 301 | 1 | 301 | 0.0 | 619 |
| **S2_GD2017_2_manure_scaffold_3_407|GD2017-2_manure_QB3_180125_Potentially_complete_Phage_48_40|GD2017-2_manure_QB3_180125** | S30_BME30_294872_50|BM_2017_Coates_4_Potentially_Complete_Phage_48-32|BM_2017_Coates_4 | 100.000 | 301 | 0 | 0 | 1 | 301 | 1 | 301 | 0.0 | 619 |
| **S2_GD2017_2_manure_scaffold_3_407|GD2017-2_manure_QB3_180125_Potentially_complete_Phage_48_40|GD2017-2_manure_QB3_180125** | S28_BME28_138683_395|BM_2017_Strous_8_UNK|BM_2017_Strous_8 | 100.000 | 301 | 0 | 0 | 1 | 301 | 1 | 301 | 0.0 | 619 |
| **S2_GD2017_2_manure_scaffold_3_407|GD2017-2_manure_QB3_180125_Potentially_complete_Phage_48_40|GD2017-2_manure_QB3_180125** | S27_BME27_629333_prodigal-single_187|BM_PHAGE_48_13|BM_2017_Strous_6 | 100.000 | 301 | 0 | 0 | 1 | 301 | 1 | 301 | 0.0 | 619 |
| **S2_GD2017_2_manure_scaffold_3_407|GD2017-2_manure_QB3_180125_Potentially_complete_Phage_48_40|GD2017-2_manure_QB3_180125** | S27_BME27_629333_177|BM_2017_Strous_6_Phage_48_13|BM_2017_Strous_6 | 100.000 | 301 | 0 | 0 | 1 | 301 | 1 | 301 | 0.0 | 619 |
| **S2_GD2017_2_manure_scaffold_3_407|GD2017-2_manure_QB3_180125_Potentially_complete_Phage_48_40|GD2017-2_manure_QB3_180125** | S33_3P_scaffold_613888_2|Genasci_Feb2018_S33_3P_UNK|Genasci_Feb2018_S33_3P | 100.000 | 301 | 0 | 0 | 1 | 301 | 1 | 301 | 0.0 | 619 |
| **S2_GD2017_2_manure_scaffold_3_407|GD2017-2_manure_QB3_180125_Potentially_complete_Phage_48_40|GD2017-2_manure_QB3_180125** | S22_GE22_scaffold_285059_prodigal-single_512|GD_PHAGE_COMPLETE_48_49|E_GD2017-2_anammox-7_S22_GE22_Biohub_180515 | 100.000 | 301 | 0 | 0 | 1 | 301 | 1 | 301 | 0.0 | 619 |
| **S2_GD2017_2_manure_scaffold_3_407|GD2017-2_manure_QB3_180125_Potentially_complete_Phage_48_40|GD2017-2_manure_QB3_180125** | S22_GE22_scaffold_285059_435|E_GD2017-2_anammox-7_S22_GE22_Biohub_170515_Potentially_Complete_48_49|E_GD2017-2_anammox-7_S22_GE22_Biohub_180515 | 100.000 | 301 | 0 | 0 | 1 | 301 | 1 | 301 | 0.0 | 619 |
| **S2_GD2017_2_manure_scaffold_3_407|GD2017-2_manure_QB3_180125_Potentially_complete_Phage_48_40|GD2017-2_manure_QB3_180125** | S20_GE20_scaffold_396645_11|E_GD2017-2_strous-5_S20_GE20_Biohub_170515_UNK|E_GD2017-2_strous-5_S20_GE20_Biohub_180515 | 100.000 | 301 | 0 | 0 | 1 | 301 | 1 | 301 | 0.0 | 619 |
| **S2_GD2017_2_manure_scaffold_3_407|GD2017-2_manure_QB3_180125_Potentially_complete_Phage_48_40|GD2017-2_manure_QB3_180125** | S15_GE15_scaffold_320047_245|E_GD2017-2_urea-2_S15_GE15_Biohub_170515_UNK|E_GD2017-2_urea-2_S15_GE15_Biohub_180515 | 100.000 | 301 | 0 | 0 | 1 | 301 | 1 | 301 | 0.0 | 619 |
| **S2_GD2017_2_manure_scaffold_3_407|GD2017-2_manure_QB3_180125_Potentially_complete_Phage_48_40|GD2017-2_manure_QB3_180125** | S14_GE14_scaffold_311130_15|E_GD2017-2_urea-2_S14_GE14_Biohub_170515_UNK|E_GD2017-2_urea-2_S14_GE14_Biohub_180515 | 100.000 | 301 | 0 | 0 | 1 | 301 | 1 | 301 | 0.0 | 619 |
| **S2_GD2017_2_manure_scaffold_3_407|GD2017-2_manure_QB3_180125_Potentially_complete_Phage_48_40|GD2017-2_manure_QB3_180125** | P0_An_pond3_S8_coassembly_k141_2723617_436|P0_An_pond3_S8_coassembly_UNK|E_GD2017-1_P0_An_pond3_S8_Biohub_coassembly | 100.000 | 301 | 0 | 0 | 1 | 301 | 1 | 301 | 0.0 | 619 |
| **S2_GD2017_2_manure_scaffold_3_407|GD2017-2_manure_QB3_180125_Potentially_complete_Phage_48_40|GD2017-2_manure_QB3_180125** | P0_An_GD2017L_S7_coassembly_k141_1013362_prodigal-single_82|GD_PHAGE_48_49|E_P0_An_GD2017L_S7_coassembly | 100.000 | 301 | 0 | 0 | 1 | 301 | 1 | 301 | 0.0 | 619 |
| **S2_GD2017_2_manure_scaffold_3_407|GD2017-2_manure_QB3_180125_Potentially_complete_Phage_48_40|GD2017-2_manure_QB3_180125** | P0_An_GD2017L_S7_coassembly_k141_1013362_76|P0_An_GD2017L_S7_coassembly_Phage_48_49|E_P0_An_GD2017L_S7_coassembly | 100.000 | 301 | 0 | 0 | 1 | 301 | 1 | 301 | 0.0 | 619 |
| **S2_GD2017_2_manure_scaffold_3_407|GD2017-2_manure_QB3_180125_Potentially_complete_Phage_48_40|GD2017-2_manure_QB3_180125** | S19_GE19_scaffold_2259_prodigal-single_283|GD_PHAGE_48_10|E_GD2017-2_strous-5-pellet_S19_GE19_Biohub_180515 | 100.000 | 301 | 0 | 0 | 1 | 301 | 1 | 301 | 0.0 | 619 |
| **S2_GD2017_2_manure_scaffold_3_407|GD2017-2_manure_QB3_180125_Potentially_complete_Phage_48_40|GD2017-2_manure_QB3_180125** | S19_GE19_scaffold_2259_259|E_GD2017-2_strous-5-pellet_S19_GE19_Biohub_170515_Phage_48_10|E_GD2017-2_strous-5-pellet_S19_GE19_Biohub_180515 | 100.000 | 301 | 0 | 0 | 1 | 301 | 1 | 301 | 0.0 | 619 |
| **S2_GD2017_2_manure_scaffold_3_407|GD2017-2_manure_QB3_180125_Potentially_complete_Phage_48_40|GD2017-2_manure_QB3_180125** | S18_GE18_scaffold_616449_290|E_GD2017-2_strous-5-prefilter_S18_GE18_Biohub_170515_UNK|E_GD2017-2_strous-5-prefilter_S18_GE18_Biohub_180515 | 100.000 | 301 | 0 | 0 | 1 | 301 | 1 | 301 | 0.0 | 619 |
| **S2_GD2017_2_manure_scaffold_3_407|GD2017-2_manure_QB3_180125_Potentially_complete_Phage_48_40|GD2017-2_manure_QB3_180125** | S16_GE16_scaffold_390213_388|E_GD2017-2_urea-3_S16_GE16_Biohub_170515_UNK|E_GD2017-2_urea-3_S16_GE16_Biohub_180515 | 100.000 | 301 | 0 | 0 | 1 | 301 | 1 | 301 | 0.0 | 619 |
| **S2_GD2017_2_manure_scaffold_3_407|GD2017-2_manure_QB3_180125_Potentially_complete_Phage_48_40|GD2017-2_manure_QB3_180125** | P0_An_pond3_S8_170907_scaffold_1005865_39|E_GD2017-1_P0_AN_POND3_S8_BIOHUB_170907_UNK|E_GD2017-1_P0_An_pond3_S8_Biohub_170907 | 100.000 | 301 | 0 | 0 | 1 | 301 | 1 | 301 | 0.0 | 619 |
| **S2_GD2017_2_manure_scaffold_3_407|GD2017-2_manure_QB3_180125_Potentially_complete_Phage_48_40|GD2017-2_manure_QB3_180125** | P0_An_GD2017L_S7_170907_scaffold_359857_22|E_GD2017-1_P0_An_GD2017L_S7_Biohub_170907_UNK|E_GD2017-1_P0_An_GD2017L_S7_Biohub_170907 | 100.000 | 301 | 0 | 0 | 1 | 301 | 1 | 301 | 0.0 | 619 |
| **S2_GD2017_2_manure_scaffold_3_407|GD2017-2_manure_QB3_180125_Potentially_complete_Phage_48_40|GD2017-2_manure_QB3_180125** | S2_GD2017_2_manure_scaffold_3_prodigal-single_480|GD_PHAGE_COMPLETE_48_40|GD2017-2_manure_QB3_180125 | 100.000 | 301 | 0 | 0 | 1 | 301 | 1 | 301 | 0.0 | 619 |
| **S2_GD2017_2_manure_scaffold_3_407|GD2017-2_manure_QB3_180125_Potentially_complete_Phage_48_40|GD2017-2_manure_QB3_180125** | S2_GD2017_2_manure_scaffold_3_407|GD2017-2_manure_QB3_180125_Potentially_complete_Phage_48_40|GD2017-2_manure_QB3_180125 | 100.000 | 301 | 0 | 0 | 1 | 301 | 1 | 301 | 0.0 | 619 |
| **S2_GD2017_2_manure_scaffold_3_406|GD2017-2_manure_QB3_180125_Potentially_complete_Phage_48_40|GD2017-2_manure_QB3_180125** | GD18-4_manure_scaffold_876276_5|GD2018-4_manure_QB3_180703_UNK|GD2018-4_manure_QB3_180703 | 100.000 | 306 | 0 | 0 | 1 | 306 | 1 | 306 | 0.0 | 641 |
| **S2_GD2017_2_manure_scaffold_3_406|GD2017-2_manure_QB3_180125_Potentially_complete_Phage_48_40|GD2017-2_manure_QB3_180125** | S30_BME30_294872_51|BM_2017_Coates_4_Potentially_Complete_Phage_48-32|BM_2017_Coates_4 | 100.000 | 306 | 0 | 0 | 1 | 306 | 1 | 306 | 0.0 | 641 |
| **S2_GD2017_2_manure_scaffold_3_406|GD2017-2_manure_QB3_180125_Potentially_complete_Phage_48_40|GD2017-2_manure_QB3_180125** | S28_BME28_138683_394|BM_2017_Strous_8_UNK|BM_2017_Strous_8 | 100.000 | 306 | 0 | 0 | 1 | 306 | 1 | 306 | 0.0 | 641 |
| **S2_GD2017_2_manure_scaffold_3_406|GD2017-2_manure_QB3_180125_Potentially_complete_Phage_48_40|GD2017-2_manure_QB3_180125** | S27_BME27_629333_prodigal-single_188|BM_PHAGE_48_13|BM_2017_Strous_6 | 100.000 | 306 | 0 | 0 | 1 | 306 | 1 | 306 | 0.0 | 641 |
| **S2_GD2017_2_manure_scaffold_3_406|GD2017-2_manure_QB3_180125_Potentially_complete_Phage_48_40|GD2017-2_manure_QB3_180125** | S27_BME27_629333_178|BM_2017_Strous_6_Phage_48_13|BM_2017_Strous_6 | 100.000 | 306 | 0 | 0 | 1 | 306 | 1 | 306 | 0.0 | 641 |
| **S2_GD2017_2_manure_scaffold_3_406|GD2017-2_manure_QB3_180125_Potentially_complete_Phage_48_40|GD2017-2_manure_QB3_180125** | S33_3P_scaffold_613888_3|Genasci_Feb2018_S33_3P_UNK|Genasci_Feb2018_S33_3P | 100.000 | 306 | 0 | 0 | 1 | 306 | 1 | 306 | 0.0 | 641 |
| **S2_GD2017_2_manure_scaffold_3_406|GD2017-2_manure_QB3_180125_Potentially_complete_Phage_48_40|GD2017-2_manure_QB3_180125** | S22_GE22_scaffold_285059_prodigal-single_511|GD_PHAGE_COMPLETE_48_49|E_GD2017-2_anammox-7_S22_GE22_Biohub_180515 | 100.000 | 306 | 0 | 0 | 1 | 306 | 1 | 306 | 0.0 | 641 |
| **S2_GD2017_2_manure_scaffold_3_406|GD2017-2_manure_QB3_180125_Potentially_complete_Phage_48_40|GD2017-2_manure_QB3_180125** | S22_GE22_scaffold_285059_434|E_GD2017-2_anammox-7_S22_GE22_Biohub_170515_Potentially_Complete_48_49|E_GD2017-2_anammox-7_S22_GE22_Biohub_180515 | 100.000 | 306 | 0 | 0 | 1 | 306 | 1 | 306 | 0.0 | 641 |
| **S2_GD2017_2_manure_scaffold_3_406|GD2017-2_manure_QB3_180125_Potentially_complete_Phage_48_40|GD2017-2_manure_QB3_180125** | S20_GE20_scaffold_396645_10|E_GD2017-2_strous-5_S20_GE20_Biohub_170515_UNK|E_GD2017-2_strous-5_S20_GE20_Biohub_180515 | 100.000 | 306 | 0 | 0 | 1 | 306 | 1 | 306 | 0.0 | 641 |
| **S2_GD2017_2_manure_scaffold_3_406|GD2017-2_manure_QB3_180125_Potentially_complete_Phage_48_40|GD2017-2_manure_QB3_180125** | S15_GE15_scaffold_320047_246|E_GD2017-2_urea-2_S15_GE15_Biohub_170515_UNK|E_GD2017-2_urea-2_S15_GE15_Biohub_180515 | 100.000 | 306 | 0 | 0 | 1 | 306 | 1 | 306 | 0.0 | 641 |
| **S2_GD2017_2_manure_scaffold_3_406|GD2017-2_manure_QB3_180125_Potentially_complete_Phage_48_40|GD2017-2_manure_QB3_180125** | S14_GE14_scaffold_311130_16|E_GD2017-2_urea-2_S14_GE14_Biohub_170515_UNK|E_GD2017-2_urea-2_S14_GE14_Biohub_180515 | 100.000 | 306 | 0 | 0 | 1 | 306 | 1 | 306 | 0.0 | 641 |
| **S2_GD2017_2_manure_scaffold_3_406|GD2017-2_manure_QB3_180125_Potentially_complete_Phage_48_40|GD2017-2_manure_QB3_180125** | P0_An_pond3_S8_coassembly_k141_2723617_437|P0_An_pond3_S8_coassembly_UNK|E_GD2017-1_P0_An_pond3_S8_Biohub_coassembly | 100.000 | 306 | 0 | 0 | 1 | 306 | 1 | 306 | 0.0 | 641 |
| **S2_GD2017_2_manure_scaffold_3_406|GD2017-2_manure_QB3_180125_Potentially_complete_Phage_48_40|GD2017-2_manure_QB3_180125** | P0_An_GD2017L_S7_coassembly_k141_1013362_prodigal-single_83|GD_PHAGE_48_49|E_P0_An_GD2017L_S7_coassembly | 100.000 | 306 | 0 | 0 | 1 | 306 | 1 | 306 | 0.0 | 641 |
| **S2_GD2017_2_manure_scaffold_3_406|GD2017-2_manure_QB3_180125_Potentially_complete_Phage_48_40|GD2017-2_manure_QB3_180125** | P0_An_GD2017L_S7_coassembly_k141_1013362_77|P0_An_GD2017L_S7_coassembly_Phage_48_49|E_P0_An_GD2017L_S7_coassembly | 100.000 | 306 | 0 | 0 | 1 | 306 | 1 | 306 | 0.0 | 641 |
| **S2_GD2017_2_manure_scaffold_3_406|GD2017-2_manure_QB3_180125_Potentially_complete_Phage_48_40|GD2017-2_manure_QB3_180125** | S19_GE19_scaffold_2259_prodigal-single_284|GD_PHAGE_48_10|E_GD2017-2_strous-5-pellet_S19_GE19_Biohub_180515 | 100.000 | 306 | 0 | 0 | 1 | 306 | 1 | 306 | 0.0 | 641 |
| **S2_GD2017_2_manure_scaffold_3_406|GD2017-2_manure_QB3_180125_Potentially_complete_Phage_48_40|GD2017-2_manure_QB3_180125** | S19_GE19_scaffold_2259_260|E_GD2017-2_strous-5-pellet_S19_GE19_Biohub_170515_Phage_48_10|E_GD2017-2_strous-5-pellet_S19_GE19_Biohub_180515 | 100.000 | 306 | 0 | 0 | 1 | 306 | 1 | 306 | 0.0 | 641 |
| **S2_GD2017_2_manure_scaffold_3_406|GD2017-2_manure_QB3_180125_Potentially_complete_Phage_48_40|GD2017-2_manure_QB3_180125** | S18_GE18_scaffold_616449_291|E_GD2017-2_strous-5-prefilter_S18_GE18_Biohub_170515_UNK|E_GD2017-2_strous-5-prefilter_S18_GE18_Biohub_180515 | 100.000 | 306 | 0 | 0 | 1 | 306 | 1 | 306 | 0.0 | 641 |
| **S2_GD2017_2_manure_scaffold_3_406|GD2017-2_manure_QB3_180125_Potentially_complete_Phage_48_40|GD2017-2_manure_QB3_180125** | S16_GE16_scaffold_390213_387|E_GD2017-2_urea-3_S16_GE16_Biohub_170515_UNK|E_GD2017-2_urea-3_S16_GE16_Biohub_180515 | 100.000 | 306 | 0 | 0 | 1 | 306 | 1 | 306 | 0.0 | 641 |
| **S2_GD2017_2_manure_scaffold_3_406|GD2017-2_manure_QB3_180125_Potentially_complete_Phage_48_40|GD2017-2_manure_QB3_180125** | P0_An_pond3_S8_170907_scaffold_1005865_38|E_GD2017-1_P0_AN_POND3_S8_BIOHUB_170907_UNK|E_GD2017-1_P0_An_pond3_S8_Biohub_170907 | 100.000 | 306 | 0 | 0 | 1 | 306 | 1 | 306 | 0.0 | 641 |
| **S2_GD2017_2_manure_scaffold_3_406|GD2017-2_manure_QB3_180125_Potentially_complete_Phage_48_40|GD2017-2_manure_QB3_180125** | P0_An_GD2017L_S7_170907_scaffold_359857_21|E_GD2017-1_P0_An_GD2017L_S7_Biohub_170907_UNK|E_GD2017-1_P0_An_GD2017L_S7_Biohub_170907 | 100.000 | 306 | 0 | 0 | 1 | 306 | 1 | 306 | 0.0 | 641 |
| **S2_GD2017_2_manure_scaffold_3_406|GD2017-2_manure_QB3_180125_Potentially_complete_Phage_48_40|GD2017-2_manure_QB3_180125** | S2_GD2017_2_manure_scaffold_3_prodigal-single_479|GD_PHAGE_COMPLETE_48_40|GD2017-2_manure_QB3_180125 | 100.000 | 306 | 0 | 0 | 1 | 306 | 1 | 306 | 0.0 | 641 |
| **S2_GD2017_2_manure_scaffold_3_406|GD2017-2_manure_QB3_180125_Potentially_complete_Phage_48_40|GD2017-2_manure_QB3_180125** | S2_GD2017_2_manure_scaffold_3_406|GD2017-2_manure_QB3_180125_Potentially_complete_Phage_48_40|GD2017-2_manure_QB3_180125 | 100.000 | 306 | 0 | 0 | 1 | 306 | 1 | 306 | 0.0 | 641 |
| **S2_GD2017_2_manure_scaffold_3_406|GD2017-2_manure_QB3_180125_Potentially_complete_Phage_48_40|GD2017-2_manure_QB3_180125** | L2m2_full_scaffold_20229_1|L2m2_UNK|L2m2 | 55.882 | 306 | 125 | 3 | 1 | 305 | 3 | 299 | 6.94E-118 | 352 |
| **S2_GD2017_2_manure_scaffold_3_410|GD2017-2_manure_QB3_180125_Potentially_complete_Phage_48_40|GD2017-2_manure_QB3_180125** | S30_BME30_294872_47|BM_2017_Coates_4_Potentially_Complete_Phage_48-32|BM_2017_Coates_4 | 100.000 | 155 | 0 | 0 | 1 | 155 | 1 | 155 | 2.5E-108 | 315 |
| **S2_GD2017_2_manure_scaffold_3_410|GD2017-2_manure_QB3_180125_Potentially_complete_Phage_48_40|GD2017-2_manure_QB3_180125** | S28_BME28_138683_398|BM_2017_Strous_8_UNK|BM_2017_Strous_8 | 100.000 | 155 | 0 | 0 | 1 | 155 | 1 | 155 | 2.5E-108 | 315 |
| **S2_GD2017_2_manure_scaffold_3_410|GD2017-2_manure_QB3_180125_Potentially_complete_Phage_48_40|GD2017-2_manure_QB3_180125** | S27_BME27_629333_prodigal-single_184|BM_PHAGE_48_13|BM_2017_Strous_6 | 100.000 | 155 | 0 | 0 | 1 | 155 | 1 | 155 | 2.5E-108 | 315 |
| **S2_GD2017_2_manure_scaffold_3_410|GD2017-2_manure_QB3_180125_Potentially_complete_Phage_48_40|GD2017-2_manure_QB3_180125** | S27_BME27_629333_174|BM_2017_Strous_6_Phage_48_13|BM_2017_Strous_6 | 100.000 | 155 | 0 | 0 | 1 | 155 | 1 | 155 | 2.5E-108 | 315 |
| **S2_GD2017_2_manure_scaffold_3_410|GD2017-2_manure_QB3_180125_Potentially_complete_Phage_48_40|GD2017-2_manure_QB3_180125** | S33_3P_scaffold_263290_5|Genasci_Feb2018_S33_3P_UNK|Genasci_Feb2018_S33_3P | 100.000 | 155 | 0 | 0 | 1 | 155 | 1 | 155 | 2.5E-108 | 315 |
| **S2_GD2017_2_manure_scaffold_3_410|GD2017-2_manure_QB3_180125_Potentially_complete_Phage_48_40|GD2017-2_manure_QB3_180125** | S22_GE22_scaffold_285059_prodigal-single_515|GD_PHAGE_COMPLETE_48_49|E_GD2017-2_anammox-7_S22_GE22_Biohub_180515 | 100.000 | 155 | 0 | 0 | 1 | 155 | 1 | 155 | 2.5E-108 | 315 |
| **S2_GD2017_2_manure_scaffold_3_410|GD2017-2_manure_QB3_180125_Potentially_complete_Phage_48_40|GD2017-2_manure_QB3_180125** | S22_GE22_scaffold_285059_438|E_GD2017-2_anammox-7_S22_GE22_Biohub_170515_Potentially_Complete_48_49|E_GD2017-2_anammox-7_S22_GE22_Biohub_180515 | 100.000 | 155 | 0 | 0 | 1 | 155 | 1 | 155 | 2.5E-108 | 315 |
| **S2_GD2017_2_manure_scaffold_3_410|GD2017-2_manure_QB3_180125_Potentially_complete_Phage_48_40|GD2017-2_manure_QB3_180125** | S20_GE20_scaffold_396645_14|E_GD2017-2_strous-5_S20_GE20_Biohub_170515_UNK|E_GD2017-2_strous-5_S20_GE20_Biohub_180515 | 100.000 | 155 | 0 | 0 | 1 | 155 | 1 | 155 | 2.5E-108 | 315 |
| **S2_GD2017_2_manure_scaffold_3_410|GD2017-2_manure_QB3_180125_Potentially_complete_Phage_48_40|GD2017-2_manure_QB3_180125** | S15_GE15_scaffold_320047_242|E_GD2017-2_urea-2_S15_GE15_Biohub_170515_UNK|E_GD2017-2_urea-2_S15_GE15_Biohub_180515 | 100.000 | 155 | 0 | 0 | 1 | 155 | 1 | 155 | 2.5E-108 | 315 |
| **S2_GD2017_2_manure_scaffold_3_410|GD2017-2_manure_QB3_180125_Potentially_complete_Phage_48_40|GD2017-2_manure_QB3_180125** | S14_GE14_scaffold_311130_12|E_GD2017-2_urea-2_S14_GE14_Biohub_170515_UNK|E_GD2017-2_urea-2_S14_GE14_Biohub_180515 | 100.000 | 155 | 0 | 0 | 1 | 155 | 1 | 155 | 2.5E-108 | 315 |
| **S2_GD2017_2_manure_scaffold_3_410|GD2017-2_manure_QB3_180125_Potentially_complete_Phage_48_40|GD2017-2_manure_QB3_180125** | P0_An_pond3_S8_coassembly_k141_2723617_433|P0_An_pond3_S8_coassembly_UNK|E_GD2017-1_P0_An_pond3_S8_Biohub_coassembly | 100.000 | 155 | 0 | 0 | 1 | 155 | 1 | 155 | 2.5E-108 | 315 |
| **S2_GD2017_2_manure_scaffold_3_410|GD2017-2_manure_QB3_180125_Potentially_complete_Phage_48_40|GD2017-2_manure_QB3_180125** | P0_An_GD2017L_S7_coassembly_k141_1013362_prodigal-single_79|GD_PHAGE_48_49|E_P0_An_GD2017L_S7_coassembly | 100.000 | 155 | 0 | 0 | 1 | 155 | 1 | 155 | 2.5E-108 | 315 |
| **S2_GD2017_2_manure_scaffold_3_410|GD2017-2_manure_QB3_180125_Potentially_complete_Phage_48_40|GD2017-2_manure_QB3_180125** | P0_An_GD2017L_S7_coassembly_k141_1013362_73|P0_An_GD2017L_S7_coassembly_Phage_48_49|E_P0_An_GD2017L_S7_coassembly | 100.000 | 155 | 0 | 0 | 1 | 155 | 1 | 155 | 2.5E-108 | 315 |
| **S2_GD2017_2_manure_scaffold_3_410|GD2017-2_manure_QB3_180125_Potentially_complete_Phage_48_40|GD2017-2_manure_QB3_180125** | S19_GE19_scaffold_2259_prodigal-single_280|GD_PHAGE_48_10|E_GD2017-2_strous-5-pellet_S19_GE19_Biohub_180515 | 100.000 | 155 | 0 | 0 | 1 | 155 | 1 | 155 | 2.5E-108 | 315 |
| **S2_GD2017_2_manure_scaffold_3_410|GD2017-2_manure_QB3_180125_Potentially_complete_Phage_48_40|GD2017-2_manure_QB3_180125** | S19_GE19_scaffold_2259_256|E_GD2017-2_strous-5-pellet_S19_GE19_Biohub_170515_Phage_48_10|E_GD2017-2_strous-5-pellet_S19_GE19_Biohub_180515 | 100.000 | 155 | 0 | 0 | 1 | 155 | 1 | 155 | 2.5E-108 | 315 |
| **S2_GD2017_2_manure_scaffold_3_410|GD2017-2_manure_QB3_180125_Potentially_complete_Phage_48_40|GD2017-2_manure_QB3_180125** | S18_GE18_scaffold_616449_287|E_GD2017-2_strous-5-prefilter_S18_GE18_Biohub_170515_UNK|E_GD2017-2_strous-5-prefilter_S18_GE18_Biohub_180515 | 100.000 | 155 | 0 | 0 | 1 | 155 | 1 | 155 | 2.5E-108 | 315 |
| **S2_GD2017_2_manure_scaffold_3_410|GD2017-2_manure_QB3_180125_Potentially_complete_Phage_48_40|GD2017-2_manure_QB3_180125** | S16_GE16_scaffold_390213_391|E_GD2017-2_urea-3_S16_GE16_Biohub_170515_UNK|E_GD2017-2_urea-3_S16_GE16_Biohub_180515 | 100.000 | 155 | 0 | 0 | 1 | 155 | 1 | 155 | 2.5E-108 | 315 |
| **S2_GD2017_2_manure_scaffold_3_410|GD2017-2_manure_QB3_180125_Potentially_complete_Phage_48_40|GD2017-2_manure_QB3_180125** | P0_An_pond3_S8_170907_scaffold_1005865_42|E_GD2017-1_P0_AN_POND3_S8_BIOHUB_170907_UNK|E_GD2017-1_P0_An_pond3_S8_Biohub_170907 | 100.000 | 155 | 0 | 0 | 1 | 155 | 1 | 155 | 2.5E-108 | 315 |
| **S2_GD2017_2_manure_scaffold_3_410|GD2017-2_manure_QB3_180125_Potentially_complete_Phage_48_40|GD2017-2_manure_QB3_180125** | P0_An_GD2017L_S7_170907_scaffold_359857_25|E_GD2017-1_P0_An_GD2017L_S7_Biohub_170907_UNK|E_GD2017-1_P0_An_GD2017L_S7_Biohub_170907 | 100.000 | 155 | 0 | 0 | 1 | 155 | 1 | 155 | 2.5E-108 | 315 |
| **S2_GD2017_2_manure_scaffold_3_410|GD2017-2_manure_QB3_180125_Potentially_complete_Phage_48_40|GD2017-2_manure_QB3_180125** | S2_GD2017_2_manure_scaffold_3_prodigal-single_483|GD_PHAGE_COMPLETE_48_40|GD2017-2_manure_QB3_180125 | 100.000 | 155 | 0 | 0 | 1 | 155 | 1 | 155 | 2.5E-108 | 315 |
| **S2_GD2017_2_manure_scaffold_3_410|GD2017-2_manure_QB3_180125_Potentially_complete_Phage_48_40|GD2017-2_manure_QB3_180125** | S2_GD2017_2_manure_scaffold_3_410|GD2017-2_manure_QB3_180125_Potentially_complete_Phage_48_40|GD2017-2_manure_QB3_180125 | 100.000 | 155 | 0 | 0 | 1 | 155 | 1 | 155 | 2.5E-108 | 315 |
| **S2_GD2017_2_manure_scaffold_3_411|GD2017-2_manure_QB3_180125_Potentially_complete_Phage_48_40|GD2017-2_manure_QB3_180125** | S30_BME30_294872_46|BM_2017_Coates_4_Potentially_Complete_Phage_48-32|BM_2017_Coates_4 | 100.000 | 879 | 0 | 0 | 1 | 879 | 1 | 879 | 0.0 | 1796 |
| **S2_GD2017_2_manure_scaffold_3_411|GD2017-2_manure_QB3_180125_Potentially_complete_Phage_48_40|GD2017-2_manure_QB3_180125** | S28_BME28_138683_399|BM_2017_Strous_8_UNK|BM_2017_Strous_8 | 100.000 | 879 | 0 | 0 | 1 | 879 | 1 | 879 | 0.0 | 1796 |
| **S2_GD2017_2_manure_scaffold_3_411|GD2017-2_manure_QB3_180125_Potentially_complete_Phage_48_40|GD2017-2_manure_QB3_180125** | S27_BME27_629333_prodigal-single_183|BM_PHAGE_48_13|BM_2017_Strous_6 | 100.000 | 879 | 0 | 0 | 1 | 879 | 1 | 879 | 0.0 | 1796 |
| **S2_GD2017_2_manure_scaffold_3_411|GD2017-2_manure_QB3_180125_Potentially_complete_Phage_48_40|GD2017-2_manure_QB3_180125** | S27_BME27_629333_173|BM_2017_Strous_6_Phage_48_13|BM_2017_Strous_6 | 100.000 | 879 | 0 | 0 | 1 | 879 | 1 | 879 | 0.0 | 1796 |
| **S2_GD2017_2_manure_scaffold_3_411|GD2017-2_manure_QB3_180125_Potentially_complete_Phage_48_40|GD2017-2_manure_QB3_180125** | S33_3P_scaffold_263290_6|Genasci_Feb2018_S33_3P_UNK|Genasci_Feb2018_S33_3P | 100.000 | 879 | 0 | 0 | 1 | 879 | 1 | 879 | 0.0 | 1796 |
| **S2_GD2017_2_manure_scaffold_3_411|GD2017-2_manure_QB3_180125_Potentially_complete_Phage_48_40|GD2017-2_manure_QB3_180125** | S22_GE22_scaffold_285059_prodigal-single_516|GD_PHAGE_COMPLETE_48_49|E_GD2017-2_anammox-7_S22_GE22_Biohub_180515 | 100.000 | 879 | 0 | 0 | 1 | 879 | 1 | 879 | 0.0 | 1796 |
| **S2_GD2017_2_manure_scaffold_3_411|GD2017-2_manure_QB3_180125_Potentially_complete_Phage_48_40|GD2017-2_manure_QB3_180125** | S22_GE22_scaffold_285059_439|E_GD2017-2_anammox-7_S22_GE22_Biohub_170515_Potentially_Complete_48_49|E_GD2017-2_anammox-7_S22_GE22_Biohub_180515 | 100.000 | 879 | 0 | 0 | 1 | 879 | 1 | 879 | 0.0 | 1796 |
| **S2_GD2017_2_manure_scaffold_3_411|GD2017-2_manure_QB3_180125_Potentially_complete_Phage_48_40|GD2017-2_manure_QB3_180125** | S20_GE20_scaffold_396645_15|E_GD2017-2_strous-5_S20_GE20_Biohub_170515_UNK|E_GD2017-2_strous-5_S20_GE20_Biohub_180515 | 100.000 | 879 | 0 | 0 | 1 | 879 | 1 | 879 | 0.0 | 1796 |
| **S2_GD2017_2_manure_scaffold_3_411|GD2017-2_manure_QB3_180125_Potentially_complete_Phage_48_40|GD2017-2_manure_QB3_180125** | S15_GE15_scaffold_320047_241|E_GD2017-2_urea-2_S15_GE15_Biohub_170515_UNK|E_GD2017-2_urea-2_S15_GE15_Biohub_180515 | 100.000 | 879 | 0 | 0 | 1 | 879 | 1 | 879 | 0.0 | 1796 |
| **S2_GD2017_2_manure_scaffold_3_411|GD2017-2_manure_QB3_180125_Potentially_complete_Phage_48_40|GD2017-2_manure_QB3_180125** | S14_GE14_scaffold_311130_11|E_GD2017-2_urea-2_S14_GE14_Biohub_170515_UNK|E_GD2017-2_urea-2_S14_GE14_Biohub_180515 | 100.000 | 879 | 0 | 0 | 1 | 879 | 1 | 879 | 0.0 | 1796 |
| **S2_GD2017_2_manure_scaffold_3_411|GD2017-2_manure_QB3_180125_Potentially_complete_Phage_48_40|GD2017-2_manure_QB3_180125** | P0_An_pond3_S8_coassembly_k141_2723617_432|P0_An_pond3_S8_coassembly_UNK|E_GD2017-1_P0_An_pond3_S8_Biohub_coassembly | 100.000 | 879 | 0 | 0 | 1 | 879 | 1 | 879 | 0.0 | 1796 |
| **S2_GD2017_2_manure_scaffold_3_411|GD2017-2_manure_QB3_180125_Potentially_complete_Phage_48_40|GD2017-2_manure_QB3_180125** | S19_GE19_scaffold_2259_prodigal-single_279|GD_PHAGE_48_10|E_GD2017-2_strous-5-pellet_S19_GE19_Biohub_180515 | 100.000 | 879 | 0 | 0 | 1 | 879 | 1 | 879 | 0.0 | 1796 |
| **S2_GD2017_2_manure_scaffold_3_411|GD2017-2_manure_QB3_180125_Potentially_complete_Phage_48_40|GD2017-2_manure_QB3_180125** | S19_GE19_scaffold_2259_255|E_GD2017-2_strous-5-pellet_S19_GE19_Biohub_170515_Phage_48_10|E_GD2017-2_strous-5-pellet_S19_GE19_Biohub_180515 | 100.000 | 879 | 0 | 0 | 1 | 879 | 1 | 879 | 0.0 | 1796 |
| **S2_GD2017_2_manure_scaffold_3_411|GD2017-2_manure_QB3_180125_Potentially_complete_Phage_48_40|GD2017-2_manure_QB3_180125** | S18_GE18_scaffold_616449_286|E_GD2017-2_strous-5-prefilter_S18_GE18_Biohub_170515_UNK|E_GD2017-2_strous-5-prefilter_S18_GE18_Biohub_180515 | 100.000 | 879 | 0 | 0 | 1 | 879 | 1 | 879 | 0.0 | 1796 |
| **S2_GD2017_2_manure_scaffold_3_411|GD2017-2_manure_QB3_180125_Potentially_complete_Phage_48_40|GD2017-2_manure_QB3_180125** | S16_GE16_scaffold_390213_392|E_GD2017-2_urea-3_S16_GE16_Biohub_170515_UNK|E_GD2017-2_urea-3_S16_GE16_Biohub_180515 | 100.000 | 879 | 0 | 0 | 1 | 879 | 1 | 879 | 0.0 | 1796 |
| **S2_GD2017_2_manure_scaffold_3_411|GD2017-2_manure_QB3_180125_Potentially_complete_Phage_48_40|GD2017-2_manure_QB3_180125** | P0_An_pond3_S8_170907_scaffold_1005865_43|E_GD2017-1_P0_AN_POND3_S8_BIOHUB_170907_UNK|E_GD2017-1_P0_An_pond3_S8_Biohub_170907 | 100.000 | 879 | 0 | 0 | 1 | 879 | 1 | 879 | 0.0 | 1796 |
| **S2_GD2017_2_manure_scaffold_3_411|GD2017-2_manure_QB3_180125_Potentially_complete_Phage_48_40|GD2017-2_manure_QB3_180125** | P0_An_GD2017L_S7_170907_scaffold_359857_26|E_GD2017-1_P0_An_GD2017L_S7_Biohub_170907_UNK|E_GD2017-1_P0_An_GD2017L_S7_Biohub_170907 | 100.000 | 879 | 0 | 0 | 1 | 879 | 1 | 879 | 0.0 | 1796 |
| **S2_GD2017_2_manure_scaffold_3_411|GD2017-2_manure_QB3_180125_Potentially_complete_Phage_48_40|GD2017-2_manure_QB3_180125** | S2_GD2017_2_manure_scaffold_3_prodigal-single_484|GD_PHAGE_COMPLETE_48_40|GD2017-2_manure_QB3_180125 | 100.000 | 879 | 0 | 0 | 1 | 879 | 1 | 879 | 0.0 | 1796 |
| **S2_GD2017_2_manure_scaffold_3_411|GD2017-2_manure_QB3_180125_Potentially_complete_Phage_48_40|GD2017-2_manure_QB3_180125** | S2_GD2017_2_manure_scaffold_3_411|GD2017-2_manure_QB3_180125_Potentially_complete_Phage_48_40|GD2017-2_manure_QB3_180125 | 100.000 | 879 | 0 | 0 | 1 | 879 | 1 | 879 | 0.0 | 1796 |
| **S2_GD2017_2_manure_scaffold_3_411|GD2017-2_manure_QB3_180125_Potentially_complete_Phage_48_40|GD2017-2_manure_QB3_180125** | P0_An_GD2017L_S7_coassembly_k141_1013362_prodigal-single_78|GD_PHAGE_48_49|E_P0_An_GD2017L_S7_coassembly | 99.886 | 879 | 1 | 0 | 1 | 879 | 1 | 879 | 0.0 | 1795 |
| **S2_GD2017_2_manure_scaffold_3_411|GD2017-2_manure_QB3_180125_Potentially_complete_Phage_48_40|GD2017-2_manure_QB3_180125** | P0_An_GD2017L_S7_coassembly_k141_1013362_72|P0_An_GD2017L_S7_coassembly_Phage_48_49|E_P0_An_GD2017L_S7_coassembly | 99.886 | 879 | 1 | 0 | 1 | 879 | 1 | 879 | 0.0 | 1795 |
| **S2_GD2017_2_manure_scaffold_3_411|GD2017-2_manure_QB3_180125_Potentially_complete_Phage_48_40|GD2017-2_manure_QB3_180125** | SR-VP_0-2_scaffold_141_955116_1|SR-VP_0-2cm_UNK|SR-VP_0-2cm | 65.834 | 761 | 247 | 6 | 129 | 879 | 1 | 758 | 0.0 | 993 |
| **S2_GD2017_2_manure_scaffold_3_411|GD2017-2_manure_QB3_180125_Potentially_complete_Phage_48_40|GD2017-2_manure_QB3_180125** | SR-VP_4-6_scaffold_141_2216387_1|SR-VP_4-6cm_Biohub_180515_UNK|SR-VP_4-6cm_Biohub_180515 | 67.020 | 661 | 208 | 4 | 54 | 706 | 1 | 659 | 0.0 | 906 |
| **S2_GD2017_2_manure_scaffold_3_411|GD2017-2_manure_QB3_180125_Potentially_complete_Phage_48_40|GD2017-2_manure_QB3_180125** | GD18-4_manure_scaffold_687770_5|GD2018-4_manure_QB3_180703_UNK|GD2018-4_manure_QB3_180703 | 100.000 | 438 | 0 | 0 | 442 | 879 | 1 | 438 | 0.0 | 891 |
| **S2_GD2017_2_manure_scaffold_3_411|GD2017-2_manure_QB3_180125_Potentially_complete_Phage_48_40|GD2017-2_manure_QB3_180125** | GD18-4_manure_scaffold_793011_1|GD2018-4_manure_QB3_180703_UNK|GD2018-4_manure_QB3_180703 | 100.000 | 429 | 0 | 0 | 1 | 429 | 1 | 429 | 0.0 | 881 |
| **S2_GD2017_2_manure_scaffold_3_411|GD2017-2_manure_QB3_180125_Potentially_complete_Phage_48_40|GD2017-2_manure_QB3_180125** | SR-VP_4-6_scaffold_141_682911_2|SR-VP_4-6cm_Biohub_180515_UNK|SR-VP_4-6cm_Biohub_180515 | 45.494 | 466 | 225 | 10 | 423 | 876 | 297 | 745 | 2.42E-116 | 382 |
| **S2_GD2017_2_manure_scaffold_3_411|GD2017-2_manure_QB3_180125_Potentially_complete_Phage_48_40|GD2017-2_manure_QB3_180125** | SR-VP_4-6_scaffold_141_682911_2|SR-VP_4-6cm_Biohub_180515_UNK|SR-VP_4-6cm_Biohub_180515 | 41.108 | 343 | 154 | 11 | 4 | 337 | 3 | 306 | 1.86E-50 | 202 |
| **S2_GD2017_2_manure_scaffold_3_411|GD2017-2_manure_QB3_180125_Potentially_complete_Phage_48_40|GD2017-2_manure_QB3_180125** | SR-VP_0-2_scaffold_141_2432070_18|SR-VP_0-2cm_UNK|SR-VP_0-2cm | 44.276 | 463 | 235 | 9 | 423 | 876 | 297 | 745 | 3.36E-115 | 379 |
| **S2_GD2017_2_manure_scaffold_3_411|GD2017-2_manure_QB3_180125_Potentially_complete_Phage_48_40|GD2017-2_manure_QB3_180125** | SR-VP_0-2_scaffold_141_2432070_18|SR-VP_0-2cm_UNK|SR-VP_0-2cm | 41.108 | 343 | 154 | 11 | 4 | 337 | 3 | 306 | 1.9E-50 | 202 |
| **S2_GD2017_2_manure_scaffold_3_411|GD2017-2_manure_QB3_180125_Potentially_complete_Phage_48_40|GD2017-2_manure_QB3_180125** | SR-VP_0-2_scaffold_141_4671315_1|SR-VP_0-2cm_UNK|SR-VP_0-2cm | 41.529 | 484 | 248 | 12 | 336 | 808 | 161 | 620 | 6.58E-99 | 332 |
| **S2_GD2017_2_manure_scaffold_3_411|GD2017-2_manure_QB3_180125_Potentially_complete_Phage_48_40|GD2017-2_manure_QB3_180125** | S16_GE16_scaffold_5545_prodigal-single_29|GD_PHAGE_46_9|E_GD2017-2_urea-3_S16_GE16_Biohub_180515 | 35.614 | 570 | 318 | 12 | 324 | 877 | 386 | 922 | 1.31E-98 | 340 |
| **S2_GD2017_2_manure_scaffold_3_411|GD2017-2_manure_QB3_180125_Potentially_complete_Phage_48_40|GD2017-2_manure_QB3_180125** | S16_GE16_scaffold_5545_prodigal-single_29|GD_PHAGE_46_9|E_GD2017-2_urea-3_S16_GE16_Biohub_180515 | 50.610 | 164 | 72 | 2 | 5 | 167 | 3 | 158 | 1.58E-38 | 167 |
| **S2_GD2017_2_manure_scaffold_3_411|GD2017-2_manure_QB3_180125_Potentially_complete_Phage_48_40|GD2017-2_manure_QB3_180125** | S16_GE16_scaffold_5545_28|E_GD2017-2_urea-3_S16_GE16_Biohub_170515_Phage-like_46_9|E_GD2017-2_urea-3_S16_GE16_Biohub_180515 | 35.614 | 570 | 318 | 12 | 324 | 877 | 386 | 922 | 1.31E-98 | 340 |
| **S2_GD2017_2_manure_scaffold_3_411|GD2017-2_manure_QB3_180125_Potentially_complete_Phage_48_40|GD2017-2_manure_QB3_180125** | S16_GE16_scaffold_5545_28|E_GD2017-2_urea-3_S16_GE16_Biohub_170515_Phage-like_46_9|E_GD2017-2_urea-3_S16_GE16_Biohub_180515 | 50.610 | 164 | 72 | 2 | 5 | 167 | 3 | 158 | 1.58E-38 | 167 |
| **S2_GD2017_2_manure_scaffold_3_411|GD2017-2_manure_QB3_180125_Potentially_complete_Phage_48_40|GD2017-2_manure_QB3_180125** | S15_GE15_scaffold_39268_57|E_GD2017-2_urea-2_S15_GE15_Biohub_170515_UNK|E_GD2017-2_urea-2_S15_GE15_Biohub_180515 | 35.439 | 570 | 319 | 12 | 324 | 877 | 386 | 922 | 7.05E-98 | 338 |
| **S2_GD2017_2_manure_scaffold_3_411|GD2017-2_manure_QB3_180125_Potentially_complete_Phage_48_40|GD2017-2_manure_QB3_180125** | S15_GE15_scaffold_39268_57|E_GD2017-2_urea-2_S15_GE15_Biohub_170515_UNK|E_GD2017-2_urea-2_S15_GE15_Biohub_180515 | 50.610 | 164 | 72 | 2 | 5 | 167 | 3 | 158 | 1.58E-38 | 167 |
| **S2_GD2017_2_manure_scaffold_3_411|GD2017-2_manure_QB3_180125_Potentially_complete_Phage_48_40|GD2017-2_manure_QB3_180125** | P0_An_pond3_S8_coassembly_k141_1206453_2|P0_An_pond3_S8_coassembly_UNK|E_GD2017-1_P0_An_pond3_S8_Biohub_coassembly | 35.439 | 570 | 319 | 12 | 324 | 877 | 386 | 922 | 7.05E-98 | 338 |
| **S2_GD2017_2_manure_scaffold_3_411|GD2017-2_manure_QB3_180125_Potentially_complete_Phage_48_40|GD2017-2_manure_QB3_180125** | P0_An_pond3_S8_coassembly_k141_1206453_2|P0_An_pond3_S8_coassembly_UNK|E_GD2017-1_P0_An_pond3_S8_Biohub_coassembly | 50.610 | 164 | 72 | 2 | 5 | 167 | 3 | 158 | 1.58E-38 | 167 |
| **S2_GD2017_2_manure_scaffold_3_411|GD2017-2_manure_QB3_180125_Potentially_complete_Phage_48_40|GD2017-2_manure_QB3_180125** | L3a1_full_idba_ud_scaffold_6_230|L3a1_UNK|L3a1 | 43.172 | 454 | 236 | 6 | 435 | 877 | 484 | 926 | 2.29E-94 | 328 |
| **S2_GD2017_2_manure_scaffold_3_411|GD2017-2_manure_QB3_180125_Potentially_complete_Phage_48_40|GD2017-2_manure_QB3_180125** | L3a1_full_idba_ud_scaffold_6_230|L3a1_UNK|L3a1 | 50.867 | 173 | 76 | 2 | 3 | 174 | 4 | 168 | 4.7E-42 | 179 |
| **S2_GD2017_2_manure_scaffold_3_411|GD2017-2_manure_QB3_180125_Potentially_complete_Phage_48_40|GD2017-2_manure_QB3_180125** | PAFVLPS_2018_scaffold_18_355|viral-cat_UNK|viral-cat | 39.220 | 436 | 242 | 8 | 446 | 877 | 495 | 911 | 3.88E-92 | 322 |
| **S2_GD2017_2_manure_scaffold_3_411|GD2017-2_manure_QB3_180125_Potentially_complete_Phage_48_40|GD2017-2_manure_QB3_180125** | PAFVLPS_2018_scaffold_18_355|viral-cat_UNK|viral-cat | 55.128 | 156 | 69 | 1 | 15 | 169 | 5 | 160 | 4.38E-40 | 172 |
| **S2_GD2017_2_manure_scaffold_3_411|GD2017-2_manure_QB3_180125_Potentially_complete_Phage_48_40|GD2017-2_manure_QB3_180125** | PAFVLPS_2018_J30_2_scaffold_1899_4|soil-virus-P14-J30-2018_UNK|soil-virus-P14-J30-2018 | 39.220 | 436 | 242 | 8 | 446 | 877 | 495 | 911 | 3.88E-92 | 322 |
| **S2_GD2017_2_manure_scaffold_3_411|GD2017-2_manure_QB3_180125_Potentially_complete_Phage_48_40|GD2017-2_manure_QB3_180125** | PAFVLPS_2018_J30_2_scaffold_1899_4|soil-virus-P14-J30-2018_UNK|soil-virus-P14-J30-2018 | 55.128 | 156 | 69 | 1 | 15 | 169 | 5 | 160 | 4.38E-40 | 172 |
| **S2_GD2017_2_manure_scaffold_3_411|GD2017-2_manure_QB3_180125_Potentially_complete_Phage_48_40|GD2017-2_manure_QB3_180125** | PAFVLPS_2018_scaffold_187463_1|viral-cat_UNK|viral-cat | 36.679 | 548 | 313 | 12 | 335 | 877 | 385 | 903 | 3.15E-91 | 319 |
| **S2_GD2017_2_manure_scaffold_3_411|GD2017-2_manure_QB3_180125_Potentially_complete_Phage_48_40|GD2017-2_manure_QB3_180125** | PAFVLPS_2018_scaffold_187463_1|viral-cat_UNK|viral-cat | 54.545 | 165 | 66 | 2 | 5 | 168 | 3 | 159 | 5.27E-42 | 178 |
| **S2_GD2017_2_manure_scaffold_3_411|GD2017-2_manure_QB3_180125_Potentially_complete_Phage_48_40|GD2017-2_manure_QB3_180125** | PAFVLPS_2018_scaffold_187487_1|viral-cat_UNK|viral-cat | 36.314 | 548 | 315 | 12 | 335 | 877 | 385 | 903 | 3.71E-91 | 319 |
| **S2_GD2017_2_manure_scaffold_3_411|GD2017-2_manure_QB3_180125_Potentially_complete_Phage_48_40|GD2017-2_manure_QB3_180125** | PAFVLPS_2018_scaffold_187487_1|viral-cat_UNK|viral-cat | 55.152 | 165 | 65 | 2 | 5 | 168 | 3 | 159 | 2.45E-42 | 179 |
| **S2_GD2017_2_manure_scaffold_3_411|GD2017-2_manure_QB3_180125_Potentially_complete_Phage_48_40|GD2017-2_manure_QB3_180125** | PAFVLPS_2018_scaffold_33_43|circular_33|viral-cat | 37.162 | 444 | 253 | 9 | 446 | 877 | 489 | 918 | 6.95E-85 | 302 |
| **S2_GD2017_2_manure_scaffold_3_411|GD2017-2_manure_QB3_180125_Potentially_complete_Phage_48_40|GD2017-2_manure_QB3_180125** | PAFVLPS_2018_scaffold_33_43|circular_33|viral-cat | 55.152 | 165 | 65 | 2 | 5 | 168 | 3 | 159 | 4.34E-40 | 172 |
| **S2_GD2017_2_manure_scaffold_3_411|GD2017-2_manure_QB3_180125_Potentially_complete_Phage_48_40|GD2017-2_manure_QB3_180125** | PAFVLPS_2018_scaffold_577014_1|viral-cat_UNK|viral-cat | 35.824 | 522 | 304 | 10 | 334 | 852 | 31 | 524 | 3.52E-81 | 282 |
| **S2_GD2017_2_manure_scaffold_3_411|GD2017-2_manure_QB3_180125_Potentially_complete_Phage_48_40|GD2017-2_manure_QB3_180125** | S20_GE20_scaffold_68378_4|E_GD2017-2_strous-5_S20_GE20_Biohub_170515_UNK|E_GD2017-2_strous-5_S20_GE20_Biohub_180515 | 37.150 | 393 | 207 | 8 | 507 | 877 | 1 | 375 | 3.9E-73 | 256 |
| **S2_GD2017_2_manure_scaffold_3_411|GD2017-2_manure_QB3_180125_Potentially_complete_Phage_48_40|GD2017-2_manure_QB3_180125** | ERMGT615_2_curated_scaffold_18774_2|ERMGT615_2_Thaumarchaeota_Nitrosopumilales_38_11_curated|ERMGT615_2 | 41.873 | 363 | 190 | 10 | 527 | 877 | 1 | 354 | 5.89E-73 | 254 |
| **S2_GD2017_2_manure_scaffold_3_411|GD2017-2_manure_QB3_180125_Potentially_complete_Phage_48_40|GD2017-2_manure_QB3_180125** | ERMGT615_2_scaffold_18774_2|ERMGT615_2_Maxbin2_010|ERMGT615_2 | 41.873 | 363 | 190 | 10 | 527 | 877 | 1 | 354 | 5.89E-73 | 254 |
| **S2_GD2017_2_manure_scaffold_3_411|GD2017-2_manure_QB3_180125_Potentially_complete_Phage_48_40|GD2017-2_manure_QB3_180125** | PAFVLPS_2018_scaffold_1123371_1|viral-cat_UNK|viral-cat | 45.965 | 285 | 150 | 3 | 445 | 728 | 55 | 336 | 1.01E-71 | 251 |
| **S2_GD2017_2_manure_scaffold_3_411|GD2017-2_manure_QB3_180125_Potentially_complete_Phage_48_40|GD2017-2_manure_QB3_180125** | LacPavin_0419_WC70S_scaffold_436490_11|LacPavin_0419_WC70S_UNK|LacPavin_0419_WC70S | 34.302 | 516 | 298 | 14 | 375 | 872 | 248 | 740 | 2.13E-68 | 253 |
| **S2_GD2017_2_manure_scaffold_3_411|GD2017-2_manure_QB3_180125_Potentially_complete_Phage_48_40|GD2017-2_manure_QB3_180125** | ALT_03122018_0_1um_scaffold_34255_1|ALT_03122018_0_1um_UNK|ALT_03122018_0_1um | 37.173 | 382 | 215 | 7 | 446 | 820 | 1 | 364 | 6.18E-68 | 243 |
| **S2_GD2017_2_manure_scaffold_3_411|GD2017-2_manure_QB3_180125_Potentially_complete_Phage_48_40|GD2017-2_manure_QB3_180125** | PAFVLPS_2018_J31_2_scaffold_29704_1|soil-virus-P15-J31-2018_UNK|soil-virus-P15-J31-2018 | 43.165 | 278 | 155 | 2 | 448 | 724 | 3 | 278 | 1.06E-67 | 238 |
| **S2_GD2017_2_manure_scaffold_3_411|GD2017-2_manure_QB3_180125_Potentially_complete_Phage_48_40|GD2017-2_manure_QB3_180125** | SR-VP_0-2_scaffold_141_5191495_prodigal-single_74|SR-VP_PHAGE_38_25|SR-VP_0-2cm | 38.333 | 360 | 212 | 6 | 396 | 751 | 277 | 630 | 1.49E-66 | 249 |
| **S2_GD2017_2_manure_scaffold_3_411|GD2017-2_manure_QB3_180125_Potentially_complete_Phage_48_40|GD2017-2_manure_QB3_180125** | SR-VP_0-2_scaffold_141_5191495_prodigal-single_74|SR-VP_PHAGE_38_25|SR-VP_0-2cm | 36.508 | 252 | 135 | 6 | 1 | 241 | 1 | 238 | 2.62E-31 | 144 |
| **S2_GD2017_2_manure_scaffold_3_411|GD2017-2_manure_QB3_180125_Potentially_complete_Phage_48_40|GD2017-2_manure_QB3_180125** | SR-VP_0-2_scaffold_141_5191495_67|SR-VP_0-2cm_Phage_38_25|SR-VP_0-2cm | 38.333 | 360 | 212 | 6 | 396 | 751 | 277 | 630 | 1.49E-66 | 249 |
| **S2_GD2017_2_manure_scaffold_3_411|GD2017-2_manure_QB3_180125_Potentially_complete_Phage_48_40|GD2017-2_manure_QB3_180125** | SR-VP_0-2_scaffold_141_5191495_67|SR-VP_0-2cm_Phage_38_25|SR-VP_0-2cm | 36.508 | 252 | 135 | 6 | 1 | 241 | 1 | 238 | 2.62E-31 | 144 |
| **S2_GD2017_2_manure_scaffold_3_411|GD2017-2_manure_QB3_180125_Potentially_complete_Phage_48_40|GD2017-2_manure_QB3_180125** | S15_GE15_scaffold_109408_2|E_GD2017-2_urea-2_S15_GE15_Biohub_170515_UNK|E_GD2017-2_urea-2_S15_GE15_Biohub_180515 | 33.553 | 456 | 274 | 12 | 423 | 867 | 165 | 602 | 2.43E-63 | 236 |
| **S2_GD2017_2_manure_scaffold_3_411|GD2017-2_manure_QB3_180125_Potentially_complete_Phage_48_40|GD2017-2_manure_QB3_180125** | P0_An_pond3_S8_coassembly_k141_1182798_1|P0_An_pond3_S8_coassembly_UNK|E_GD2017-1_P0_An_pond3_S8_Biohub_coassembly | 33.553 | 456 | 274 | 12 | 423 | 867 | 304 | 741 | 2.13E-62 | 236 |
| **S2_GD2017_2_manure_scaffold_3_411|GD2017-2_manure_QB3_180125_Potentially_complete_Phage_48_40|GD2017-2_manure_QB3_180125** | P0_An_pond3_S8_coassembly_k141_1182798_1|P0_An_pond3_S8_coassembly_UNK|E_GD2017-1_P0_An_pond3_S8_Biohub_coassembly | 36.957 | 276 | 139 | 7 | 1 | 258 | 1 | 259 | 4.6E-35 | 156 |
| **S2_GD2017_2_manure_scaffold_3_411|GD2017-2_manure_QB3_180125_Potentially_complete_Phage_48_40|GD2017-2_manure_QB3_180125** | SR-VP_0-2_scaffold_141_6432269_1|SR-VP_0-2cm_UNK|SR-VP_0-2cm | 33.183 | 443 | 260 | 9 | 439 | 867 | 308 | 728 | 3.37E-62 | 236 |
| **S2_GD2017_2_manure_scaffold_3_411|GD2017-2_manure_QB3_180125_Potentially_complete_Phage_48_40|GD2017-2_manure_QB3_180125** | PAFVLPS_2018_J30_2_scaffold_29892_1|soil-virus-P14-J30-2018_UNK|soil-virus-P14-J30-2018 | 35.977 | 353 | 201 | 8 | 537 | 877 | 8 | 347 | 7.08E-62 | 224 |
| **S2_GD2017_2_manure_scaffold_3_411|GD2017-2_manure_QB3_180125_Potentially_complete_Phage_48_40|GD2017-2_manure_QB3_180125** | SR-VP_2-4_scaffold_141_646991_1|SR-VP_2-4cm_UNK|SR-VP_2-4cm | 37.261 | 314 | 193 | 3 | 440 | 751 | 34 | 345 | 4.41E-61 | 222 |
| **S2_GD2017_2_manure_scaffold_3_411|GD2017-2_manure_QB3_180125_Potentially_complete_Phage_48_40|GD2017-2_manure_QB3_180125** | GWB1_scaffold_1082_4|GWB1 | 34.141 | 454 | 252 | 13 | 444 | 873 | 312 | 742 | 2.16E-60 | 231 |
| **S2_GD2017_2_manure_scaffold_3_411|GD2017-2_manure_QB3_180125_Potentially_complete_Phage_48_40|GD2017-2_manure_QB3_180125** | GWB1_scaffold_1082_4|GWB1 | 46.061 | 165 | 80 | 2 | 4 | 167 | 2 | 158 | 4.91E-32 | 146 |
| **S2_GD2017_2_manure_scaffold_3_411|GD2017-2_manure_QB3_180125_Potentially_complete_Phage_48_40|GD2017-2_manure_QB3_180125** | PAFVLPS_2018_scaffold_46833_8|viral-cat_UNK|viral-cat | 33.487 | 433 | 265 | 9 | 336 | 763 | 226 | 640 | 6.17E-60 | 230 |
| **S2_GD2017_2_manure_scaffold_3_411|GD2017-2_manure_QB3_180125_Potentially_complete_Phage_48_40|GD2017-2_manure_QB3_180125** | PAFVLPS_2018_scaffold_46833_8|viral-cat_UNK|viral-cat | 35.878 | 262 | 141 | 7 | 1 | 251 | 1 | 246 | 8.69E-32 | 145 |
| **S2_GD2017_2_manure_scaffold_3_411|GD2017-2_manure_QB3_180125_Potentially_complete_Phage_48_40|GD2017-2_manure_QB3_180125** | LacPavin_0419_WC70S_scaffold_94267_2|LacPavin_0419_WC70S_UNK|LacPavin_0419_WC70S | 56.989 | 186 | 71 | 2 | 1 | 186 | 1 | 177 | 7.21E-60 | 218 |
| **S2_GD2017_2_manure_scaffold_3_411|GD2017-2_manure_QB3_180125_Potentially_complete_Phage_48_40|GD2017-2_manure_QB3_180125** | SR-VP_0-2_scaffold_141_6448357_8|SR-VP_0-2cm_UNK|SR-VP_0-2cm | 38.040 | 347 | 210 | 3 | 439 | 784 | 319 | 661 | 8.39E-60 | 229 |
| **S2_GD2017_2_manure_scaffold_3_411|GD2017-2_manure_QB3_180125_Potentially_complete_Phage_48_40|GD2017-2_manure_QB3_180125** | U2s1_full_idba_ud_scaffold_45754_1|U2s1_UNK|U2s1 | 45.935 | 246 | 131 | 2 | 423 | 667 | 129 | 373 | 5.56E-59 | 217 |
| **S2_GD2017_2_manure_scaffold_3_411|GD2017-2_manure_QB3_180125_Potentially_complete_Phage_48_40|GD2017-2_manure_QB3_180125** | L3m2_full_idba_ud_scaffold_11238_2|L3m2_concoct_70|L3m2 | 33.410 | 437 | 271 | 9 | 320 | 752 | 95 | 515 | 8.69E-59 | 222 |
| **S2_GD2017_2_manure_scaffold_3_411|GD2017-2_manure_QB3_180125_Potentially_complete_Phage_48_40|GD2017-2_manure_QB3_180125** | ERMGT157_2_scaffold_16082_2|ERMGT157_2_UNK|ERMGT157_2 | 33.708 | 445 | 257 | 11 | 446 | 867 | 90 | 519 | 4.93E-58 | 219 |
| **S2_GD2017_2_manure_scaffold_3_411|GD2017-2_manure_QB3_180125_Potentially_complete_Phage_48_40|GD2017-2_manure_QB3_180125** | SR-VP_0-2_scaffold_141_5112071_2|SR-VP_0-2cm_UNK|SR-VP_0-2cm | 30.837 | 454 | 281 | 8 | 437 | 867 | 317 | 760 | 7.26E-58 | 224 |
| **S2_GD2017_2_manure_scaffold_3_411|GD2017-2_manure_QB3_180125_Potentially_complete_Phage_48_40|GD2017-2_manure_QB3_180125** | SR-VP_0-2_scaffold_141_5112071_2|SR-VP_0-2cm_UNK|SR-VP_0-2cm | 37.405 | 262 | 137 | 7 | 1 | 251 | 1 | 246 | 3.96E-33 | 150 |
| **S2_GD2017_2_manure_scaffold_3_411|GD2017-2_manure_QB3_180125_Potentially_complete_Phage_48_40|GD2017-2_manure_QB3_180125** | PAFVLPS_2018_scaffold_386_5|viral-cat_UNK|viral-cat | 37.700 | 313 | 192 | 3 | 440 | 751 | 320 | 630 | 1.06E-57 | 223 |
| **S2_GD2017_2_manure_scaffold_3_411|GD2017-2_manure_QB3_180125_Potentially_complete_Phage_48_40|GD2017-2_manure_QB3_180125** | PAFVLPS_2018_scaffold_386_5|viral-cat_UNK|viral-cat | 36.260 | 262 | 140 | 7 | 1 | 251 | 1 | 246 | 1.96E-32 | 148 |
| **S2_GD2017_2_manure_scaffold_3_411|GD2017-2_manure_QB3_180125_Potentially_complete_Phage_48_40|GD2017-2_manure_QB3_180125** | L3m1_full_idba_ud_scaffold_1665_2|L3m1_UNK|L3m1 | 33.257 | 436 | 273 | 8 | 320 | 752 | 211 | 631 | 1.31E-57 | 221 |
| **S2_GD2017_2_manure_scaffold_3_411|GD2017-2_manure_QB3_180125_Potentially_complete_Phage_48_40|GD2017-2_manure_QB3_180125** | L3m1_full_idba_ud_scaffold_1665_2|L3m1_UNK|L3m1 | 36.900 | 271 | 126 | 7 | 1 | 251 | 1 | 246 | 9.76E-32 | 145 |
| **S2_GD2017_2_manure_scaffold_3_411|GD2017-2_manure_QB3_180125_Potentially_complete_Phage_48_40|GD2017-2_manure_QB3_180125** | SRVP18_trench_1_20cm_scaffold_10130_2|SRVP18_trench_1_20cm_UNK|SRVP18_trench_1_20cm | 30.530 | 547 | 338 | 14 | 333 | 867 | 222 | 738 | 3.29E-56 | 219 |
| **S2_GD2017_2_manure_scaffold_3_411|GD2017-2_manure_QB3_180125_Potentially_complete_Phage_48_40|GD2017-2_manure_QB3_180125** | SRVP18_trench_1_20cm_scaffold_10130_2|SRVP18_trench_1_20cm_UNK|SRVP18_trench_1_20cm | 36.111 | 288 | 158 | 6 | 4 | 281 | 3 | 274 | 1.22E-35 | 157 |
| **S2_GD2017_2_manure_scaffold_3_411|GD2017-2_manure_QB3_180125_Potentially_complete_Phage_48_40|GD2017-2_manure_QB3_180125** | SR-VP_2-4_scaffold_141_3693826_2|SR-VP_2-4cm_UNK|SR-VP_2-4cm | 43.382 | 272 | 135 | 7 | 617 | 877 | 4 | 267 | 1.44E-54 | 201 |
| **S2_GD2017_2_manure_scaffold_3_411|GD2017-2_manure_QB3_180125_Potentially_complete_Phage_48_40|GD2017-2_manure_QB3_180125** | PAFVLPS_2018_scaffold_895318_1|viral-cat_UNK|viral-cat | 33.146 | 356 | 209 | 8 | 459 | 789 | 2 | 353 | 1.47E-49 | 191 |
| **S2_GD2017_2_manure_scaffold_3_411|GD2017-2_manure_QB3_180125_Potentially_complete_Phage_48_40|GD2017-2_manure_QB3_180125** | P0_An_GD2017L_S7_coassembly_k141_2601098_1|P0_An_GD2017L_S7_coassembly_UNK|E_P0_An_GD2017L_S7_coassembly | 32.468 | 385 | 238 | 9 | 491 | 867 | 104 | 474 | 2.36E-49 | 194 |
| **S2_GD2017_2_manure_scaffold_3_411|GD2017-2_manure_QB3_180125_Potentially_complete_Phage_48_40|GD2017-2_manure_QB3_180125** | SR-VP_4-6_scaffold_141_2760307_1|SR-VP_4-6cm_Biohub_180515_UNK|SR-VP_4-6cm_Biohub_180515 | 65.823 | 158 | 51 | 2 | 724 | 879 | 1 | 157 | 8.77E-49 | 181 |
| **S2_GD2017_2_manure_scaffold_3_411|GD2017-2_manure_QB3_180125_Potentially_complete_Phage_48_40|GD2017-2_manure_QB3_180125** | BC_09192017_0_5m_scaffold_7473_2|BC_09192017_0_5m_UNK|BC_09192017_0_5m | 36.474 | 329 | 199 | 5 | 425 | 750 | 280 | 601 | 2.72E-48 | 198 |
| **S2_GD2017_2_manure_scaffold_3_411|GD2017-2_manure_QB3_180125_Potentially_complete_Phage_48_40|GD2017-2_manure_QB3_180125** | PAFVLPS_2018_J28_2_scaffold_69475_1|soil-virus-P12-J28-2018_UNK|soil-virus-P12-J28-2018 | 37.692 | 260 | 149 | 4 | 618 | 877 | 1 | 247 | 1.21E-47 | 181 |
| **S2_GD2017_2_manure_scaffold_3_411|GD2017-2_manure_QB3_180125_Potentially_complete_Phage_48_40|GD2017-2_manure_QB3_180125** | FFC_07242016_10_scaffold_18482_1|FFC_07242016_10_UNK|FFC_07242016_10 | 53.333 | 165 | 77 | 0 | 22 | 186 | 1 | 165 | 4.55E-47 | 183 |
| **S2_GD2017_2_manure_scaffold_3_411|GD2017-2_manure_QB3_180125_Potentially_complete_Phage_48_40|GD2017-2_manure_QB3_180125** | PAFVLPS_2018_scaffold_268418_2|viral-cat_UNK|viral-cat | 56.364 | 165 | 63 | 2 | 5 | 168 | 3 | 159 | 1.67E-45 | 181 |
| **S2_GD2017_2_manure_scaffold_3_411|GD2017-2_manure_QB3_180125_Potentially_complete_Phage_48_40|GD2017-2_manure_QB3_180125** | PLM4_65_coex_sep16_scaffold_112227_1|PLM4_65cm_coex_sep2016_UNK|PLM4_65cm_coex_sep2016 | 32.517 | 449 | 243 | 13 | 333 | 768 | 123 | 524 | 6.43E-45 | 183 |
| **S2_GD2017_2_manure_scaffold_3_411|GD2017-2_manure_QB3_180125_Potentially_complete_Phage_48_40|GD2017-2_manure_QB3_180125** | L3a2_full_idba_ud_scaffold_8334_4|L3a2_MaxBin2_EukRep_ggKbase_unknown_002|L3a2 | 49.730 | 185 | 81 | 3 | 3 | 183 | 4 | 180 | 6.15E-44 | 181 |
| **S2_GD2017_2_manure_scaffold_3_411|GD2017-2_manure_QB3_180125_Potentially_complete_Phage_48_40|GD2017-2_manure_QB3_180125** | AB_082018_0_1um_scaffold_2553_4|AB_082018_0_1um_UNK|AB_082018_0_1um | 34.043 | 329 | 207 | 5 | 425 | 750 | 280 | 601 | 7.78E-44 | 183 |
| **S2_GD2017_2_manure_scaffold_3_411|GD2017-2_manure_QB3_180125_Potentially_complete_Phage_48_40|GD2017-2_manure_QB3_180125** | S20_GE20_scaffold_118836_3|E_GD2017-2_strous-5_S20_GE20_Biohub_170515_UNK|E_GD2017-2_strous-5_S20_GE20_Biohub_180515 | 50.610 | 164 | 72 | 2 | 5 | 167 | 3 | 158 | 8.46E-44 | 169 |
| **S2_GD2017_2_manure_scaffold_3_411|GD2017-2_manure_QB3_180125_Potentially_complete_Phage_48_40|GD2017-2_manure_QB3_180125** | FFC_04162018_0_1um_scaffold_1491_2|FFC_04162018_0_1um_UNK|FFC_04162018_0_1um | 32.995 | 394 | 238 | 9 | 377 | 757 | 1278 | 1658 | 9E-44 | 185 |
| **S2_GD2017_2_manure_scaffold_3_411|GD2017-2_manure_QB3_180125_Potentially_complete_Phage_48_40|GD2017-2_manure_QB3_180125** | PAFVLPS_2018_J31_2_scaffold_29704_2|soil-virus-P15-J31-2018_UNK|soil-virus-P15-J31-2018 | 53.939 | 165 | 67 | 2 | 5 | 168 | 3 | 159 | 9.35E-44 | 176 |
| **S2_GD2017_2_manure_scaffold_3_411|GD2017-2_manure_QB3_180125_Potentially_complete_Phage_48_40|GD2017-2_manure_QB3_180125** | LacPavin_0419_WC53_scaffold_515305_2|LacPavin_0419_WC53_UNK|LacPavin_0419_WC53 | 33.628 | 339 | 212 | 6 | 425 | 757 | 280 | 611 | 3.51E-43 | 182 |
| **S2_GD2017_2_manure_scaffold_3_411|GD2017-2_manure_QB3_180125_Potentially_complete_Phage_48_40|GD2017-2_manure_QB3_180125** | LacPavin_0419_WC70S_scaffold_80622_1|LacPavin_0419_WC70S_UNK|LacPavin_0419_WC70S | 31.768 | 362 | 229 | 8 | 334 | 691 | 36 | 383 | 7.76E-42 | 169 |
| **S2_GD2017_2_manure_scaffold_3_411|GD2017-2_manure_QB3_180125_Potentially_complete_Phage_48_40|GD2017-2_manure_QB3_180125** | LacPavin_0818_WC45_scaffold_305942_32|LacPavin_0818_WC45_UNK|LacPavin_0818_WC45 | 33.243 | 367 | 229 | 7 | 429 | 788 | 285 | 642 | 7.05E-40 | 171 |
| **S2_GD2017_2_manure_scaffold_3_411|GD2017-2_manure_QB3_180125_Potentially_complete_Phage_48_40|GD2017-2_manure_QB3_180125** | PLM4_65_b1_redo_sep16_scaffold_358230_1|PLM4_65cm_b1_redo_sep2016_UNK|PLM4_65cm_b1_redo_sep2016 | 31.563 | 339 | 190 | 6 | 493 | 820 | 1 | 308 | 1.27E-39 | 162 |
| **S2_GD2017_2_manure_scaffold_3_411|GD2017-2_manure_QB3_180125_Potentially_complete_Phage_48_40|GD2017-2_manure_QB3_180125** | PAFVLPS_2018_scaffold_250137_1|viral-cat_UNK|viral-cat | 34.559 | 272 | 171 | 5 | 425 | 692 | 302 | 570 | 5.87E-39 | 165 |
| **S2_GD2017_2_manure_scaffold_3_411|GD2017-2_manure_QB3_180125_Potentially_complete_Phage_48_40|GD2017-2_manure_QB3_180125** | L3m1_full_idba_ud_scaffold_79967_2|L3m1_UNK|L3m1 | 32.659 | 346 | 196 | 8 | 547 | 867 | 21 | 354 | 2.18E-38 | 159 |
| **S2_GD2017_2_manure_scaffold_3_411|GD2017-2_manure_QB3_180125_Potentially_complete_Phage_48_40|GD2017-2_manure_QB3_180125** | P0_An_GD2017L_S7_coassembly_k141_1814973_2|P0_An_GD2017L_S7_coassembly_UNK|E_P0_An_GD2017L_S7_coassembly | 36.957 | 276 | 139 | 7 | 1 | 258 | 1 | 259 | 1.68E-37 | 156 |
| **S2_GD2017_2_manure_scaffold_3_411|GD2017-2_manure_QB3_180125_Potentially_complete_Phage_48_40|GD2017-2_manure_QB3_180125** | ERMLT660_2_scaffold_16732_1|ERMLT660_2_UNK|ERMLT660_2 | 39.910 | 223 | 130 | 3 | 424 | 643 | 190 | 411 | 7.6E-37 | 155 |
| **S2_GD2017_2_manure_scaffold_3_411|GD2017-2_manure_QB3_180125_Potentially_complete_Phage_48_40|GD2017-2_manure_QB3_180125** | S16_GE16_scaffold_524391_2|E_GD2017-2_urea-3_S16_GE16_Biohub_170515_UNK|E_GD2017-2_urea-3_S16_GE16_Biohub_180515 | 31.579 | 323 | 200 | 8 | 553 | 867 | 2 | 311 | 1.36E-36 | 152 |
| **S2_GD2017_2_manure_scaffold_3_411|GD2017-2_manure_QB3_180125_Potentially_complete_Phage_48_40|GD2017-2_manure_QB3_180125** | S16_GE16_scaffold_443094_1|E_GD2017-2_urea-3_S16_GE16_Biohub_170515_UNK|E_GD2017-2_urea-3_S16_GE16_Biohub_180515 | 36.765 | 272 | 137 | 7 | 5 | 258 | 2 | 256 | 1.12E-35 | 152 |
| **S2_GD2017_2_manure_scaffold_3_411|GD2017-2_manure_QB3_180125_Potentially_complete_Phage_48_40|GD2017-2_manure_QB3_180125** | BML_08042016_6_5m_scaffold_15601_1|BML_08042016_6_5m_UNK|BML_08042016_6_5m | 33.660 | 306 | 190 | 7 | 440 | 736 | 79 | 380 | 1.65E-35 | 154 |
| **S2_GD2017_2_manure_scaffold_3_411|GD2017-2_manure_QB3_180125_Potentially_complete_Phage_48_40|GD2017-2_manure_QB3_180125** | gwa1_scaffold_9117_17|GWA1 | 46.061 | 165 | 80 | 2 | 4 | 167 | 2 | 158 | 8.93E-35 | 146 |
| **S2_GD2017_2_manure_scaffold_3_411|GD2017-2_manure_QB3_180125_Potentially_complete_Phage_48_40|GD2017-2_manure_QB3_180125** | BML_coassembly_scaffold_632_29|BML_coassembly_UNK|BML_coassembly | 33.660 | 306 | 190 | 7 | 440 | 736 | 322 | 623 | 1.96E-34 | 154 |
| **S2_GD2017_2_manure_scaffold_3_411|GD2017-2_manure_QB3_180125_Potentially_complete_Phage_48_40|GD2017-2_manure_QB3_180125** | AB_092018_0_1um_scaffold_105197_1|AB_092018_0_1um_UNK|AB_092018_0_1um | 33.333 | 282 | 175 | 6 | 482 | 757 | 1 | 275 | 3.13E-34 | 147 |
| **S2_GD2017_2_manure_scaffold_3_411|GD2017-2_manure_QB3_180125_Potentially_complete_Phage_48_40|GD2017-2_manure_QB3_180125** | ERMGT828_2_scaffold_19588_2|ERMGT828_2_UNK|ERMGT828_2 | 39.113 | 248 | 127 | 6 | 5 | 241 | 6 | 240 | 3.52E-34 | 150 |
| **S2_GD2017_2_manure_scaffold_3_411|GD2017-2_manure_QB3_180125_Potentially_complete_Phage_48_40|GD2017-2_manure_QB3_180125** | ERMGT828_2_scaffold_19588_2|ERMGT828_2_UNK|ERMGT828_2 | 36.441 | 236 | 146 | 4 | 440 | 673 | 322 | 555 | 1.15E-33 | 149 |
| **S2_GD2017_2_manure_scaffold_3_411|GD2017-2_manure_QB3_180125_Potentially_complete_Phage_48_40|GD2017-2_manure_QB3_180125** | LacPavin_0419_WC70S_scaffold_945204_7|LacPavin_0419_WC70S_UNK|LacPavin_0419_WC70S | 31.715 | 309 | 202 | 4 | 446 | 751 | 298 | 600 | 3.55E-34 | 154 |
| **S2_GD2017_2_manure_scaffold_3_411|GD2017-2_manure_QB3_180125_Potentially_complete_Phage_48_40|GD2017-2_manure_QB3_180125** | LacPavin_0818_WC55_scaffold_113784_prodigal-single_370|LP_PHAGE_COMPLETE_34_34|LacPavin_0818_WC55 | 31.715 | 309 | 202 | 4 | 446 | 751 | 298 | 600 | 3.55E-34 | 154 |
| **S2_GD2017_2_manure_scaffold_3_411|GD2017-2_manure_QB3_180125_Potentially_complete_Phage_48_40|GD2017-2_manure_QB3_180125** | LacPavin_0818_WC55_scaffold_113784_359|LacPavin_0818_WC55_Potentially_Complete_Phage_34_34|LacPavin_0818_WC55 | 31.715 | 309 | 202 | 4 | 446 | 751 | 298 | 600 | 3.55E-34 | 154 |
| **S2_GD2017_2_manure_scaffold_3_411|GD2017-2_manure_QB3_180125_Potentially_complete_Phage_48_40|GD2017-2_manure_QB3_180125** | LacPavin_0818_WC45_scaffold_80267_119|LacPavin_0818_WC45_UNK|LacPavin_0818_WC45 | 31.715 | 309 | 202 | 4 | 446 | 751 | 298 | 600 | 3.55E-34 | 154 |
| **S2_GD2017_2_manure_scaffold_3_411|GD2017-2_manure_QB3_180125_Potentially_complete_Phage_48_40|GD2017-2_manure_QB3_180125** | LacPavin_0718_WC55_scaffold_0_31|LacPavin_0718_WC55_Phage_34_15|LacPavin_0718_WC55 | 31.715 | 309 | 202 | 4 | 446 | 751 | 298 | 600 | 3.55E-34 | 154 |
| **S2_GD2017_2_manure_scaffold_3_411|GD2017-2_manure_QB3_180125_Potentially_complete_Phage_48_40|GD2017-2_manure_QB3_180125** | LacPavin_0718_WC45_scaffold_3_119|LacPavin_0718_WC45_UNK|LacPavin_0718_WC45 | 31.715 | 309 | 202 | 4 | 446 | 751 | 298 | 600 | 3.55E-34 | 154 |
| **S2_GD2017_2_manure_scaffold_3_411|GD2017-2_manure_QB3_180125_Potentially_complete_Phage_48_40|GD2017-2_manure_QB3_180125** | P0_An_GD2017L_S7_coassembly_k141_2564368_1|P0_An_GD2017L_S7_coassembly_UNK|E_P0_An_GD2017L_S7_coassembly | 49.296 | 142 | 63 | 2 | 5 | 145 | 3 | 136 | 4.74E-34 | 139 |
| **S2_GD2017_2_manure_scaffold_3_412|GD2017-2_manure_QB3_180125_Potentially_complete_Phage_48_40|GD2017-2_manure_QB3_180125** | GD18-4_manure_scaffold_687770_4|GD2018-4_manure_QB3_180703_UNK|GD2018-4_manure_QB3_180703 | 100.000 | 224 | 0 | 0 | 1 | 224 | 1 | 224 | 7.53E-164 | 461 |
| **S2_GD2017_2_manure_scaffold_3_412|GD2017-2_manure_QB3_180125_Potentially_complete_Phage_48_40|GD2017-2_manure_QB3_180125** | S30_BME30_294872_45|BM_2017_Coates_4_Potentially_Complete_Phage_48-32|BM_2017_Coates_4 | 100.000 | 224 | 0 | 0 | 1 | 224 | 1 | 224 | 7.53E-164 | 461 |
| **S2_GD2017_2_manure_scaffold_3_412|GD2017-2_manure_QB3_180125_Potentially_complete_Phage_48_40|GD2017-2_manure_QB3_180125** | S28_BME28_138683_400|BM_2017_Strous_8_UNK|BM_2017_Strous_8 | 100.000 | 224 | 0 | 0 | 1 | 224 | 1 | 224 | 7.53E-164 | 461 |
| **S2_GD2017_2_manure_scaffold_3_412|GD2017-2_manure_QB3_180125_Potentially_complete_Phage_48_40|GD2017-2_manure_QB3_180125** | S27_BME27_629333_prodigal-single_182|BM_PHAGE_48_13|BM_2017_Strous_6 | 100.000 | 224 | 0 | 0 | 1 | 224 | 1 | 224 | 7.53E-164 | 461 |
| **S2_GD2017_2_manure_scaffold_3_412|GD2017-2_manure_QB3_180125_Potentially_complete_Phage_48_40|GD2017-2_manure_QB3_180125** | S27_BME27_629333_172|BM_2017_Strous_6_Phage_48_13|BM_2017_Strous_6 | 100.000 | 224 | 0 | 0 | 1 | 224 | 1 | 224 | 7.53E-164 | 461 |
| **S2_GD2017_2_manure_scaffold_3_412|GD2017-2_manure_QB3_180125_Potentially_complete_Phage_48_40|GD2017-2_manure_QB3_180125** | S33_3P_scaffold_263290_7|Genasci_Feb2018_S33_3P_UNK|Genasci_Feb2018_S33_3P | 100.000 | 224 | 0 | 0 | 1 | 224 | 1 | 224 | 7.53E-164 | 461 |
| **S2_GD2017_2_manure_scaffold_3_412|GD2017-2_manure_QB3_180125_Potentially_complete_Phage_48_40|GD2017-2_manure_QB3_180125** | S22_GE22_scaffold_285059_prodigal-single_517|GD_PHAGE_COMPLETE_48_49|E_GD2017-2_anammox-7_S22_GE22_Biohub_180515 | 100.000 | 224 | 0 | 0 | 1 | 224 | 1 | 224 | 7.53E-164 | 461 |
| **S2_GD2017_2_manure_scaffold_3_412|GD2017-2_manure_QB3_180125_Potentially_complete_Phage_48_40|GD2017-2_manure_QB3_180125** | S22_GE22_scaffold_285059_440|E_GD2017-2_anammox-7_S22_GE22_Biohub_170515_Potentially_Complete_48_49|E_GD2017-2_anammox-7_S22_GE22_Biohub_180515 | 100.000 | 224 | 0 | 0 | 1 | 224 | 1 | 224 | 7.53E-164 | 461 |
| **S2_GD2017_2_manure_scaffold_3_412|GD2017-2_manure_QB3_180125_Potentially_complete_Phage_48_40|GD2017-2_manure_QB3_180125** | S20_GE20_scaffold_396645_16|E_GD2017-2_strous-5_S20_GE20_Biohub_170515_UNK|E_GD2017-2_strous-5_S20_GE20_Biohub_180515 | 100.000 | 224 | 0 | 0 | 1 | 224 | 1 | 224 | 7.53E-164 | 461 |
| **S2_GD2017_2_manure_scaffold_3_412|GD2017-2_manure_QB3_180125_Potentially_complete_Phage_48_40|GD2017-2_manure_QB3_180125** | S15_GE15_scaffold_320047_240|E_GD2017-2_urea-2_S15_GE15_Biohub_170515_UNK|E_GD2017-2_urea-2_S15_GE15_Biohub_180515 | 100.000 | 224 | 0 | 0 | 1 | 224 | 1 | 224 | 7.53E-164 | 461 |
| **S2_GD2017_2_manure_scaffold_3_412|GD2017-2_manure_QB3_180125_Potentially_complete_Phage_48_40|GD2017-2_manure_QB3_180125** | S14_GE14_scaffold_311130_10|E_GD2017-2_urea-2_S14_GE14_Biohub_170515_UNK|E_GD2017-2_urea-2_S14_GE14_Biohub_180515 | 100.000 | 224 | 0 | 0 | 1 | 224 | 1 | 224 | 7.53E-164 | 461 |
| **S2_GD2017_2_manure_scaffold_3_412|GD2017-2_manure_QB3_180125_Potentially_complete_Phage_48_40|GD2017-2_manure_QB3_180125** | P0_An_pond3_S8_coassembly_k141_2723617_431|P0_An_pond3_S8_coassembly_UNK|E_GD2017-1_P0_An_pond3_S8_Biohub_coassembly | 100.000 | 224 | 0 | 0 | 1 | 224 | 1 | 224 | 7.53E-164 | 461 |
| **S2_GD2017_2_manure_scaffold_3_412|GD2017-2_manure_QB3_180125_Potentially_complete_Phage_48_40|GD2017-2_manure_QB3_180125** | P0_An_GD2017L_S7_coassembly_k141_1013362_prodigal-single_77|GD_PHAGE_48_49|E_P0_An_GD2017L_S7_coassembly | 100.000 | 224 | 0 | 0 | 1 | 224 | 1 | 224 | 7.53E-164 | 461 |
| **S2_GD2017_2_manure_scaffold_3_412|GD2017-2_manure_QB3_180125_Potentially_complete_Phage_48_40|GD2017-2_manure_QB3_180125** | P0_An_GD2017L_S7_coassembly_k141_1013362_71|P0_An_GD2017L_S7_coassembly_Phage_48_49|E_P0_An_GD2017L_S7_coassembly | 100.000 | 224 | 0 | 0 | 1 | 224 | 1 | 224 | 7.53E-164 | 461 |
| **S2_GD2017_2_manure_scaffold_3_412|GD2017-2_manure_QB3_180125_Potentially_complete_Phage_48_40|GD2017-2_manure_QB3_180125** | S19_GE19_scaffold_2259_prodigal-single_278|GD_PHAGE_48_10|E_GD2017-2_strous-5-pellet_S19_GE19_Biohub_180515 | 100.000 | 224 | 0 | 0 | 1 | 224 | 1 | 224 | 7.53E-164 | 461 |
| **S2_GD2017_2_manure_scaffold_3_412|GD2017-2_manure_QB3_180125_Potentially_complete_Phage_48_40|GD2017-2_manure_QB3_180125** | S19_GE19_scaffold_2259_254|E_GD2017-2_strous-5-pellet_S19_GE19_Biohub_170515_Phage_48_10|E_GD2017-2_strous-5-pellet_S19_GE19_Biohub_180515 | 100.000 | 224 | 0 | 0 | 1 | 224 | 1 | 224 | 7.53E-164 | 461 |
| **S2_GD2017_2_manure_scaffold_3_412|GD2017-2_manure_QB3_180125_Potentially_complete_Phage_48_40|GD2017-2_manure_QB3_180125** | S18_GE18_scaffold_616449_285|E_GD2017-2_strous-5-prefilter_S18_GE18_Biohub_170515_UNK|E_GD2017-2_strous-5-prefilter_S18_GE18_Biohub_180515 | 100.000 | 224 | 0 | 0 | 1 | 224 | 1 | 224 | 7.53E-164 | 461 |
| **S2_GD2017_2_manure_scaffold_3_412|GD2017-2_manure_QB3_180125_Potentially_complete_Phage_48_40|GD2017-2_manure_QB3_180125** | S16_GE16_scaffold_390213_393|E_GD2017-2_urea-3_S16_GE16_Biohub_170515_UNK|E_GD2017-2_urea-3_S16_GE16_Biohub_180515 | 100.000 | 224 | 0 | 0 | 1 | 224 | 1 | 224 | 7.53E-164 | 461 |
| **S2_GD2017_2_manure_scaffold_3_412|GD2017-2_manure_QB3_180125_Potentially_complete_Phage_48_40|GD2017-2_manure_QB3_180125** | P0_An_pond3_S8_170907_scaffold_1005865_44|E_GD2017-1_P0_AN_POND3_S8_BIOHUB_170907_UNK|E_GD2017-1_P0_An_pond3_S8_Biohub_170907 | 100.000 | 224 | 0 | 0 | 1 | 224 | 1 | 224 | 7.53E-164 | 461 |
| **S2_GD2017_2_manure_scaffold_3_412|GD2017-2_manure_QB3_180125_Potentially_complete_Phage_48_40|GD2017-2_manure_QB3_180125** | S2_GD2017_2_manure_scaffold_3_prodigal-single_485|GD_PHAGE_COMPLETE_48_40|GD2017-2_manure_QB3_180125 | 100.000 | 224 | 0 | 0 | 1 | 224 | 1 | 224 | 7.53E-164 | 461 |
| **S2_GD2017_2_manure_scaffold_3_412|GD2017-2_manure_QB3_180125_Potentially_complete_Phage_48_40|GD2017-2_manure_QB3_180125** | S2_GD2017_2_manure_scaffold_3_412|GD2017-2_manure_QB3_180125_Potentially_complete_Phage_48_40|GD2017-2_manure_QB3_180125 | 100.000 | 224 | 0 | 0 | 1 | 224 | 1 | 224 | 7.53E-164 | 461 |
| **S2_GD2017_2_manure_scaffold_3_412|GD2017-2_manure_QB3_180125_Potentially_complete_Phage_48_40|GD2017-2_manure_QB3_180125** | P0_An_GD2017L_S7_170907_scaffold_359857_27|E_GD2017-1_P0_An_GD2017L_S7_Biohub_170907_UNK|E_GD2017-1_P0_An_GD2017L_S7_Biohub_170907 | 100.000 | 162 | 0 | 0 | 1 | 162 | 1 | 162 | 7.2E-116 | 337 |
| **S2_GD2017_2_manure_scaffold_3_412|GD2017-2_manure_QB3_180125_Potentially_complete_Phage_48_40|GD2017-2_manure_QB3_180125** | SR-VP_4-6_scaffold_141_2760307_3|SR-VP_4-6cm_Biohub_180515_UNK|SR-VP_4-6cm_Biohub_180515 | 59.193 | 223 | 87 | 4 | 2 | 224 | 5 | 223 | 2.69E-81 | 252 |
| **S2_GD2017_2_manure_scaffold_3_412|GD2017-2_manure_QB3_180125_Potentially_complete_Phage_48_40|GD2017-2_manure_QB3_180125** | SR-VP_0-2_scaffold_141_955116_3|SR-VP_0-2cm_UNK|SR-VP_0-2cm | 59.193 | 223 | 87 | 4 | 2 | 224 | 5 | 223 | 2.69E-81 | 252 |
| **S2_GD2017_2_manure_scaffold_3_412|GD2017-2_manure_QB3_180125_Potentially_complete_Phage_48_40|GD2017-2_manure_QB3_180125** | L3a1_full_idba_ud_scaffold_87338_2|L3a1_UNK|L3a1 | 60.345 | 174 | 67 | 2 | 2 | 175 | 7 | 178 | 2.48E-70 | 223 |
| **S2_GD2017_2_manure_scaffold_3_412|GD2017-2_manure_QB3_180125_Potentially_complete_Phage_48_40|GD2017-2_manure_QB3_180125** | L3a2_full_idba_ud_scaffold_8334_6|L3a2_MaxBin2_EukRep_ggKbase_unknown_002|L3a2 | 52.381 | 210 | 97 | 3 | 2 | 211 | 7 | 213 | 1.66E-67 | 217 |
| **S2_GD2017_2_manure_scaffold_3_412|GD2017-2_manure_QB3_180125_Potentially_complete_Phage_48_40|GD2017-2_manure_QB3_180125** | L3a1_full_idba_ud_scaffold_6_231|L3a1_UNK|L3a1 | 52.381 | 210 | 97 | 3 | 2 | 211 | 7 | 213 | 1.95E-67 | 217 |
| **S2_GD2017_2_manure_scaffold_3_412|GD2017-2_manure_QB3_180125_Potentially_complete_Phage_48_40|GD2017-2_manure_QB3_180125** | SR-VP_4-6_scaffold_141_682911_3|SR-VP_4-6cm_Biohub_180515_UNK|SR-VP_4-6cm_Biohub_180515 | 49.524 | 210 | 103 | 3 | 1 | 210 | 1 | 207 | 5.38E-65 | 210 |
| **S2_GD2017_2_manure_scaffold_3_412|GD2017-2_manure_QB3_180125_Potentially_complete_Phage_48_40|GD2017-2_manure_QB3_180125** | SR-VP_0-2_scaffold_141_2432070_19|SR-VP_0-2cm_UNK|SR-VP_0-2cm | 49.524 | 210 | 103 | 3 | 1 | 210 | 1 | 207 | 7.39E-65 | 210 |
| **S2_GD2017_2_manure_scaffold_3_412|GD2017-2_manure_QB3_180125_Potentially_complete_Phage_48_40|GD2017-2_manure_QB3_180125** | PAFVLPS_2018_scaffold_18_354|viral-cat_UNK|viral-cat | 42.647 | 204 | 107 | 4 | 2 | 202 | 8 | 204 | 5.94E-48 | 167 |
| **S2_GD2017_2_manure_scaffold_3_412|GD2017-2_manure_QB3_180125_Potentially_complete_Phage_48_40|GD2017-2_manure_QB3_180125** | PAFVLPS_2018_J30_2_scaffold_1899_5|soil-virus-P14-J30-2018_UNK|soil-virus-P14-J30-2018 | 42.647 | 204 | 107 | 4 | 2 | 202 | 8 | 204 | 5.94E-48 | 167 |
| **S2_GD2017_2_manure_scaffold_3_412|GD2017-2_manure_QB3_180125_Potentially_complete_Phage_48_40|GD2017-2_manure_QB3_180125** | PAFVLPS_2018_scaffold_33_44|circular_33|viral-cat | 41.791 | 201 | 113 | 4 | 2 | 202 | 5 | 201 | 3.79E-45 | 160 |
| **S2_GD2017_2_manure_scaffold_3_412|GD2017-2_manure_QB3_180125_Potentially_complete_Phage_48_40|GD2017-2_manure_QB3_180125** | S16_GE16_scaffold_5545_prodigal-single_31|GD_PHAGE_46_9|E_GD2017-2_urea-3_S16_GE16_Biohub_180515 | 43.229 | 192 | 102 | 5 | 2 | 193 | 5 | 189 | 2.24E-44 | 157 |
| **S2_GD2017_2_manure_scaffold_3_412|GD2017-2_manure_QB3_180125_Potentially_complete_Phage_48_40|GD2017-2_manure_QB3_180125** | S16_GE16_scaffold_5545_30|E_GD2017-2_urea-3_S16_GE16_Biohub_170515_Phage-like_46_9|E_GD2017-2_urea-3_S16_GE16_Biohub_180515 | 43.229 | 192 | 102 | 5 | 2 | 193 | 5 | 189 | 2.24E-44 | 157 |
| **S2_GD2017_2_manure_scaffold_3_412|GD2017-2_manure_QB3_180125_Potentially_complete_Phage_48_40|GD2017-2_manure_QB3_180125** | S15_GE15_scaffold_39268_54|E_GD2017-2_urea-2_S15_GE15_Biohub_170515_UNK|E_GD2017-2_urea-2_S15_GE15_Biohub_180515 | 44.944 | 178 | 94 | 4 | 2 | 179 | 5 | 178 | 6.45E-44 | 157 |
| **S2_GD2017_2_manure_scaffold_3_412|GD2017-2_manure_QB3_180125_Potentially_complete_Phage_48_40|GD2017-2_manure_QB3_180125** | PAFVLPS_2018_J30_2_scaffold_29892_2|soil-virus-P14-J30-2018_UNK|soil-virus-P14-J30-2018 | 41.709 | 199 | 112 | 4 | 2 | 200 | 5 | 199 | 1.31E-43 | 158 |
| **S2_GD2017_2_manure_scaffold_3_412|GD2017-2_manure_QB3_180125_Potentially_complete_Phage_48_40|GD2017-2_manure_QB3_180125** | PAFVLPS_2018_scaffold_477083_1|viral-cat_UNK|viral-cat | 39.801 | 201 | 117 | 4 | 6 | 206 | 3 | 199 | 4.04E-43 | 155 |
| **S2_GD2017_2_manure_scaffold_3_412|GD2017-2_manure_QB3_180125_Potentially_complete_Phage_48_40|GD2017-2_manure_QB3_180125** | P0_An_GD2017L_S7_170907_scaffold_575547_3|E_GD2017-1_P0_An_GD2017L_S7_Biohub_170907_UNK|E_GD2017-1_P0_An_GD2017L_S7_Biohub_170907 | 100.000 | 67 | 0 | 0 | 158 | 224 | 1 | 67 | 4.65E-38 | 137 |
| **S2_GD2017_2_manure_scaffold_3_412|GD2017-2_manure_QB3_180125_Potentially_complete_Phage_48_40|GD2017-2_manure_QB3_180125** | BC_09192017_0_5m_scaffold_15350_3|BC_09192017_0_5m_UNK|BC_09192017_0_5m | 39.896 | 193 | 112 | 3 | 1 | 191 | 1 | 191 | 1.31E-37 | 140 |
| **S2_GD2017_2_manure_scaffold_3_412|GD2017-2_manure_QB3_180125_Potentially_complete_Phage_48_40|GD2017-2_manure_QB3_180125** | P0_An_GD2017L_S7_170907_scaffold_2243732_5|E_GD2017-1_P0_An_GD2017L_S7_Biohub_170907_UNK|E_GD2017-1_P0_An_GD2017L_S7_Biohub_170907 | 40.704 | 199 | 110 | 4 | 1 | 195 | 1 | 195 | 9.94E-37 | 138 |
| **S2_GD2017_2_manure_scaffold_3_412|GD2017-2_manure_QB3_180125_Potentially_complete_Phage_48_40|GD2017-2_manure_QB3_180125** | P0_An_pond3_S8_coassembly_k141_276347_5|P0_An_pond3_S8_coassembly_UNK|E_GD2017-1_P0_An_pond3_S8_Biohub_coassembly | 40.704 | 199 | 110 | 4 | 1 | 195 | 1 | 195 | 1E-36 | 138 |
| **S2_GD2017_2_manure_scaffold_3_412|GD2017-2_manure_QB3_180125_Potentially_complete_Phage_48_40|GD2017-2_manure_QB3_180125** | P0_An_GD2017L_S7_coassembly_k141_961924_10|P0_An_GD2017L_S7_coassembly_UNK|E_P0_An_GD2017L_S7_coassembly | 40.704 | 199 | 110 | 4 | 1 | 195 | 1 | 195 | 1E-36 | 138 |
| **S2_GD2017_2_manure_scaffold_3_412|GD2017-2_manure_QB3_180125_Potentially_complete_Phage_48_40|GD2017-2_manure_QB3_180125** | P0_An_pond3_S8_170907_scaffold_875447_5|E_GD2017-1_P0_AN_POND3_S8_BIOHUB_170907_UNK|E_GD2017-1_P0_An_pond3_S8_Biohub_170907 | 40.704 | 199 | 110 | 4 | 1 | 195 | 1 | 195 | 1E-36 | 138 |
| **S2_GD2017_2_manure_scaffold_3_412|GD2017-2_manure_QB3_180125_Potentially_complete_Phage_48_40|GD2017-2_manure_QB3_180125** | SR-VP_2-4_scaffold_141_3693826_1|SR-VP_2-4cm_UNK|SR-VP_2-4cm | 66.304 | 92 | 30 | 1 | 2 | 92 | 7 | 98 | 8.36E-36 | 132 |
| **S2_GD2017_2_manure_scaffold_3_412|GD2017-2_manure_QB3_180125_Potentially_complete_Phage_48_40|GD2017-2_manure_QB3_180125** | BML_09012016_9m_scaffold_53149_1|BML_09012016_9m_UNK|BML_09012016_9m | 38.614 | 202 | 115 | 5 | 2 | 201 | 7 | 201 | 1.27E-35 | 135 |
| **S2_GD2017_2_manure_scaffold_3_412|GD2017-2_manure_QB3_180125_Potentially_complete_Phage_48_40|GD2017-2_manure_QB3_180125** | BML_08022017_1_5m_scaffold_12516_2|BML_08022017_1_5m_UNK|BML_08022017_1_5m | 38.614 | 202 | 115 | 5 | 2 | 201 | 7 | 201 | 1.27E-35 | 135 |
| **S2_GD2017_2_manure_scaffold_3_412|GD2017-2_manure_QB3_180125_Potentially_complete_Phage_48_40|GD2017-2_manure_QB3_180125** | BML_08042016_6_5m_scaffold_11517_4|BML_08042016_6_5m_UNK|BML_08042016_6_5m | 38.614 | 202 | 115 | 5 | 2 | 201 | 7 | 201 | 1.27E-35 | 135 |
| **S2_GD2017_2_manure_scaffold_3_412|GD2017-2_manure_QB3_180125_Potentially_complete_Phage_48_40|GD2017-2_manure_QB3_180125** | BML_coassembly_scaffold_632_28|BML_coassembly_UNK|BML_coassembly | 38.614 | 202 | 115 | 5 | 2 | 201 | 7 | 201 | 1.27E-35 | 135 |
| **S2_GD2017_2_manure_scaffold_3_412|GD2017-2_manure_QB3_180125_Potentially_complete_Phage_48_40|GD2017-2_manure_QB3_180125** | BC_09192017_0_5m_scaffold_2201_5|BC_09192017_0_5m_UNK|BC_09192017_0_5m | 39.062 | 192 | 113 | 3 | 2 | 191 | 7 | 196 | 2.36E-34 | 132 |
| **S2_GD2017_2_manure_scaffold_3_412|GD2017-2_manure_QB3_180125_Potentially_complete_Phage_48_40|GD2017-2_manure_QB3_180125** | LacPavin_0818_WC40_scaffold_819229_36|LacPavin_0818_WC40_UNK|LacPavin_0818_WC40 | 38.298 | 188 | 111 | 4 | 2 | 187 | 7 | 191 | 1.34E-33 | 130 |
| **S2_GD2017_2_manure_scaffold_3_412|GD2017-2_manure_QB3_180125_Potentially_complete_Phage_48_40|GD2017-2_manure_QB3_180125** | FFC_04162018_0_1um_scaffold_401_2|FFC_04162018_0_1um_UNK|FFC_04162018_0_1um | 36.866 | 217 | 126 | 6 | 2 | 212 | 7 | 218 | 2.45E-33 | 130 |
| **S2_GD2017_2_manure_scaffold_3_412|GD2017-2_manure_QB3_180125_Potentially_complete_Phage_48_40|GD2017-2_manure_QB3_180125** | BC_09192017_0_5m_scaffold_1685_3|BC_09192017_0_5m_UNK|BC_09192017_0_5m | 35.000 | 220 | 129 | 5 | 2 | 214 | 7 | 219 | 7.04E-33 | 129 |
| **S2_GD2017_2_manure_scaffold_3_412|GD2017-2_manure_QB3_180125_Potentially_complete_Phage_48_40|GD2017-2_manure_QB3_180125** | FFC_04162018_0_1um_scaffold_383_3|FFC_04162018_0_1um_UNK|FFC_04162018_0_1um | 36.979 | 192 | 115 | 5 | 3 | 191 | 8 | 196 | 1.17E-32 | 128 |
| **S2_GD2017_2_manure_scaffold_3_412|GD2017-2_manure_QB3_180125_Potentially_complete_Phage_48_40|GD2017-2_manure_QB3_180125** | LacPavin_0419_WC70S_scaffold_945204_8|LacPavin_0419_WC70S_UNK|LacPavin_0419_WC70S | 40.123 | 162 | 92 | 4 | 2 | 163 | 7 | 163 | 2.91E-32 | 127 |
| **S2_GD2017_2_manure_scaffold_3_412|GD2017-2_manure_QB3_180125_Potentially_complete_Phage_48_40|GD2017-2_manure_QB3_180125** | LacPavin_0818_WC55_scaffold_113784_prodigal-single_371|LP_PHAGE_COMPLETE_34_34|LacPavin_0818_WC55 | 40.123 | 162 | 92 | 4 | 2 | 163 | 7 | 163 | 2.91E-32 | 127 |
| **S2_GD2017_2_manure_scaffold_3_412|GD2017-2_manure_QB3_180125_Potentially_complete_Phage_48_40|GD2017-2_manure_QB3_180125** | LacPavin_0818_WC55_scaffold_113784_360|LacPavin_0818_WC55_Potentially_Complete_Phage_34_34|LacPavin_0818_WC55 | 40.123 | 162 | 92 | 4 | 2 | 163 | 7 | 163 | 2.91E-32 | 127 |
| **S2_GD2017_2_manure_scaffold_3_412|GD2017-2_manure_QB3_180125_Potentially_complete_Phage_48_40|GD2017-2_manure_QB3_180125** | LacPavin_0818_WC45_scaffold_80267_120|LacPavin_0818_WC45_UNK|LacPavin_0818_WC45 | 40.123 | 162 | 92 | 4 | 2 | 163 | 7 | 163 | 2.91E-32 | 127 |
| **S2_GD2017_2_manure_scaffold_3_412|GD2017-2_manure_QB3_180125_Potentially_complete_Phage_48_40|GD2017-2_manure_QB3_180125** | LacPavin_0718_WC55_scaffold_0_30|LacPavin_0718_WC55_Phage_34_15|LacPavin_0718_WC55 | 40.123 | 162 | 92 | 4 | 2 | 163 | 7 | 163 | 2.91E-32 | 127 |
| **S2_GD2017_2_manure_scaffold_3_412|GD2017-2_manure_QB3_180125_Potentially_complete_Phage_48_40|GD2017-2_manure_QB3_180125** | LacPavin_0718_WC45_scaffold_3_120|LacPavin_0718_WC45_UNK|LacPavin_0718_WC45 | 40.123 | 162 | 92 | 4 | 2 | 163 | 7 | 163 | 2.91E-32 | 127 |
| **S2_GD2017_2_manure_scaffold_3_412|GD2017-2_manure_QB3_180125_Potentially_complete_Phage_48_40|GD2017-2_manure_QB3_180125** | BC_09192017_0_5m_scaffold_2747_26|BC_09192017_0_5m_UNK|BC_09192017_0_5m | 39.560 | 182 | 106 | 3 | 12 | 191 | 1 | 180 | 4.77E-32 | 126 |
| **S2_GD2017_2_manure_scaffold_3_412|GD2017-2_manure_QB3_180125_Potentially_complete_Phage_48_40|GD2017-2_manure_QB3_180125** | PAFVLPS_2018_J28_2_scaffold_69475_2|soil-virus-P12-J28-2018_UNK|soil-virus-P12-J28-2018 | 41.844 | 141 | 78 | 3 | 2 | 142 | 5 | 141 | 1.74E-31 | 123 |

| **SR-VP_0-2_scaffold_141_2510002_86|SR-VP_0-2cm_Jumbo_Phage_48_23|SR-VP_0-2cm** | SR-VP_4-6_scaffold_141_5680703_prodigal-single_435|SR-VP_PHAGE_48_20|SR-VP_4-6cm_Biohub_180515 | 100.000 | 958 | 0 | 0 | 1 | 958 | 1 | 958 | 0.0 | 1987 |
| --- | --- | --- | --- | --- | --- | --- | --- | --- | --- | --- | --- |
| **SR-VP_0-2_scaffold_141_2510002_86|SR-VP_0-2cm_Jumbo_Phage_48_23|SR-VP_0-2cm** | SR-VP_4-6_scaffold_141_5680703_425|SR-VP_4-6cm_Biohub_180515_Phage_48_20|SR-VP_4-6cm_Biohub_180515 | 100.000 | 958 | 0 | 0 | 1 | 958 | 1 | 958 | 0.0 | 1987 |
| **SR-VP_0-2_scaffold_141_2510002_86|SR-VP_0-2cm_Jumbo_Phage_48_23|SR-VP_0-2cm** | SR-VP_0-2_scaffold_141_2510002_prodigal-single_88|SR-VP_PHAGE_48_23_A|SR-VP_0-2cm | 100.000 | 958 | 0 | 0 | 1 | 958 | 1 | 958 | 0.0 | 1987 |
| **SR-VP_0-2_scaffold_141_2510002_86|SR-VP_0-2cm_Jumbo_Phage_48_23|SR-VP_0-2cm** | SR-VP_0-2_scaffold_141_2510002_86|SR-VP_0-2cm_Jumbo_Phage_48_23|SR-VP_0-2cm | 100.000 | 958 | 0 | 0 | 1 | 958 | 1 | 958 | 0.0 | 1987 |
| **SR-VP_0-2_scaffold_141_2510002_86|SR-VP_0-2cm_Jumbo_Phage_48_23|SR-VP_0-2cm** | SRVP18_trench_2_45cm_scaffold_8879_6|SRVP18_trench_2_45cm_UNK|SRVP18_trench_2_45cm | 100.000 | 293 | 0 | 0 | 1 | 293 | 1 | 293 | 0.0 | 613 |
| **SR-VP_0-2_scaffold_141_2510002_86|SR-VP_0-2cm_Jumbo_Phage_48_23|SR-VP_0-2cm** | PAFVLPS_2018_scaffold_181917_5|viral-cat_UNK|viral-cat | 54.630 | 540 | 237 | 4 | 31 | 563 | 24 | 562 | 0.0 | 605 |
| **SR-VP_0-2_scaffold_141_2510002_86|SR-VP_0-2cm_Jumbo_Phage_48_23|SR-VP_0-2cm** | SRVP18_trench_2_45cm_scaffold_9776_3|SRVP18_trench_2_45cm_UNK|SRVP18_trench_2_45cm | 100.000 | 273 | 0 | 0 | 686 | 958 | 1 | 273 | 0.0 | 566 |
| **SR-VP_0-2_scaffold_141_2510002_86|SR-VP_0-2cm_Jumbo_Phage_48_23|SR-VP_0-2cm** | PAFVLPS_2018_scaffold_449630_8|viral-cat_UNK|viral-cat | 59.375 | 448 | 179 | 3 | 482 | 927 | 21 | 467 | 0.0 | 543 |
| **SR-VP_0-2_scaffold_141_2510002_86|SR-VP_0-2cm_Jumbo_Phage_48_23|SR-VP_0-2cm** | SR-VP_0-2_scaffold_141_1145840_10|SR-VP_0-2cm_UNK|SR-VP_0-2cm | 52.711 | 461 | 200 | 8 | 125 | 570 | 17 | 474 | 1.2E-152 | 473 |
| **SR-VP_0-2_scaffold_141_2510002_86|SR-VP_0-2cm_Jumbo_Phage_48_23|SR-VP_0-2cm** | ALT_072018_0_1um_scaffold_1497_14|ALT_072018_0_1um_UNK|ALT_072018_0_1um | 44.600 | 500 | 265 | 6 | 69 | 564 | 32 | 523 | 3.79E-128 | 413 |
| **SR-VP_0-2_scaffold_141_2510002_86|SR-VP_0-2cm_Jumbo_Phage_48_23|SR-VP_0-2cm** | ALT_03122018_0_1um_scaffold_1137_10|ALT_03122018_0_1um_UNK|ALT_03122018_0_1um | 44.600 | 500 | 265 | 6 | 69 | 564 | 32 | 523 | 3.79E-128 | 413 |
| **SR-VP_0-2_scaffold_141_2510002_86|SR-VP_0-2cm_Jumbo_Phage_48_23|SR-VP_0-2cm** | ALT_082018_0_1um_scaffold_5003_4|ALT_082018_0_1um_UNK|ALT_082018_0_1um | 44.600 | 500 | 265 | 6 | 69 | 564 | 32 | 523 | 3.79E-128 | 413 |
| **SR-VP_0-2_scaffold_141_2510002_86|SR-VP_0-2cm_Jumbo_Phage_48_23|SR-VP_0-2cm** | SRVP18_trench_3_45cm_scaffold_58634_1|SRVP18_trench_3_45cm_UNK|SRVP18_trench_3_45cm | 52.975 | 353 | 158 | 5 | 515 | 862 | 1 | 350 | 2.25E-114 | 366 |
| **SR-VP_0-2_scaffold_141_2510002_86|SR-VP_0-2cm_Jumbo_Phage_48_23|SR-VP_0-2cm** | SRVP18_hole-7m-from-trench_1_80cm_scaffold_11972_2|SRVP18_hole-7m-from-trench_1_80cm_UNK|SRVP18_hole-7m-from-trench_1_80cm | 39.382 | 518 | 283 | 12 | 69 | 570 | 1 | 503 | 2.02E-101 | 340 |
| **SR-VP_0-2_scaffold_141_2510002_86|SR-VP_0-2cm_Jumbo_Phage_48_23|SR-VP_0-2cm** | BML_08182015_8_5m_scaffold_100_77|BML_08182015_8_5m_UNK|BML_08182015_8_5m | 36.449 | 535 | 320 | 8 | 48 | 570 | 65 | 591 | 5.34E-101 | 342 |
| **SR-VP_0-2_scaffold_141_2510002_86|SR-VP_0-2cm_Jumbo_Phage_48_23|SR-VP_0-2cm** | BML_08182015_6_5m_scaffold_4816_1|BML_08182015_6_5m_UNK|BML_08182015_6_5m | 36.449 | 535 | 320 | 8 | 48 | 570 | 65 | 591 | 5.34E-101 | 342 |
| **SR-VP_0-2_scaffold_141_2510002_86|SR-VP_0-2cm_Jumbo_Phage_48_23|SR-VP_0-2cm** | BML_coassembly_scaffold_727_2|BML_coassembly_UNK|BML_coassembly | 36.449 | 535 | 320 | 8 | 48 | 570 | 65 | 591 | 5.34E-101 | 342 |
| **SR-VP_0-2_scaffold_141_2510002_86|SR-VP_0-2cm_Jumbo_Phage_48_23|SR-VP_0-2cm** | SRVP18_trench_8_20cm_scaffold_11_9|SRVP18_trench_8_20cm_UNK|SRVP18_trench_8_20cm | 48.378 | 370 | 174 | 5 | 132 | 485 | 252 | 620 | 3.28E-98 | 336 |
| **SR-VP_0-2_scaffold_141_2510002_86|SR-VP_0-2cm_Jumbo_Phage_48_23|SR-VP_0-2cm** | SRVP18_trench_3_45cm_scaffold_31_9|SRVP18_trench_3_45cm_UNK|SRVP18_trench_3_45cm | 48.378 | 370 | 174 | 5 | 132 | 485 | 252 | 620 | 3.28E-98 | 336 |
| **SR-VP_0-2_scaffold_141_2510002_86|SR-VP_0-2cm_Jumbo_Phage_48_23|SR-VP_0-2cm** | SRVP18_trench_5_45cm_scaffold_22_9|SRVP18_trench_5_45cm_UNK|SRVP18_trench_5_45cm | 48.378 | 370 | 174 | 5 | 132 | 485 | 252 | 620 | 3.28E-98 | 336 |
| **SR-VP_0-2_scaffold_141_2510002_86|SR-VP_0-2cm_Jumbo_Phage_48_23|SR-VP_0-2cm** | SRVP18_trench_1_20cm_scaffold_29_37|SRVP18_trench_1_20cm_UNK|SRVP18_trench_1_20cm | 48.378 | 370 | 174 | 5 | 132 | 485 | 252 | 620 | 3.28E-98 | 336 |
| **SR-VP_0-2_scaffold_141_2510002_86|SR-VP_0-2cm_Jumbo_Phage_48_23|SR-VP_0-2cm** | SRVP18_trench_2_45cm_scaffold_17_232|SRVP18_trench_2_45cm_UNK|SRVP18_trench_2_45cm | 48.378 | 370 | 174 | 5 | 132 | 485 | 252 | 620 | 3.28E-98 | 336 |
| **SR-VP_0-2_scaffold_141_2510002_86|SR-VP_0-2cm_Jumbo_Phage_48_23|SR-VP_0-2cm** | SRVP18_trench_1_45cm_scaffold_9_9|SRVP18_trench_1_45cm_UNK|SRVP18_trench_1_45cm | 48.378 | 370 | 174 | 5 | 132 | 485 | 252 | 620 | 3.28E-98 | 336 |
| **SR-VP_0-2_scaffold_141_2510002_86|SR-VP_0-2cm_Jumbo_Phage_48_23|SR-VP_0-2cm** | SRVP18_trench_4_45cm_scaffold_9_232|SRVP18_trench_4_45cm_UNK|SRVP18_trench_4_45cm | 48.378 | 370 | 174 | 5 | 132 | 485 | 252 | 620 | 3.28E-98 | 336 |
| **SR-VP_0-2_scaffold_141_2510002_86|SR-VP_0-2cm_Jumbo_Phage_48_23|SR-VP_0-2cm** | SR-VP_0-2_scaffold_141_1201955_1|SR-VP_0-2cm_UNK|SR-VP_0-2cm | 40.821 | 463 | 248 | 10 | 51 | 495 | 55 | 509 | 4.42E-98 | 328 |
| **SR-VP_0-2_scaffold_141_2510002_86|SR-VP_0-2cm_Jumbo_Phage_48_23|SR-VP_0-2cm** | SR-VP_0-2_scaffold_141_3920600_3|SR-VP_0-2cm_UNK|SR-VP_0-2cm | 47.838 | 370 | 176 | 5 | 132 | 485 | 252 | 620 | 6.64E-96 | 330 |
| **SR-VP_0-2_scaffold_141_2510002_86|SR-VP_0-2cm_Jumbo_Phage_48_23|SR-VP_0-2cm** | DGJ12_scaffold_4241_5|DGJ12_UNK|DGJ12 | 47.554 | 368 | 171 | 5 | 130 | 490 | 153 | 505 | 7.31E-96 | 332 |
| **SR-VP_0-2_scaffold_141_2510002_86|SR-VP_0-2cm_Jumbo_Phage_48_23|SR-VP_0-2cm** | P0_An_pond3_S8_coassembly_k141_1114945_57|P0_An_pond3_S8_coassembly_UNK|E_GD2017-1_P0_An_pond3_S8_Biohub_coassembly | 39.381 | 485 | 267 | 8 | 91 | 563 | 184 | 653 | 5.01E-92 | 320 |
| **SR-VP_0-2_scaffold_141_2510002_86|SR-VP_0-2cm_Jumbo_Phage_48_23|SR-VP_0-2cm** | P0_An_GD2017L_S7_coassembly_k141_3927569_8|P0_An_GD2017L_S7_coassembly_UNK|E_P0_An_GD2017L_S7_coassembly | 39.381 | 485 | 267 | 8 | 91 | 563 | 184 | 653 | 5.01E-92 | 320 |
| **SR-VP_0-2_scaffold_141_2510002_86|SR-VP_0-2cm_Jumbo_Phage_48_23|SR-VP_0-2cm** | P0_An_pond3_S8_170907_scaffold_305759_11|E_GD2017-1_P0_AN_POND3_S8_BIOHUB_170907_UNK|E_GD2017-1_P0_An_pond3_S8_Biohub_170907 | 39.381 | 485 | 267 | 8 | 91 | 563 | 184 | 653 | 5.01E-92 | 320 |
| **SR-VP_0-2_scaffold_141_2510002_86|SR-VP_0-2cm_Jumbo_Phage_48_23|SR-VP_0-2cm** | P0_An_GD2017L_S7_170907_scaffold_13450_11|E_GD2017-1_P0_An_GD2017L_S7_Biohub_170907_UNK|E_GD2017-1_P0_An_GD2017L_S7_Biohub_170907 | 39.381 | 485 | 267 | 8 | 91 | 563 | 184 | 653 | 5.01E-92 | 320 |
| **SR-VP_0-2_scaffold_141_2510002_86|SR-VP_0-2cm_Jumbo_Phage_48_23|SR-VP_0-2cm** | SR-VP_2-4_scaffold_141_4801625_22|SR-VP_2-4cm_UNK|SR-VP_2-4cm | 37.783 | 442 | 250 | 7 | 142 | 563 | 1 | 437 | 5.58E-92 | 314 |
| **SR-VP_0-2_scaffold_141_2510002_86|SR-VP_0-2cm_Jumbo_Phage_48_23|SR-VP_0-2cm** | rattlesnake_spring_18_scaffold_81133_443|rattlesnake_spring_18_Phage_42_13|rattlesnake_spring_18 | 46.006 | 363 | 182 | 6 | 136 | 491 | 51 | 406 | 4.96E-89 | 317 |
| **SR-VP_0-2_scaffold_141_2510002_86|SR-VP_0-2cm_Jumbo_Phage_48_23|SR-VP_0-2cm** | SRVP18_trench_2_45cm_scaffold_9776_4|SRVP18_trench_2_45cm_UNK|SRVP18_trench_2_45cm | 99.265 | 136 | 1 | 0 | 484 | 619 | 1 | 136 | 2.64E-85 | 281 |
| **SR-VP_0-2_scaffold_141_2510002_86|SR-VP_0-2cm_Jumbo_Phage_48_23|SR-VP_0-2cm** | ar11r2_scaffold_25218_1|AlumRock_MS11_UNK|ALUMROCK_MS11 | 48.640 | 331 | 159 | 6 | 155 | 480 | 1 | 325 | 1.29E-84 | 292 |
| **SR-VP_0-2_scaffold_141_2510002_86|SR-VP_0-2cm_Jumbo_Phage_48_23|SR-VP_0-2cm** | S2_012_000_R2_scaffold_23925_3|S2_012_000_R2_UNK|S2_012_000_R2 | 43.597 | 367 | 193 | 6 | 133 | 491 | 28 | 388 | 1.36E-84 | 290 |
| **SR-VP_0-2_scaffold_141_2510002_86|SR-VP_0-2cm_Jumbo_Phage_48_23|SR-VP_0-2cm** | PLM2_5_b1_jun17_scaffold_5857_9|PLM2_5cm_b1_jun2017_UNK|PLM2_5cm_b1_jun2017 | 44.875 | 361 | 185 | 5 | 136 | 489 | 51 | 404 | 1.96E-84 | 303 |
| **SR-VP_0-2_scaffold_141_2510002_86|SR-VP_0-2cm_Jumbo_Phage_48_23|SR-VP_0-2cm** | LacPavin_0818_WC50_scaffold_1103828_11|LacPavin_0818_WC50_UNK|LacPavin_0818_WC50 | 42.056 | 428 | 224 | 8 | 125 | 535 | 256 | 676 | 2.72E-84 | 301 |
| **SR-VP_0-2_scaffold_141_2510002_86|SR-VP_0-2cm_Jumbo_Phage_48_23|SR-VP_0-2cm** | S2_009_000_R2_scaffold_49907_1|S2_009_000_R2_UNK|S2_009_000_R2 | 43.597 | 367 | 193 | 6 | 133 | 491 | 28 | 388 | 3.09E-84 | 290 |
| **SR-VP_0-2_scaffold_141_2510002_86|SR-VP_0-2cm_Jumbo_Phage_48_23|SR-VP_0-2cm** | PAFVLPS_2018_scaffold_1108955_1|viral-cat_UNK|viral-cat | 45.104 | 337 | 179 | 3 | 114 | 445 | 5 | 340 | 6.54E-84 | 285 |
| **SR-VP_0-2_scaffold_141_2510002_86|SR-VP_0-2cm_Jumbo_Phage_48_23|SR-VP_0-2cm** | PAFVLPS_2018_scaffold_92_101|circular_92|viral-cat | 38.990 | 495 | 266 | 12 | 53 | 540 | 172 | 637 | 1.32E-83 | 297 |
| **SR-VP_0-2_scaffold_141_2510002_86|SR-VP_0-2cm_Jumbo_Phage_48_23|SR-VP_0-2cm** | PAFVLPS_2018_J31_2_scaffold_2574_5|soil-virus-P15-J31-2018_UNK|soil-virus-P15-J31-2018 | 38.990 | 495 | 266 | 12 | 53 | 540 | 172 | 637 | 1.32E-83 | 297 |
| **SR-VP_0-2_scaffold_141_2510002_86|SR-VP_0-2cm_Jumbo_Phage_48_23|SR-VP_0-2cm** | Salt_Pond_R2_restored_DShore_MG_scaffold_14405_1|JGI_Salt_Pond_R2_restored_DShore_MG_UNK|JGI_Salt_Pond_R2_restored_DShore_MG | 45.682 | 359 | 176 | 5 | 133 | 490 | 15 | 355 | 2.92E-83 | 291 |
| **SR-VP_0-2_scaffold_141_2510002_86|SR-VP_0-2cm_Jumbo_Phage_48_23|SR-VP_0-2cm** | RifSed_csp2_10ft_3_scaffold_6_prodigal-single_257|RIF_PHAGE_35_13|RifSed_csp2_10ft_3 | 37.743 | 514 | 269 | 15 | 89 | 563 | 186 | 687 | 1.24E-82 | 295 |
| **SR-VP_0-2_scaffold_141_2510002_86|SR-VP_0-2cm_Jumbo_Phage_48_23|SR-VP_0-2cm** | RifSed_csp2_10ft_3_scaffold_6_240|RifSed_csp2_10ft_3_Phage-like_35_13|RifSed_csp2_10ft_3 | 37.743 | 514 | 269 | 15 | 89 | 563 | 186 | 687 | 1.24E-82 | 295 |
| **SR-VP_0-2_scaffold_141_2510002_86|SR-VP_0-2cm_Jumbo_Phage_48_23|SR-VP_0-2cm** | water-treatment_AWTP-2_inf_bulk_2_scaffold_32577_1|AWTP-2_inf_bulk_2_UNK|AWTP-2_inf_bulk_2 | 42.857 | 364 | 188 | 9 | 132 | 481 | 30 | 387 | 1.1E-81 | 281 |
| **SR-VP_0-2_scaffold_141_2510002_86|SR-VP_0-2cm_Jumbo_Phage_48_23|SR-VP_0-2cm** | GWB1_scaffold_652_110|GWB1_phage|GWB1 | 44.125 | 383 | 196 | 6 | 137 | 507 | 30 | 406 | 2.24E-79 | 288 |
| **SR-VP_0-2_scaffold_141_2510002_86|SR-VP_0-2cm_Jumbo_Phage_48_23|SR-VP_0-2cm** | gwa1_scaffold_2561_9|GWA1 | 44.125 | 383 | 196 | 6 | 137 | 507 | 30 | 406 | 2.24E-79 | 288 |
| **SR-VP_0-2_scaffold_141_2510002_86|SR-VP_0-2cm_Jumbo_Phage_48_23|SR-VP_0-2cm** | rifcsplowo2_12_scaffold_227342_1|RifCSPlowO2_12_FULL_UNK|RIFCSPLOWO2_12_FULL | 40.701 | 371 | 205 | 5 | 115 | 481 | 118 | 477 | 3.5E-79 | 277 |
| **SR-VP_0-2_scaffold_141_2510002_86|SR-VP_0-2cm_Jumbo_Phage_48_23|SR-VP_0-2cm** | S2_GD2017_2_manure_scaffold_69903_1|GD2017-2_manure_UNK|GD2017-2_manure_QB3_180125 | 42.000 | 400 | 197 | 10 | 115 | 487 | 45 | 436 | 3.86E-79 | 276 |
| **SR-VP_0-2_scaffold_141_2510002_86|SR-VP_0-2cm_Jumbo_Phage_48_23|SR-VP_0-2cm** | CG10_big_fil_rev_8_21_14_0.10_scaffold_6509_5|CG10_big_fil_rev_8_21_14_0_10_UNK|CG10_big_fil_rev_8_21_14_0_10 | 42.480 | 379 | 202 | 6 | 113 | 487 | 108 | 474 | 4.62E-79 | 283 |
| **SR-VP_0-2_scaffold_141_2510002_86|SR-VP_0-2cm_Jumbo_Phage_48_23|SR-VP_0-2cm** | JBCH_GD18_4_SO2_S177_scaffold_203583_1|JBCH_GD18_4_SO2_S177_UNK|GD2018-4_Sp2_QB3_180703 | 40.701 | 371 | 205 | 5 | 115 | 481 | 20 | 379 | 8.37E-79 | 274 |
| **SR-VP_0-2_scaffold_141_2510002_86|SR-VP_0-2cm_Jumbo_Phage_48_23|SR-VP_0-2cm** | water-treatment_AWTP-2_inf_bulk_2_scaffold_95_8|AWTP-2_inf_bulk_2_UNK|AWTP-2_inf_bulk_2 | 43.467 | 375 | 189 | 10 | 124 | 481 | 15 | 383 | 8.65E-79 | 285 |
| **SR-VP_0-2_scaffold_141_2510002_86|SR-VP_0-2cm_Jumbo_Phage_48_23|SR-VP_0-2cm** | rifoxyc1_full_scaffold_58681_1|RifOxyC1_full_UNK|RIFOXYC1_FULL | 44.759 | 353 | 178 | 5 | 139 | 479 | 1 | 348 | 2.04E-78 | 272 |
| **SR-VP_0-2_scaffold_141_2510002_86|SR-VP_0-2cm_Jumbo_Phage_48_23|SR-VP_0-2cm** | H16_Tanzania_scaffold_58_15|H16_Tanzania_UNK|H16_Tanzania | 40.863 | 394 | 212 | 7 | 131 | 517 | 29 | 408 | 3.1E-78 | 285 |
| **SR-VP_0-2_scaffold_141_2510002_86|SR-VP_0-2cm_Jumbo_Phage_48_23|SR-VP_0-2cm** | SR-VP_4-6_scaffold_141_6270851_2|SR-VP_4-6cm_Biohub_180515_UNK|SR-VP_4-6cm_Biohub_180515 | 44.764 | 382 | 178 | 10 | 139 | 493 | 141 | 516 | 3.67E-78 | 284 |
| **SR-VP_0-2_scaffold_141_2510002_86|SR-VP_0-2cm_Jumbo_Phage_48_23|SR-VP_0-2cm** | pig_ID_885_F41_scaffold_78983_2|pig_ID_885_F41_UNK|pig_ID_885_F41 | 42.659 | 361 | 188 | 5 | 132 | 481 | 29 | 381 | 5.28E-78 | 271 |
| **SR-VP_0-2_scaffold_141_2510002_86|SR-VP_0-2cm_Jumbo_Phage_48_23|SR-VP_0-2cm** | SR-VP_0-2_scaffold_141_4101478_13|SR-VP_0-2cm_UNK|SR-VP_0-2cm | 44.054 | 370 | 183 | 6 | 132 | 481 | 68 | 433 | 8.22E-78 | 281 |
| **SR-VP_0-2_scaffold_141_2510002_86|SR-VP_0-2cm_Jumbo_Phage_48_23|SR-VP_0-2cm** | PAFVLPS_2018_J26_2_scaffold_3254_1|soil-virus-P10-J26-2018_UNK|soil-virus-P10-J26-2018 | 38.285 | 478 | 263 | 11 | 129 | 586 | 123 | 588 | 1.49E-77 | 278 |
| **SR-VP_0-2_scaffold_141_2510002_86|SR-VP_0-2cm_Jumbo_Phage_48_23|SR-VP_0-2cm** | rifcsphigho2_01_scaffold_331662_1|RifCSPhighO2_01_full_UNK|RIFCSPHIGHO2_01_FULL | 39.788 | 377 | 211 | 6 | 115 | 487 | 48 | 412 | 1.56E-77 | 272 |
| **SR-VP_0-2_scaffold_141_2510002_86|SR-VP_0-2cm_Jumbo_Phage_48_23|SR-VP_0-2cm** | CG_2015-0102_scaffold_90759_1|CG_2015-0102_UNK|CG_2015-0102 | 42.105 | 380 | 203 | 7 | 131 | 503 | 87 | 456 | 3.12E-77 | 273 |
| **SR-VP_0-2_scaffold_141_2510002_86|SR-VP_0-2cm_Jumbo_Phage_48_23|SR-VP_0-2cm** | GD18-4_manure_scaffold_358173_3|GD2018-4_manure_QB3_180703_UNK|GD2018-4_manure_QB3_180703 | 43.421 | 380 | 181 | 8 | 127 | 484 | 107 | 474 | 3.93E-77 | 280 |
| **SR-VP_0-2_scaffold_141_2510002_86|SR-VP_0-2cm_Jumbo_Phage_48_23|SR-VP_0-2cm** | P0_An_pond3_S8_coassembly_k141_1807598_14|P0_An_pond3_S8_coassembly_UNK|E_GD2017-1_P0_An_pond3_S8_Biohub_coassembly | 43.421 | 380 | 181 | 8 | 127 | 484 | 107 | 474 | 3.93E-77 | 280 |
| **SR-VP_0-2_scaffold_141_2510002_86|SR-VP_0-2cm_Jumbo_Phage_48_23|SR-VP_0-2cm** | P0_An_GD2017L_S7_coassembly_k141_1153455_4|P0_An_GD2017L_S7_coassembly_UNK|E_P0_An_GD2017L_S7_coassembly | 43.421 | 380 | 181 | 8 | 127 | 484 | 107 | 474 | 3.93E-77 | 280 |
| **SR-VP_0-2_scaffold_141_2510002_86|SR-VP_0-2cm_Jumbo_Phage_48_23|SR-VP_0-2cm** | P0_An_pond3_S8_170907_scaffold_76835_4|E_GD2017-1_P0_AN_POND3_S8_BIOHUB_170907_UNK|E_GD2017-1_P0_An_pond3_S8_Biohub_170907 | 43.421 | 380 | 181 | 8 | 127 | 484 | 107 | 474 | 3.93E-77 | 280 |
| **SR-VP_0-2_scaffold_141_2510002_86|SR-VP_0-2cm_Jumbo_Phage_48_23|SR-VP_0-2cm** | P0_An_GD2017L_S7_170907_scaffold_1660639_198|E_GD2017-1_P0_An_GD2017L_S7_Biohub_170907_Phage-like_CRISPR_62_55|E_GD2017-1_P0_An_GD2017L_S7_Biohub_170907 | 43.421 | 380 | 181 | 8 | 127 | 484 | 107 | 474 | 3.93E-77 | 280 |
| **SR-VP_0-2_scaffold_141_2510002_86|SR-VP_0-2cm_Jumbo_Phage_48_23|SR-VP_0-2cm** | S_p2_S4_coassembly_k141_3334842_2|S_p2_S4_coassembly_UNK|S_p2_S4_coassembly | 41.240 | 371 | 203 | 5 | 115 | 481 | 251 | 610 | 4.96E-77 | 276 |
| **SR-VP_0-2_scaffold_141_2510002_86|SR-VP_0-2cm_Jumbo_Phage_48_23|SR-VP_0-2cm** | PAFVLPS_2018_scaffold_13917_17|viral-cat_UNK|viral-cat | 38.285 | 478 | 263 | 11 | 129 | 586 | 184 | 649 | 5.66E-77 | 278 |
| **SR-VP_0-2_scaffold_141_2510002_86|SR-VP_0-2cm_Jumbo_Phage_48_23|SR-VP_0-2cm** | CG_2015-17_scaffold_98963_1|CG_2015-17_UNK|CG_2015-17 | 42.105 | 380 | 203 | 7 | 131 | 503 | 17 | 386 | 6E-77 | 273 |
| **SR-VP_0-2_scaffold_141_2510002_86|SR-VP_0-2cm_Jumbo_Phage_48_23|SR-VP_0-2cm** | BML_08182015_8_5m_scaffold_346_59|BML_08182015_8_5m_UNK|BML_08182015_8_5m | 42.051 | 390 | 191 | 10 | 133 | 492 | 27 | 411 | 7.87E-77 | 280 |
| **SR-VP_0-2_scaffold_141_2510002_86|SR-VP_0-2cm_Jumbo_Phage_48_23|SR-VP_0-2cm** | BML_08182015_6_5m_scaffold_778_17|BML_08182015_6_5m_UNK|BML_08182015_6_5m | 42.051 | 390 | 191 | 10 | 133 | 492 | 27 | 411 | 7.87E-77 | 280 |
| **SR-VP_0-2_scaffold_141_2510002_86|SR-VP_0-2cm_Jumbo_Phage_48_23|SR-VP_0-2cm** | BML_08182015_1_5m_scaffold_1132_8|BML_08182015_1_5m_UNK|BML_08182015_1_5m | 42.051 | 390 | 191 | 10 | 133 | 492 | 27 | 411 | 7.87E-77 | 280 |
| **SR-VP_0-2_scaffold_141_2510002_86|SR-VP_0-2cm_Jumbo_Phage_48_23|SR-VP_0-2cm** | Crystal_Geyser_4_8_14_3_um_filter_scaffold_40196_1|CG_4_8_14_3_um_filter_Proteobacteria_50_5__maxbin2.output.069|CG_4_8_14_3_um_filter | 42.778 | 360 | 184 | 7 | 136 | 481 | 2 | 353 | 9.83E-77 | 275 |
| **SR-VP_0-2_scaffold_141_2510002_86|SR-VP_0-2cm_Jumbo_Phage_48_23|SR-VP_0-2cm** | SR-2_scaffold_141_1262958_1|SR-2_Biohub_180515_UNK|SR2-17_Biohub_180515 | 39.788 | 377 | 211 | 6 | 115 | 487 | 55 | 419 | 1.08E-76 | 271 |
| **SR-VP_0-2_scaffold_141_2510002_86|SR-VP_0-2cm_Jumbo_Phage_48_23|SR-VP_0-2cm** | gwc1_scaffold_78_108|GWC1_Phage|GWC1 | 44.986 | 369 | 190 | 8 | 136 | 497 | 92 | 454 | 1.12E-76 | 282 |
| **SR-VP_0-2_scaffold_141_2510002_86|SR-VP_0-2cm_Jumbo_Phage_48_23|SR-VP_0-2cm** | GWB1_scaffold_18_323|GWB1_scaffold_18_complete_phage_genome_31_26|GWB1 | 44.986 | 369 | 190 | 8 | 136 | 497 | 92 | 454 | 1.12E-76 | 282 |
| **SR-VP_0-2_scaffold_141_2510002_86|SR-VP_0-2cm_Jumbo_Phage_48_23|SR-VP_0-2cm** | gwe1_scaffold_2321_7|GWE1_Phage|GWE1 | 38.242 | 421 | 239 | 8 | 132 | 534 | 25 | 442 | 1.13E-76 | 276 |
| **SR-VP_0-2_scaffold_141_2510002_86|SR-VP_0-2cm_Jumbo_Phage_48_23|SR-VP_0-2cm** | L2c2_full_idba_ud_scaffold_5903_5|L2c2_MaxBin2_EukRep_ggKbase_unknown_002|L2c2 | 46.328 | 354 | 168 | 7 | 140 | 481 | 329 | 672 | 1.27E-76 | 287 |
| **SR-VP_0-2_scaffold_141_2510002_86|SR-VP_0-2cm_Jumbo_Phage_48_23|SR-VP_0-2cm** | PAFVLPS_2018_scaffold_618177_1|viral-cat_UNK|viral-cat | 43.360 | 369 | 188 | 9 | 136 | 489 | 64 | 426 | 1.51E-76 | 270 |
| **SR-VP_0-2_scaffold_141_2510002_86|SR-VP_0-2cm_Jumbo_Phage_48_23|SR-VP_0-2cm** | LAC_NA01_scaffold_20246_1|NA01_UNK|NA01 | 42.377 | 387 | 199 | 8 | 137 | 509 | 61 | 437 | 1.55E-76 | 268 |
| **SR-VP_0-2_scaffold_141_2510002_86|SR-VP_0-2cm_Jumbo_Phage_48_23|SR-VP_0-2cm** | CG_2015-01t_scaffold_12134_1|CG_2015-01t_UNK|CG_2015-01t | 43.210 | 405 | 199 | 10 | 113 | 490 | 283 | 683 | 1.63E-76 | 280 |
| **SR-VP_0-2_scaffold_141_2510002_86|SR-VP_0-2cm_Jumbo_Phage_48_23|SR-VP_0-2cm** | k87_5988256_1|zodeltone_water_may_2017_UNK|zodeltone_water_may_2017 | 42.597 | 385 | 201 | 6 | 137 | 509 | 10 | 386 | 3.54E-76 | 266 |
| **SR-VP_0-2_scaffold_141_2510002_86|SR-VP_0-2cm_Jumbo_Phage_48_23|SR-VP_0-2cm** | gwc1_scaffold_5043_5|GWC1 | 41.953 | 379 | 189 | 7 | 137 | 494 | 30 | 398 | 4.14E-76 | 279 |
| **SR-VP_0-2_scaffold_141_2510002_86|SR-VP_0-2cm_Jumbo_Phage_48_23|SR-VP_0-2cm** | GWB1_scaffold_4318_21|GWB1_phage|GWB1 | 41.953 | 379 | 189 | 7 | 137 | 494 | 30 | 398 | 4.14E-76 | 279 |
| **SR-VP_0-2_scaffold_141_2510002_86|SR-VP_0-2cm_Jumbo_Phage_48_23|SR-VP_0-2cm** | DRTY7_scaffold_2738_4|DRTY7_UNK|DRTY7 | 42.377 | 387 | 199 | 8 | 137 | 509 | 31 | 407 | 4.71E-76 | 270 |
| **SR-VP_0-2_scaffold_141_2510002_86|SR-VP_0-2cm_Jumbo_Phage_48_23|SR-VP_0-2cm** | BML_02132018_6_5m_scaffold_680_13|BML_02132018_6_5m_UNK|BML_02132018_6_5m | 40.503 | 358 | 201 | 4 | 128 | 481 | 13 | 362 | 5.43E-76 | 272 |
| **SR-VP_0-2_scaffold_141_2510002_86|SR-VP_0-2cm_Jumbo_Phage_48_23|SR-VP_0-2cm** | P0_An_GD2017L_S7_170907_scaffold_897254_1|E_GD2017-1_P0_An_GD2017L_S7_Biohub_170907_UNK|E_GD2017-1_P0_An_GD2017L_S7_Biohub_170907 | 42.821 | 390 | 195 | 11 | 133 | 507 | 31 | 407 | 5.77E-76 | 267 |
| **SR-VP_0-2_scaffold_141_2510002_86|SR-VP_0-2cm_Jumbo_Phage_48_23|SR-VP_0-2cm** | S35_SO-1_scaffold_340710_1|Genasci_Feb2018_S35_SO-1_UNK|Genasci_Feb2018_S35_SO-1 | 40.599 | 367 | 203 | 5 | 119 | 481 | 1 | 356 | 6.92E-76 | 270 |
| **SR-VP_0-2_scaffold_141_2510002_86|SR-VP_0-2cm_Jumbo_Phage_48_23|SR-VP_0-2cm** | LacPavin_0818_WC40_scaffold_58866_4|LacPavin_0818_WC40_UNK|LacPavin_0818_WC40 | 43.213 | 361 | 192 | 7 | 132 | 485 | 36 | 390 | 8.17E-76 | 271 |
| **SR-VP_0-2_scaffold_141_2510002_86|SR-VP_0-2cm_Jumbo_Phage_48_23|SR-VP_0-2cm** | S28_BME28_42659_1|BM_2017_Strous_8_UNK|BM_2017_Strous_8 | 42.602 | 392 | 195 | 10 | 125 | 497 | 55 | 435 | 8.73E-76 | 267 |
| **SR-VP_0-2_scaffold_141_2510002_86|SR-VP_0-2cm_Jumbo_Phage_48_23|SR-VP_0-2cm** | PAFVLPS_2018_scaffold_141932_3|viral-cat_UNK|viral-cat | 42.659 | 361 | 195 | 6 | 134 | 488 | 349 | 703 | 1E-75 | 281 |
| **SR-VP_0-2_scaffold_141_2510002_86|SR-VP_0-2cm_Jumbo_Phage_48_23|SR-VP_0-2cm** | P0_An_GD2017L_S7_170907_scaffold_2265713_1|E_GD2017-1_P0_An_GD2017L_S7_Biohub_170907_UNK|E_GD2017-1_P0_An_GD2017L_S7_Biohub_170907 | 42.602 | 392 | 195 | 10 | 125 | 497 | 55 | 435 | 1.07E-75 | 266 |
| **SR-VP_0-2_scaffold_141_2510002_86|SR-VP_0-2cm_Jumbo_Phage_48_23|SR-VP_0-2cm** | rifcsphigho2_02_scaffold_18912_4|RIFCSPHIGHO2_02_FULL_Archaea_Woesearchaeota_45_15|RIFCSPHIGHO2_02_FULL | 40.701 | 371 | 205 | 5 | 115 | 481 | 250 | 609 | 1.23E-75 | 279 |
| **SR-VP_0-2_scaffold_141_2510002_86|SR-VP_0-2cm_Jumbo_Phage_48_23|SR-VP_0-2cm** | rifcsplowo2_01_scaffold_2432_2|RIFCSPLOWO2_01_FULL_Archaea_Woesearchaeota_46_24|RIFCSPLOWO2_01_FULL | 40.701 | 371 | 205 | 5 | 115 | 481 | 250 | 609 | 1.23E-75 | 279 |
| **SR-VP_0-2_scaffold_141_2510002_86|SR-VP_0-2cm_Jumbo_Phage_48_23|SR-VP_0-2cm** | rifcsphigho2_01_scaffold_2880_2|RIFCSPHIGHO2_01_FULL_Archaea_Woesearchaeota_44_17|RIFCSPHIGHO2_01_FULL | 40.701 | 371 | 205 | 5 | 115 | 481 | 250 | 609 | 1.23E-75 | 279 |
| **SR-VP_0-2_scaffold_141_2510002_86|SR-VP_0-2cm_Jumbo_Phage_48_23|SR-VP_0-2cm** | gwa2_scaffold_3385_2|GW2011_AR3|GWA2 | 40.701 | 371 | 205 | 5 | 115 | 481 | 250 | 609 | 1.23E-75 | 279 |
| **SR-VP_0-2_scaffold_141_2510002_86|SR-VP_0-2cm_Jumbo_Phage_48_23|SR-VP_0-2cm** | ngawha_2_scaffold_11421_1|NGAWHA_2_UNK|NGAWHA_2 | 41.645 | 389 | 220 | 5 | 133 | 518 | 200 | 584 | 1.47E-75 | 271 |
| **SR-VP_0-2_scaffold_141_2510002_86|SR-VP_0-2cm_Jumbo_Phage_48_23|SR-VP_0-2cm** | ALT_082018_0_1um_scaffold_111343_1|ALT_082018_0_1um_UNK|ALT_082018_0_1um | 42.175 | 377 | 195 | 9 | 128 | 488 | 12 | 381 | 1.97E-75 | 264 |
| **SR-VP_0-2_scaffold_141_2510002_86|SR-VP_0-2cm_Jumbo_Phage_48_23|SR-VP_0-2cm** | JSantini_GMIN_scaffold_34_116|JSantini_GMIN_Biofilm_Phage_38_21|JSantini_GMIN_Biofilm | 42.935 | 368 | 194 | 8 | 130 | 485 | 219 | 582 | 2.47E-75 | 274 |
| **SR-VP_0-2_scaffold_141_2510002_86|SR-VP_0-2cm_Jumbo_Phage_48_23|SR-VP_0-2cm** | Salt_Pond_R1_B_D2_MG_scaffold_54040_1|JGI_Salt_Pond_R1_B_D2_MG_UNK|JGI_Salt_Pond_R1_B_D2_MG | 46.328 | 354 | 176 | 6 | 136 | 481 | 12 | 359 | 3.31E-75 | 270 |
| **SR-VP_0-2_scaffold_141_2510002_85|SR-VP_0-2cm_Jumbo_Phage_48_23|SR-VP_0-2cm** | SRVP18_trench_2_45cm_scaffold_9776_2|SRVP18_trench_2_45cm_UNK|SRVP18_trench_2_45cm | 100.000 | 363 | 0 | 0 | 1 | 363 | 1 | 363 | 0.0 | 743 |
| **SR-VP_0-2_scaffold_141_2510002_85|SR-VP_0-2cm_Jumbo_Phage_48_23|SR-VP_0-2cm** | SR-VP_4-6_scaffold_141_5680703_prodigal-single_434|SR-VP_PHAGE_48_20|SR-VP_4-6cm_Biohub_180515 | 100.000 | 363 | 0 | 0 | 1 | 363 | 1 | 363 | 0.0 | 743 |
| **SR-VP_0-2_scaffold_141_2510002_85|SR-VP_0-2cm_Jumbo_Phage_48_23|SR-VP_0-2cm** | SR-VP_4-6_scaffold_141_5680703_424|SR-VP_4-6cm_Biohub_180515_Phage_48_20|SR-VP_4-6cm_Biohub_180515 | 100.000 | 363 | 0 | 0 | 1 | 363 | 1 | 363 | 0.0 | 743 |
| **SR-VP_0-2_scaffold_141_2510002_85|SR-VP_0-2cm_Jumbo_Phage_48_23|SR-VP_0-2cm** | SR-VP_0-2_scaffold_141_2510002_prodigal-single_87|SR-VP_PHAGE_48_23_A|SR-VP_0-2cm | 100.000 | 363 | 0 | 0 | 1 | 363 | 1 | 363 | 0.0 | 743 |
| **SR-VP_0-2_scaffold_141_2510002_85|SR-VP_0-2cm_Jumbo_Phage_48_23|SR-VP_0-2cm** | SR-VP_0-2_scaffold_141_2510002_85|SR-VP_0-2cm_Jumbo_Phage_48_23|SR-VP_0-2cm | 100.000 | 363 | 0 | 0 | 1 | 363 | 1 | 363 | 0.0 | 743 |
| **SR-VP_0-2_scaffold_141_2510002_85|SR-VP_0-2cm_Jumbo_Phage_48_23|SR-VP_0-2cm** | SR-VP_2-4_scaffold_141_1102992_66|SR-VP_2-4cm_UNK|SR-VP_2-4cm | 48.077 | 364 | 180 | 6 | 1 | 357 | 1 | 362 | 2.15E-109 | 334 |
| **SR-VP_0-2_scaffold_141_2510002_85|SR-VP_0-2cm_Jumbo_Phage_48_23|SR-VP_0-2cm** | SR-VP_0-2_scaffold_141_7296648_244|SR-VP_0-2cm_UNK|SR-VP_0-2cm | 48.077 | 364 | 180 | 6 | 1 | 357 | 1 | 362 | 2.15E-109 | 334 |
| **SR-VP_0-2_scaffold_141_2510002_85|SR-VP_0-2cm_Jumbo_Phage_48_23|SR-VP_0-2cm** | SRVP18_core_1_40-35cm_scaffold_8373_3|SRVP18_core_1_40-35cm_UNK|SRVP18_core_1_40-35cm | 49.591 | 367 | 162 | 6 | 8 | 358 | 6 | 365 | 6.59E-109 | 333 |
| **SR-VP_0-2_scaffold_141_2510002_85|SR-VP_0-2cm_Jumbo_Phage_48_23|SR-VP_0-2cm** | LacPavin_0818_WC40_scaffold_445850_3|LacPavin_0818_WC40_UNK|LacPavin_0818_WC40 | 52.239 | 268 | 119 | 4 | 98 | 359 | 113 | 377 | 6.45E-86 | 275 |
| **SR-VP_0-2_scaffold_141_2510002_85|SR-VP_0-2cm_Jumbo_Phage_48_23|SR-VP_0-2cm** | LacPavin_0818_WC40_scaffold_180876_2|LacPavin_0818_WC40_UNK|LacPavin_0818_WC40 | 48.410 | 283 | 136 | 3 | 80 | 359 | 77 | 352 | 7.65E-85 | 271 |
| **SR-VP_0-2_scaffold_141_2510002_85|SR-VP_0-2cm_Jumbo_Phage_48_23|SR-VP_0-2cm** | LacPavin_0818_WC50_scaffold_667175_3|LacPavin_0818_WC50_UNK|LacPavin_0818_WC50 | 49.117 | 283 | 134 | 3 | 80 | 359 | 213 | 488 | 6.83E-84 | 273 |
| **SR-VP_0-2_scaffold_141_2510002_85|SR-VP_0-2cm_Jumbo_Phage_48_23|SR-VP_0-2cm** | BML_08182015_1_5m_scaffold_24379_2|BML_08182015_1_5m_UNK|BML_08182015_1_5m | 53.469 | 245 | 110 | 2 | 117 | 358 | 62 | 305 | 8.22E-84 | 267 |
| **SR-VP_0-2_scaffold_141_2510002_85|SR-VP_0-2cm_Jumbo_Phage_48_23|SR-VP_0-2cm** | AB_092018_0_1um_scaffold_61801_1|AB_092018_0_1um_UNK|AB_092018_0_1um | 52.790 | 233 | 106 | 2 | 129 | 358 | 3 | 234 | 4.39E-83 | 263 |
| **SR-VP_0-2_scaffold_141_2510002_85|SR-VP_0-2cm_Jumbo_Phage_48_23|SR-VP_0-2cm** | FFC_092018_0_1um_scaffold_2195_1|FFC_092018_0_1um_UNK|FFC_092018_0_1um | 58.036 | 224 | 90 | 2 | 138 | 358 | 603 | 825 | 1.78E-82 | 278 |
| **SR-VP_0-2_scaffold_141_2510002_85|SR-VP_0-2cm_Jumbo_Phage_48_23|SR-VP_0-2cm** | AB_092018_0_1um_scaffold_34262_1|AB_092018_0_1um_UNK|AB_092018_0_1um | 48.410 | 283 | 136 | 3 | 80 | 359 | 214 | 489 | 4.94E-82 | 269 |
| **SR-VP_0-2_scaffold_141_2510002_85|SR-VP_0-2cm_Jumbo_Phage_48_23|SR-VP_0-2cm** | BML_coassembly_scaffold_232968_1|BML_coassembly_UNK|BML_coassembly | 52.653 | 245 | 112 | 2 | 117 | 358 | 88 | 331 | 6.22E-82 | 263 |
| **SR-VP_0-2_scaffold_141_2510002_85|SR-VP_0-2cm_Jumbo_Phage_48_23|SR-VP_0-2cm** | FFC_04162018_0_1um_scaffold_2303_1|FFC_04162018_0_1um_UNK|FFC_04162018_0_1um | 58.036 | 224 | 90 | 2 | 138 | 358 | 856 | 1078 | 9.39E-81 | 278 |
| **SR-VP_0-2_scaffold_141_2510002_85|SR-VP_0-2cm_Jumbo_Phage_48_23|SR-VP_0-2cm** | LacPavin_0419_WC70S_scaffold_198126_1|LacPavin_0419_WC70S_UNK|LacPavin_0419_WC70S | 56.889 | 225 | 93 | 2 | 138 | 359 | 697 | 920 | 2.7E-80 | 274 |
| **SR-VP_0-2_scaffold_141_2510002_85|SR-VP_0-2cm_Jumbo_Phage_48_23|SR-VP_0-2cm** | FFC_07242016_10_scaffold_3_216|FFC_07242016_10_Large_Phage_41_5|FFC_07242016_10 | 58.036 | 224 | 90 | 2 | 138 | 358 | 1226 | 1448 | 5.86E-80 | 278 |
| **SR-VP_0-2_scaffold_141_2510002_85|SR-VP_0-2cm_Jumbo_Phage_48_23|SR-VP_0-2cm** | LacPavin_0818_WC55_scaffold_946946_1|LacPavin_0818_WC55_UNK|LacPavin_0818_WC55 | 56.889 | 225 | 93 | 2 | 138 | 359 | 811 | 1034 | 1.02E-79 | 274 |
| **SR-VP_0-2_scaffold_141_2510002_85|SR-VP_0-2cm_Jumbo_Phage_48_23|SR-VP_0-2cm** | FFC_04162018_0_1um_scaffold_8251_1|FFC_04162018_0_1um_UNK|FFC_04162018_0_1um | 52.361 | 233 | 107 | 2 | 129 | 358 | 171 | 402 | 1.76E-79 | 259 |
| **SR-VP_0-2_scaffold_141_2510002_85|SR-VP_0-2cm_Jumbo_Phage_48_23|SR-VP_0-2cm** | LacPavin_0419_WC53_scaffold_456014_7|LacPavin_0419_WC53_UNK|LacPavin_0419_WC53 | 56.889 | 225 | 93 | 2 | 138 | 359 | 1051 | 1274 | 7.99E-79 | 274 |
| **SR-VP_0-2_scaffold_141_2510002_85|SR-VP_0-2cm_Jumbo_Phage_48_23|SR-VP_0-2cm** | LacPavin_0419_WC53_scaffold_36678_62|LacPavin_0419_WC53_UNK|LacPavin_0419_WC53 | 53.138 | 239 | 108 | 2 | 123 | 358 | 710 | 947 | 1.26E-78 | 270 |
| **SR-VP_0-2_scaffold_141_2510002_85|SR-VP_0-2cm_Jumbo_Phage_48_23|SR-VP_0-2cm** | LacPavin_0419_WC70S_scaffold_882983_4|LacPavin_0419_WC70S_UNK|LacPavin_0419_WC70S | 52.720 | 239 | 109 | 2 | 123 | 358 | 710 | 947 | 3.66E-78 | 269 |
| **SR-VP_0-2_scaffold_141_2510002_85|SR-VP_0-2cm_Jumbo_Phage_48_23|SR-VP_0-2cm** | scaff_0001503963_2|Unk_small|chonglepansoilsip | 55.319 | 235 | 101 | 2 | 123 | 354 | 154 | 387 | 7.01E-78 | 256 |
| **SR-VP_0-2_scaffold_141_2510002_85|SR-VP_0-2cm_Jumbo_Phage_48_23|SR-VP_0-2cm** | LacPavin_0419_WC53_scaffold_75329_16|LacPavin_0419_WC53_Potentially_Complete_Phage_42_65|LacPavin_0419_WC53 | 53.219 | 233 | 105 | 2 | 129 | 358 | 1675 | 1906 | 5.51E-76 | 267 |
| **SR-VP_0-2_scaffold_141_2510002_85|SR-VP_0-2cm_Jumbo_Phage_48_23|SR-VP_0-2cm** | LacPavin_0419_WC53_scaffold_118977_10|LacPavin_0419_WC53_UNK|LacPavin_0419_WC53 | 50.598 | 251 | 116 | 2 | 111 | 358 | 1078 | 1323 | 1.75E-75 | 265 |
| **SR-VP_0-2_scaffold_141_2510002_85|SR-VP_0-2cm_Jumbo_Phage_48_23|SR-VP_0-2cm** | BC_09192017_0_5m_scaffold_8132_4|BC_09192017_0_5m_UNK|BC_09192017_0_5m | 53.165 | 237 | 107 | 2 | 129 | 362 | 879 | 1114 | 4.65E-75 | 262 |
| **SR-VP_0-2_scaffold_141_2510002_85|SR-VP_0-2cm_Jumbo_Phage_48_23|SR-VP_0-2cm** | PAFVLPS_2018_scaffold_342543_3|viral-cat_UNK|viral-cat | 47.600 | 250 | 122 | 3 | 119 | 360 | 8 | 256 | 2.4E-73 | 239 |
| **SR-VP_0-2_scaffold_141_2510002_85|SR-VP_0-2cm_Jumbo_Phage_48_23|SR-VP_0-2cm** | LacPavin_0818_WC45_scaffold_247185_2|LacPavin_0818_WC45_UNK|LacPavin_0818_WC45 | 50.628 | 239 | 111 | 2 | 80 | 318 | 68 | 299 | 5.61E-73 | 239 |
| **SR-VP_0-2_scaffold_141_2510002_85|SR-VP_0-2cm_Jumbo_Phage_48_23|SR-VP_0-2cm** | AB_072018_0_1um_scaffold_3109_1|AB_072018_0_1um_UNK|AB_072018_0_1um | 59.239 | 184 | 74 | 1 | 138 | 321 | 372 | 554 | 8.45E-69 | 236 |
| **SR-VP_0-2_scaffold_141_2510002_85|SR-VP_0-2cm_Jumbo_Phage_48_23|SR-VP_0-2cm** | ALT_03122018_0_1um_scaffold_2_37|ALT_03122018_0_1um_partial_phage_44_12|ALT_03122018_0_1um | 46.586 | 249 | 124 | 3 | 110 | 353 | 99 | 343 | 9.64E-69 | 230 |
| **SR-VP_0-2_scaffold_141_2510002_85|SR-VP_0-2cm_Jumbo_Phage_48_23|SR-VP_0-2cm** | BML_coassembly_scaffold_42454_3|BML_coassembly_UNK|BML_coassembly | 51.818 | 220 | 102 | 2 | 142 | 358 | 156 | 374 | 4.45E-67 | 226 |
| **SR-VP_0-2_scaffold_141_2510002_85|SR-VP_0-2cm_Jumbo_Phage_48_23|SR-VP_0-2cm** | S27_BME27_611489_14|BM_2017_Strous_6_UNK|BM_2017_Strous_6 | 49.275 | 276 | 123 | 7 | 87 | 353 | 96 | 363 | 4.67E-65 | 221 |
| **SR-VP_0-2_scaffold_141_2510002_85|SR-VP_0-2cm_Jumbo_Phage_48_23|SR-VP_0-2cm** | S19_GE19_scaffold_115722_12|GD2017-2_strous-5-pellet_S19_GE19_Biohub_170515_UNK|E_GD2017-2_strous-5-pellet_S19_GE19_Biohub_180515 | 49.275 | 276 | 123 | 7 | 87 | 353 | 96 | 363 | 4.67E-65 | 221 |
| **SR-VP_0-2_scaffold_141_2510002_85|SR-VP_0-2cm_Jumbo_Phage_48_23|SR-VP_0-2cm** | S16_GE16_scaffold_191292_10|E_GD2017-2_urea-3_S16_GE16_Biohub_170515_UNK|E_GD2017-2_urea-3_S16_GE16_Biohub_180515 | 49.275 | 276 | 123 | 7 | 87 | 353 | 96 | 363 | 4.67E-65 | 221 |
| **SR-VP_0-2_scaffold_141_2510002_85|SR-VP_0-2cm_Jumbo_Phage_48_23|SR-VP_0-2cm** | scaffold_104756_14|2019_SCN_bioreactor_t3_biofilm_UNK|2019_SCN_bioreactor_t3_biofilm | 48.551 | 276 | 125 | 7 | 87 | 353 | 96 | 363 | 1.64E-64 | 219 |
| **SR-VP_0-2_scaffold_141_2510002_85|SR-VP_0-2cm_Jumbo_Phage_48_23|SR-VP_0-2cm** | scaffold_1443_44|2019_SCN_bioreactor_t2_plantonic_UNK|2019_SCN_bioreactor_t2_planktonic | 48.551 | 276 | 125 | 7 | 87 | 353 | 96 | 363 | 1.64E-64 | 219 |
| **SR-VP_0-2_scaffold_141_2510002_85|SR-VP_0-2cm_Jumbo_Phage_48_23|SR-VP_0-2cm** | scaffold_125037_14|2019_SCN_bioreactor_t2_biofilm_UNK|2019_SCN_bioreactor_t2_biofilm | 48.551 | 276 | 125 | 7 | 87 | 353 | 96 | 363 | 1.64E-64 | 219 |
| **SR-VP_0-2_scaffold_141_2510002_85|SR-VP_0-2cm_Jumbo_Phage_48_23|SR-VP_0-2cm** | scaffold_124576_14|2019_SCN_bioreactor_t1_biofilm_UNK|2019_SCN_bioreactor_t1_biofilm | 48.551 | 276 | 125 | 7 | 87 | 353 | 96 | 363 | 1.64E-64 | 219 |
| **SR-VP_0-2_scaffold_141_2510002_85|SR-VP_0-2cm_Jumbo_Phage_48_23|SR-VP_0-2cm** | SCN_reactora_scaffold_60411_14|SCN_bioreactor_inoc_reactora_UNK|2019_SCN_bioreactor_inoc_reactora | 48.551 | 276 | 125 | 7 | 87 | 353 | 96 | 363 | 1.64E-64 | 219 |
| **SR-VP_0-2_scaffold_141_2510002_85|SR-VP_0-2cm_Jumbo_Phage_48_23|SR-VP_0-2cm** | scnpilot_solids2_trim150_scaffold_666_18|SCNPILOT_SOLID2_TRIM150_UNK|SCNPILOT_SOLID2_TRIM150 | 48.551 | 276 | 125 | 7 | 87 | 353 | 96 | 363 | 1.79E-64 | 219 |
| **SR-VP_0-2_scaffold_141_2510002_85|SR-VP_0-2cm_Jumbo_Phage_48_23|SR-VP_0-2cm** | SCNpilot_solid_2_scaffold_700_14|SCNpilot_solid_2_UNK|SCNPILOT_SOLID_2 | 48.551 | 276 | 125 | 7 | 87 | 353 | 96 | 363 | 1.79E-64 | 219 |
| **SR-VP_0-2_scaffold_141_2510002_85|SR-VP_0-2cm_Jumbo_Phage_48_23|SR-VP_0-2cm** | scnpilot_expt_750_p_scaffold_1076_14|scnpilot_dereplicated_Virus_unknown_3|SCNPILOT_EXPT_750_P | 48.551 | 276 | 125 | 7 | 87 | 353 | 96 | 363 | 1.79E-64 | 219 |
| **SR-VP_0-2_scaffold_141_2510002_85|SR-VP_0-2cm_Jumbo_Phage_48_23|SR-VP_0-2cm** | scnpilot_cont_500_p_scaffold_8611_4|SCNpilot_cont_500_p_UNK|SCNPILOT_CONT_500_P | 48.551 | 276 | 125 | 7 | 87 | 353 | 96 | 363 | 1.79E-64 | 219 |
| **SR-VP_0-2_scaffold_141_2510002_85|SR-VP_0-2cm_Jumbo_Phage_48_23|SR-VP_0-2cm** | SRVP18_core_1_40-35cm_scaffold_41688_1|SRVP18_core_1_40-35cm_UNK|SRVP18_core_1_40-35cm | 100.000 | 99 | 0 | 0 | 265 | 363 | 1 | 99 | 5.44E-64 | 209 |
| **SR-VP_0-2_scaffold_141_2510002_85|SR-VP_0-2cm_Jumbo_Phage_48_23|SR-VP_0-2cm** | SCNpilot_expt_1000_bf_scaffold_1100_11|SCNPILOT_EXPT_750_P_Eukaryotic_virus_38_17|SCNPILOT_EXPT_1000_BF | 48.188 | 276 | 126 | 7 | 87 | 353 | 96 | 363 | 6.49E-64 | 218 |
| **SR-VP_0-2_scaffold_141_2510002_85|SR-VP_0-2cm_Jumbo_Phage_48_23|SR-VP_0-2cm** | scaffold_164011_58|J1_AP2_S144_UNK|J1_AP2_S144 | 51.185 | 211 | 90 | 2 | 163 | 360 | 460 | 670 | 7.15E-63 | 223 |
| **SR-VP_0-2_scaffold_141_2510002_85|SR-VP_0-2cm_Jumbo_Phage_48_23|SR-VP_0-2cm** | LacPavin_0419_WC53_scaffold_629_4|LacPavin_0419_WC53_UNK|LacPavin_0419_WC53 | 46.614 | 251 | 129 | 3 | 112 | 358 | 48 | 297 | 1.09E-62 | 213 |
| **SR-VP_0-2_scaffold_141_2510002_85|SR-VP_0-2cm_Jumbo_Phage_48_23|SR-VP_0-2cm** | ALT_03122018_0_1um_scaffold_20765_3|ALT_03122018_0_1um_UNK|ALT_03122018_0_1um | 44.565 | 276 | 135 | 5 | 97 | 358 | 1 | 272 | 1.1E-61 | 209 |
| **SR-VP_0-2_scaffold_141_2510002_85|SR-VP_0-2cm_Jumbo_Phage_48_23|SR-VP_0-2cm** | H11_Tanzania_scaffold_1245_3|H11_Tanzania_UNK|H11_Tanzania | 48.544 | 206 | 94 | 3 | 163 | 356 | 315 | 520 | 1.67E-61 | 216 |
| **SR-VP_0-2_scaffold_141_2510002_85|SR-VP_0-2cm_Jumbo_Phage_48_23|SR-VP_0-2cm** | H10_Tanzania_scaffold_1287_4|H10_Tanzania_UNK|H10_Tanzania | 48.544 | 206 | 94 | 3 | 163 | 356 | 315 | 520 | 1.67E-61 | 216 |
| **SR-VP_0-2_scaffold_141_2510002_85|SR-VP_0-2cm_Jumbo_Phage_48_23|SR-VP_0-2cm** | pig_ID_2001_F74_scaffold_82830_1|pig_ID_2001_F74_UNK|pig_ID_2001_F74 | 46.930 | 228 | 101 | 3 | 139 | 354 | 103 | 322 | 2.55E-61 | 210 |
| **SR-VP_0-2_scaffold_141_2510002_85|SR-VP_0-2cm_Jumbo_Phage_48_23|SR-VP_0-2cm** | AB_072018_0_1um_scaffold_3643_6|AB_072018_0_1um_UNK|AB_072018_0_1um | 50.254 | 197 | 94 | 2 | 164 | 357 | 22 | 217 | 3.2E-61 | 206 |
| **SR-VP_0-2_scaffold_141_2510002_85|SR-VP_0-2cm_Jumbo_Phage_48_23|SR-VP_0-2cm** | AB_092018_0_1um_scaffold_99802_1|AB_092018_0_1um_UNK|AB_092018_0_1um | 54.455 | 202 | 88 | 2 | 142 | 340 | 126 | 326 | 5.29E-61 | 210 |
| **SR-VP_0-2_scaffold_141_2510002_85|SR-VP_0-2cm_Jumbo_Phage_48_23|SR-VP_0-2cm** | AB_082018_0_1um_scaffold_29987_1|AB_082018_0_1um_UNK|AB_082018_0_1um | 54.455 | 202 | 88 | 2 | 142 | 340 | 126 | 326 | 1.11E-60 | 209 |
| **SR-VP_0-2_scaffold_141_2510002_85|SR-VP_0-2cm_Jumbo_Phage_48_23|SR-VP_0-2cm** | LacPavin_0718_WC55_scaffold_5535_1|LacPavin_0718_WC55_UNK|LacPavin_0718_WC55 | 46.544 | 217 | 111 | 2 | 142 | 353 | 34 | 250 | 1.75E-60 | 206 |
| **SR-VP_0-2_scaffold_141_2510002_85|SR-VP_0-2cm_Jumbo_Phage_48_23|SR-VP_0-2cm** | pig_ID_1851_F40_A2_scaffold_145969_1|pig_ID_1851_F40_A2_UNK|pig_ID_1851_F40_A2 | 49.510 | 204 | 91 | 2 | 163 | 354 | 68 | 271 | 4.45E-60 | 205 |
| **SR-VP_0-2_scaffold_141_2510002_85|SR-VP_0-2cm_Jumbo_Phage_48_23|SR-VP_0-2cm** | pig_ID_1851_F40_A1_scaffold_182866_1|pig_ID_1851_F40_A1_UNK|pig_ID_1851_F40_A1 | 47.664 | 214 | 100 | 2 | 153 | 354 | 100 | 313 | 7.76E-60 | 206 |
| **SR-VP_0-2_scaffold_141_2510002_85|SR-VP_0-2cm_Jumbo_Phage_48_23|SR-VP_0-2cm** | LacPavin_0419_WC70S_scaffold_672646_6|LacPavin_0419_WC70S_UNK|LacPavin_0419_WC70S | 55.152 | 165 | 71 | 1 | 197 | 358 | 1 | 165 | 8.8E-60 | 201 |
| **SR-VP_0-2_scaffold_141_2510002_85|SR-VP_0-2cm_Jumbo_Phage_48_23|SR-VP_0-2cm** | RHP_09252018_0_1um_scaffold_8006_6|RHP_09252018_0_1um_UNK|RHP_09252018_0_1um | 50.254 | 197 | 94 | 2 | 164 | 357 | 192 | 387 | 9.93E-60 | 208 |
| **SR-VP_0-2_scaffold_141_2510002_85|SR-VP_0-2cm_Jumbo_Phage_48_23|SR-VP_0-2cm** | LacPavin_0818_WC50_scaffold_348136_2|LacPavin_0818_WC50_UNK|LacPavin_0818_WC50 | 46.502 | 243 | 125 | 3 | 112 | 350 | 5 | 246 | 1.68E-59 | 204 |
| **SR-VP_0-2_scaffold_141_2510002_85|SR-VP_0-2cm_Jumbo_Phage_48_23|SR-VP_0-2cm** | FFC_07242016_10_scaffold_2297_2|FFC_07242016_10_UNK|FFC_07242016_10 | 50.254 | 197 | 94 | 2 | 164 | 357 | 178 | 373 | 1.72E-59 | 207 |
| **SR-VP_0-2_scaffold_141_2510002_85|SR-VP_0-2cm_Jumbo_Phage_48_23|SR-VP_0-2cm** | pig_ID_1851_F40_2_B2_scaffold_116853_1|pig_ID_1851_F40_2_B2_UNK|pig_ID_1851_F40_2_B2 | 47.664 | 214 | 100 | 2 | 153 | 354 | 154 | 367 | 3.55E-59 | 206 |
| **SR-VP_0-2_scaffold_141_2510002_85|SR-VP_0-2cm_Jumbo_Phage_48_23|SR-VP_0-2cm** | LacPavin_0818_WC40_scaffold_1261844_6|LacPavin_0818_WC40_UNK|LacPavin_0818_WC40 | 51.244 | 201 | 92 | 2 | 164 | 358 | 59 | 259 | 2.55E-58 | 200 |
| **SR-VP_0-2_scaffold_141_2510002_85|SR-VP_0-2cm_Jumbo_Phage_48_23|SR-VP_0-2cm** | pig_ID_3687_F49_scaffold_148723_2|pig_ID_3687_F49_UNK|pig_ID_3687_F49 | 48.768 | 203 | 92 | 2 | 164 | 354 | 155 | 357 | 3.49E-58 | 203 |
| **SR-VP_0-2_scaffold_141_2510002_85|SR-VP_0-2cm_Jumbo_Phage_48_23|SR-VP_0-2cm** | FFC_092018_0_1um_scaffold_13629_3|FFC_092018_0_1um_UNK|FFC_092018_0_1um | 54.268 | 164 | 72 | 1 | 198 | 358 | 2 | 165 | 1.19E-57 | 195 |
| **SR-VP_0-2_scaffold_141_2510002_85|SR-VP_0-2cm_Jumbo_Phage_48_23|SR-VP_0-2cm** | Salt_Pond_R1_C_D1_MG_scaffold_14324_3|JGI_Salt_Pond_R1_C_D1_MG_UNK|JGI_Salt_Pond_R1_C_D1_MG | 46.222 | 225 | 110 | 2 | 136 | 353 | 197 | 417 | 3.28E-57 | 202 |
| **SR-VP_0-2_scaffold_141_2510002_85|SR-VP_0-2cm_Jumbo_Phage_48_23|SR-VP_0-2cm** | SRR1747063_scaffold_4_9|M17_unknown_43_25|M17 | 48.768 | 203 | 92 | 2 | 164 | 354 | 313 | 515 | 1.53E-56 | 203 |
| **SR-VP_0-2_scaffold_141_2510002_85|SR-VP_0-2cm_Jumbo_Phage_48_23|SR-VP_0-2cm** | SRR1747056_scaffold_29_83|F25_Potentially_Complete_Phage_29_42|F25 | 48.768 | 203 | 92 | 2 | 164 | 354 | 313 | 515 | 1.53E-56 | 203 |
| **SR-VP_0-2_scaffold_141_2510002_85|SR-VP_0-2cm_Jumbo_Phage_48_23|SR-VP_0-2cm** | SRR1747046_scaffold_7_168|F16_UNK|F16 | 48.768 | 203 | 92 | 2 | 164 | 354 | 313 | 515 | 1.53E-56 | 203 |
| **SR-VP_0-2_scaffold_141_2510002_85|SR-VP_0-2cm_Jumbo_Phage_48_23|SR-VP_0-2cm** | pig_ID_934_F48_scaffold_129992_1|pig_ID_934_F48_UNK|pig_ID_934_F48 | 50.000 | 192 | 94 | 1 | 153 | 342 | 292 | 483 | 1.69E-56 | 202 |
| **SR-VP_0-2_scaffold_141_2510002_85|SR-VP_0-2cm_Jumbo_Phage_48_23|SR-VP_0-2cm** | RHP_09252018_0_1um_scaffold_11910_8|RHP_09252018_0_1um_UNK|RHP_09252018_0_1um | 38.851 | 296 | 159 | 5 | 84 | 358 | 282 | 576 | 4.91E-56 | 203 |
| **SR-VP_0-2_scaffold_141_2510002_85|SR-VP_0-2cm_Jumbo_Phage_48_23|SR-VP_0-2cm** | LacPavin_0818_WC40_scaffold_305333_3|LacPavin_0818_WC40_UNK|LacPavin_0818_WC40 | 44.770 | 239 | 94 | 3 | 123 | 358 | 710 | 913 | 5.7E-56 | 207 |
| **SR-VP_0-2_scaffold_141_2510002_85|SR-VP_0-2cm_Jumbo_Phage_48_23|SR-VP_0-2cm** | LacPavin_0419_WC53_scaffold_559877_4|LacPavin_0419_WC53_UNK|LacPavin_0419_WC53 | 46.083 | 217 | 112 | 1 | 142 | 353 | 621 | 837 | 7.37E-56 | 207 |
| **SR-VP_0-2_scaffold_141_2510002_85|SR-VP_0-2cm_Jumbo_Phage_48_23|SR-VP_0-2cm** | LacPavin_0818_WC55_scaffold_375134_8|LacPavin_0818_WC55_UNK|LacPavin_0818_WC55 | 46.083 | 217 | 112 | 1 | 142 | 353 | 621 | 837 | 7.37E-56 | 207 |
| **SR-VP_0-2_scaffold_141_2510002_85|SR-VP_0-2cm_Jumbo_Phage_48_23|SR-VP_0-2cm** | LacPavin_0818_WC50_scaffold_169986_4|LacPavin_0818_WC50_UNK|LacPavin_0818_WC50 | 46.083 | 217 | 112 | 1 | 142 | 353 | 621 | 837 | 7.37E-56 | 207 |
| **SR-VP_0-2_scaffold_141_2510002_85|SR-VP_0-2cm_Jumbo_Phage_48_23|SR-VP_0-2cm** | LacPavin_0818_WC45_scaffold_141060_83|LacPavin_0818_WC45_UNK|LacPavin_0818_WC45 | 46.083 | 217 | 112 | 1 | 142 | 353 | 621 | 837 | 7.37E-56 | 207 |
| **SR-VP_0-2_scaffold_141_2510002_85|SR-VP_0-2cm_Jumbo_Phage_48_23|SR-VP_0-2cm** | LacPavin_0818_WC40_scaffold_259024_14|LacPavin_0818_WC40_UNK|LacPavin_0818_WC40 | 46.083 | 217 | 112 | 1 | 142 | 353 | 621 | 837 | 7.37E-56 | 207 |
| **SR-VP_0-2_scaffold_141_2510002_85|SR-VP_0-2cm_Jumbo_Phage_48_23|SR-VP_0-2cm** | LacPavin_0718_WC40_scaffold_17_82|LacPavin_0718_WC40_UNK|LacPavin_0718_WC40 | 46.083 | 217 | 112 | 1 | 142 | 353 | 621 | 837 | 7.37E-56 | 207 |
| **SR-VP_0-2_scaffold_141_2510002_85|SR-VP_0-2cm_Jumbo_Phage_48_23|SR-VP_0-2cm** | FFC_07242016_10_scaffold_12139_1|FFC_07242016_10_UNK|FFC_07242016_10 | 44.872 | 234 | 119 | 5 | 131 | 358 | 161 | 390 | 5.44E-55 | 196 |
| **SR-VP_0-2_scaffold_141_2510002_85|SR-VP_0-2cm_Jumbo_Phage_48_23|SR-VP_0-2cm** | pig_ID_2051_F75_scaffold_81142_3|pig_ID_2051_F75_UNK|pig_ID_2051_F75 | 55.280 | 161 | 69 | 2 | 182 | 339 | 10 | 170 | 1.18E-54 | 189 |
| **SR-VP_0-2_scaffold_141_2510002_85|SR-VP_0-2cm_Jumbo_Phage_48_23|SR-VP_0-2cm** | pig_ID_934_F48_scaffold_128145_1|pig_ID_934_F48_UNK|pig_ID_934_F48 | 51.309 | 191 | 78 | 3 | 164 | 339 | 241 | 431 | 1.46E-54 | 196 |
| **SR-VP_0-2_scaffold_141_2510002_85|SR-VP_0-2cm_Jumbo_Phage_48_23|SR-VP_0-2cm** | pig_ID_1851_F40_2_B1_scaffold_274581_1|pig_ID_1851_F40_2_B1_UNK|pig_ID_1851_F40_2_B1 | 53.333 | 165 | 77 | 0 | 153 | 317 | 194 | 358 | 1.07E-53 | 192 |
| **SR-VP_0-2_scaffold_141_2510002_85|SR-VP_0-2cm_Jumbo_Phage_48_23|SR-VP_0-2cm** | AB_092018_0_1um_scaffold_50823_4|AB_092018_0_1um_UNK|AB_092018_0_1um | 51.087 | 184 | 84 | 2 | 181 | 358 | 2 | 185 | 1.12E-53 | 186 |
| **SR-VP_0-2_scaffold_141_2510002_85|SR-VP_0-2cm_Jumbo_Phage_48_23|SR-VP_0-2cm** | pig_ID_2856_F42_A2_scaffold_92574_1|pig_ID_2856_F42_A2_UNK|pig_ID_2856_F42_A2 | 53.012 | 166 | 78 | 0 | 153 | 318 | 218 | 383 | 5.56E-53 | 190 |
| **SR-VP_0-2_scaffold_141_2510002_85|SR-VP_0-2cm_Jumbo_Phage_48_23|SR-VP_0-2cm** | H10_Tanzania_scaffold_52764_1|H10_Tanzania_UNK|H10_Tanzania | 54.321 | 162 | 71 | 1 | 181 | 339 | 201 | 362 | 8.01E-53 | 190 |
| **SR-VP_0-2_scaffold_141_2510002_85|SR-VP_0-2cm_Jumbo_Phage_48_23|SR-VP_0-2cm** | BML_09192017_9_75m_scaffold_49016_2|BML_09192017_9_75m_UNK|BML_09192017_9_75m | 43.421 | 228 | 119 | 4 | 137 | 355 | 103 | 329 | 2.67E-52 | 187 |
| **SR-VP_0-2_scaffold_141_2510002_85|SR-VP_0-2cm_Jumbo_Phage_48_23|SR-VP_0-2cm** | LacPavin_0419_WC70S_scaffold_680580_1|LacPavin_0419_WC70S_UNK|LacPavin_0419_WC70S | 40.647 | 278 | 140 | 10 | 82 | 339 | 73 | 345 | 6.13E-51 | 184 |
| **SR-VP_0-2_scaffold_141_2510002_85|SR-VP_0-2cm_Jumbo_Phage_48_23|SR-VP_0-2cm** | LacPavin_0419_WC53_scaffold_456735_1|LacPavin_0419_WC53_UNK|LacPavin_0419_WC53 | 47.150 | 193 | 96 | 3 | 164 | 351 | 237 | 428 | 1.64E-50 | 185 |
| **SR-VP_0-2_scaffold_141_2510002_85|SR-VP_0-2cm_Jumbo_Phage_48_23|SR-VP_0-2cm** | js4906-20-4_S3_scaffold_2103_5|js4906-20-4_S3_UNK|js4906-20-4_S3 | 44.221 | 199 | 104 | 3 | 163 | 354 | 351 | 549 | 1.08E-49 | 186 |
| **SR-VP_0-2_scaffold_141_2510002_85|SR-VP_0-2cm_Jumbo_Phage_48_23|SR-VP_0-2cm** | LacPavin_0818_WC50_scaffold_619121_3|LacPavin_0818_WC50_UNK|LacPavin_0818_WC50 | 46.766 | 201 | 100 | 4 | 164 | 358 | 452 | 651 | 2.74E-49 | 186 |
| **SR-VP_0-2_scaffold_141_2510002_85|SR-VP_0-2cm_Jumbo_Phage_48_23|SR-VP_0-2cm** | LacPavin_0818_WC45_scaffold_238187_1|LacPavin_0818_WC45_UNK|LacPavin_0818_WC45 | 56.522 | 138 | 57 | 1 | 228 | 362 | 2 | 139 | 6.99E-49 | 172 |
| **SR-VP_0-2_scaffold_141_2510002_85|SR-VP_0-2cm_Jumbo_Phage_48_23|SR-VP_0-2cm** | LacPavin_0818_WC40_scaffold_47343_3|LacPavin_0818_WC40_UNK|LacPavin_0818_WC40 | 40.000 | 280 | 153 | 6 | 86 | 354 | 742 | 1017 | 8.21E-49 | 188 |
| **SR-VP_0-2_scaffold_141_2510002_85|SR-VP_0-2cm_Jumbo_Phage_48_23|SR-VP_0-2cm** | pig_ID_1851_F40_2_scaffold_101562_1|pig_ID_1851_F40_2_UNK|pig_ID_1851_F40_2 | 54.487 | 156 | 71 | 0 | 153 | 308 | 362 | 517 | 8.43E-49 | 182 |
| **SR-VP_0-2_scaffold_141_2510002_85|SR-VP_0-2cm_Jumbo_Phage_48_23|SR-VP_0-2cm** | aot2015-NO13_SRR1761687_USA_scaffold_37443_2|aot2015-NO13_SRR1761687_USA_UNK|aot2015-NO13_SRR1761687_USA | 40.000 | 220 | 121 | 4 | 139 | 348 | 58 | 276 | 1.23E-47 | 174 |
| **SR-VP_0-2_scaffold_141_2510002_85|SR-VP_0-2cm_Jumbo_Phage_48_23|SR-VP_0-2cm** | LacPavin_0419_WC53_scaffold_142857_1|LacPavin_0419_WC53_UNK|LacPavin_0419_WC53 | 55.072 | 138 | 59 | 1 | 228 | 362 | 3 | 140 | 3.11E-47 | 167 |
| **SR-VP_0-2_scaffold_141_2510002_85|SR-VP_0-2cm_Jumbo_Phage_48_23|SR-VP_0-2cm** | pig_ID_3784_F96_scaffold_132457_1|pig_ID_3784_F96_UNK|pig_ID_3784_F96 | 53.416 | 161 | 63 | 1 | 164 | 312 | 306 | 466 | 3.19E-47 | 177 |
| **SR-VP_0-2_scaffold_141_2510002_85|SR-VP_0-2cm_Jumbo_Phage_48_23|SR-VP_0-2cm** | LacPavin_0818_WC45_scaffold_1085480_1|LacPavin_0818_WC45_UNK|LacPavin_0818_WC45 | 38.351 | 279 | 146 | 9 | 82 | 339 | 63 | 336 | 6.91E-47 | 174 |
| **SR-VP_0-2_scaffold_141_2510002_85|SR-VP_0-2cm_Jumbo_Phage_48_23|SR-VP_0-2cm** | pig_ID_3687_F49_scaffold_143529_2|pig_ID_3687_F49_UNK|pig_ID_3687_F49 | 46.411 | 209 | 94 | 3 | 163 | 356 | 38 | 243 | 7.7E-47 | 170 |
| **SR-VP_0-2_scaffold_141_2510002_84|SR-VP_0-2cm_Jumbo_Phage_48_23|SR-VP_0-2cm** | SR-VP_4-6_scaffold_141_5680703_prodigal-single_433|SR-VP_PHAGE_48_20|SR-VP_4-6cm_Biohub_180515 | 100.000 | 352 | 0 | 0 | 1 | 352 | 1 | 352 | 0.0 | 735 |
| **SR-VP_0-2_scaffold_141_2510002_84|SR-VP_0-2cm_Jumbo_Phage_48_23|SR-VP_0-2cm** | SR-VP_4-6_scaffold_141_5680703_423|SR-VP_4-6cm_Biohub_180515_Phage_48_20|SR-VP_4-6cm_Biohub_180515 | 100.000 | 352 | 0 | 0 | 1 | 352 | 1 | 352 | 0.0 | 735 |
| **SR-VP_0-2_scaffold_141_2510002_84|SR-VP_0-2cm_Jumbo_Phage_48_23|SR-VP_0-2cm** | SR-VP_0-2_scaffold_141_2510002_prodigal-single_86|SR-VP_PHAGE_48_23_A|SR-VP_0-2cm | 100.000 | 352 | 0 | 0 | 1 | 352 | 1 | 352 | 0.0 | 735 |
| **SR-VP_0-2_scaffold_141_2510002_84|SR-VP_0-2cm_Jumbo_Phage_48_23|SR-VP_0-2cm** | SR-VP_0-2_scaffold_141_2510002_84|SR-VP_0-2cm_Jumbo_Phage_48_23|SR-VP_0-2cm | 100.000 | 352 | 0 | 0 | 1 | 352 | 1 | 352 | 0.0 | 735 |
| **SR-VP_0-2_scaffold_141_2510002_84|SR-VP_0-2cm_Jumbo_Phage_48_23|SR-VP_0-2cm** | SRVP18_trench_2_45cm_scaffold_9776_1|SRVP18_trench_2_45cm_UNK|SRVP18_trench_2_45cm | 100.000 | 228 | 0 | 0 | 1 | 228 | 1 | 228 | 4.99E-169 | 480 |
| **SR-VP_0-2_scaffold_141_2510002_84|SR-VP_0-2cm_Jumbo_Phage_48_23|SR-VP_0-2cm** | scaff_0000061948_15|chonglepansoilsip_UNK|chonglepansoilsip | 55.794 | 233 | 94 | 4 | 129 | 352 | 12 | 244 | 3.71E-83 | 263 |
| **SR-VP_0-2_scaffold_141_2510002_83|SR-VP_0-2cm_Jumbo_Phage_48_23|SR-VP_0-2cm** | SR-VP_4-6_scaffold_141_5680703_prodigal-single_432|SR-VP_PHAGE_48_20|SR-VP_4-6cm_Biohub_180515 | 100.000 | 289 | 0 | 0 | 1 | 289 | 1 | 289 | 0.0 | 602 |
| **SR-VP_0-2_scaffold_141_2510002_83|SR-VP_0-2cm_Jumbo_Phage_48_23|SR-VP_0-2cm** | SR-VP_4-6_scaffold_141_5680703_422|SR-VP_4-6cm_Biohub_180515_Phage_48_20|SR-VP_4-6cm_Biohub_180515 | 100.000 | 289 | 0 | 0 | 1 | 289 | 1 | 289 | 0.0 | 602 |
| **SR-VP_0-2_scaffold_141_2510002_83|SR-VP_0-2cm_Jumbo_Phage_48_23|SR-VP_0-2cm** | SR-VP_0-2_scaffold_141_2510002_prodigal-single_85|SR-VP_PHAGE_48_23_A|SR-VP_0-2cm | 100.000 | 289 | 0 | 0 | 1 | 289 | 1 | 289 | 0.0 | 602 |
| **SR-VP_0-2_scaffold_141_2510002_83|SR-VP_0-2cm_Jumbo_Phage_48_23|SR-VP_0-2cm** | SR-VP_0-2_scaffold_141_2510002_83|SR-VP_0-2cm_Jumbo_Phage_48_23|SR-VP_0-2cm | 100.000 | 289 | 0 | 0 | 1 | 289 | 1 | 289 | 0.0 | 602 |
| **SR-VP_0-2_scaffold_141_2510002_83|SR-VP_0-2cm_Jumbo_Phage_48_23|SR-VP_0-2cm** | SRVP18_trench_2_45cm_scaffold_3985_6|SRVP18_trench_2_45cm_UNK|SRVP18_trench_2_45cm | 96.386 | 249 | 7 | 1 | 1 | 247 | 1 | 249 | 7.98E-174 | 491 |
| **SR-VP_0-2_scaffold_141_2510002_83|SR-VP_0-2cm_Jumbo_Phage_48_23|SR-VP_0-2cm** | SRVP18_trench_3_45cm_scaffold_56039_2|SRVP18_trench_3_45cm_UNK|SRVP18_trench_3_45cm | 99.569 | 232 | 1 | 0 | 1 | 232 | 1 | 232 | 3.89E-171 | 483 |
| **SR-VP_0-2_scaffold_141_2510002_83|SR-VP_0-2cm_Jumbo_Phage_48_23|SR-VP_0-2cm** | SRVP18_core_1_40-35cm_scaffold_25693_2|SRVP18_core_1_40-35cm_UNK|SRVP18_core_1_40-35cm | 43.730 | 311 | 147 | 10 | 1 | 289 | 1 | 305 | 3.44E-73 | 237 |
| **SR-VP_0-2_scaffold_141_2510002_83|SR-VP_0-2cm_Jumbo_Phage_48_23|SR-VP_0-2cm** | ALT_03122018_0_1um_scaffold_4613_8|ALT_03122018_0_1um_UNK|ALT_03122018_0_1um | 42.759 | 290 | 147 | 7 | 6 | 289 | 7 | 283 | 1.7E-62 | 209 |
| **SR-VP_0-2_scaffold_141_2510002_83|SR-VP_0-2cm_Jumbo_Phage_48_23|SR-VP_0-2cm** | ALT_09252017_20_scaffold_6914_5|ALT_09252017_20_UNK|ALT_09252017_20 | 43.103 | 290 | 146 | 6 | 6 | 289 | 7 | 283 | 2.73E-62 | 208 |
| **SR-VP_0-2_scaffold_141_2510002_83|SR-VP_0-2cm_Jumbo_Phage_48_23|SR-VP_0-2cm** | S2_018_000_R2_scaffold_7834_4|S2_018_000_R2_UNK|S2_018_000_R2 | 43.537 | 294 | 135 | 13 | 9 | 289 | 18 | 293 | 5.68E-61 | 206 |
| **SR-VP_0-2_scaffold_141_2510002_83|SR-VP_0-2cm_Jumbo_Phage_48_23|SR-VP_0-2cm** | DD1S77SP2S22A_scaffold_134261_1|DD1S77SP2S22A_UNK|DD1S77SP2S22A | 40.210 | 286 | 155 | 5 | 11 | 289 | 23 | 299 | 2.03E-58 | 199 |
| **SR-VP_0-2_scaffold_141_2510002_83|SR-VP_0-2cm_Jumbo_Phage_48_23|SR-VP_0-2cm** | SR-VP_0-2_scaffold_141_7296648_151|SR-VP_0-2cm_UNK|SR-VP_0-2cm | 38.411 | 302 | 170 | 7 | 1 | 289 | 1 | 299 | 6.09E-57 | 195 |
| **SR-VP_0-2_scaffold_141_2510002_83|SR-VP_0-2cm_Jumbo_Phage_48_23|SR-VP_0-2cm** | water-treatment_AWTP-2_inf_bulk_2_scaffold_2370_8|AWTP-2_inf_bulk_2_UNK|AWTP-2_inf_bulk_2 | 35.540 | 287 | 175 | 6 | 9 | 289 | 9 | 291 | 2.63E-54 | 188 |
| **SR-VP_0-2_scaffold_141_2510002_83|SR-VP_0-2cm_Jumbo_Phage_48_23|SR-VP_0-2cm** | LacPavin_0818_WC40_scaffold_238879_prodigal-single_301|LP_PHAGE_38_17|LacPavin_0818_WC40 | 35.640 | 289 | 160 | 6 | 11 | 289 | 19 | 291 | 7.86E-52 | 182 |
| **SR-VP_0-2_scaffold_141_2510002_83|SR-VP_0-2cm_Jumbo_Phage_48_23|SR-VP_0-2cm** | LacPavin_0818_WC40_scaffold_238879_297|LacPavin_0818_WC40_Possible_Phage_38_17|LacPavin_0818_WC40 | 35.640 | 289 | 160 | 6 | 11 | 289 | 19 | 291 | 7.86E-52 | 182 |
| **SR-VP_0-2_scaffold_141_2510002_83|SR-VP_0-2cm_Jumbo_Phage_48_23|SR-VP_0-2cm** | BML_coassembly_scaffold_1858_62|BML_coassembly_UNK|BML_coassembly | 36.656 | 311 | 158 | 9 | 1 | 289 | 61 | 354 | 2.19E-51 | 182 |
| **SR-VP_0-2_scaffold_141_2510002_83|SR-VP_0-2cm_Jumbo_Phage_48_23|SR-VP_0-2cm** | scnpilot_solids2_trim150_scaffold_133_prodigal-single_260|SCN_PHAGE_32_18|SCNPILOT_SOLID2_TRIM150 | 35.764 | 288 | 177 | 4 | 8 | 289 | 13 | 298 | 1.38E-50 | 179 |
| **SR-VP_0-2_scaffold_141_2510002_83|SR-VP_0-2cm_Jumbo_Phage_48_23|SR-VP_0-2cm** | scnpilot_solids2_trim150_scaffold_133_264|SCNPILOT_SOLID2_TRIM150_Phage_3|SCNPILOT_SOLID2_TRIM150 | 35.764 | 288 | 177 | 4 | 8 | 289 | 13 | 298 | 1.38E-50 | 179 |
| **SR-VP_0-2_scaffold_141_2510002_83|SR-VP_0-2cm_Jumbo_Phage_48_23|SR-VP_0-2cm** | scnpilot_solids1_trim150_scaffold_5228_6|SCNPILOT_SOLID_1_TRIM150_UNK|SCNPILOT_SOLID_1_TRIM150 | 35.764 | 288 | 177 | 4 | 8 | 289 | 13 | 298 | 1.38E-50 | 179 |
| **SR-VP_0-2_scaffold_141_2510002_83|SR-VP_0-2cm_Jumbo_Phage_48_23|SR-VP_0-2cm** | SCNpilot_solid_2_scaffold_334_66|SCNpilot_solid_2_UNK|SCNPILOT_SOLID_2 | 35.764 | 288 | 177 | 4 | 8 | 289 | 13 | 298 | 1.38E-50 | 179 |
| **SR-VP_0-2_scaffold_141_2510002_83|SR-VP_0-2cm_Jumbo_Phage_48_23|SR-VP_0-2cm** | SCNpilot_solid_1_scaffold_841_17|SCNpilot_solid_1_UNK|SCNPILOT_SOLID_1 | 35.764 | 288 | 177 | 4 | 8 | 289 | 13 | 298 | 1.38E-50 | 179 |
| **SR-VP_0-2_scaffold_141_2510002_83|SR-VP_0-2cm_Jumbo_Phage_48_23|SR-VP_0-2cm** | SCN_reactora_scaffold_77407_6|SCN_bioreactor_inoc_reactora_UNK|2019_SCN_bioreactor_inoc_reactora | 35.764 | 288 | 177 | 4 | 8 | 289 | 13 | 298 | 1.38E-50 | 179 |
| **SR-VP_0-2_scaffold_141_2510002_83|SR-VP_0-2cm_Jumbo_Phage_48_23|SR-VP_0-2cm** | PAFVLPS_2018_scaffold_107324_2|viral-cat_UNK|viral-cat | 35.034 | 294 | 175 | 4 | 1 | 289 | 1 | 283 | 2.99E-50 | 177 |
| **SR-VP_0-2_scaffold_141_2510002_83|SR-VP_0-2cm_Jumbo_Phage_48_23|SR-VP_0-2cm** | S2_006_000_R2_scaffold_59512_2|S2_006_000_R2_UNK|S2_006_000_R2 | 45.852 | 229 | 99 | 10 | 9 | 225 | 18 | 233 | 3.58E-50 | 176 |
| **SR-VP_0-2_scaffold_141_2510002_83|SR-VP_0-2cm_Jumbo_Phage_48_23|SR-VP_0-2cm** | PAFVLPS_2018_J28_2_scaffold_13797_7|soil-virus-P12-J28-2018_UNK|soil-virus-P12-J28-2018 | 32.990 | 291 | 177 | 6 | 7 | 289 | 11 | 291 | 1.97E-48 | 173 |
| **SR-VP_0-2_scaffold_141_2510002_83|SR-VP_0-2cm_Jumbo_Phage_48_23|SR-VP_0-2cm** | JGI24723J26617_10000007_prodigal-single_103|OS_PHAGE_40_40|JGI_Cruoil_06 | 37.324 | 284 | 163 | 6 | 9 | 289 | 10 | 281 | 2.25E-48 | 172 |
| **SR-VP_0-2_scaffold_141_2510002_83|SR-VP_0-2cm_Jumbo_Phage_48_23|SR-VP_0-2cm** | JGI24723J26617_10000007_743|JGI_Cruoil_06_Phage-like_40_40|JGI_Cruoil_06 | 37.324 | 284 | 163 | 6 | 9 | 289 | 10 | 281 | 2.25E-48 | 172 |
| **SR-VP_0-2_scaffold_141_2510002_83|SR-VP_0-2cm_Jumbo_Phage_48_23|SR-VP_0-2cm** | JGI24723J26617_10000007_133|JGI_Cruoil_06_Phage-like_40_40|JGI_Cruoil_06 | 37.324 | 284 | 163 | 6 | 9 | 289 | 10 | 281 | 2.25E-48 | 172 |
| **SR-VP_0-2_scaffold_141_2510002_83|SR-VP_0-2cm_Jumbo_Phage_48_23|SR-VP_0-2cm** | PAFVLPS_2018_scaffold_671_226|viral-cat_UNK|viral-cat | 32.990 | 291 | 177 | 6 | 7 | 289 | 11 | 291 | 2.37E-48 | 173 |
| **SR-VP_0-2_scaffold_141_2510002_83|SR-VP_0-2cm_Jumbo_Phage_48_23|SR-VP_0-2cm** | DD1S77SP2S22A_scaffold_736_6|DD1S77SP2S22A_concoct.147|DD1S77SP2S22A | 36.789 | 299 | 151 | 9 | 8 | 289 | 7 | 284 | 5.42E-48 | 172 |
| **SR-VP_0-2_scaffold_141_2510002_83|SR-VP_0-2cm_Jumbo_Phage_48_23|SR-VP_0-2cm** | ERMLT890_scaffold_33266_2|ERMLT890_UNK|ERMLT890 | 35.495 | 293 | 169 | 6 | 3 | 289 | 1 | 279 | 1.31E-47 | 171 |
| **SR-VP_0-2_scaffold_141_2510002_83|SR-VP_0-2cm_Jumbo_Phage_48_23|SR-VP_0-2cm** | water-treatment_AWTP-2_inf_bulk_2_scaffold_811_33|AWTP-2_inf_bulk_2_UNK|AWTP-2_inf_bulk_2 | 34.828 | 290 | 160 | 8 | 9 | 289 | 16 | 285 | 2.41E-46 | 167 |
| **SR-VP_0-2_scaffold_141_2510002_83|SR-VP_0-2cm_Jumbo_Phage_48_23|SR-VP_0-2cm** | RHP_09252018_0_1um_scaffold_64_457|RHP_09252018_0_1um_partial_phage_37_39|RHP_09252018_0_1um | 36.585 | 287 | 163 | 8 | 11 | 289 | 19 | 294 | 4.68E-46 | 167 |
| **SR-VP_0-2_scaffold_141_2510002_83|SR-VP_0-2cm_Jumbo_Phage_48_23|SR-VP_0-2cm** | ERMLT366_16_10D_scaffold_35_26|ERMLT366_16_10D_UNK|ERMLT366_16_10D | 34.915 | 295 | 151 | 8 | 2 | 289 | 17 | 277 | 2.43E-43 | 159 |
| **SR-VP_0-2_scaffold_141_2510002_83|SR-VP_0-2cm_Jumbo_Phage_48_23|SR-VP_0-2cm** | AB_092018_0_1um_scaffold_18571_8|AB_092018_0_1um_UNK|AB_092018_0_1um | 35.135 | 296 | 158 | 8 | 9 | 289 | 8 | 284 | 4.74E-43 | 159 |
| **SR-VP_0-2_scaffold_141_2510002_83|SR-VP_0-2cm_Jumbo_Phage_48_23|SR-VP_0-2cm** | PAFVLPS_2018_J22_2_scaffold_81910_2|soil-virus-P6-J22_UNK|soil-virus-P6-J22 | 37.450 | 251 | 139 | 6 | 1 | 245 | 1 | 239 | 8.47E-43 | 157 |
| **SR-VP_0-2_scaffold_141_2510002_83|SR-VP_0-2cm_Jumbo_Phage_48_23|SR-VP_0-2cm** | RHP_09252018_0_1um_scaffold_526_6|RHP_09252018_0_1um_UNK|RHP_09252018_0_1um | 35.915 | 284 | 166 | 7 | 11 | 289 | 411 | 683 | 2.33E-42 | 165 |
| **SR-VP_0-2_scaffold_141_2510002_83|SR-VP_0-2cm_Jumbo_Phage_48_23|SR-VP_0-2cm** | CG_2015-11_scaffold_94845_2|CG_2015-11_UNK|CG_2015-11 | 34.437 | 302 | 168 | 9 | 1 | 289 | 1 | 285 | 1.2E-41 | 155 |
| **SR-VP_0-2_scaffold_141_2510002_83|SR-VP_0-2cm_Jumbo_Phage_48_23|SR-VP_0-2cm** | CG_2015-03_scaffold_9232_3|CG_2015-03_UNK|CG_2015-03 | 34.437 | 302 | 168 | 9 | 1 | 289 | 1 | 285 | 1.2E-41 | 155 |
| **SR-VP_0-2_scaffold_141_2510002_83|SR-VP_0-2cm_Jumbo_Phage_48_23|SR-VP_0-2cm** | CG_2015-01t_scaffold_126_67|CG_2015-01t_UNK|CG_2015-01t | 34.437 | 302 | 168 | 9 | 1 | 289 | 1 | 285 | 1.2E-41 | 155 |
| **SR-VP_0-2_scaffold_141_2510002_83|SR-VP_0-2cm_Jumbo_Phage_48_23|SR-VP_0-2cm** | PAFVLPS_2018_scaffold_335733_3|viral-cat_UNK|viral-cat | 32.765 | 293 | 176 | 6 | 9 | 289 | 18 | 301 | 1.63E-41 | 155 |
| **SR-VP_0-2_scaffold_141_2510002_83|SR-VP_0-2cm_Jumbo_Phage_48_23|SR-VP_0-2cm** | ERMZT366_2_scaffold_2473_8|ERMZT366_2_UNK|ERMZT366_2 | 34.364 | 291 | 163 | 6 | 11 | 289 | 117 | 391 | 2.57E-41 | 157 |
| **SR-VP_0-2_scaffold_141_2510002_83|SR-VP_0-2cm_Jumbo_Phage_48_23|SR-VP_0-2cm** | AB_082018_0_1um_scaffold_34722_2|AB_082018_0_1um_UNK|AB_082018_0_1um | 34.589 | 292 | 157 | 8 | 13 | 289 | 2 | 274 | 4.83E-41 | 154 |
| **SR-VP_0-2_scaffold_141_2510002_83|SR-VP_0-2cm_Jumbo_Phage_48_23|SR-VP_0-2cm** | SR-VP_2-4_scaffold_141_4258783_1|SR-VP_2-4cm_UNK|SR-VP_2-4cm | 52.593 | 135 | 59 | 2 | 1 | 130 | 1 | 135 | 2.13E-40 | 147 |
| **SR-VP_0-2_scaffold_141_2510002_83|SR-VP_0-2cm_Jumbo_Phage_48_23|SR-VP_0-2cm** | FFC_04162018_0_1um_scaffold_21823_1|FFC_04162018_0_1um_UNK|FFC_04162018_0_1um | 36.042 | 283 | 154 | 8 | 11 | 289 | 11 | 270 | 1.11E-39 | 150 |
| **SR-VP_0-2_scaffold_141_2510002_83|SR-VP_0-2cm_Jumbo_Phage_48_23|SR-VP_0-2cm** | PAFVLPS_2018_scaffold_58740_9|viral-cat_UNK|viral-cat | 34.228 | 298 | 171 | 9 | 9 | 289 | 14 | 303 | 1.47E-39 | 150 |
| **SR-VP_0-2_scaffold_141_2510002_83|SR-VP_0-2cm_Jumbo_Phage_48_23|SR-VP_0-2cm** | RTP_08282017_0_2um_scaffold_17_209|RTP_08282017_0_2um_potentially_complete_phage_45_94|RTP_08282017_0_2um | 33.217 | 286 | 173 | 5 | 11 | 289 | 19 | 293 | 2.84E-39 | 149 |
| **SR-VP_0-2_scaffold_141_2510002_83|SR-VP_0-2cm_Jumbo_Phage_48_23|SR-VP_0-2cm** | RHP_09252018_0_1um_scaffold_21_209|RHP_09252018_0_1um_potentially_complete_phage_45_154|RHP_09252018_0_1um | 33.217 | 286 | 173 | 5 | 11 | 289 | 19 | 293 | 2.84E-39 | 149 |
| **SR-VP_0-2_scaffold_141_2510002_83|SR-VP_0-2cm_Jumbo_Phage_48_23|SR-VP_0-2cm** | RTP_09252017_15_scaffold_35525_2|RTP_09252017_15_UNK|RTP_09252017_15 | 33.217 | 286 | 173 | 5 | 11 | 289 | 19 | 293 | 2.84E-39 | 149 |
| **SR-VP_0-2_scaffold_141_2510002_83|SR-VP_0-2cm_Jumbo_Phage_48_23|SR-VP_0-2cm** | SRVP18_hole-7m-from-trench_1_80cm_scaffold_2762_3|SRVP18_hole-7m-from-trench_1_80cm_UNK|SRVP18_hole-7m-from-trench_1_80cm | 32.423 | 293 | 179 | 7 | 9 | 289 | 10 | 295 | 1.71E-38 | 147 |
| **SR-VP_0-2_scaffold_141_2510002_83|SR-VP_0-2cm_Jumbo_Phage_48_23|SR-VP_0-2cm** | SRVP18_hole-7m-from-trench_1_20cm_scaffold_2720_20|SRVP18_hole-7m-from-trench_1_20cm_UNK|SRVP18_hole-7m-from-trench_1_20cm | 32.423 | 293 | 179 | 7 | 9 | 289 | 10 | 295 | 1.71E-38 | 147 |
| **SR-VP_0-2_scaffold_141_2510002_83|SR-VP_0-2cm_Jumbo_Phage_48_23|SR-VP_0-2cm** | CG_2015-01t_scaffold_82_71|CG_2015-01t_UNK|CG_2015-01t | 32.993 | 294 | 167 | 9 | 11 | 289 | 14 | 292 | 1.8E-38 | 147 |
| **SR-VP_0-2_scaffold_141_2510002_83|SR-VP_0-2cm_Jumbo_Phage_48_23|SR-VP_0-2cm** | MISA_07232018_0_1um_scaffold_21548_3|MISA_07232018_0_1um_UNK|MISA_07232018_0_1um | 33.818 | 275 | 157 | 7 | 22 | 289 | 1 | 257 | 7.29E-37 | 142 |
| **SR-VP_0-2_scaffold_141_2510002_83|SR-VP_0-2cm_Jumbo_Phage_48_23|SR-VP_0-2cm** | PAFVLPS_2018_scaffold_88660_4|viral-cat_UNK|viral-cat | 32.432 | 296 | 170 | 8 | 11 | 289 | 11 | 293 | 2.21E-36 | 142 |
| **SR-VP_0-2_scaffold_141_2510002_83|SR-VP_0-2cm_Jumbo_Phage_48_23|SR-VP_0-2cm** | FFC_092018_0_1um_scaffold_3820_3|FFC_092018_0_1um_UNK|FFC_092018_0_1um | 32.770 | 296 | 168 | 7 | 8 | 289 | 244 | 522 | 2.55E-36 | 147 |
| **SR-VP_0-2_scaffold_141_2510002_83|SR-VP_0-2cm_Jumbo_Phage_48_23|SR-VP_0-2cm** | PAFVLPS_2018_scaffold_32338_3|viral-cat_UNK|viral-cat | 32.765 | 293 | 173 | 6 | 11 | 289 | 11 | 293 | 2.59E-36 | 142 |
| **SR-VP_0-2_scaffold_141_2510002_83|SR-VP_0-2cm_Jumbo_Phage_48_23|SR-VP_0-2cm** | PAFVLPS_2018_scaffold_493319_2|viral-cat_UNK|viral-cat | 34.553 | 246 | 140 | 6 | 50 | 289 | 5 | 235 | 2.93E-36 | 140 |
| **SR-VP_0-2_scaffold_141_2510002_83|SR-VP_0-2cm_Jumbo_Phage_48_23|SR-VP_0-2cm** | ERMGT615_2_scaffold_24717_2|ERMGT615_2_UNK|ERMGT615_2 | 36.522 | 230 | 127 | 4 | 11 | 234 | 10 | 226 | 9.41E-36 | 139 |
| **SR-VP_0-2_scaffold_141_2510002_83|SR-VP_0-2cm_Jumbo_Phage_48_23|SR-VP_0-2cm** | FFC_072018_0_1um_scaffold_2375_2|FFC_072018_0_1um_UNK|FFC_072018_0_1um | 32.432 | 296 | 169 | 7 | 8 | 289 | 264 | 542 | 1.57E-35 | 145 |
| **SR-VP_0-2_scaffold_141_2510002_83|SR-VP_0-2cm_Jumbo_Phage_48_23|SR-VP_0-2cm** | FFC_04162018_0_1um_scaffold_6379_1|FFC_04162018_0_1um_UNK|FFC_04162018_0_1um | 31.419 | 296 | 172 | 8 | 8 | 289 | 177 | 455 | 9.54E-35 | 141 |
| **SR-VP_0-2_scaffold_141_2510002_83|SR-VP_0-2cm_Jumbo_Phage_48_23|SR-VP_0-2cm** | PAFVLPS_2018_scaffold_54160_8|viral-cat_UNK|viral-cat | 32.517 | 286 | 158 | 9 | 13 | 289 | 2 | 261 | 1.73E-34 | 136 |
| **SR-VP_0-2_scaffold_141_2510002_83|SR-VP_0-2cm_Jumbo_Phage_48_23|SR-VP_0-2cm** | FFC_082018_0_1um_scaffold_770_17|FFC_082018_0_1um_UNK|FFC_082018_0_1um | 31.419 | 296 | 172 | 8 | 8 | 289 | 264 | 542 | 2.06E-34 | 142 |
| **SR-VP_0-2_scaffold_141_2510002_83|SR-VP_0-2cm_Jumbo_Phage_48_23|SR-VP_0-2cm** | AB_082018_0_1um_scaffold_26788_2|AB_082018_0_1um_UNK|AB_082018_0_1um | 31.081 | 296 | 173 | 8 | 8 | 289 | 135 | 413 | 4.96E-34 | 139 |
| **SR-VP_0-2_scaffold_141_2510002_83|SR-VP_0-2cm_Jumbo_Phage_48_23|SR-VP_0-2cm** | PAFVLPS_2018_scaffold_10591_12|viral-cat_UNK|viral-cat | 30.584 | 291 | 179 | 10 | 9 | 289 | 7 | 284 | 3.44E-33 | 133 |
| **SR-VP_0-2_scaffold_141_2510002_83|SR-VP_0-2cm_Jumbo_Phage_48_23|SR-VP_0-2cm** | PAFVLPS_2018_J30_2_scaffold_661_38|soil-virus-P14-J30-2018_UNK|soil-virus-P14-J30-2018 | 30.584 | 291 | 179 | 10 | 9 | 289 | 7 | 284 | 3.44E-33 | 133 |
| **SR-VP_0-2_scaffold_141_2510002_83|SR-VP_0-2cm_Jumbo_Phage_48_23|SR-VP_0-2cm** | PAFVLPS_2018_J30_2_scaffold_67803_1|soil-virus-P14-J30-2018_UNK|soil-virus-P14-J30-2018 | 31.599 | 269 | 163 | 6 | 33 | 289 | 1 | 260 | 4.44E-33 | 132 |
| **SR-VP_0-2_scaffold_141_2510002_83|SR-VP_0-2cm_Jumbo_Phage_48_23|SR-VP_0-2cm** | AB_092018_0_1um_scaffold_27302_5|AB_092018_0_1um_UNK|AB_092018_0_1um | 33.617 | 235 | 142 | 5 | 8 | 238 | 264 | 488 | 2.5E-32 | 135 |
| **SR-VP_0-2_scaffold_141_2510002_83|SR-VP_0-2cm_Jumbo_Phage_48_23|SR-VP_0-2cm** | BML_02132018_0_5m_scaffold_16455_2|BML_02132018_0_5m_UNK|BML_02132018_0_5m | 29.825 | 285 | 186 | 6 | 11 | 289 | 15 | 291 | 4.4E-32 | 130 |
| **SR-VP_0-2_scaffold_141_2510002_83|SR-VP_0-2cm_Jumbo_Phage_48_23|SR-VP_0-2cm** | CG_2015-04_scaffold_72116_1|CG_2015-04_UNK|CG_2015-04 | 40.351 | 171 | 87 | 4 | 1 | 159 | 1 | 168 | 8.19E-32 | 127 |
| **SR-VP_0-2_scaffold_141_2510002_87|SR-VP_0-2cm_Jumbo_Phage_48_23|SR-VP_0-2cm** | SR-VP_4-6_scaffold_141_5680703_426|SR-VP_4-6cm_Biohub_180515_Phage_48_20|SR-VP_4-6cm_Biohub_180515 | 100.000 | 95 | 0 | 0 | 1 | 95 | 1 | 95 | 8.2E-61 | 190 |
| **SR-VP_0-2_scaffold_141_2510002_87|SR-VP_0-2cm_Jumbo_Phage_48_23|SR-VP_0-2cm** | SR-VP_0-2_scaffold_141_2510002_87|SR-VP_0-2cm_Jumbo_Phage_48_23|SR-VP_0-2cm | 100.000 | 95 | 0 | 0 | 1 | 95 | 1 | 95 | 8.2E-61 | 190 |
| **SR-VP_0-2_scaffold_141_2510002_87|SR-VP_0-2cm_Jumbo_Phage_48_23|SR-VP_0-2cm** | SR-VP_4-6_scaffold_141_5680703_prodigal-single_436|SR-VP_PHAGE_48_20|SR-VP_4-6cm_Biohub_180515 | 100.000 | 86 | 0 | 0 | 10 | 95 | 1 | 86 | 2.71E-53 | 171 |
| **SR-VP_0-2_scaffold_141_2510002_87|SR-VP_0-2cm_Jumbo_Phage_48_23|SR-VP_0-2cm** | SR-VP_0-2_scaffold_141_2510002_prodigal-single_89|SR-VP_PHAGE_48_23_A|SR-VP_0-2cm | 100.000 | 86 | 0 | 0 | 10 | 95 | 1 | 86 | 2.71E-53 | 171 |
| **SR-VP_0-2_scaffold_141_2510002_88|SR-VP_0-2cm_Jumbo_Phage_48_23|SR-VP_0-2cm** | SRVP18_trench_2_45cm_scaffold_8879_5|SRVP18_trench_2_45cm_UNK|SRVP18_trench_2_45cm | 100.000 | 96 | 0 | 0 | 1 | 96 | 1 | 96 | 9.86E-64 | 197 |
| **SR-VP_0-2_scaffold_141_2510002_88|SR-VP_0-2cm_Jumbo_Phage_48_23|SR-VP_0-2cm** | SR-VP_4-6_scaffold_141_5680703_prodigal-single_437|SR-VP_PHAGE_48_20|SR-VP_4-6cm_Biohub_180515 | 100.000 | 96 | 0 | 0 | 1 | 96 | 1 | 96 | 9.86E-64 | 197 |
| **SR-VP_0-2_scaffold_141_2510002_88|SR-VP_0-2cm_Jumbo_Phage_48_23|SR-VP_0-2cm** | SR-VP_4-6_scaffold_141_5680703_427|SR-VP_4-6cm_Biohub_180515_Phage_48_20|SR-VP_4-6cm_Biohub_180515 | 100.000 | 96 | 0 | 0 | 1 | 96 | 1 | 96 | 9.86E-64 | 197 |
| **SR-VP_0-2_scaffold_141_2510002_88|SR-VP_0-2cm_Jumbo_Phage_48_23|SR-VP_0-2cm** | SR-VP_0-2_scaffold_141_2510002_prodigal-single_90|SR-VP_PHAGE_48_23_A|SR-VP_0-2cm | 100.000 | 96 | 0 | 0 | 1 | 96 | 1 | 96 | 9.86E-64 | 197 |
| **SR-VP_0-2_scaffold_141_2510002_88|SR-VP_0-2cm_Jumbo_Phage_48_23|SR-VP_0-2cm** | SR-VP_0-2_scaffold_141_2510002_88|SR-VP_0-2cm_Jumbo_Phage_48_23|SR-VP_0-2cm | 100.000 | 96 | 0 | 0 | 1 | 96 | 1 | 96 | 9.86E-64 | 197 |
| **SR-VP_0-2_scaffold_141_2510002_89|SR-VP_0-2cm_Jumbo_Phage_48_23|SR-VP_0-2cm** | SRVP18_trench_2_45cm_scaffold_8879_4|SRVP18_trench_2_45cm_UNK|SRVP18_trench_2_45cm | 100.000 | 109 | 0 | 0 | 1 | 109 | 1 | 109 | 4.04E-75 | 228 |
| **SR-VP_0-2_scaffold_141_2510002_89|SR-VP_0-2cm_Jumbo_Phage_48_23|SR-VP_0-2cm** | SR-VP_4-6_scaffold_141_5680703_prodigal-single_438|SR-VP_PHAGE_48_20|SR-VP_4-6cm_Biohub_180515 | 100.000 | 109 | 0 | 0 | 1 | 109 | 1 | 109 | 4.04E-75 | 228 |
| **SR-VP_0-2_scaffold_141_2510002_89|SR-VP_0-2cm_Jumbo_Phage_48_23|SR-VP_0-2cm** | SR-VP_4-6_scaffold_141_5680703_428|SR-VP_4-6cm_Biohub_180515_Phage_48_20|SR-VP_4-6cm_Biohub_180515 | 100.000 | 109 | 0 | 0 | 1 | 109 | 1 | 109 | 4.04E-75 | 228 |
| **SR-VP_0-2_scaffold_141_2510002_89|SR-VP_0-2cm_Jumbo_Phage_48_23|SR-VP_0-2cm** | SR-VP_0-2_scaffold_141_2510002_prodigal-single_91|SR-VP_PHAGE_48_23_A|SR-VP_0-2cm | 100.000 | 109 | 0 | 0 | 1 | 109 | 1 | 109 | 4.04E-75 | 228 |
| **SR-VP_0-2_scaffold_141_2510002_89|SR-VP_0-2cm_Jumbo_Phage_48_23|SR-VP_0-2cm** | SR-VP_0-2_scaffold_141_2510002_89|SR-VP_0-2cm_Jumbo_Phage_48_23|SR-VP_0-2cm | 100.000 | 109 | 0 | 0 | 1 | 109 | 1 | 109 | 4.04E-75 | 228 |

| **SR-VP_0-2_scaffold_141_5191495_66|SR-VP_0-2cm_Phage_38_25|SR-VP_0-2cm** | SR-VP_0-2_scaffold_141_5191495_prodigal-single_73|SR-VP_PHAGE_38_25|SR-VP_0-2cm | 100.000 | 1080 | 0 | 0 | 1 | 1080 | 1 | 1080 | 0.0 | 2220 |
| --- | --- | --- | --- | --- | --- | --- | --- | --- | --- | --- | --- |
| **SR-VP_0-2_scaffold_141_5191495_66|SR-VP_0-2cm_Phage_38_25|SR-VP_0-2cm** | SR-VP_0-2_scaffold_141_5191495_66|SR-VP_0-2cm_Phage_38_25|SR-VP_0-2cm | 100.000 | 1080 | 0 | 0 | 1 | 1080 | 1 | 1080 | 0.0 | 2220 |
| **SR-VP_0-2_scaffold_141_5191495_66|SR-VP_0-2cm_Phage_38_25|SR-VP_0-2cm** | ERMZT366_2_scaffold_1600_1|ERMZT366_2_UNK|ERMZT366_2 | 57.490 | 1028 | 426 | 5 | 12 | 1032 | 1 | 1024 | 0.0 | 1244 |
| **SR-VP_0-2_scaffold_141_5191495_66|SR-VP_0-2cm_Phage_38_25|SR-VP_0-2cm** | P0_An_pond3_S8_coassembly_k141_4231480_1|P0_An_pond3_S8_coassembly_UNK|E_GD2017-1_P0_An_pond3_S8_Biohub_coassembly | 88.443 | 623 | 72 | 0 | 10 | 632 | 1 | 623 | 0.0 | 1181 |
| **SR-VP_0-2_scaffold_141_5191495_66|SR-VP_0-2cm_Phage_38_25|SR-VP_0-2cm** | SR-VP_0-2_scaffold_141_6448357_9|SR-VP_0-2cm_UNK|SR-VP_0-2cm | 51.447 | 1071 | 516 | 4 | 10 | 1078 | 1 | 1069 | 0.0 | 1150 |
| **SR-VP_0-2_scaffold_141_5191495_66|SR-VP_0-2cm_Phage_38_25|SR-VP_0-2cm** | S15_GE15_scaffold_591272_2|E_GD2017-2_urea-2_S15_GE15_Biohub_170515_UNK|E_GD2017-2_urea-2_S15_GE15_Biohub_180515 | 89.384 | 584 | 62 | 0 | 10 | 593 | 1 | 584 | 0.0 | 1121 |
| **SR-VP_0-2_scaffold_141_5191495_66|SR-VP_0-2cm_Phage_38_25|SR-VP_0-2cm** | S30_BME30_294872_48|BM_2017_Coates_4_Potentially_Complete_Phage_48-32|BM_2017_Coates_4 | 49.200 | 1063 | 537 | 2 | 19 | 1080 | 11 | 1071 | 0.0 | 1096 |
| **SR-VP_0-2_scaffold_141_5191495_66|SR-VP_0-2cm_Phage_38_25|SR-VP_0-2cm** | S28_BME28_138683_397|BM_2017_Strous_8_UNK|BM_2017_Strous_8 | 49.200 | 1063 | 537 | 2 | 19 | 1080 | 11 | 1071 | 0.0 | 1096 |
| **SR-VP_0-2_scaffold_141_5191495_66|SR-VP_0-2cm_Phage_38_25|SR-VP_0-2cm** | S27_BME27_629333_prodigal-single_185|BM_PHAGE_48_13|BM_2017_Strous_6 | 49.200 | 1063 | 537 | 2 | 19 | 1080 | 11 | 1071 | 0.0 | 1096 |
| **SR-VP_0-2_scaffold_141_5191495_66|SR-VP_0-2cm_Phage_38_25|SR-VP_0-2cm** | S27_BME27_629333_175|BM_2017_Strous_6_Phage_48_13|BM_2017_Strous_6 | 49.200 | 1063 | 537 | 2 | 19 | 1080 | 11 | 1071 | 0.0 | 1096 |
| **SR-VP_0-2_scaffold_141_5191495_66|SR-VP_0-2cm_Phage_38_25|SR-VP_0-2cm** | S22_GE22_scaffold_285059_prodigal-single_514|GD_PHAGE_COMPLETE_48_49|E_GD2017-2_anammox-7_S22_GE22_Biohub_180515 | 49.200 | 1063 | 537 | 2 | 19 | 1080 | 11 | 1071 | 0.0 | 1096 |
| **SR-VP_0-2_scaffold_141_5191495_66|SR-VP_0-2cm_Phage_38_25|SR-VP_0-2cm** | S22_GE22_scaffold_285059_437|E_GD2017-2_anammox-7_S22_GE22_Biohub_170515_Potentially_Complete_48_49|E_GD2017-2_anammox-7_S22_GE22_Biohub_180515 | 49.200 | 1063 | 537 | 2 | 19 | 1080 | 11 | 1071 | 0.0 | 1096 |
| **SR-VP_0-2_scaffold_141_5191495_66|SR-VP_0-2cm_Phage_38_25|SR-VP_0-2cm** | S20_GE20_scaffold_396645_13|E_GD2017-2_strous-5_S20_GE20_Biohub_170515_UNK|E_GD2017-2_strous-5_S20_GE20_Biohub_180515 | 49.200 | 1063 | 537 | 2 | 19 | 1080 | 11 | 1071 | 0.0 | 1096 |
| **SR-VP_0-2_scaffold_141_5191495_66|SR-VP_0-2cm_Phage_38_25|SR-VP_0-2cm** | S15_GE15_scaffold_320047_243|E_GD2017-2_urea-2_S15_GE15_Biohub_170515_UNK|E_GD2017-2_urea-2_S15_GE15_Biohub_180515 | 49.200 | 1063 | 537 | 2 | 19 | 1080 | 11 | 1071 | 0.0 | 1096 |
| **SR-VP_0-2_scaffold_141_5191495_66|SR-VP_0-2cm_Phage_38_25|SR-VP_0-2cm** | S14_GE14_scaffold_311130_13|E_GD2017-2_urea-2_S14_GE14_Biohub_170515_UNK|E_GD2017-2_urea-2_S14_GE14_Biohub_180515 | 49.200 | 1063 | 537 | 2 | 19 | 1080 | 11 | 1071 | 0.0 | 1096 |
| **SR-VP_0-2_scaffold_141_5191495_66|SR-VP_0-2cm_Phage_38_25|SR-VP_0-2cm** | P0_An_pond3_S8_coassembly_k141_2723617_434|P0_An_pond3_S8_coassembly_UNK|E_GD2017-1_P0_An_pond3_S8_Biohub_coassembly | 49.200 | 1063 | 537 | 2 | 19 | 1080 | 11 | 1071 | 0.0 | 1096 |
| **SR-VP_0-2_scaffold_141_5191495_66|SR-VP_0-2cm_Phage_38_25|SR-VP_0-2cm** | P0_An_GD2017L_S7_coassembly_k141_1013362_prodigal-single_80|GD_PHAGE_48_49|E_P0_An_GD2017L_S7_coassembly | 49.200 | 1063 | 537 | 2 | 19 | 1080 | 11 | 1071 | 0.0 | 1096 |
| **SR-VP_0-2_scaffold_141_5191495_66|SR-VP_0-2cm_Phage_38_25|SR-VP_0-2cm** | P0_An_GD2017L_S7_coassembly_k141_1013362_74|P0_An_GD2017L_S7_coassembly_Phage_48_49|E_P0_An_GD2017L_S7_coassembly | 49.200 | 1063 | 537 | 2 | 19 | 1080 | 11 | 1071 | 0.0 | 1096 |
| **SR-VP_0-2_scaffold_141_5191495_66|SR-VP_0-2cm_Phage_38_25|SR-VP_0-2cm** | S19_GE19_scaffold_2259_prodigal-single_281|GD_PHAGE_48_10|E_GD2017-2_strous-5-pellet_S19_GE19_Biohub_180515 | 49.200 | 1063 | 537 | 2 | 19 | 1080 | 11 | 1071 | 0.0 | 1096 |
| **SR-VP_0-2_scaffold_141_5191495_66|SR-VP_0-2cm_Phage_38_25|SR-VP_0-2cm** | S19_GE19_scaffold_2259_257|E_GD2017-2_strous-5-pellet_S19_GE19_Biohub_170515_Phage_48_10|E_GD2017-2_strous-5-pellet_S19_GE19_Biohub_180515 | 49.200 | 1063 | 537 | 2 | 19 | 1080 | 11 | 1071 | 0.0 | 1096 |
| **SR-VP_0-2_scaffold_141_5191495_66|SR-VP_0-2cm_Phage_38_25|SR-VP_0-2cm** | S18_GE18_scaffold_616449_288|E_GD2017-2_strous-5-prefilter_S18_GE18_Biohub_170515_UNK|E_GD2017-2_strous-5-prefilter_S18_GE18_Biohub_180515 | 49.200 | 1063 | 537 | 2 | 19 | 1080 | 11 | 1071 | 0.0 | 1096 |
| **SR-VP_0-2_scaffold_141_5191495_66|SR-VP_0-2cm_Phage_38_25|SR-VP_0-2cm** | S16_GE16_scaffold_390213_390|E_GD2017-2_urea-3_S16_GE16_Biohub_170515_UNK|E_GD2017-2_urea-3_S16_GE16_Biohub_180515 | 49.200 | 1063 | 537 | 2 | 19 | 1080 | 11 | 1071 | 0.0 | 1096 |
| **SR-VP_0-2_scaffold_141_5191495_66|SR-VP_0-2cm_Phage_38_25|SR-VP_0-2cm** | P0_An_pond3_S8_170907_scaffold_1005865_41|E_GD2017-1_P0_AN_POND3_S8_BIOHUB_170907_UNK|E_GD2017-1_P0_An_pond3_S8_Biohub_170907 | 49.200 | 1063 | 537 | 2 | 19 | 1080 | 11 | 1071 | 0.0 | 1096 |
| **SR-VP_0-2_scaffold_141_5191495_66|SR-VP_0-2cm_Phage_38_25|SR-VP_0-2cm** | P0_An_GD2017L_S7_170907_scaffold_359857_24|E_GD2017-1_P0_An_GD2017L_S7_Biohub_170907_UNK|E_GD2017-1_P0_An_GD2017L_S7_Biohub_170907 | 49.200 | 1063 | 537 | 2 | 19 | 1080 | 11 | 1071 | 0.0 | 1096 |
| **SR-VP_0-2_scaffold_141_5191495_66|SR-VP_0-2cm_Phage_38_25|SR-VP_0-2cm** | S2_GD2017_2_manure_scaffold_3_prodigal-single_482|GD_PHAGE_COMPLETE_48_40|GD2017-2_manure_QB3_180125 | 49.200 | 1063 | 537 | 2 | 19 | 1080 | 11 | 1071 | 0.0 | 1096 |
| **SR-VP_0-2_scaffold_141_5191495_66|SR-VP_0-2cm_Phage_38_25|SR-VP_0-2cm** | S2_GD2017_2_manure_scaffold_3_409|GD2017-2_manure_QB3_180125_Potentially_complete_Phage_48_40|GD2017-2_manure_QB3_180125 | 49.200 | 1063 | 537 | 2 | 19 | 1080 | 11 | 1071 | 0.0 | 1096 |
| **SR-VP_0-2_scaffold_141_5191495_66|SR-VP_0-2cm_Phage_38_25|SR-VP_0-2cm** | SR-VP_4-6_scaffold_141_5344030_1|SR-VP_4-6cm_Biohub_180515_UNK|SR-VP_4-6cm_Biohub_180515 | 99.627 | 536 | 2 | 0 | 304 | 839 | 33 | 568 | 0.0 | 1086 |
| **SR-VP_0-2_scaffold_141_5191495_66|SR-VP_0-2cm_Phage_38_25|SR-VP_0-2cm** | GD18-4_manure_scaffold_876276_8|GD2018-4_manure_QB3_180703_UNK|GD2018-4_manure_QB3_180703 | 49.135 | 1040 | 526 | 2 | 19 | 1057 | 11 | 1048 | 0.0 | 1071 |
| **SR-VP_0-2_scaffold_141_5191495_66|SR-VP_0-2cm_Phage_38_25|SR-VP_0-2cm** | SR-VP_0-2_scaffold_141_2432070_17|SR-VP_0-2cm_UNK|SR-VP_0-2cm | 48.037 | 1070 | 553 | 2 | 12 | 1080 | 2 | 1069 | 0.0 | 1070 |
| **SR-VP_0-2_scaffold_141_5191495_66|SR-VP_0-2cm_Phage_38_25|SR-VP_0-2cm** | AB_072018_0_1um_scaffold_612_15|AB_072018_0_1um_UNK|AB_072018_0_1um | 50.933 | 1072 | 506 | 9 | 13 | 1078 | 3 | 1060 | 0.0 | 1064 |
| **SR-VP_0-2_scaffold_141_5191495_66|SR-VP_0-2cm_Phage_38_25|SR-VP_0-2cm** | AB_092018_0_1um_scaffold_3669_7|AB_092018_0_1um_UNK|AB_092018_0_1um | 50.933 | 1072 | 506 | 9 | 13 | 1078 | 3 | 1060 | 0.0 | 1062 |
| **SR-VP_0-2_scaffold_141_5191495_66|SR-VP_0-2cm_Phage_38_25|SR-VP_0-2cm** | AB_082018_0_1um_scaffold_2553_3|AB_082018_0_1um_UNK|AB_082018_0_1um | 50.933 | 1072 | 506 | 9 | 13 | 1078 | 3 | 1060 | 0.0 | 1062 |
| **SR-VP_0-2_scaffold_141_5191495_66|SR-VP_0-2cm_Phage_38_25|SR-VP_0-2cm** | FFC_04162018_0_1um_scaffold_562_4|FFC_04162018_0_1um_UNK|FFC_04162018_0_1um | 50.000 | 1064 | 522 | 5 | 15 | 1078 | 5 | 1058 | 0.0 | 1059 |
| **SR-VP_0-2_scaffold_141_5191495_66|SR-VP_0-2cm_Phage_38_25|SR-VP_0-2cm** | FFC_04162018_0_1um_scaffold_1491_2|FFC_04162018_0_1um_UNK|FFC_04162018_0_1um | 50.749 | 1068 | 506 | 9 | 13 | 1074 | 3 | 1056 | 0.0 | 1057 |
| **SR-VP_0-2_scaffold_141_5191495_66|SR-VP_0-2cm_Phage_38_25|SR-VP_0-2cm** | LacPavin_0419_WC70S_scaffold_945204_4|LacPavin_0419_WC70S_UNK|LacPavin_0419_WC70S | 48.596 | 1068 | 535 | 7 | 15 | 1076 | 5 | 1064 | 0.0 | 1046 |
| **SR-VP_0-2_scaffold_141_5191495_66|SR-VP_0-2cm_Phage_38_25|SR-VP_0-2cm** | LacPavin_0818_WC55_scaffold_113784_prodigal-single_368|LP_PHAGE_COMPLETE_34_34|LacPavin_0818_WC55 | 48.596 | 1068 | 535 | 7 | 15 | 1076 | 5 | 1064 | 0.0 | 1046 |
| **SR-VP_0-2_scaffold_141_5191495_66|SR-VP_0-2cm_Phage_38_25|SR-VP_0-2cm** | LacPavin_0818_WC55_scaffold_113784_357|LacPavin_0818_WC55_Potentially_Complete_Phage_34_34|LacPavin_0818_WC55 | 48.596 | 1068 | 535 | 7 | 15 | 1076 | 5 | 1064 | 0.0 | 1046 |
| **SR-VP_0-2_scaffold_141_5191495_66|SR-VP_0-2cm_Phage_38_25|SR-VP_0-2cm** | LacPavin_0818_WC45_scaffold_80267_117|LacPavin_0818_WC45_UNK|LacPavin_0818_WC45 | 48.596 | 1068 | 535 | 7 | 15 | 1076 | 5 | 1064 | 0.0 | 1046 |
| **SR-VP_0-2_scaffold_141_5191495_66|SR-VP_0-2cm_Phage_38_25|SR-VP_0-2cm** | LacPavin_0718_WC55_scaffold_0_33|LacPavin_0718_WC55_Phage_34_15|LacPavin_0718_WC55 | 48.596 | 1068 | 535 | 7 | 15 | 1076 | 5 | 1064 | 0.0 | 1046 |
| **SR-VP_0-2_scaffold_141_5191495_66|SR-VP_0-2cm_Phage_38_25|SR-VP_0-2cm** | LacPavin_0718_WC45_scaffold_3_117|LacPavin_0718_WC45_UNK|LacPavin_0718_WC45 | 48.596 | 1068 | 535 | 7 | 15 | 1076 | 5 | 1064 | 0.0 | 1046 |
| **SR-VP_0-2_scaffold_141_5191495_66|SR-VP_0-2cm_Phage_38_25|SR-VP_0-2cm** | PLM4_65_b1_redo_sep16_scaffold_45075_3|PLM4_65cm_b1_redo_sep2016_UNK|PLM4_65cm_b1_redo_sep2016 | 52.466 | 953 | 451 | 2 | 10 | 962 | 1 | 951 | 0.0 | 1027 |
| **SR-VP_0-2_scaffold_141_5191495_66|SR-VP_0-2cm_Phage_38_25|SR-VP_0-2cm** | L3a1_full_idba_ud_scaffold_6_227|L3a1_UNK|L3a1 | 47.344 | 1073 | 556 | 4 | 12 | 1080 | 2 | 1069 | 0.0 | 1019 |
| **SR-VP_0-2_scaffold_141_5191495_66|SR-VP_0-2cm_Phage_38_25|SR-VP_0-2cm** | LacPavin_0818_WC40_scaffold_164700_3|LacPavin_0818_WC40_UNK|LacPavin_0818_WC40 | 50.000 | 1018 | 491 | 7 | 66 | 1078 | 21 | 1025 | 0.0 | 991 |
| **SR-VP_0-2_scaffold_141_5191495_66|SR-VP_0-2cm_Phage_38_25|SR-VP_0-2cm** | PAFVLPS_2018_scaffold_386_6|viral-cat_UNK|viral-cat | 45.775 | 1077 | 554 | 9 | 15 | 1078 | 4 | 1063 | 0.0 | 982 |
| **SR-VP_0-2_scaffold_141_5191495_66|SR-VP_0-2cm_Phage_38_25|SR-VP_0-2cm** | BML_coassembly_scaffold_632_30|BML_coassembly_UNK|BML_coassembly | 45.379 | 1082 | 558 | 11 | 10 | 1076 | 1 | 1064 | 0.0 | 947 |
| **SR-VP_0-2_scaffold_141_5191495_66|SR-VP_0-2cm_Phage_38_25|SR-VP_0-2cm** | S15_GE15_scaffold_39268_58|E_GD2017-2_urea-2_S15_GE15_Biohub_170515_UNK|E_GD2017-2_urea-2_S15_GE15_Biohub_180515 | 44.517 | 1076 | 592 | 5 | 10 | 1080 | 1 | 1076 | 0.0 | 946 |
| **SR-VP_0-2_scaffold_141_5191495_66|SR-VP_0-2cm_Phage_38_25|SR-VP_0-2cm** | S16_GE16_scaffold_5545_prodigal-single_28|GD_PHAGE_46_9|E_GD2017-2_urea-3_S16_GE16_Biohub_180515 | 44.517 | 1076 | 592 | 5 | 10 | 1080 | 1 | 1076 | 0.0 | 946 |
| **SR-VP_0-2_scaffold_141_5191495_66|SR-VP_0-2cm_Phage_38_25|SR-VP_0-2cm** | S16_GE16_scaffold_5545_27|E_GD2017-2_urea-3_S16_GE16_Biohub_170515_Phage-like_46_9|E_GD2017-2_urea-3_S16_GE16_Biohub_180515 | 44.517 | 1076 | 592 | 5 | 10 | 1080 | 1 | 1076 | 0.0 | 946 |
| **SR-VP_0-2_scaffold_141_5191495_66|SR-VP_0-2cm_Phage_38_25|SR-VP_0-2cm** | PAFVLPS_2018_scaffold_33_42|circular_33|viral-cat | 44.837 | 1075 | 579 | 8 | 10 | 1076 | 1 | 1069 | 0.0 | 939 |
| **SR-VP_0-2_scaffold_141_5191495_66|SR-VP_0-2cm_Phage_38_25|SR-VP_0-2cm** | PAFVLPS_2018_scaffold_18_356|viral-cat_UNK|viral-cat | 44.517 | 1076 | 590 | 6 | 10 | 1080 | 1 | 1074 | 0.0 | 939 |
| **SR-VP_0-2_scaffold_141_5191495_66|SR-VP_0-2cm_Phage_38_25|SR-VP_0-2cm** | PAFVLPS_2018_J30_2_scaffold_1899_3|soil-virus-P14-J30-2018_UNK|soil-virus-P14-J30-2018 | 44.517 | 1076 | 590 | 6 | 10 | 1080 | 1 | 1074 | 0.0 | 939 |
| **SR-VP_0-2_scaffold_141_5191495_66|SR-VP_0-2cm_Phage_38_25|SR-VP_0-2cm** | SR-VP_0-2_scaffold_141_7948322_1|SR-VP_0-2cm_UNK|SR-VP_0-2cm | 46.364 | 990 | 527 | 3 | 93 | 1080 | 2 | 989 | 0.0 | 936 |
| **SR-VP_0-2_scaffold_141_5191495_66|SR-VP_0-2cm_Phage_38_25|SR-VP_0-2cm** | L3m1_full_idba_ud_scaffold_1665_1|L3m1_UNK|L3m1 | 48.617 | 940 | 469 | 3 | 145 | 1078 | 2 | 933 | 0.0 | 935 |
| **SR-VP_0-2_scaffold_141_5191495_66|SR-VP_0-2cm_Phage_38_25|SR-VP_0-2cm** | BC_09192017_0_5m_scaffold_204_33|BC_09192017_0_5m_UNK|BC_09192017_0_5m | 45.200 | 1073 | 561 | 9 | 14 | 1076 | 3 | 1058 | 0.0 | 926 |
| **SR-VP_0-2_scaffold_141_5191495_66|SR-VP_0-2cm_Phage_38_25|SR-VP_0-2cm** | PAFVLPS_2018_scaffold_128948_1|viral-cat_UNK|viral-cat | 43.462 | 1086 | 589 | 9 | 10 | 1080 | 1 | 1076 | 0.0 | 920 |
| **SR-VP_0-2_scaffold_141_5191495_66|SR-VP_0-2cm_Phage_38_25|SR-VP_0-2cm** | LacPavin_0419_WC53_scaffold_515305_1|LacPavin_0419_WC53_UNK|LacPavin_0419_WC53 | 49.431 | 967 | 471 | 7 | 117 | 1078 | 1 | 954 | 0.0 | 917 |
| **SR-VP_0-2_scaffold_141_5191495_66|SR-VP_0-2cm_Phage_38_25|SR-VP_0-2cm** | P0_An_pond3_S8_coassembly_k141_276347_2|P0_An_pond3_S8_coassembly_UNK|E_GD2017-1_P0_An_pond3_S8_Biohub_coassembly | 43.949 | 1099 | 539 | 13 | 15 | 1077 | 9 | 1066 | 0.0 | 914 |
| **SR-VP_0-2_scaffold_141_5191495_66|SR-VP_0-2cm_Phage_38_25|SR-VP_0-2cm** | P0_An_GD2017L_S7_coassembly_k141_961924_7|P0_An_GD2017L_S7_coassembly_UNK|E_P0_An_GD2017L_S7_coassembly | 43.949 | 1099 | 539 | 13 | 15 | 1077 | 9 | 1066 | 0.0 | 914 |
| **SR-VP_0-2_scaffold_141_5191495_66|SR-VP_0-2cm_Phage_38_25|SR-VP_0-2cm** | P0_An_pond3_S8_170907_scaffold_875447_2|E_GD2017-1_P0_AN_POND3_S8_BIOHUB_170907_UNK|E_GD2017-1_P0_An_pond3_S8_Biohub_170907 | 43.949 | 1099 | 539 | 13 | 15 | 1077 | 9 | 1066 | 0.0 | 914 |
| **SR-VP_0-2_scaffold_141_5191495_66|SR-VP_0-2cm_Phage_38_25|SR-VP_0-2cm** | P0_An_GD2017L_S7_170907_scaffold_2243732_2|E_GD2017-1_P0_An_GD2017L_S7_Biohub_170907_UNK|E_GD2017-1_P0_An_GD2017L_S7_Biohub_170907 | 43.949 | 1099 | 539 | 13 | 15 | 1077 | 9 | 1066 | 0.0 | 914 |
| **SR-VP_0-2_scaffold_141_5191495_66|SR-VP_0-2cm_Phage_38_25|SR-VP_0-2cm** | SR-VP_4-6_scaffold_141_847411_1|SR-VP_4-6cm_Biohub_180515_UNK|SR-VP_4-6cm_Biohub_180515 | 46.448 | 943 | 502 | 2 | 12 | 953 | 2 | 942 | 0.0 | 900 |
| **SR-VP_0-2_scaffold_141_5191495_66|SR-VP_0-2cm_Phage_38_25|SR-VP_0-2cm** | LacPavin_0419_WC70S_scaffold_436490_10|LacPavin_0419_WC70S_UNK|LacPavin_0419_WC70S | 46.285 | 996 | 504 | 9 | 97 | 1080 | 414 | 1390 | 0.0 | 898 |
| **SR-VP_0-2_scaffold_141_5191495_66|SR-VP_0-2cm_Phage_38_25|SR-VP_0-2cm** | LacPavin_0419_WC70S_scaffold_29687_53|LacPavin_0419_WC70S_UNK|LacPavin_0419_WC70S | 43.878 | 1078 | 573 | 15 | 14 | 1078 | 3 | 1061 | 0.0 | 894 |
| **SR-VP_0-2_scaffold_141_5191495_66|SR-VP_0-2cm_Phage_38_25|SR-VP_0-2cm** | LacPavin_0419_WC53_scaffold_459219_54|LacPavin_0419_WC53_UNK|LacPavin_0419_WC53 | 43.878 | 1078 | 573 | 15 | 14 | 1078 | 3 | 1061 | 0.0 | 894 |
| **SR-VP_0-2_scaffold_141_5191495_66|SR-VP_0-2cm_Phage_38_25|SR-VP_0-2cm** | LacPavin_0818_WC40_scaffold_701884_6|LacPavin_0818_WC40_UNK|LacPavin_0818_WC40 | 43.878 | 1078 | 573 | 15 | 14 | 1078 | 3 | 1061 | 0.0 | 894 |
| **SR-VP_0-2_scaffold_141_5191495_66|SR-VP_0-2cm_Phage_38_25|SR-VP_0-2cm** | MISA_07112018_0_1um_scaffold_3654_1|MISA_07112018_0_1um_UNK|MISA_07112018_0_1um | 44.574 | 1032 | 544 | 7 | 42 | 1058 | 1 | 1019 | 0.0 | 885 |
| **SR-VP_0-2_scaffold_141_5191495_66|SR-VP_0-2cm_Phage_38_25|SR-VP_0-2cm** | LacPavin_0419_WC53_scaffold_182236_12|LacPavin_0419_WC53_UNK|LacPavin_0419_WC53 | 44.074 | 1080 | 565 | 13 | 14 | 1076 | 3 | 1060 | 0.0 | 885 |
| **SR-VP_0-2_scaffold_141_5191495_66|SR-VP_0-2cm_Phage_38_25|SR-VP_0-2cm** | LacPavin_0818_WC40_scaffold_227017_5|LacPavin_0818_WC40_UNK|LacPavin_0818_WC40 | 44.074 | 1080 | 565 | 13 | 14 | 1076 | 3 | 1060 | 0.0 | 885 |
| **SR-VP_0-2_scaffold_141_5191495_66|SR-VP_0-2cm_Phage_38_25|SR-VP_0-2cm** | 3300005805_____Ga0079957_1000004_111|imgvr_subset_UNK|imgvr_subset | 43.349 | 1075 | 584 | 13 | 14 | 1078 | 3 | 1062 | 0.0 | 883 |
| **SR-VP_0-2_scaffold_141_5191495_66|SR-VP_0-2cm_Phage_38_25|SR-VP_0-2cm** | P0_An_GD2017L_S7_coassembly_k141_3981987_2|P0_An_GD2017L_S7_coassembly_UNK|E_P0_An_GD2017L_S7_coassembly | 88.684 | 433 | 49 | 0 | 10 | 442 | 1 | 433 | 0.0 | 828 |
| **SR-VP_0-2_scaffold_141_5191495_66|SR-VP_0-2cm_Phage_38_25|SR-VP_0-2cm** | LacPavin_0818_WC40_scaffold_811308_1|LacPavin_0818_WC40_UNK|LacPavin_0818_WC40 | 53.255 | 768 | 349 | 5 | 13 | 779 | 3 | 761 | 0.0 | 813 |
| **SR-VP_0-2_scaffold_141_5191495_66|SR-VP_0-2cm_Phage_38_25|SR-VP_0-2cm** | S16_GE16_scaffold_200857_1|E_GD2017-2_urea-3_S16_GE16_Biohub_170515_UNK|E_GD2017-2_urea-3_S16_GE16_Biohub_180515 | 85.120 | 457 | 68 | 0 | 283 | 739 | 1 | 457 | 0.0 | 810 |
| **SR-VP_0-2_scaffold_141_5191495_66|SR-VP_0-2cm_Phage_38_25|SR-VP_0-2cm** | Salt_Pond_R1_A_D2_MG_scaffold_231_6|JGI_Salt_Pond_R1_A_D2_MG_UNK|JGI_Salt_Pond_R1_A_D2_MG | 40.300 | 1067 | 605 | 9 | 25 | 1076 | 5 | 1054 | 0.0 | 798 |
| **SR-VP_0-2_scaffold_141_5191495_66|SR-VP_0-2cm_Phage_38_25|SR-VP_0-2cm** | BC_09192017_0_5m_scaffold_7473_3|BC_09192017_0_5m_UNK|BC_09192017_0_5m | 47.991 | 871 | 435 | 7 | 213 | 1078 | 1 | 858 | 0.0 | 798 |
| **SR-VP_0-2_scaffold_141_5191495_66|SR-VP_0-2cm_Phage_38_25|SR-VP_0-2cm** | Salt_Pond_R1_C_D1_MG_scaffold_2500_2|JGI_Salt_Pond_R1_C_D1_MG_UNK|JGI_Salt_Pond_R1_C_D1_MG | 40.487 | 1067 | 603 | 9 | 25 | 1076 | 5 | 1054 | 0.0 | 798 |
| **SR-VP_0-2_scaffold_141_5191495_66|SR-VP_0-2cm_Phage_38_25|SR-VP_0-2cm** | Salt_Pond_R1_A_D1_MG_scaffold_1858_5|Salt_Pond_R1_A_D1_MG_UNK|JGI_Salt_Pond_R1_A_D1_MG | 40.487 | 1067 | 603 | 9 | 25 | 1076 | 5 | 1054 | 0.0 | 798 |
| **SR-VP_0-2_scaffold_141_5191495_66|SR-VP_0-2cm_Phage_38_25|SR-VP_0-2cm** | BC_09192017_0_5m_scaffold_13069_6|BC_09192017_0_5m_UNK|BC_09192017_0_5m | 55.940 | 665 | 282 | 3 | 15 | 674 | 5 | 663 | 0.0 | 773 |
| **SR-VP_0-2_scaffold_141_5191495_66|SR-VP_0-2cm_Phage_38_25|SR-VP_0-2cm** | L3a2_full_idba_ud_scaffold_8334_1|L3a2_MaxBin2_EukRep_ggKbase_unknown_002|L3a2 | 45.283 | 848 | 455 | 4 | 201 | 1044 | 2 | 844 | 0.0 | 769 |
| **SR-VP_0-2_scaffold_141_5191495_66|SR-VP_0-2cm_Phage_38_25|SR-VP_0-2cm** | GS605_0p1_scaffold_738_22|lsdeep_GS605_0p1_UNK|lsdeep_GS605_0p1 | 40.202 | 1087 | 590 | 17 | 14 | 1080 | 6 | 1052 | 0.0 | 761 |
| **SR-VP_0-2_scaffold_141_5191495_66|SR-VP_0-2cm_Phage_38_25|SR-VP_0-2cm** | AB_082018_0_1um_scaffold_2922_2|AB_082018_0_1um_UNK|AB_082018_0_1um | 39.405 | 1076 | 608 | 18 | 19 | 1078 | 1 | 1048 | 0.0 | 761 |
| **SR-VP_0-2_scaffold_141_5191495_66|SR-VP_0-2cm_Phage_38_25|SR-VP_0-2cm** | LacPavin_0818_WC55_scaffold_68513_31|LacPavin_0818_WC55_UNK|LacPavin_0818_WC55 | 39.199 | 1074 | 609 | 16 | 21 | 1078 | 2 | 1047 | 0.0 | 761 |
| **SR-VP_0-2_scaffold_141_5191495_66|SR-VP_0-2cm_Phage_38_25|SR-VP_0-2cm** | LacPavin_0718_WC55_scaffold_501_23|LacPavin_0718_WC55_UNK|LacPavin_0718_WC55 | 39.199 | 1074 | 609 | 16 | 21 | 1078 | 2 | 1047 | 0.0 | 761 |
| **SR-VP_0-2_scaffold_141_5191495_66|SR-VP_0-2cm_Phage_38_25|SR-VP_0-2cm** | LacPavin_0818_WC45_scaffold_283915_26|LacPavin_0818_WC45_UNK|LacPavin_0818_WC45 | 39.199 | 1074 | 609 | 16 | 21 | 1078 | 2 | 1047 | 0.0 | 760 |
| **SR-VP_0-2_scaffold_141_5191495_66|SR-VP_0-2cm_Phage_38_25|SR-VP_0-2cm** | AB_072018_0_1um_scaffold_4422_4|AB_072018_0_1um_UNK|AB_072018_0_1um | 39.607 | 1068 | 601 | 18 | 27 | 1078 | 1 | 1040 | 0.0 | 758 |
| **SR-VP_0-2_scaffold_141_5191495_66|SR-VP_0-2cm_Phage_38_25|SR-VP_0-2cm** | Salt_Pond_SF2_C_H2O_MG_scaffold_950_6|JGI_Salt_Pond_SF2_C_H2O_MG_UNK|JGI_Salt_Pond_SF2_C_H2O_MG | 39.171 | 1062 | 614 | 12 | 25 | 1078 | 6 | 1043 | 0.0 | 755 |
| **SR-VP_0-2_scaffold_141_5191495_66|SR-VP_0-2cm_Phage_38_25|SR-VP_0-2cm** | P0_An_pond3_S8_coassembly_k141_3000671_1|P0_An_pond3_S8_coassembly_UNK|E_GD2017-1_P0_An_pond3_S8_Biohub_coassembly | 84.071 | 452 | 72 | 0 | 629 | 1080 | 1 | 452 | 0.0 | 754 |
| **SR-VP_0-2_scaffold_141_5191495_66|SR-VP_0-2cm_Phage_38_25|SR-VP_0-2cm** | FFC_092018_0_1um_scaffold_17_166|FFC_092018_0_1um_partial_phage_31_10|FFC_092018_0_1um | 39.038 | 1081 | 605 | 17 | 19 | 1078 | 1 | 1048 | 0.0 | 753 |
| **SR-VP_0-2_scaffold_141_5191495_66|SR-VP_0-2cm_Phage_38_25|SR-VP_0-2cm** | SR-VP_4-6_scaffold_141_4681429_2|SR-VP_4-6cm_Biohub_180515_UNK|SR-VP_4-6cm_Biohub_180515 | 39.541 | 1090 | 603 | 21 | 15 | 1080 | 3 | 1060 | 0.0 | 751 |
| **SR-VP_0-2_scaffold_141_5191495_66|SR-VP_0-2cm_Phage_38_25|SR-VP_0-2cm** | LacPavin_0818_WC55_scaffold_896135_3|LacPavin_0818_WC55_UNK|LacPavin_0818_WC55 | 37.558 | 1081 | 621 | 18 | 19 | 1078 | 1 | 1048 | 0.0 | 751 |
| **SR-VP_0-2_scaffold_141_5191495_66|SR-VP_0-2cm_Phage_38_25|SR-VP_0-2cm** | H1a2_full_idba_ud_scaffold_13397_7|H1a2_UNK|H1a2 | 55.799 | 638 | 276 | 4 | 15 | 650 | 4 | 637 | 0.0 | 749 |
| **SR-VP_0-2_scaffold_141_5191495_66|SR-VP_0-2cm_Phage_38_25|SR-VP_0-2cm** | SR-VP_0-2_scaffold_141_4769078_1|SR-VP_0-2cm_UNK|SR-VP_0-2cm | 40.351 | 1083 | 592 | 22 | 24 | 1080 | 4 | 1058 | 0.0 | 745 |
| **SR-VP_0-2_scaffold_141_5191495_66|SR-VP_0-2cm_Phage_38_25|SR-VP_0-2cm** | OR_07232018_dam_0_1um_scaffold_14_256|OR_07232018_dam_0_1um_partial_phage_30_17|OR_07232018_dam_0_1um | 39.279 | 1082 | 600 | 20 | 19 | 1078 | 1 | 1047 | 0.0 | 743 |
| **SR-VP_0-2_scaffold_141_5191495_66|SR-VP_0-2cm_Phage_38_25|SR-VP_0-2cm** | FFC_04162018_0_1um_scaffold_557_3|FFC_04162018_0_1um_UNK|FFC_04162018_0_1um | 38.002 | 1071 | 626 | 16 | 21 | 1078 | 2 | 1047 | 0.0 | 738 |
| **SR-VP_0-2_scaffold_141_5191495_66|SR-VP_0-2cm_Phage_38_25|SR-VP_0-2cm** | ALT_082018_0_1um_scaffold_7999_4|ALT_082018_0_1um_UNK|ALT_082018_0_1um | 38.863 | 1073 | 614 | 20 | 21 | 1078 | 4 | 1049 | 0.0 | 738 |
| **SR-VP_0-2_scaffold_141_5191495_66|SR-VP_0-2cm_Phage_38_25|SR-VP_0-2cm** | Salt_Pond_R2_restored_H2O_MG_scaffold_486_2|JGI_Salt_Pond_R2_restored_H2O_MG_UNK|JGI_Salt_Pond_R2_restored_H2O_MG | 38.282 | 1071 | 616 | 14 | 25 | 1080 | 5 | 1045 | 0.0 | 738 |
| **SR-VP_0-2_scaffold_141_5191495_66|SR-VP_0-2cm_Phage_38_25|SR-VP_0-2cm** | BML_08182015_1_5m_scaffold_13_140|BML_08182015_1_5m_UNK|BML_08182015_1_5m | 38.679 | 1060 | 622 | 15 | 25 | 1078 | 17 | 1054 | 0.0 | 738 |
| **SR-VP_0-2_scaffold_141_5191495_66|SR-VP_0-2cm_Phage_38_25|SR-VP_0-2cm** | Salt_Pond_R2_restored_H2O_MG_scaffold_772_11|JGI_Salt_Pond_R2_restored_H2O_MG_UNK|JGI_Salt_Pond_R2_restored_H2O_MG | 38.497 | 1078 | 604 | 16 | 25 | 1080 | 5 | 1045 | 0.0 | 738 |
| **SR-VP_0-2_scaffold_141_5191495_66|SR-VP_0-2cm_Phage_38_25|SR-VP_0-2cm** | BML_coassembly_scaffold_125_135|BML_coassembly_UNK|BML_coassembly | 38.679 | 1060 | 622 | 15 | 25 | 1078 | 17 | 1054 | 0.0 | 738 |
| **SR-VP_0-2_scaffold_141_5191495_66|SR-VP_0-2cm_Phage_38_25|SR-VP_0-2cm** | Salt_Pond_SF2_B_H2O_MG_scaffold_13406_4|JGI_Salt_Pond_SF2_B_H2O_MG_UNK|JGI_Salt_Pond_SF2_B_H2O_MG | 38.960 | 1019 | 587 | 12 | 69 | 1078 | 1 | 993 | 0.0 | 737 |
| **SR-VP_0-2_scaffold_141_5191495_66|SR-VP_0-2cm_Phage_38_25|SR-VP_0-2cm** | Salt_Pond_R1_B_H2O_MG_scaffold_34_29|JGI_Salt_Pond_R1_B_H2O_MG_UNK|JGI_Salt_Pond_R1_B_H2O_MG | 39.554 | 1077 | 597 | 18 | 21 | 1078 | 2 | 1043 | 0.0 | 737 |
| **SR-VP_0-2_scaffold_141_5191495_65|SR-VP_0-2cm_Phage_38_25|SR-VP_0-2cm** | SR-VP_4-6_scaffold_141_6798969_2|SR-VP_4-6cm_Biohub_180515_UNK|SR-VP_4-6cm_Biohub_180515 | 100.000 | 141 | 0 | 0 | 1 | 141 | 1 | 141 | 1.32E-98 | 289 |
| **SR-VP_0-2_scaffold_141_5191495_65|SR-VP_0-2cm_Phage_38_25|SR-VP_0-2cm** | SR-VP_0-2_scaffold_141_5191495_prodigal-single_72|SR-VP_PHAGE_38_25|SR-VP_0-2cm | 100.000 | 141 | 0 | 0 | 1 | 141 | 1 | 141 | 1.32E-98 | 289 |
| **SR-VP_0-2_scaffold_141_5191495_65|SR-VP_0-2cm_Phage_38_25|SR-VP_0-2cm** | SR-VP_0-2_scaffold_141_5191495_65|SR-VP_0-2cm_Phage_38_25|SR-VP_0-2cm | 100.000 | 141 | 0 | 0 | 1 | 141 | 1 | 141 | 1.32E-98 | 289 |
| **SR-VP_0-2_scaffold_141_5191495_65|SR-VP_0-2cm_Phage_38_25|SR-VP_0-2cm** | SR-VP_0-2_scaffold_141_1357281_4|SR-VP_0-2cm_UNK|SR-VP_0-2cm | 50.355 | 141 | 67 | 2 | 1 | 138 | 1 | 141 | 9.13E-35 | 128 |
| **SR-VP_0-2_scaffold_141_5191495_64|SR-VP_0-2cm_Phage_38_25|SR-VP_0-2cm** | SR-VP_0-2_scaffold_141_5191495_prodigal-single_71|SR-VP_PHAGE_38_25|SR-VP_0-2cm | 100.000 | 311 | 0 | 0 | 1 | 311 | 1 | 311 | 0.0 | 640 |
| **SR-VP_0-2_scaffold_141_5191495_64|SR-VP_0-2cm_Phage_38_25|SR-VP_0-2cm** | SR-VP_0-2_scaffold_141_5191495_64|SR-VP_0-2cm_Phage_38_25|SR-VP_0-2cm | 100.000 | 311 | 0 | 0 | 1 | 311 | 1 | 311 | 0.0 | 640 |
| **SR-VP_0-2_scaffold_141_5191495_64|SR-VP_0-2cm_Phage_38_25|SR-VP_0-2cm** | SR-VP_4-6_scaffold_141_1637869_2|SR-VP_4-6cm_Biohub_180515_UNK|SR-VP_4-6cm_Biohub_180515 | 99.531 | 213 | 1 | 0 | 1 | 213 | 1 | 213 | 1.37E-156 | 446 |
| **SR-VP_0-2_scaffold_141_5191495_64|SR-VP_0-2cm_Phage_38_25|SR-VP_0-2cm** | PAFVLPS_2018_scaffold_178920_3|viral-cat_UNK|viral-cat | 63.141 | 312 | 113 | 2 | 1 | 311 | 1 | 311 | 1.5E-143 | 417 |
| **SR-VP_0-2_scaffold_141_5191495_64|SR-VP_0-2cm_Phage_38_25|SR-VP_0-2cm** | SR-VP_0-2_scaffold_141_7274537_3|SR-VP_0-2cm_UNK|SR-VP_0-2cm | 60.256 | 312 | 122 | 2 | 1 | 311 | 1 | 311 | 1.83E-135 | 396 |
| **SR-VP_0-2_scaffold_141_5191495_64|SR-VP_0-2cm_Phage_38_25|SR-VP_0-2cm** | SR-VP_0-2_scaffold_141_6448357_11|SR-VP_0-2cm_UNK|SR-VP_0-2cm | 59.547 | 309 | 123 | 2 | 1 | 308 | 1 | 308 | 6.73E-135 | 395 |
| **SR-VP_0-2_scaffold_141_5191495_64|SR-VP_0-2cm_Phage_38_25|SR-VP_0-2cm** | L3m2_full_idba_ud_scaffold_21988_2|L3m2_UNK|L3m2 | 59.355 | 310 | 123 | 2 | 1 | 309 | 1 | 308 | 3.44E-131 | 385 |
| **SR-VP_0-2_scaffold_141_5191495_64|SR-VP_0-2cm_Phage_38_25|SR-VP_0-2cm** | L3m1_full_idba_ud_scaffold_1103_46|L3m1_UNK|L3m1 | 59.355 | 310 | 123 | 2 | 1 | 309 | 1 | 308 | 3.44E-131 | 385 |
| **SR-VP_0-2_scaffold_141_5191495_64|SR-VP_0-2cm_Phage_38_25|SR-VP_0-2cm** | S15_GE15_scaffold_607369_6|E_GD2017-2_urea-2_S15_GE15_Biohub_170515_UNK|E_GD2017-2_urea-2_S15_GE15_Biohub_180515 | 87.745 | 204 | 24 | 1 | 1 | 204 | 1 | 203 | 8.44E-126 | 368 |
| **SR-VP_0-2_scaffold_141_5191495_64|SR-VP_0-2cm_Phage_38_25|SR-VP_0-2cm** | Mad1_40_16_scaffold_31893_8|Mad1_40_16_UNK|Mad1_40_16 | 57.051 | 312 | 130 | 3 | 1 | 309 | 1 | 311 | 1.14E-125 | 372 |
| **SR-VP_0-2_scaffold_141_5191495_64|SR-VP_0-2cm_Phage_38_25|SR-VP_0-2cm** | SR-VP_0-2_scaffold_141_3175919_4|SR-VP_0-2cm_UNK|SR-VP_0-2cm | 58.934 | 319 | 120 | 6 | 1 | 311 | 1 | 316 | 1.32E-125 | 372 |
| **SR-VP_0-2_scaffold_141_5191495_64|SR-VP_0-2cm_Phage_38_25|SR-VP_0-2cm** | ERMGT157_2_scaffold_21646_2|ERMGT157_2_UNK|ERMGT157_2 | 58.065 | 310 | 125 | 3 | 1 | 309 | 1 | 306 | 8E-124 | 367 |
| **SR-VP_0-2_scaffold_141_5191495_64|SR-VP_0-2cm_Phage_38_25|SR-VP_0-2cm** | LacPavin_0419_WC70S_scaffold_436490_8|LacPavin_0419_WC70S_UNK|LacPavin_0419_WC70S | 56.869 | 313 | 127 | 4 | 1 | 311 | 1 | 307 | 6.8E-123 | 364 |
| **SR-VP_0-2_scaffold_141_5191495_64|SR-VP_0-2cm_Phage_38_25|SR-VP_0-2cm** | PLM4_32_b1_sep16_scaffold_168671_3|PLM4_32cm_b1_sep2016_UNK|PLM4_32cm_b1_sep2016 | 56.230 | 313 | 106 | 4 | 1 | 311 | 1 | 284 | 4.82E-122 | 361 |
| **SR-VP_0-2_scaffold_141_5191495_64|SR-VP_0-2cm_Phage_38_25|SR-VP_0-2cm** | PAFVLPS_2018_scaffold_265627_3|viral-cat_UNK|viral-cat | 55.031 | 318 | 132 | 6 | 1 | 311 | 1 | 314 | 1.37E-117 | 351 |
| **SR-VP_0-2_scaffold_141_5191495_64|SR-VP_0-2cm_Phage_38_25|SR-VP_0-2cm** | H1a2_full_idba_ud_scaffold_13397_6|H1a2_UNK|H1a2 | 55.660 | 318 | 133 | 5 | 1 | 311 | 1 | 317 | 6.64E-117 | 350 |
| **SR-VP_0-2_scaffold_141_5191495_64|SR-VP_0-2cm_Phage_38_25|SR-VP_0-2cm** | PAFVLPS_2018_scaffold_386_7|viral-cat_UNK|viral-cat | 54.088 | 318 | 138 | 5 | 1 | 311 | 1 | 317 | 4.03E-115 | 345 |
| **SR-VP_0-2_scaffold_141_5191495_64|SR-VP_0-2cm_Phage_38_25|SR-VP_0-2cm** | PAFVLPS_2018_scaffold_7515_43|viral-cat_UNK|viral-cat | 54.062 | 320 | 134 | 6 | 1 | 311 | 1 | 316 | 6.1E-112 | 337 |
| **SR-VP_0-2_scaffold_141_5191495_64|SR-VP_0-2cm_Phage_38_25|SR-VP_0-2cm** | PAFVLPS_2018_scaffold_33_41|circular_33|viral-cat | 52.716 | 313 | 142 | 4 | 1 | 311 | 1 | 309 | 2.53E-104 | 317 |
| **SR-VP_0-2_scaffold_141_5191495_64|SR-VP_0-2cm_Phage_38_25|SR-VP_0-2cm** | S15_GE15_scaffold_39268_60|E_GD2017-2_urea-2_S15_GE15_Biohub_170515_UNK|E_GD2017-2_urea-2_S15_GE15_Biohub_180515 | 51.258 | 318 | 142 | 5 | 1 | 308 | 1 | 315 | 5.26E-102 | 312 |
| **SR-VP_0-2_scaffold_141_5191495_64|SR-VP_0-2cm_Phage_38_25|SR-VP_0-2cm** | P0_An_pond3_S8_coassembly_k141_1548338_2|P0_An_pond3_S8_coassembly_UNK|E_GD2017-1_P0_An_pond3_S8_Biohub_coassembly | 51.258 | 318 | 142 | 5 | 1 | 308 | 1 | 315 | 5.26E-102 | 312 |
| **SR-VP_0-2_scaffold_141_5191495_64|SR-VP_0-2cm_Phage_38_25|SR-VP_0-2cm** | S16_GE16_scaffold_5545_prodigal-single_26|GD_PHAGE_46_9|E_GD2017-2_urea-3_S16_GE16_Biohub_180515 | 51.258 | 318 | 142 | 5 | 1 | 308 | 1 | 315 | 5.26E-102 | 312 |
| **SR-VP_0-2_scaffold_141_5191495_64|SR-VP_0-2cm_Phage_38_25|SR-VP_0-2cm** | S16_GE16_scaffold_5545_25|E_GD2017-2_urea-3_S16_GE16_Biohub_170515_Phage-like_46_9|E_GD2017-2_urea-3_S16_GE16_Biohub_180515 | 51.258 | 318 | 142 | 5 | 1 | 308 | 1 | 315 | 5.26E-102 | 312 |
| **SR-VP_0-2_scaffold_141_5191495_64|SR-VP_0-2cm_Phage_38_25|SR-VP_0-2cm** | PAFVLPS_2018_J31_2_scaffold_3728_7|soil-virus-P15-J31-2018_UNK|soil-virus-P15-J31-2018 | 50.479 | 313 | 152 | 3 | 1 | 311 | 1 | 312 | 2.6E-101 | 310 |
| **SR-VP_0-2_scaffold_141_5191495_64|SR-VP_0-2cm_Phage_38_25|SR-VP_0-2cm** | ERMZT366_2_scaffold_1600_2|ERMZT366_2_UNK|ERMZT366_2 | 50.321 | 312 | 145 | 5 | 3 | 309 | 4 | 310 | 1.32E-100 | 308 |
| **SR-VP_0-2_scaffold_141_5191495_64|SR-VP_0-2cm_Phage_38_25|SR-VP_0-2cm** | L1a2_full_idba_ud_scaffold_43783_2|L1a2_UNK|L1a2 | 52.104 | 309 | 140 | 6 | 1 | 304 | 3 | 308 | 3.89E-100 | 307 |
| **SR-VP_0-2_scaffold_141_5191495_64|SR-VP_0-2cm_Phage_38_25|SR-VP_0-2cm** | ERMGT300_2_scaffold_18467_2|ERMGT300_2_UNK|ERMGT300_2 | 48.562 | 313 | 150 | 6 | 3 | 309 | 4 | 311 | 2.41E-97 | 300 |
| **SR-VP_0-2_scaffold_141_5191495_64|SR-VP_0-2cm_Phage_38_25|SR-VP_0-2cm** | LacPavin_0419_WC70S_scaffold_53030_2|LacPavin_0419_WC70S_UNK|LacPavin_0419_WC70S | 47.500 | 320 | 149 | 4 | 1 | 308 | 1 | 313 | 3.06E-94 | 292 |
| **SR-VP_0-2_scaffold_141_5191495_64|SR-VP_0-2cm_Phage_38_25|SR-VP_0-2cm** | PLM4_65_b1_redo_sep16_scaffold_45075_1|PLM4_65cm_b1_redo_sep2016_UNK|PLM4_65cm_b1_redo_sep2016 | 55.472 | 265 | 112 | 4 | 47 | 309 | 3 | 263 | 4.79E-94 | 290 |
| **SR-VP_0-2_scaffold_141_5191495_64|SR-VP_0-2cm_Phage_38_25|SR-VP_0-2cm** | SRVP18_trench_1_20cm_scaffold_44678_3|SRVP18_trench_1_20cm_UNK|SRVP18_trench_1_20cm | 60.177 | 226 | 87 | 2 | 1 | 225 | 1 | 224 | 6.45E-93 | 285 |
| **SR-VP_0-2_scaffold_141_5191495_64|SR-VP_0-2cm_Phage_38_25|SR-VP_0-2cm** | FFC_04162018_0_1um_scaffold_562_5|FFC_04162018_0_1um_UNK|FFC_04162018_0_1um | 46.875 | 320 | 154 | 4 | 1 | 311 | 2 | 314 | 1.59E-92 | 288 |
| **SR-VP_0-2_scaffold_141_5191495_64|SR-VP_0-2cm_Phage_38_25|SR-VP_0-2cm** | BC_09192017_0_5m_scaffold_13069_5|BC_09192017_0_5m_UNK|BC_09192017_0_5m | 46.562 | 320 | 155 | 4 | 1 | 311 | 2 | 314 | 1.94E-92 | 287 |
| **SR-VP_0-2_scaffold_141_5191495_64|SR-VP_0-2cm_Phage_38_25|SR-VP_0-2cm** | BML_07122017_9_5m_scaffold_15179_2|BML_07122017_9_5m_UNK|BML_07122017_9_5m | 46.177 | 327 | 151 | 5 | 1 | 311 | 1 | 318 | 2.92E-92 | 287 |
| **SR-VP_0-2_scaffold_141_5191495_64|SR-VP_0-2cm_Phage_38_25|SR-VP_0-2cm** | BML_coassembly_scaffold_632_31|BML_coassembly_UNK|BML_coassembly | 46.177 | 327 | 151 | 5 | 1 | 311 | 1 | 318 | 2.92E-92 | 287 |
| **SR-VP_0-2_scaffold_141_5191495_64|SR-VP_0-2cm_Phage_38_25|SR-VP_0-2cm** | LacPavin_0419_WC70S_scaffold_945204_3|LacPavin_0419_WC70S_UNK|LacPavin_0419_WC70S | 45.181 | 332 | 149 | 4 | 1 | 311 | 2 | 321 | 5.46E-91 | 284 |
| **SR-VP_0-2_scaffold_141_5191495_64|SR-VP_0-2cm_Phage_38_25|SR-VP_0-2cm** | LacPavin_0818_WC55_scaffold_113784_prodigal-single_367|LP_PHAGE_COMPLETE_34_34|LacPavin_0818_WC55 | 45.181 | 332 | 149 | 4 | 1 | 311 | 2 | 321 | 5.46E-91 | 284 |
| **SR-VP_0-2_scaffold_141_5191495_64|SR-VP_0-2cm_Phage_38_25|SR-VP_0-2cm** | LacPavin_0818_WC55_scaffold_113784_356|LacPavin_0818_WC55_Potentially_Complete_Phage_34_34|LacPavin_0818_WC55 | 45.181 | 332 | 149 | 4 | 1 | 311 | 2 | 321 | 5.46E-91 | 284 |
| **SR-VP_0-2_scaffold_141_5191495_64|SR-VP_0-2cm_Phage_38_25|SR-VP_0-2cm** | LacPavin_0818_WC45_scaffold_80267_116|LacPavin_0818_WC45_UNK|LacPavin_0818_WC45 | 45.181 | 332 | 149 | 4 | 1 | 311 | 2 | 321 | 5.46E-91 | 284 |
| **SR-VP_0-2_scaffold_141_5191495_64|SR-VP_0-2cm_Phage_38_25|SR-VP_0-2cm** | LacPavin_0718_WC55_scaffold_0_34|LacPavin_0718_WC55_Phage_34_15|LacPavin_0718_WC55 | 45.181 | 332 | 149 | 4 | 1 | 311 | 2 | 321 | 5.46E-91 | 284 |
| **SR-VP_0-2_scaffold_141_5191495_64|SR-VP_0-2cm_Phage_38_25|SR-VP_0-2cm** | LacPavin_0718_WC45_scaffold_3_116|LacPavin_0718_WC45_UNK|LacPavin_0718_WC45 | 45.181 | 332 | 149 | 4 | 1 | 311 | 2 | 321 | 5.46E-91 | 284 |
| **SR-VP_0-2_scaffold_141_5191495_64|SR-VP_0-2cm_Phage_38_25|SR-VP_0-2cm** | ERMGT642_2_scaffold_16862_2|ERMGT642_2_UNK|ERMGT642_2 | 47.771 | 314 | 160 | 3 | 1 | 311 | 1 | 313 | 2.92E-90 | 282 |
| **SR-VP_0-2_scaffold_141_5191495_64|SR-VP_0-2cm_Phage_38_25|SR-VP_0-2cm** | PAFVLPS_2018_J28_2_scaffold_6032_6|soil-virus-P12-J28-2018_UNK|soil-virus-P12-J28-2018 | 51.923 | 260 | 118 | 4 | 26 | 279 | 2 | 260 | 8.79E-89 | 276 |
| **SR-VP_0-2_scaffold_141_5191495_64|SR-VP_0-2cm_Phage_38_25|SR-VP_0-2cm** | LacPavin_0818_WC45_scaffold_305942_29|LacPavin_0818_WC45_UNK|LacPavin_0818_WC45 | 47.826 | 322 | 149 | 6 | 1 | 311 | 1 | 314 | 1.66E-88 | 277 |
| **SR-VP_0-2_scaffold_141_5191495_64|SR-VP_0-2cm_Phage_38_25|SR-VP_0-2cm** | SR-VP_2-4_scaffold_141_6534590_1|SR-VP_2-4cm_UNK|SR-VP_2-4cm | 44.850 | 301 | 162 | 3 | 1 | 298 | 1 | 300 | 1.97E-87 | 274 |
| **SR-VP_0-2_scaffold_141_5191495_64|SR-VP_0-2cm_Phage_38_25|SR-VP_0-2cm** | LacPavin_0818_WC50_scaffold_515436_1|LacPavin_0818_WC50_UNK|LacPavin_0818_WC50 | 43.810 | 315 | 167 | 4 | 2 | 310 | 1 | 311 | 5.86E-87 | 273 |
| **SR-VP_0-2_scaffold_141_5191495_64|SR-VP_0-2cm_Phage_38_25|SR-VP_0-2cm** | L3a2_full_idba_ud_scaffold_20020_5|L3a2_UNK|L3a2 | 45.483 | 321 | 162 | 6 | 1 | 311 | 1 | 318 | 6.45E-87 | 273 |
| **SR-VP_0-2_scaffold_141_5191495_64|SR-VP_0-2cm_Phage_38_25|SR-VP_0-2cm** | L3a1_full_idba_ud_scaffold_6_225|L3a1_UNK|L3a1 | 45.483 | 321 | 162 | 6 | 1 | 311 | 1 | 318 | 6.45E-87 | 273 |
| **SR-VP_0-2_scaffold_141_5191495_64|SR-VP_0-2cm_Phage_38_25|SR-VP_0-2cm** | LacPavin_0818_WC40_scaffold_428090_48|LacPavin_0818_WC40_UNK|LacPavin_0818_WC40 | 43.810 | 315 | 167 | 4 | 2 | 310 | 1 | 311 | 1.82E-86 | 272 |
| **SR-VP_0-2_scaffold_141_5191495_64|SR-VP_0-2cm_Phage_38_25|SR-VP_0-2cm** | GS605_0p1_scaffold_738_20|lsdeep_GS605_0p1_UNK|lsdeep_GS605_0p1 | 43.226 | 310 | 170 | 3 | 2 | 309 | 1 | 306 | 5.09E-86 | 271 |
| **SR-VP_0-2_scaffold_141_5191495_64|SR-VP_0-2cm_Phage_38_25|SR-VP_0-2cm** | SR-VP_4-6_scaffold_141_6798969_3|SR-VP_4-6cm_Biohub_180515_UNK|SR-VP_4-6cm_Biohub_180515 | 99.242 | 132 | 1 | 0 | 180 | 311 | 1 | 132 | 1.81E-85 | 263 |
| **SR-VP_0-2_scaffold_141_5191495_64|SR-VP_0-2cm_Phage_38_25|SR-VP_0-2cm** | Salt_Pond_R2_restored_H2O_MG_scaffold_217_27|JGI_Salt_Pond_R2_restored_H2O_MG_UNK|JGI_Salt_Pond_R2_restored_H2O_MG | 45.631 | 309 | 160 | 5 | 3 | 308 | 7 | 310 | 3.73E-85 | 269 |
| **SR-VP_0-2_scaffold_141_5191495_64|SR-VP_0-2cm_Phage_38_25|SR-VP_0-2cm** | P0_An_pond3_S8_coassembly_k141_276347_1|P0_An_pond3_S8_coassembly_UNK|E_GD2017-1_P0_An_pond3_S8_Biohub_coassembly | 42.271 | 317 | 173 | 4 | 1 | 311 | 3 | 315 | 4.03E-85 | 269 |
| **SR-VP_0-2_scaffold_141_5191495_64|SR-VP_0-2cm_Phage_38_25|SR-VP_0-2cm** | P0_An_GD2017L_S7_170907_scaffold_2243732_1|E_GD2017-1_P0_An_GD2017L_S7_Biohub_170907_UNK|E_GD2017-1_P0_An_GD2017L_S7_Biohub_170907 | 42.271 | 317 | 173 | 4 | 1 | 311 | 3 | 315 | 4.03E-85 | 269 |
| **SR-VP_0-2_scaffold_141_5191495_64|SR-VP_0-2cm_Phage_38_25|SR-VP_0-2cm** | LacPavin_0818_WC40_scaffold_329966_1|LacPavin_0818_WC40_UNK|LacPavin_0818_WC40 | 44.660 | 309 | 161 | 4 | 8 | 310 | 1 | 305 | 7.27E-85 | 268 |
| **SR-VP_0-2_scaffold_141_5191495_64|SR-VP_0-2cm_Phage_38_25|SR-VP_0-2cm** | Salt_Pond_SF2_A_H2O_MG_scaffold_17766_3|JGI_Salt_Pond_SF2_A_H2O_MG_UNK|JGI_Salt_Pond_SF2_A_H2O_MG | 47.241 | 290 | 146 | 3 | 23 | 308 | 2 | 288 | 8.54E-85 | 267 |
| **SR-VP_0-2_scaffold_141_5191495_64|SR-VP_0-2cm_Phage_38_25|SR-VP_0-2cm** | LacPavin_0818_WC40_scaffold_164700_2|LacPavin_0818_WC40_UNK|LacPavin_0818_WC40 | 46.250 | 320 | 159 | 5 | 1 | 311 | 2 | 317 | 8.69E-85 | 268 |
| **SR-VP_0-2_scaffold_141_5191495_64|SR-VP_0-2cm_Phage_38_25|SR-VP_0-2cm** | Salt_Pond_SF2_B_H2O_MG_scaffold_6150_4|JGI_Salt_Pond_SF2_B_H2O_MG_UNK|JGI_Salt_Pond_SF2_B_H2O_MG | 44.127 | 315 | 165 | 5 | 2 | 310 | 1 | 310 | 1.04E-84 | 268 |
| **SR-VP_0-2_scaffold_141_5191495_64|SR-VP_0-2cm_Phage_38_25|SR-VP_0-2cm** | Salt_Pond_SF2_A_H2O_MG_scaffold_10650_2|JGI_Salt_Pond_SF2_A_H2O_MG_UNK|JGI_Salt_Pond_SF2_A_H2O_MG | 44.127 | 315 | 165 | 5 | 2 | 310 | 1 | 310 | 1.04E-84 | 268 |
| **SR-VP_0-2_scaffold_141_5191495_64|SR-VP_0-2cm_Phage_38_25|SR-VP_0-2cm** | Salt_Pond_SF2_C_H2O_MG_scaffold_9791_1|JGI_Salt_Pond_SF2_C_H2O_MG_UNK|JGI_Salt_Pond_SF2_C_H2O_MG | 44.127 | 315 | 165 | 5 | 2 | 310 | 1 | 310 | 1.04E-84 | 268 |
| **SR-VP_0-2_scaffold_141_5191495_64|SR-VP_0-2cm_Phage_38_25|SR-VP_0-2cm** | Salt_Pond_R2_restored_H2O_MG_scaffold_200_2|JGI_Salt_Pond_R2_restored_H2O_MG_UNK|JGI_Salt_Pond_R2_restored_H2O_MG | 44.156 | 308 | 165 | 4 | 4 | 308 | 7 | 310 | 5.24E-84 | 266 |
| **SR-VP_0-2_scaffold_141_5191495_64|SR-VP_0-2cm_Phage_38_25|SR-VP_0-2cm** | ALT_082018_0_1um_scaffold_60661_1|ALT_082018_0_1um_UNK|ALT_082018_0_1um | 45.625 | 320 | 160 | 6 | 1 | 311 | 1 | 315 | 6.87E-84 | 266 |
| **SR-VP_0-2_scaffold_141_5191495_64|SR-VP_0-2cm_Phage_38_25|SR-VP_0-2cm** | Salt_Pond_SF2_A_H2O_MG_scaffold_22849_5|JGI_Salt_Pond_SF2_A_H2O_MG_UNK|JGI_Salt_Pond_SF2_A_H2O_MG | 44.013 | 309 | 166 | 4 | 4 | 309 | 7 | 311 | 1.24E-83 | 265 |
| **SR-VP_0-2_scaffold_141_5191495_64|SR-VP_0-2cm_Phage_38_25|SR-VP_0-2cm** | Salt_Pond_R2A_B_H2O_MG_scaffold_32192_3|JGI_Salt_Pond_R2A_B_H2O_MG_UNK|JGI_Salt_Pond_R2A_B_H2O_MG | 44.013 | 309 | 166 | 4 | 4 | 309 | 7 | 311 | 1.24E-83 | 265 |
| **SR-VP_0-2_scaffold_141_5191495_64|SR-VP_0-2cm_Phage_38_25|SR-VP_0-2cm** | FFC_092018_0_1um_scaffold_9152_2|FFC_092018_0_1um_UNK|FFC_092018_0_1um | 45.511 | 323 | 153 | 6 | 1 | 311 | 2 | 313 | 1.5E-83 | 265 |
| **SR-VP_0-2_scaffold_141_5191495_64|SR-VP_0-2cm_Phage_38_25|SR-VP_0-2cm** | L3m1_full_idba_ud_scaffold_69292_1|L3m1_UNK|L3m1 | 54.622 | 238 | 105 | 2 | 75 | 311 | 5 | 240 | 1.87E-83 | 262 |
| **SR-VP_0-2_scaffold_141_5191495_64|SR-VP_0-2cm_Phage_38_25|SR-VP_0-2cm** | Salt_Pond_SF2_B_H2O_MG_scaffold_3586_7|JGI_Salt_Pond_SF2_B_H2O_MG_UNK|JGI_Salt_Pond_SF2_B_H2O_MG | 44.013 | 309 | 166 | 4 | 4 | 309 | 7 | 311 | 1.95E-83 | 265 |
| **SR-VP_0-2_scaffold_141_5191495_64|SR-VP_0-2cm_Phage_38_25|SR-VP_0-2cm** | Salt_Pond_R2_restored_H2O_MG_scaffold_186_3|JGI_Salt_Pond_R2_restored_H2O_MG_UNK|JGI_Salt_Pond_R2_restored_H2O_MG | 43.408 | 311 | 166 | 4 | 6 | 310 | 5 | 311 | 2.59E-83 | 264 |
| **SR-VP_0-2_scaffold_141_5191495_64|SR-VP_0-2cm_Phage_38_25|SR-VP_0-2cm** | LacPavin_0419_WC53_scaffold_69987_2|LacPavin_0419_WC53_UNK|LacPavin_0419_WC53 | 45.820 | 323 | 156 | 5 | 1 | 311 | 2 | 317 | 3.04E-83 | 264 |
| **SR-VP_0-2_scaffold_141_5191495_64|SR-VP_0-2cm_Phage_38_25|SR-VP_0-2cm** | P0_An_GD2017L_S7_coassembly_k141_961924_6|P0_An_GD2017L_S7_coassembly_UNK|E_P0_An_GD2017L_S7_coassembly | 41.270 | 315 | 177 | 4 | 1 | 311 | 4 | 314 | 6.11E-83 | 263 |
| **SR-VP_0-2_scaffold_141_5191495_64|SR-VP_0-2cm_Phage_38_25|SR-VP_0-2cm** | ERMGT418_2_scaffold_35482_1|ERMGT418_2_UNK|ERMGT418_2 | 51.362 | 257 | 119 | 3 | 54 | 309 | 1 | 252 | 6.65E-83 | 261 |
| **SR-VP_0-2_scaffold_141_5191495_64|SR-VP_0-2cm_Phage_38_25|SR-VP_0-2cm** | gwa1_scaffold_152_159|GWA1_scaffold_152_phage_34_9|GWA1 | 44.118 | 306 | 164 | 4 | 6 | 310 | 17 | 316 | 6.71E-83 | 263 |
| **SR-VP_0-2_scaffold_141_5191495_64|SR-VP_0-2cm_Phage_38_25|SR-VP_0-2cm** | AB_092018_0_1um_scaffold_3669_6|AB_092018_0_1um_UNK|AB_092018_0_1um | 45.820 | 323 | 156 | 5 | 1 | 311 | 2 | 317 | 1.09E-82 | 263 |
| **SR-VP_0-2_scaffold_141_5191495_64|SR-VP_0-2cm_Phage_38_25|SR-VP_0-2cm** | Salt_Pond_SF2_C_H2O_MG_scaffold_5031_8|JGI_Salt_Pond_SF2_C_H2O_MG_UNK|JGI_Salt_Pond_SF2_C_H2O_MG | 43.506 | 308 | 167 | 4 | 4 | 308 | 7 | 310 | 1.17E-82 | 262 |
| **SR-VP_0-2_scaffold_141_5191495_64|SR-VP_0-2cm_Phage_38_25|SR-VP_0-2cm** | AB_082018_0_1um_scaffold_2553_2|AB_082018_0_1um_UNK|AB_082018_0_1um | 45.820 | 323 | 156 | 5 | 1 | 311 | 2 | 317 | 1.44E-82 | 262 |
| **SR-VP_0-2_scaffold_141_5191495_64|SR-VP_0-2cm_Phage_38_25|SR-VP_0-2cm** | Salt_Pond_SF2_B_H2O_MG_scaffold_138_83|JGI_Salt_Pond_SF2_B_H2O_MG_UNK|JGI_Salt_Pond_SF2_B_H2O_MG | 42.444 | 311 | 169 | 4 | 4 | 308 | 2 | 308 | 1.56E-82 | 262 |
| **SR-VP_0-2_scaffold_141_5191495_64|SR-VP_0-2cm_Phage_38_25|SR-VP_0-2cm** | Salt_Pond_R2A_C_H2O_MG_scaffold_2826_3|JGI_Salt_Pond_R2A_C_H2O_MG_UNK|JGI_Salt_Pond_R2A_C_H2O_MG | 42.444 | 311 | 169 | 4 | 4 | 308 | 2 | 308 | 1.56E-82 | 262 |
| **SR-VP_0-2_scaffold_141_5191495_64|SR-VP_0-2cm_Phage_38_25|SR-VP_0-2cm** | Salt_Pond_SF2_C_H2O_MG_scaffold_161_8|JGI_Salt_Pond_SF2_C_H2O_MG_UNK|JGI_Salt_Pond_SF2_C_H2O_MG | 42.444 | 311 | 169 | 4 | 4 | 308 | 2 | 308 | 1.56E-82 | 262 |
| **SR-VP_0-2_scaffold_141_5191495_64|SR-VP_0-2cm_Phage_38_25|SR-VP_0-2cm** | AB_082018_0_1um_scaffold_62521_1|AB_082018_0_1um_UNK|AB_082018_0_1um | 43.506 | 308 | 161 | 4 | 7 | 309 | 6 | 305 | 1.67E-82 | 262 |
| **SR-VP_0-2_scaffold_141_5191495_64|SR-VP_0-2cm_Phage_38_25|SR-VP_0-2cm** | FFC_04162018_0_1um_scaffold_1491_3|FFC_04162018_0_1um_UNK|FFC_04162018_0_1um | 45.820 | 323 | 156 | 5 | 1 | 311 | 2 | 317 | 1.67E-82 | 262 |
| **SR-VP_0-2_scaffold_141_5191495_64|SR-VP_0-2cm_Phage_38_25|SR-VP_0-2cm** | LacPavin_0818_WC55_scaffold_68513_30|LacPavin_0818_WC55_UNK|LacPavin_0818_WC55 | 45.603 | 307 | 158 | 5 | 6 | 309 | 9 | 309 | 1.72E-82 | 262 |
| **SR-VP_0-2_scaffold_141_5191495_64|SR-VP_0-2cm_Phage_38_25|SR-VP_0-2cm** | LacPavin_0818_WC45_scaffold_283915_27|LacPavin_0818_WC45_UNK|LacPavin_0818_WC45 | 45.603 | 307 | 158 | 5 | 6 | 309 | 9 | 309 | 1.72E-82 | 262 |
| **SR-VP_0-2_scaffold_141_5191495_64|SR-VP_0-2cm_Phage_38_25|SR-VP_0-2cm** | LacPavin_0818_WC40_scaffold_515987_11|LacPavin_0818_WC40_UNK|LacPavin_0818_WC40 | 45.603 | 307 | 158 | 5 | 6 | 309 | 9 | 309 | 1.72E-82 | 262 |
| **SR-VP_0-2_scaffold_141_5191495_64|SR-VP_0-2cm_Phage_38_25|SR-VP_0-2cm** | LacPavin_0718_WC55_scaffold_501_24|LacPavin_0718_WC55_UNK|LacPavin_0718_WC55 | 45.603 | 307 | 158 | 5 | 6 | 309 | 9 | 309 | 1.72E-82 | 262 |
| **SR-VP_0-2_scaffold_141_5191495_64|SR-VP_0-2cm_Phage_38_25|SR-VP_0-2cm** | Salt_Pond_R2A_B_H2O_MG_scaffold_40727_1|JGI_Salt_Pond_R2A_B_H2O_MG_UNK|JGI_Salt_Pond_R2A_B_H2O_MG | 45.424 | 295 | 151 | 4 | 4 | 292 | 2 | 292 | 2.45E-82 | 261 |
| **SR-VP_0-2_scaffold_141_5191495_64|SR-VP_0-2cm_Phage_38_25|SR-VP_0-2cm** | gwc1_scaffold_7733_5|GWC1 | 43.791 | 306 | 165 | 4 | 6 | 310 | 4 | 303 | 2.53E-82 | 261 |
| **SR-VP_0-2_scaffold_141_5191495_64|SR-VP_0-2cm_Phage_38_25|SR-VP_0-2cm** | GWB1_scaffold_9691_4|GWB1 | 43.791 | 306 | 165 | 4 | 6 | 310 | 4 | 303 | 2.53E-82 | 261 |
| **SR-VP_0-2_scaffold_141_5191495_64|SR-VP_0-2cm_Phage_38_25|SR-VP_0-2cm** | SW_scaffold_85616_4|LAC_SW_UNK|lac_sw | 43.464 | 306 | 166 | 4 | 7 | 309 | 10 | 311 | 2.76E-82 | 261 |
| **SR-VP_0-2_scaffold_141_5191495_64|SR-VP_0-2cm_Phage_38_25|SR-VP_0-2cm** | AB_072018_0_1um_scaffold_612_14|AB_072018_0_1um_UNK|AB_072018_0_1um | 45.820 | 323 | 156 | 5 | 1 | 311 | 2 | 317 | 4.03E-82 | 261 |
| **SR-VP_0-2_scaffold_141_5191495_64|SR-VP_0-2cm_Phage_38_25|SR-VP_0-2cm** | GS605_0p1_scaffold_613_28|lsdeep_GS605_0p1_UNK|lsdeep_GS605_0p1 | 42.810 | 306 | 171 | 2 | 6 | 310 | 2 | 304 | 4.49E-82 | 261 |
| **SR-VP_0-2_scaffold_141_5191495_64|SR-VP_0-2cm_Phage_38_25|SR-VP_0-2cm** | Salt_Pond_SF2_B_H2O_MG_scaffold_645_2|JGI_Salt_Pond_SF2_B_H2O_MG_UNK|JGI_Salt_Pond_SF2_B_H2O_MG | 42.765 | 311 | 168 | 4 | 4 | 308 | 2 | 308 | 5.49E-82 | 261 |
| **SR-VP_0-2_scaffold_141_5191495_64|SR-VP_0-2cm_Phage_38_25|SR-VP_0-2cm** | Salt_Pond_R2_restored_H2O_MG_scaffold_99_63|JGI_Salt_Pond_R2_restored_H2O_MG_UNK|JGI_Salt_Pond_R2_restored_H2O_MG | 42.444 | 311 | 169 | 4 | 4 | 308 | 2 | 308 | 6.46E-82 | 260 |
| **SR-VP_0-2_scaffold_141_5191495_64|SR-VP_0-2cm_Phage_38_25|SR-VP_0-2cm** | RHP_09252018_0_1um_scaffold_13831_4|RHP_09252018_0_1um_UNK|RHP_09252018_0_1um | 44.127 | 315 | 165 | 5 | 2 | 310 | 1 | 310 | 9.23E-82 | 260 |
| **SR-VP_0-2_scaffold_141_5191495_64|SR-VP_0-2cm_Phage_38_25|SR-VP_0-2cm** | Salt_Pond_SF2_B_H2O_MG_scaffold_415_25|JGI_Salt_Pond_SF2_B_H2O_MG_UNK|JGI_Salt_Pond_SF2_B_H2O_MG | 44.654 | 318 | 161 | 7 | 1 | 311 | 1 | 310 | 9.79E-82 | 260 |
| **SR-VP_0-2_scaffold_141_5191495_64|SR-VP_0-2cm_Phage_38_25|SR-VP_0-2cm** | Salt_Pond_SF2_C_H2O_MG_scaffold_10454_5|JGI_Salt_Pond_SF2_C_H2O_MG_UNK|JGI_Salt_Pond_SF2_C_H2O_MG | 44.654 | 318 | 161 | 7 | 1 | 311 | 1 | 310 | 1.04E-81 | 260 |
| **SR-VP_0-2_scaffold_141_5191495_64|SR-VP_0-2cm_Phage_38_25|SR-VP_0-2cm** | Salt_Pond_SF2_A_H2O_MG_scaffold_4505_4|JGI_Salt_Pond_SF2_A_H2O_MG_UNK|JGI_Salt_Pond_SF2_A_H2O_MG | 42.765 | 311 | 168 | 4 | 4 | 308 | 2 | 308 | 1.11E-81 | 260 |
| **SR-VP_0-2_scaffold_141_5191495_64|SR-VP_0-2cm_Phage_38_25|SR-VP_0-2cm** | BML_08182015_1_5m_scaffold_2384_2|BML_08182015_1_5m_UNK|BML_08182015_1_5m | 42.395 | 309 | 166 | 4 | 7 | 310 | 6 | 307 | 1.34E-81 | 259 |
| **SR-VP_0-2_scaffold_141_5191495_64|SR-VP_0-2cm_Phage_38_25|SR-VP_0-2cm** | BML_coassembly_scaffold_18082_7|BML_coassembly_UNK|BML_coassembly | 42.395 | 309 | 166 | 4 | 7 | 310 | 6 | 307 | 1.34E-81 | 259 |
| **SR-VP_0-2_scaffold_141_5191495_64|SR-VP_0-2cm_Phage_38_25|SR-VP_0-2cm** | GS883_0p1_scaffold_748_8|lsdeep_GS883_0p1_UNK|lsdeep_GS883_0p1 | 43.974 | 307 | 165 | 5 | 6 | 309 | 4 | 306 | 1.39E-81 | 259 |
| **SR-VP_0-2_scaffold_141_5191495_64|SR-VP_0-2cm_Phage_38_25|SR-VP_0-2cm** | BML_coassembly_scaffold_20320_9|BML_coassembly_UNK|BML_coassembly | 42.857 | 315 | 170 | 4 | 2 | 310 | 1 | 311 | 1.46E-81 | 259 |
| **SR-VP_0-2_scaffold_141_5191495_64|SR-VP_0-2cm_Phage_38_25|SR-VP_0-2cm** | FFC_092018_0_1um_scaffold_93_2|FFC_092018_0_1um_UNK|FFC_092018_0_1um | 42.444 | 311 | 174 | 3 | 2 | 311 | 1 | 307 | 1.55E-81 | 259 |
| **SR-VP_0-2_scaffold_141_5191495_63|SR-VP_0-2cm_Phage_38_25|SR-VP_0-2cm** | SR-VP_0-2_scaffold_141_5191495_prodigal-single_70|SR-VP_PHAGE_38_25|SR-VP_0-2cm | 100.000 | 241 | 0 | 0 | 1 | 241 | 1 | 241 | 5.01E-176 | 493 |
| **SR-VP_0-2_scaffold_141_5191495_63|SR-VP_0-2cm_Phage_38_25|SR-VP_0-2cm** | SR-VP_0-2_scaffold_141_5191495_63|SR-VP_0-2cm_Phage_38_25|SR-VP_0-2cm | 100.000 | 241 | 0 | 0 | 1 | 241 | 1 | 241 | 5.01E-176 | 493 |
| **SR-VP_0-2_scaffold_141_5191495_63|SR-VP_0-2cm_Phage_38_25|SR-VP_0-2cm** | SR-VP_4-6_scaffold_141_1637869_1|SR-VP_4-6cm_Biohub_180515_UNK|SR-VP_4-6cm_Biohub_180515 | 100.000 | 176 | 0 | 0 | 66 | 241 | 1 | 176 | 3.77E-126 | 365 |
| **SR-VP_0-2_scaffold_141_5191495_63|SR-VP_0-2cm_Phage_38_25|SR-VP_0-2cm** | SR-VP_0-2_scaffold_141_3175919_5|SR-VP_0-2cm_UNK|SR-VP_0-2cm | 57.971 | 207 | 85 | 1 | 37 | 241 | 1 | 207 | 6.32E-81 | 251 |
| **SR-VP_0-2_scaffold_141_5191495_67|SR-VP_0-2cm_Phage_38_25|SR-VP_0-2cm** | SR-VP_0-2_scaffold_141_5191495_prodigal-single_74|SR-VP_PHAGE_38_25|SR-VP_0-2cm | 100.000 | 773 | 0 | 0 | 1 | 773 | 1 | 773 | 0.0 | 1562 |
| **SR-VP_0-2_scaffold_141_5191495_67|SR-VP_0-2cm_Phage_38_25|SR-VP_0-2cm** | SR-VP_0-2_scaffold_141_5191495_67|SR-VP_0-2cm_Phage_38_25|SR-VP_0-2cm | 100.000 | 773 | 0 | 0 | 1 | 773 | 1 | 773 | 0.0 | 1562 |
| **SR-VP_0-2_scaffold_141_5191495_67|SR-VP_0-2cm_Phage_38_25|SR-VP_0-2cm** | P0_An_pond3_S8_coassembly_k141_1182798_1|P0_An_pond3_S8_coassembly_UNK|E_GD2017-1_P0_An_pond3_S8_Biohub_coassembly | 64.533 | 781 | 244 | 9 | 1 | 773 | 1 | 756 | 0.0 | 992 |
| **SR-VP_0-2_scaffold_141_5191495_67|SR-VP_0-2cm_Phage_38_25|SR-VP_0-2cm** | SR-VP_0-2_scaffold_141_5112071_2|SR-VP_0-2cm_UNK|SR-VP_0-2cm | 58.625 | 771 | 303 | 7 | 1 | 762 | 1 | 764 | 0.0 | 886 |
| **SR-VP_0-2_scaffold_141_5191495_67|SR-VP_0-2cm_Phage_38_25|SR-VP_0-2cm** | PAFVLPS_2018_scaffold_46833_8|viral-cat_UNK|viral-cat | 56.427 | 778 | 322 | 7 | 1 | 769 | 1 | 770 | 0.0 | 882 |
| **SR-VP_0-2_scaffold_141_5191495_67|SR-VP_0-2cm_Phage_38_25|SR-VP_0-2cm** | PAFVLPS_2018_scaffold_386_5|viral-cat_UNK|viral-cat | 56.041 | 778 | 325 | 7 | 1 | 769 | 1 | 770 | 0.0 | 876 |
| **SR-VP_0-2_scaffold_141_5191495_67|SR-VP_0-2cm_Phage_38_25|SR-VP_0-2cm** | SRVP18_trench_1_20cm_scaffold_10130_2|SRVP18_trench_1_20cm_UNK|SRVP18_trench_1_20cm | 54.876 | 769 | 326 | 7 | 3 | 770 | 2 | 750 | 0.0 | 819 |
| **SR-VP_0-2_scaffold_141_5191495_67|SR-VP_0-2cm_Phage_38_25|SR-VP_0-2cm** | SR-VP_0-2_scaffold_141_6448357_8|SR-VP_0-2cm_UNK|SR-VP_0-2cm | 52.895 | 760 | 334 | 5 | 1 | 759 | 1 | 737 | 0.0 | 806 |
| **SR-VP_0-2_scaffold_141_5191495_67|SR-VP_0-2cm_Phage_38_25|SR-VP_0-2cm** | S15_GE15_scaffold_109408_2|E_GD2017-2_urea-2_S15_GE15_Biohub_170515_UNK|E_GD2017-2_urea-2_S15_GE15_Biohub_180515 | 62.715 | 641 | 207 | 8 | 141 | 773 | 1 | 617 | 0.0 | 780 |
| **SR-VP_0-2_scaffold_141_5191495_67|SR-VP_0-2cm_Phage_38_25|SR-VP_0-2cm** | SR-VP_0-2_scaffold_141_6432269_1|SR-VP_0-2cm_UNK|SR-VP_0-2cm | 51.643 | 761 | 346 | 6 | 11 | 770 | 1 | 740 | 0.0 | 766 |
| **SR-VP_0-2_scaffold_141_5191495_67|SR-VP_0-2cm_Phage_38_25|SR-VP_0-2cm** | LacPavin_0419_WC70S_scaffold_436490_11|LacPavin_0419_WC70S_UNK|LacPavin_0419_WC70S | 48.816 | 760 | 364 | 7 | 3 | 760 | 1 | 737 | 0.0 | 730 |
| **SR-VP_0-2_scaffold_141_5191495_67|SR-VP_0-2cm_Phage_38_25|SR-VP_0-2cm** | L3m1_full_idba_ud_scaffold_1665_2|L3m1_UNK|L3m1 | 55.591 | 635 | 276 | 3 | 1 | 632 | 1 | 632 | 0.0 | 707 |
| **SR-VP_0-2_scaffold_141_5191495_67|SR-VP_0-2cm_Phage_38_25|SR-VP_0-2cm** | ERMGT828_2_scaffold_19588_2|ERMGT828_2_UNK|ERMGT828_2 | 61.191 | 554 | 209 | 6 | 3 | 552 | 4 | 555 | 0.0 | 672 |
| **SR-VP_0-2_scaffold_141_5191495_67|SR-VP_0-2cm_Phage_38_25|SR-VP_0-2cm** | PAFVLPS_2018_scaffold_250137_1|viral-cat_UNK|viral-cat | 56.818 | 572 | 242 | 5 | 1 | 570 | 1 | 569 | 0.0 | 647 |
| **SR-VP_0-2_scaffold_141_5191495_67|SR-VP_0-2cm_Phage_38_25|SR-VP_0-2cm** | S16_GE16_scaffold_443094_1|E_GD2017-2_urea-3_S16_GE16_Biohub_170515_UNK|E_GD2017-2_urea-3_S16_GE16_Biohub_180515 | 69.359 | 421 | 119 | 4 | 4 | 419 | 1 | 416 | 0.0 | 583 |
| **SR-VP_0-2_scaffold_141_5191495_67|SR-VP_0-2cm_Phage_38_25|SR-VP_0-2cm** | H1a2_full_idba_ud_scaffold_81450_1|H1a2_UNK|H1a2 | 59.875 | 481 | 191 | 2 | 3 | 481 | 4 | 484 | 0.0 | 583 |
| **SR-VP_0-2_scaffold_141_5191495_67|SR-VP_0-2cm_Phage_38_25|SR-VP_0-2cm** | L3m2_full_idba_ud_scaffold_11238_2|L3m2_concoct_70|L3m2 | 53.179 | 519 | 237 | 3 | 117 | 632 | 1 | 516 | 0.0 | 545 |
| **SR-VP_0-2_scaffold_141_5191495_67|SR-VP_0-2cm_Phage_38_25|SR-VP_0-2cm** | ERMGT157_2_scaffold_16082_2|ERMGT157_2_UNK|ERMGT157_2 | 54.183 | 526 | 236 | 4 | 243 | 767 | 7 | 528 | 0.0 | 538 |
| **SR-VP_0-2_scaffold_141_5191495_67|SR-VP_0-2cm_Phage_38_25|SR-VP_0-2cm** | GWB1_scaffold_1082_4|GWB1 | 44.048 | 672 | 344 | 14 | 3 | 662 | 1 | 652 | 1.07E-173 | 528 |
| **SR-VP_0-2_scaffold_141_5191495_67|SR-VP_0-2cm_Phage_38_25|SR-VP_0-2cm** | P0_An_GD2017L_S7_coassembly_k141_1814973_2|P0_An_GD2017L_S7_coassembly_UNK|E_P0_An_GD2017L_S7_coassembly | 67.467 | 375 | 112 | 4 | 1 | 370 | 1 | 370 | 8.89E-169 | 501 |
| **SR-VP_0-2_scaffold_141_5191495_67|SR-VP_0-2cm_Phage_38_25|SR-VP_0-2cm** | SR-VP_0-2_scaffold_141_2432070_18|SR-VP_0-2cm_UNK|SR-VP_0-2cm | 39.211 | 760 | 415 | 15 | 5 | 752 | 4 | 728 | 1.35E-165 | 507 |
| **SR-VP_0-2_scaffold_141_5191495_67|SR-VP_0-2cm_Phage_38_25|SR-VP_0-2cm** | SR-VP_4-6_scaffold_141_682911_2|SR-VP_4-6cm_Biohub_180515_UNK|SR-VP_4-6cm_Biohub_180515 | 39.211 | 760 | 415 | 15 | 5 | 752 | 4 | 728 | 4.74E-165 | 506 |
| **SR-VP_0-2_scaffold_141_5191495_67|SR-VP_0-2cm_Phage_38_25|SR-VP_0-2cm** | P0_An_GD2017L_S7_coassembly_k141_2601098_1|P0_An_GD2017L_S7_coassembly_UNK|E_P0_An_GD2017L_S7_coassembly | 61.671 | 407 | 133 | 5 | 370 | 773 | 103 | 489 | 5.94E-164 | 493 |
| **SR-VP_0-2_scaffold_141_5191495_67|SR-VP_0-2cm_Phage_38_25|SR-VP_0-2cm** | ERMLT262_2_scaffold_18192_1|ERMLT262_2_UNK|ERMLT262_2 | 61.039 | 385 | 145 | 5 | 109 | 489 | 1 | 384 | 2.31E-155 | 467 |
| **SR-VP_0-2_scaffold_141_5191495_67|SR-VP_0-2cm_Phage_38_25|SR-VP_0-2cm** | SRVP18_trench_5_20cm_scaffold_80670_1|SRVP18_trench_5_20cm_UNK|SRVP18_trench_5_20cm | 55.344 | 421 | 186 | 2 | 71 | 490 | 1 | 420 | 2.32E-153 | 464 |
| **SR-VP_0-2_scaffold_141_5191495_67|SR-VP_0-2cm_Phage_38_25|SR-VP_0-2cm** | PAFVLPS_2018_scaffold_895318_1|viral-cat_UNK|viral-cat | 57.215 | 395 | 156 | 5 | 338 | 724 | 1 | 390 | 4.05E-150 | 454 |
| **SR-VP_0-2_scaffold_141_5191495_67|SR-VP_0-2cm_Phage_38_25|SR-VP_0-2cm** | SR-VP_0-2_scaffold_141_4671315_1|SR-VP_0-2cm_UNK|SR-VP_0-2cm | 40.000 | 630 | 341 | 11 | 62 | 683 | 1 | 601 | 9.08E-150 | 462 |
| **SR-VP_0-2_scaffold_141_5191495_67|SR-VP_0-2cm_Phage_38_25|SR-VP_0-2cm** | ALT_03122018_0_1um_scaffold_34255_1|ALT_03122018_0_1um_UNK|ALT_03122018_0_1um | 55.172 | 435 | 164 | 7 | 326 | 759 | 1 | 405 | 1.4E-149 | 453 |
| **SR-VP_0-2_scaffold_141_5191495_67|SR-VP_0-2cm_Phage_38_25|SR-VP_0-2cm** | PLM4_65_coex_sep16_scaffold_112227_1|PLM4_65cm_coex_sep2016_UNK|PLM4_65cm_coex_sep2016 | 45.290 | 552 | 269 | 7 | 101 | 645 | 1 | 526 | 1.22E-146 | 453 |
| **SR-VP_0-2_scaffold_141_5191495_67|SR-VP_0-2cm_Phage_38_25|SR-VP_0-2cm** | SR-VP_2-4_scaffold_141_646991_1|SR-VP_2-4cm_UNK|SR-VP_2-4cm | 59.827 | 346 | 138 | 1 | 288 | 632 | 2 | 347 | 1.41E-142 | 433 |
| **SR-VP_0-2_scaffold_141_5191495_67|SR-VP_0-2cm_Phage_38_25|SR-VP_0-2cm** | PAFVLPS_2018_J28_2_scaffold_69624_1|soil-virus-P12-J28-2018_UNK|soil-virus-P12-J28-2018 | 56.131 | 367 | 160 | 1 | 99 | 464 | 6 | 372 | 1.72E-140 | 429 |
| **SR-VP_0-2_scaffold_141_5191495_67|SR-VP_0-2cm_Phage_38_25|SR-VP_0-2cm** | ERMGT244_2_scaffold_26701_1|ERMGT244_2_UNK|ERMGT244_2 | 57.865 | 356 | 149 | 1 | 44 | 399 | 1 | 355 | 1.44E-136 | 418 |
| **SR-VP_0-2_scaffold_141_5191495_67|SR-VP_0-2cm_Phage_38_25|SR-VP_0-2cm** | SR-VP_2-4_scaffold_141_8221994_1|SR-VP_2-4cm_UNK|SR-VP_2-4cm | 56.183 | 372 | 161 | 2 | 133 | 503 | 2 | 372 | 3.05E-132 | 407 |
| **SR-VP_0-2_scaffold_141_5191495_67|SR-VP_0-2cm_Phage_38_25|SR-VP_0-2cm** | S16_GE16_scaffold_524391_2|E_GD2017-2_urea-3_S16_GE16_Biohub_170515_UNK|E_GD2017-2_urea-3_S16_GE16_Biohub_180515 | 59.130 | 345 | 118 | 5 | 432 | 773 | 2 | 326 | 2.37E-127 | 393 |
| **SR-VP_0-2_scaffold_141_5191495_67|SR-VP_0-2cm_Phage_38_25|SR-VP_0-2cm** | SR-VP_4-6_scaffold_141_3583822_1|SR-VP_4-6cm_Biohub_180515_UNK|SR-VP_4-6cm_Biohub_180515 | 100.000 | 191 | 0 | 0 | 583 | 773 | 1 | 191 | 1.36E-126 | 386 |
| **SR-VP_0-2_scaffold_141_5191495_67|SR-VP_0-2cm_Phage_38_25|SR-VP_0-2cm** | ERMLT660_2_scaffold_16732_1|ERMLT660_2_UNK|ERMLT660_2 | 46.585 | 410 | 217 | 2 | 115 | 522 | 2 | 411 | 4.39E-122 | 383 |
| **SR-VP_0-2_scaffold_141_5191495_67|SR-VP_0-2cm_Phage_38_25|SR-VP_0-2cm** | L3m1_full_idba_ud_scaffold_79967_2|L3m1_UNK|L3m1 | 55.228 | 373 | 150 | 7 | 406 | 769 | 1 | 365 | 8.32E-122 | 380 |
| **SR-VP_0-2_scaffold_141_5191495_67|SR-VP_0-2cm_Phage_38_25|SR-VP_0-2cm** | LacPavin_0419_WC70S_scaffold_80622_1|LacPavin_0419_WC70S_UNK|LacPavin_0419_WC70S | 50.811 | 370 | 181 | 1 | 202 | 570 | 14 | 383 | 2.03E-120 | 377 |
| **SR-VP_0-2_scaffold_141_5191495_67|SR-VP_0-2cm_Phage_38_25|SR-VP_0-2cm** | PLM4_65_b1_redo_sep16_scaffold_358230_1|PLM4_65cm_b1_redo_sep2016_UNK|PLM4_65cm_b1_redo_sep2016 | 48.622 | 399 | 165 | 6 | 373 | 770 | 1 | 360 | 6.36E-113 | 357 |
| **SR-VP_0-2_scaffold_141_5191495_67|SR-VP_0-2cm_Phage_38_25|SR-VP_0-2cm** | LacPavin_0419_WC70S_scaffold_945204_7|LacPavin_0419_WC70S_UNK|LacPavin_0419_WC70S | 36.526 | 616 | 359 | 9 | 22 | 635 | 20 | 605 | 2.27E-107 | 357 |
| **SR-VP_0-2_scaffold_141_5191495_67|SR-VP_0-2cm_Phage_38_25|SR-VP_0-2cm** | LacPavin_0818_WC55_scaffold_113784_prodigal-single_370|LP_PHAGE_COMPLETE_34_34|LacPavin_0818_WC55 | 36.526 | 616 | 359 | 9 | 22 | 635 | 20 | 605 | 2.27E-107 | 357 |
| **SR-VP_0-2_scaffold_141_5191495_67|SR-VP_0-2cm_Phage_38_25|SR-VP_0-2cm** | LacPavin_0818_WC55_scaffold_113784_359|LacPavin_0818_WC55_Potentially_Complete_Phage_34_34|LacPavin_0818_WC55 | 36.526 | 616 | 359 | 9 | 22 | 635 | 20 | 605 | 2.27E-107 | 357 |
| **SR-VP_0-2_scaffold_141_5191495_67|SR-VP_0-2cm_Phage_38_25|SR-VP_0-2cm** | LacPavin_0818_WC45_scaffold_80267_119|LacPavin_0818_WC45_UNK|LacPavin_0818_WC45 | 36.526 | 616 | 359 | 9 | 22 | 635 | 20 | 605 | 2.27E-107 | 357 |
| **SR-VP_0-2_scaffold_141_5191495_67|SR-VP_0-2cm_Phage_38_25|SR-VP_0-2cm** | LacPavin_0718_WC55_scaffold_0_31|LacPavin_0718_WC55_Phage_34_15|LacPavin_0718_WC55 | 36.526 | 616 | 359 | 9 | 22 | 635 | 20 | 605 | 2.27E-107 | 357 |
| **SR-VP_0-2_scaffold_141_5191495_67|SR-VP_0-2cm_Phage_38_25|SR-VP_0-2cm** | LacPavin_0718_WC45_scaffold_3_119|LacPavin_0718_WC45_UNK|LacPavin_0718_WC45 | 36.526 | 616 | 359 | 9 | 22 | 635 | 20 | 605 | 2.27E-107 | 357 |
| **SR-VP_0-2_scaffold_141_5191495_67|SR-VP_0-2cm_Phage_38_25|SR-VP_0-2cm** | ERMGT828_2_scaffold_50788_1|ERMGT828_2_UNK|ERMGT828_2 | 46.610 | 354 | 188 | 1 | 70 | 422 | 1 | 354 | 9.78E-106 | 338 |
| **SR-VP_0-2_scaffold_141_5191495_67|SR-VP_0-2cm_Phage_38_25|SR-VP_0-2cm** | BC_09192017_0_5m_scaffold_7473_2|BC_09192017_0_5m_UNK|BC_09192017_0_5m | 36.447 | 653 | 372 | 16 | 11 | 654 | 5 | 623 | 3.56E-103 | 351 |
| **SR-VP_0-2_scaffold_141_5191495_67|SR-VP_0-2cm_Phage_38_25|SR-VP_0-2cm** | AB_082018_0_1um_scaffold_2553_4|AB_082018_0_1um_UNK|AB_082018_0_1um | 36.177 | 633 | 364 | 15 | 11 | 634 | 5 | 606 | 8.83E-102 | 343 |
| **SR-VP_0-2_scaffold_141_5191495_67|SR-VP_0-2cm_Phage_38_25|SR-VP_0-2cm** | LacPavin_0419_WC53_scaffold_515305_2|LacPavin_0419_WC53_UNK|LacPavin_0419_WC53 | 36.133 | 631 | 363 | 15 | 11 | 632 | 5 | 604 | 1.49E-100 | 344 |
| **SR-VP_0-2_scaffold_141_5191495_67|SR-VP_0-2cm_Phage_38_25|SR-VP_0-2cm** | PAFVLPS_2018_scaffold_18_355|viral-cat_UNK|viral-cat | 39.121 | 478 | 277 | 8 | 163 | 630 | 325 | 798 | 6.6E-99 | 337 |
| **SR-VP_0-2_scaffold_141_5191495_67|SR-VP_0-2cm_Phage_38_25|SR-VP_0-2cm** | PAFVLPS_2018_scaffold_18_355|viral-cat_UNK|viral-cat | 47.853 | 163 | 80 | 1 | 3 | 165 | 1 | 158 | 3.95E-36 | 159 |
| **SR-VP_0-2_scaffold_141_5191495_67|SR-VP_0-2cm_Phage_38_25|SR-VP_0-2cm** | PAFVLPS_2018_J30_2_scaffold_1899_4|soil-virus-P14-J30-2018_UNK|soil-virus-P14-J30-2018 | 39.121 | 478 | 277 | 8 | 163 | 630 | 325 | 798 | 6.6E-99 | 337 |
| **SR-VP_0-2_scaffold_141_5191495_67|SR-VP_0-2cm_Phage_38_25|SR-VP_0-2cm** | PAFVLPS_2018_J30_2_scaffold_1899_4|soil-virus-P14-J30-2018_UNK|soil-virus-P14-J30-2018 | 47.853 | 163 | 80 | 1 | 3 | 165 | 1 | 158 | 3.95E-36 | 159 |
| **SR-VP_0-2_scaffold_141_5191495_67|SR-VP_0-2cm_Phage_38_25|SR-VP_0-2cm** | BML_coassembly_scaffold_632_29|BML_coassembly_UNK|BML_coassembly | 34.046 | 655 | 387 | 12 | 10 | 635 | 5 | 643 | 9.61E-99 | 334 |
| **SR-VP_0-2_scaffold_141_5191495_67|SR-VP_0-2cm_Phage_38_25|SR-VP_0-2cm** | FFC_04162018_0_1um_scaffold_1491_2|FFC_04162018_0_1um_UNK|FFC_04162018_0_1um | 36.037 | 641 | 370 | 15 | 1 | 632 | 1042 | 1651 | 1.04E-98 | 347 |
| **SR-VP_0-2_scaffold_141_5191495_67|SR-VP_0-2cm_Phage_38_25|SR-VP_0-2cm** | PAFVLPS_2018_J28_2_scaffold_23939_3|soil-virus-P12-J28-2018_UNK|soil-virus-P12-J28-2018 | 53.548 | 310 | 128 | 6 | 468 | 769 | 1 | 302 | 3.25E-98 | 317 |
| **SR-VP_0-2_scaffold_141_5191495_67|SR-VP_0-2cm_Phage_38_25|SR-VP_0-2cm** | PAFVLPS_2018_scaffold_881662_1|viral-cat_UNK|viral-cat | 44.612 | 399 | 207 | 7 | 94 | 485 | 7 | 398 | 1.87E-95 | 313 |
| **SR-VP_0-2_scaffold_141_5191495_67|SR-VP_0-2cm_Phage_38_25|SR-VP_0-2cm** | SR-VP_0-2_scaffold_141_6993612_1|SR-VP_0-2cm_UNK|SR-VP_0-2cm | 54.248 | 306 | 138 | 2 | 1 | 306 | 1 | 304 | 1.55E-93 | 305 |
| **SR-VP_0-2_scaffold_141_5191495_67|SR-VP_0-2cm_Phage_38_25|SR-VP_0-2cm** | S15_GE15_scaffold_39268_57|E_GD2017-2_urea-2_S15_GE15_Biohub_170515_UNK|E_GD2017-2_urea-2_S15_GE15_Biohub_180515 | 38.242 | 455 | 265 | 9 | 187 | 630 | 351 | 800 | 1.04E-91 | 318 |
| **SR-VP_0-2_scaffold_141_5191495_67|SR-VP_0-2cm_Phage_38_25|SR-VP_0-2cm** | S15_GE15_scaffold_39268_57|E_GD2017-2_urea-2_S15_GE15_Biohub_170515_UNK|E_GD2017-2_urea-2_S15_GE15_Biohub_180515 | 43.373 | 166 | 89 | 1 | 3 | 168 | 1 | 161 | 6.33E-33 | 149 |
| **SR-VP_0-2_scaffold_141_5191495_67|SR-VP_0-2cm_Phage_38_25|SR-VP_0-2cm** | P0_An_pond3_S8_coassembly_k141_1206453_2|P0_An_pond3_S8_coassembly_UNK|E_GD2017-1_P0_An_pond3_S8_Biohub_coassembly | 38.242 | 455 | 265 | 9 | 187 | 630 | 351 | 800 | 1.04E-91 | 318 |
| **SR-VP_0-2_scaffold_141_5191495_67|SR-VP_0-2cm_Phage_38_25|SR-VP_0-2cm** | P0_An_pond3_S8_coassembly_k141_1206453_2|P0_An_pond3_S8_coassembly_UNK|E_GD2017-1_P0_An_pond3_S8_Biohub_coassembly | 43.373 | 166 | 89 | 1 | 3 | 168 | 1 | 161 | 6.33E-33 | 149 |
| **SR-VP_0-2_scaffold_141_5191495_67|SR-VP_0-2cm_Phage_38_25|SR-VP_0-2cm** | ALT_072018_0_1um_scaffold_43809_2|ALT_072018_0_1um_UNK|ALT_072018_0_1um | 59.341 | 273 | 101 | 4 | 457 | 722 | 2 | 271 | 1.34E-91 | 300 |
| **SR-VP_0-2_scaffold_141_5191495_67|SR-VP_0-2cm_Phage_38_25|SR-VP_0-2cm** | PAFVLPS_2018_scaffold_33_43|circular_33|viral-cat | 38.444 | 450 | 267 | 8 | 187 | 630 | 348 | 793 | 2.67E-91 | 317 |
| **SR-VP_0-2_scaffold_141_5191495_67|SR-VP_0-2cm_Phage_38_25|SR-VP_0-2cm** | PAFVLPS_2018_scaffold_33_43|circular_33|viral-cat | 46.061 | 165 | 84 | 1 | 3 | 167 | 1 | 160 | 1.6E-36 | 160 |
| **SR-VP_0-2_scaffold_141_5191495_67|SR-VP_0-2cm_Phage_38_25|SR-VP_0-2cm** | S16_GE16_scaffold_5545_prodigal-single_29|GD_PHAGE_46_9|E_GD2017-2_urea-3_S16_GE16_Biohub_180515 | 38.022 | 455 | 266 | 9 | 187 | 630 | 351 | 800 | 4.23E-91 | 317 |
| **SR-VP_0-2_scaffold_141_5191495_67|SR-VP_0-2cm_Phage_38_25|SR-VP_0-2cm** | S16_GE16_scaffold_5545_prodigal-single_29|GD_PHAGE_46_9|E_GD2017-2_urea-3_S16_GE16_Biohub_180515 | 43.373 | 166 | 89 | 1 | 3 | 168 | 1 | 161 | 6.33E-33 | 149 |
| **SR-VP_0-2_scaffold_141_5191495_67|SR-VP_0-2cm_Phage_38_25|SR-VP_0-2cm** | S16_GE16_scaffold_5545_28|E_GD2017-2_urea-3_S16_GE16_Biohub_170515_Phage-like_46_9|E_GD2017-2_urea-3_S16_GE16_Biohub_180515 | 38.022 | 455 | 266 | 9 | 187 | 630 | 351 | 800 | 4.23E-91 | 317 |
| **SR-VP_0-2_scaffold_141_5191495_67|SR-VP_0-2cm_Phage_38_25|SR-VP_0-2cm** | S16_GE16_scaffold_5545_28|E_GD2017-2_urea-3_S16_GE16_Biohub_170515_Phage-like_46_9|E_GD2017-2_urea-3_S16_GE16_Biohub_180515 | 43.373 | 166 | 89 | 1 | 3 | 168 | 1 | 161 | 6.33E-33 | 149 |
| **SR-VP_0-2_scaffold_141_5191495_67|SR-VP_0-2cm_Phage_38_25|SR-VP_0-2cm** | PLM4_65_b1_redo_sep16_scaffold_358230_2|PLM4_65cm_b1_redo_sep2016_UNK|PLM4_65cm_b1_redo_sep2016 | 59.016 | 244 | 100 | 0 | 1 | 244 | 1 | 244 | 1.14E-90 | 296 |
| **SR-VP_0-2_scaffold_141_5191495_67|SR-VP_0-2cm_Phage_38_25|SR-VP_0-2cm** | SR-VP_4-6_scaffold_141_6282548_1|SR-VP_4-6cm_Biohub_180515_UNK|SR-VP_4-6cm_Biohub_180515 | 58.130 | 246 | 103 | 0 | 1 | 246 | 1 | 246 | 1.19E-88 | 290 |
| **SR-VP_0-2_scaffold_141_5191495_67|SR-VP_0-2cm_Phage_38_25|SR-VP_0-2cm** | L3a1_full_idba_ud_scaffold_6_230|L3a1_UNK|L3a1 | 35.824 | 522 | 309 | 11 | 121 | 632 | 297 | 802 | 9.69E-88 | 308 |
| **SR-VP_0-2_scaffold_141_5191495_67|SR-VP_0-2cm_Phage_38_25|SR-VP_0-2cm** | L3a1_full_idba_ud_scaffold_6_230|L3a1_UNK|L3a1 | 46.386 | 166 | 83 | 2 | 1 | 165 | 1 | 161 | 1.3E-35 | 157 |
| **SR-VP_0-2_scaffold_141_5191495_67|SR-VP_0-2cm_Phage_38_25|SR-VP_0-2cm** | ALT_03122018_0_1um_scaffold_15827_1|ALT_03122018_0_1um_UNK|ALT_03122018_0_1um | 57.377 | 244 | 104 | 0 | 1 | 244 | 1 | 244 | 2.53E-86 | 284 |
| **SR-VP_0-2_scaffold_141_5191495_67|SR-VP_0-2cm_Phage_38_25|SR-VP_0-2cm** | PAFVLPS_2018_scaffold_187463_1|viral-cat_UNK|viral-cat | 34.237 | 590 | 343 | 14 | 187 | 764 | 344 | 900 | 8.59E-85 | 299 |
| **SR-VP_0-2_scaffold_141_5191495_67|SR-VP_0-2cm_Phage_38_25|SR-VP_0-2cm** | PAFVLPS_2018_scaffold_187463_1|viral-cat_UNK|viral-cat | 46.061 | 165 | 84 | 1 | 3 | 167 | 1 | 160 | 1.83E-35 | 157 |
| **SR-VP_0-2_scaffold_141_5191495_67|SR-VP_0-2cm_Phage_38_25|SR-VP_0-2cm** | LacPavin_0818_WC45_scaffold_305942_32|LacPavin_0818_WC45_UNK|LacPavin_0818_WC45 | 33.082 | 662 | 403 | 15 | 11 | 660 | 5 | 638 | 1.02E-84 | 297 |
| **SR-VP_0-2_scaffold_141_5191495_67|SR-VP_0-2cm_Phage_38_25|SR-VP_0-2cm** | PAFVLPS_2018_scaffold_187487_1|viral-cat_UNK|viral-cat | 33.441 | 619 | 362 | 15 | 163 | 764 | 315 | 900 | 3.8E-84 | 297 |
| **SR-VP_0-2_scaffold_141_5191495_67|SR-VP_0-2cm_Phage_38_25|SR-VP_0-2cm** | PAFVLPS_2018_scaffold_187487_1|viral-cat_UNK|viral-cat | 44.848 | 165 | 86 | 1 | 3 | 167 | 1 | 160 | 1.99E-34 | 154 |
| **SR-VP_0-2_scaffold_141_5191495_67|SR-VP_0-2cm_Phage_38_25|SR-VP_0-2cm** | Mad1_40_16_scaffold_197295_1|Mad1_40_16_UNK|Mad1_40_16 | 53.307 | 257 | 119 | 1 | 292 | 547 | 229 | 485 | 1.06E-82 | 283 |
| **SR-VP_0-2_scaffold_141_5191495_67|SR-VP_0-2cm_Phage_38_25|SR-VP_0-2cm** | Mad1_40_16_scaffold_197295_1|Mad1_40_16_UNK|Mad1_40_16 | 59.545 | 220 | 89 | 0 | 3 | 222 | 2 | 221 | 2.14E-76 | 266 |
| **SR-VP_0-2_scaffold_141_5191495_67|SR-VP_0-2cm_Phage_38_25|SR-VP_0-2cm** | LacPavin_0818_WC40_scaffold_875999_59|LacPavin_0818_WC40_UNK|LacPavin_0818_WC40 | 33.225 | 617 | 355 | 15 | 11 | 627 | 3 | 562 | 1.31E-81 | 285 |
| **SR-VP_0-2_scaffold_141_5191495_67|SR-VP_0-2cm_Phage_38_25|SR-VP_0-2cm** | PAFVLPS_2018_scaffold_577014_1|viral-cat_UNK|viral-cat | 37.130 | 439 | 264 | 8 | 198 | 630 | 3 | 435 | 2.25E-80 | 278 |
| **SR-VP_0-2_scaffold_141_5191495_67|SR-VP_0-2cm_Phage_38_25|SR-VP_0-2cm** | P0_An_pond3_S8_coassembly_k141_1874539_1|P0_An_pond3_S8_coassembly_UNK|E_GD2017-1_P0_An_pond3_S8_Biohub_coassembly | 60.870 | 207 | 81 | 0 | 1 | 207 | 1 | 207 | 8.9E-79 | 262 |
| **SR-VP_0-2_scaffold_141_5191495_67|SR-VP_0-2cm_Phage_38_25|SR-VP_0-2cm** | ERMGT138_2_scaffold_32894_2|ERMGT138_2_UNK|ERMGT138_2 | 42.623 | 305 | 166 | 4 | 3 | 303 | 9 | 308 | 1.61E-77 | 263 |
| **SR-VP_0-2_scaffold_141_5191495_67|SR-VP_0-2cm_Phage_38_25|SR-VP_0-2cm** | U2s1_full_idba_ud_scaffold_45754_1|U2s1_UNK|U2s1 | 39.628 | 376 | 216 | 5 | 179 | 546 | 1 | 373 | 3.06E-76 | 262 |
| **SR-VP_0-2_scaffold_141_5191495_67|SR-VP_0-2cm_Phage_38_25|SR-VP_0-2cm** | FFC_07242016_10_scaffold_18482_1|FFC_07242016_10_UNK|FFC_07242016_10 | 38.162 | 359 | 204 | 5 | 17 | 368 | 4 | 351 | 6.36E-72 | 249 |
| **SR-VP_0-2_scaffold_141_5191495_67|SR-VP_0-2cm_Phage_38_25|SR-VP_0-2cm** | PAFVLPS_2018_scaffold_693522_2|viral-cat_UNK|viral-cat | 70.552 | 163 | 48 | 0 | 1 | 163 | 1 | 163 | 6.79E-70 | 237 |
| **SR-VP_0-2_scaffold_141_5191495_67|SR-VP_0-2cm_Phage_38_25|SR-VP_0-2cm** | LacPavin_0419_WC70S_scaffold_94267_2|LacPavin_0419_WC70S_UNK|LacPavin_0419_WC70S | 38.028 | 355 | 193 | 7 | 4 | 346 | 3 | 342 | 2.17E-69 | 243 |
| **SR-VP_0-2_scaffold_141_5191495_67|SR-VP_0-2cm_Phage_38_25|SR-VP_0-2cm** | GD18-4_manure_scaffold_687770_5|GD2018-4_manure_QB3_180703_UNK|GD2018-4_manure_QB3_180703 | 34.725 | 455 | 248 | 12 | 325 | 760 | 4 | 428 | 9.02E-69 | 244 |
| **SR-VP_0-2_scaffold_141_5191495_67|SR-VP_0-2cm_Phage_38_25|SR-VP_0-2cm** | P0_An_GD2017L_S7_coassembly_k141_1013362_prodigal-single_78|GD_PHAGE_48_49|E_P0_An_GD2017L_S7_coassembly | 34.269 | 499 | 288 | 14 | 277 | 760 | 396 | 869 | 1.37E-67 | 251 |
| **SR-VP_0-2_scaffold_141_5191495_67|SR-VP_0-2cm_Phage_38_25|SR-VP_0-2cm** | P0_An_GD2017L_S7_coassembly_k141_1013362_prodigal-single_78|GD_PHAGE_48_49|E_P0_An_GD2017L_S7_coassembly | 36.508 | 252 | 135 | 6 | 1 | 238 | 1 | 241 | 2.71E-31 | 144 |
| **SR-VP_0-2_scaffold_141_5191495_67|SR-VP_0-2cm_Phage_38_25|SR-VP_0-2cm** | P0_An_GD2017L_S7_coassembly_k141_1013362_72|P0_An_GD2017L_S7_coassembly_Phage_48_49|E_P0_An_GD2017L_S7_coassembly | 34.269 | 499 | 288 | 14 | 277 | 760 | 396 | 869 | 1.37E-67 | 251 |
| **SR-VP_0-2_scaffold_141_5191495_67|SR-VP_0-2cm_Phage_38_25|SR-VP_0-2cm** | P0_An_GD2017L_S7_coassembly_k141_1013362_72|P0_An_GD2017L_S7_coassembly_Phage_48_49|E_P0_An_GD2017L_S7_coassembly | 36.508 | 252 | 135 | 6 | 1 | 238 | 1 | 241 | 2.71E-31 | 144 |
| **SR-VP_0-2_scaffold_141_5191495_67|SR-VP_0-2cm_Phage_38_25|SR-VP_0-2cm** | S30_BME30_294872_46|BM_2017_Coates_4_Potentially_Complete_Phage_48-32|BM_2017_Coates_4 | 34.068 | 499 | 289 | 14 | 277 | 760 | 396 | 869 | 2.91E-67 | 250 |
| **SR-VP_0-2_scaffold_141_5191495_67|SR-VP_0-2cm_Phage_38_25|SR-VP_0-2cm** | S30_BME30_294872_46|BM_2017_Coates_4_Potentially_Complete_Phage_48-32|BM_2017_Coates_4 | 36.508 | 252 | 135 | 6 | 1 | 238 | 1 | 241 | 2.75E-31 | 144 |
| **SR-VP_0-2_scaffold_141_5191495_67|SR-VP_0-2cm_Phage_38_25|SR-VP_0-2cm** | S28_BME28_138683_399|BM_2017_Strous_8_UNK|BM_2017_Strous_8 | 34.068 | 499 | 289 | 14 | 277 | 760 | 396 | 869 | 2.91E-67 | 250 |
| **SR-VP_0-2_scaffold_141_5191495_67|SR-VP_0-2cm_Phage_38_25|SR-VP_0-2cm** | S28_BME28_138683_399|BM_2017_Strous_8_UNK|BM_2017_Strous_8 | 36.508 | 252 | 135 | 6 | 1 | 238 | 1 | 241 | 2.75E-31 | 144 |
| **SR-VP_0-2_scaffold_141_5191495_67|SR-VP_0-2cm_Phage_38_25|SR-VP_0-2cm** | S27_BME27_629333_prodigal-single_183|BM_PHAGE_48_13|BM_2017_Strous_6 | 34.068 | 499 | 289 | 14 | 277 | 760 | 396 | 869 | 2.91E-67 | 250 |
| **SR-VP_0-2_scaffold_141_5191495_67|SR-VP_0-2cm_Phage_38_25|SR-VP_0-2cm** | S27_BME27_629333_prodigal-single_183|BM_PHAGE_48_13|BM_2017_Strous_6 | 36.508 | 252 | 135 | 6 | 1 | 238 | 1 | 241 | 2.75E-31 | 144 |
| **SR-VP_0-2_scaffold_141_5191495_67|SR-VP_0-2cm_Phage_38_25|SR-VP_0-2cm** | S27_BME27_629333_173|BM_2017_Strous_6_Phage_48_13|BM_2017_Strous_6 | 34.068 | 499 | 289 | 14 | 277 | 760 | 396 | 869 | 2.91E-67 | 250 |
| **SR-VP_0-2_scaffold_141_5191495_67|SR-VP_0-2cm_Phage_38_25|SR-VP_0-2cm** | S27_BME27_629333_173|BM_2017_Strous_6_Phage_48_13|BM_2017_Strous_6 | 36.508 | 252 | 135 | 6 | 1 | 238 | 1 | 241 | 2.75E-31 | 144 |
| **SR-VP_0-2_scaffold_141_5191495_67|SR-VP_0-2cm_Phage_38_25|SR-VP_0-2cm** | S33_3P_scaffold_263290_6|Genasci_Feb2018_S33_3P_UNK|Genasci_Feb2018_S33_3P | 34.068 | 499 | 289 | 14 | 277 | 760 | 396 | 869 | 2.91E-67 | 250 |
| **SR-VP_0-2_scaffold_141_5191495_67|SR-VP_0-2cm_Phage_38_25|SR-VP_0-2cm** | S33_3P_scaffold_263290_6|Genasci_Feb2018_S33_3P_UNK|Genasci_Feb2018_S33_3P | 36.508 | 252 | 135 | 6 | 1 | 238 | 1 | 241 | 2.75E-31 | 144 |
| **SR-VP_0-2_scaffold_141_5191495_67|SR-VP_0-2cm_Phage_38_25|SR-VP_0-2cm** | S22_GE22_scaffold_285059_prodigal-single_516|GD_PHAGE_COMPLETE_48_49|E_GD2017-2_anammox-7_S22_GE22_Biohub_180515 | 34.068 | 499 | 289 | 14 | 277 | 760 | 396 | 869 | 2.91E-67 | 250 |
| **SR-VP_0-2_scaffold_141_5191495_67|SR-VP_0-2cm_Phage_38_25|SR-VP_0-2cm** | S22_GE22_scaffold_285059_prodigal-single_516|GD_PHAGE_COMPLETE_48_49|E_GD2017-2_anammox-7_S22_GE22_Biohub_180515 | 36.508 | 252 | 135 | 6 | 1 | 238 | 1 | 241 | 2.75E-31 | 144 |
| **SR-VP_0-2_scaffold_141_5191495_67|SR-VP_0-2cm_Phage_38_25|SR-VP_0-2cm** | S22_GE22_scaffold_285059_439|E_GD2017-2_anammox-7_S22_GE22_Biohub_170515_Potentially_Complete_48_49|E_GD2017-2_anammox-7_S22_GE22_Biohub_180515 | 34.068 | 499 | 289 | 14 | 277 | 760 | 396 | 869 | 2.91E-67 | 250 |
| **SR-VP_0-2_scaffold_141_5191495_67|SR-VP_0-2cm_Phage_38_25|SR-VP_0-2cm** | S22_GE22_scaffold_285059_439|E_GD2017-2_anammox-7_S22_GE22_Biohub_170515_Potentially_Complete_48_49|E_GD2017-2_anammox-7_S22_GE22_Biohub_180515 | 36.508 | 252 | 135 | 6 | 1 | 238 | 1 | 241 | 2.75E-31 | 144 |
| **SR-VP_0-2_scaffold_141_5191495_67|SR-VP_0-2cm_Phage_38_25|SR-VP_0-2cm** | S20_GE20_scaffold_396645_15|E_GD2017-2_strous-5_S20_GE20_Biohub_170515_UNK|E_GD2017-2_strous-5_S20_GE20_Biohub_180515 | 34.068 | 499 | 289 | 14 | 277 | 760 | 396 | 869 | 2.91E-67 | 250 |
| **SR-VP_0-2_scaffold_141_5191495_67|SR-VP_0-2cm_Phage_38_25|SR-VP_0-2cm** | S20_GE20_scaffold_396645_15|E_GD2017-2_strous-5_S20_GE20_Biohub_170515_UNK|E_GD2017-2_strous-5_S20_GE20_Biohub_180515 | 36.508 | 252 | 135 | 6 | 1 | 238 | 1 | 241 | 2.75E-31 | 144 |
| **SR-VP_0-2_scaffold_141_5191495_67|SR-VP_0-2cm_Phage_38_25|SR-VP_0-2cm** | S15_GE15_scaffold_320047_241|E_GD2017-2_urea-2_S15_GE15_Biohub_170515_UNK|E_GD2017-2_urea-2_S15_GE15_Biohub_180515 | 34.068 | 499 | 289 | 14 | 277 | 760 | 396 | 869 | 2.91E-67 | 250 |
| **SR-VP_0-2_scaffold_141_5191495_67|SR-VP_0-2cm_Phage_38_25|SR-VP_0-2cm** | S15_GE15_scaffold_320047_241|E_GD2017-2_urea-2_S15_GE15_Biohub_170515_UNK|E_GD2017-2_urea-2_S15_GE15_Biohub_180515 | 36.508 | 252 | 135 | 6 | 1 | 238 | 1 | 241 | 2.75E-31 | 144 |
| **SR-VP_0-2_scaffold_141_5191495_67|SR-VP_0-2cm_Phage_38_25|SR-VP_0-2cm** | S14_GE14_scaffold_311130_11|E_GD2017-2_urea-2_S14_GE14_Biohub_170515_UNK|E_GD2017-2_urea-2_S14_GE14_Biohub_180515 | 34.068 | 499 | 289 | 14 | 277 | 760 | 396 | 869 | 2.91E-67 | 250 |
| **SR-VP_0-2_scaffold_141_5191495_67|SR-VP_0-2cm_Phage_38_25|SR-VP_0-2cm** | S14_GE14_scaffold_311130_11|E_GD2017-2_urea-2_S14_GE14_Biohub_170515_UNK|E_GD2017-2_urea-2_S14_GE14_Biohub_180515 | 36.508 | 252 | 135 | 6 | 1 | 238 | 1 | 241 | 2.75E-31 | 144 |
| **SR-VP_0-2_scaffold_141_5191495_67|SR-VP_0-2cm_Phage_38_25|SR-VP_0-2cm** | P0_An_pond3_S8_coassembly_k141_2723617_432|P0_An_pond3_S8_coassembly_UNK|E_GD2017-1_P0_An_pond3_S8_Biohub_coassembly | 34.068 | 499 | 289 | 14 | 277 | 760 | 396 | 869 | 2.91E-67 | 250 |
| **SR-VP_0-2_scaffold_141_5191495_67|SR-VP_0-2cm_Phage_38_25|SR-VP_0-2cm** | P0_An_pond3_S8_coassembly_k141_2723617_432|P0_An_pond3_S8_coassembly_UNK|E_GD2017-1_P0_An_pond3_S8_Biohub_coassembly | 36.508 | 252 | 135 | 6 | 1 | 238 | 1 | 241 | 2.75E-31 | 144 |
| **SR-VP_0-2_scaffold_141_5191495_67|SR-VP_0-2cm_Phage_38_25|SR-VP_0-2cm** | S19_GE19_scaffold_2259_prodigal-single_279|GD_PHAGE_48_10|E_GD2017-2_strous-5-pellet_S19_GE19_Biohub_180515 | 34.068 | 499 | 289 | 14 | 277 | 760 | 396 | 869 | 2.91E-67 | 250 |
| **SR-VP_0-2_scaffold_141_5191495_67|SR-VP_0-2cm_Phage_38_25|SR-VP_0-2cm** | S19_GE19_scaffold_2259_prodigal-single_279|GD_PHAGE_48_10|E_GD2017-2_strous-5-pellet_S19_GE19_Biohub_180515 | 36.508 | 252 | 135 | 6 | 1 | 238 | 1 | 241 | 2.75E-31 | 144 |
| **SR-VP_0-2_scaffold_141_5191495_67|SR-VP_0-2cm_Phage_38_25|SR-VP_0-2cm** | S19_GE19_scaffold_2259_255|E_GD2017-2_strous-5-pellet_S19_GE19_Biohub_170515_Phage_48_10|E_GD2017-2_strous-5-pellet_S19_GE19_Biohub_180515 | 34.068 | 499 | 289 | 14 | 277 | 760 | 396 | 869 | 2.91E-67 | 250 |
| **SR-VP_0-2_scaffold_141_5191495_67|SR-VP_0-2cm_Phage_38_25|SR-VP_0-2cm** | S19_GE19_scaffold_2259_255|E_GD2017-2_strous-5-pellet_S19_GE19_Biohub_170515_Phage_48_10|E_GD2017-2_strous-5-pellet_S19_GE19_Biohub_180515 | 36.508 | 252 | 135 | 6 | 1 | 238 | 1 | 241 | 2.75E-31 | 144 |
| **SR-VP_0-2_scaffold_141_5191495_67|SR-VP_0-2cm_Phage_38_25|SR-VP_0-2cm** | S18_GE18_scaffold_616449_286|E_GD2017-2_strous-5-prefilter_S18_GE18_Biohub_170515_UNK|E_GD2017-2_strous-5-prefilter_S18_GE18_Biohub_180515 | 34.068 | 499 | 289 | 14 | 277 | 760 | 396 | 869 | 2.91E-67 | 250 |
| **SR-VP_0-2_scaffold_141_5191495_67|SR-VP_0-2cm_Phage_38_25|SR-VP_0-2cm** | S18_GE18_scaffold_616449_286|E_GD2017-2_strous-5-prefilter_S18_GE18_Biohub_170515_UNK|E_GD2017-2_strous-5-prefilter_S18_GE18_Biohub_180515 | 36.508 | 252 | 135 | 6 | 1 | 238 | 1 | 241 | 2.75E-31 | 144 |
| **SR-VP_0-2_scaffold_141_5191495_67|SR-VP_0-2cm_Phage_38_25|SR-VP_0-2cm** | S16_GE16_scaffold_390213_392|E_GD2017-2_urea-3_S16_GE16_Biohub_170515_UNK|E_GD2017-2_urea-3_S16_GE16_Biohub_180515 | 34.068 | 499 | 289 | 14 | 277 | 760 | 396 | 869 | 2.91E-67 | 250 |
| **SR-VP_0-2_scaffold_141_5191495_67|SR-VP_0-2cm_Phage_38_25|SR-VP_0-2cm** | S16_GE16_scaffold_390213_392|E_GD2017-2_urea-3_S16_GE16_Biohub_170515_UNK|E_GD2017-2_urea-3_S16_GE16_Biohub_180515 | 36.508 | 252 | 135 | 6 | 1 | 238 | 1 | 241 | 2.75E-31 | 144 |
| **SR-VP_0-2_scaffold_141_5191495_67|SR-VP_0-2cm_Phage_38_25|SR-VP_0-2cm** | P0_An_pond3_S8_170907_scaffold_1005865_43|E_GD2017-1_P0_AN_POND3_S8_BIOHUB_170907_UNK|E_GD2017-1_P0_An_pond3_S8_Biohub_170907 | 34.068 | 499 | 289 | 14 | 277 | 760 | 396 | 869 | 2.91E-67 | 250 |
| **SR-VP_0-2_scaffold_141_5191495_67|SR-VP_0-2cm_Phage_38_25|SR-VP_0-2cm** | P0_An_pond3_S8_170907_scaffold_1005865_43|E_GD2017-1_P0_AN_POND3_S8_BIOHUB_170907_UNK|E_GD2017-1_P0_An_pond3_S8_Biohub_170907 | 36.508 | 252 | 135 | 6 | 1 | 238 | 1 | 241 | 2.75E-31 | 144 |
| **SR-VP_0-2_scaffold_141_5191495_67|SR-VP_0-2cm_Phage_38_25|SR-VP_0-2cm** | P0_An_GD2017L_S7_170907_scaffold_359857_26|E_GD2017-1_P0_An_GD2017L_S7_Biohub_170907_UNK|E_GD2017-1_P0_An_GD2017L_S7_Biohub_170907 | 34.068 | 499 | 289 | 14 | 277 | 760 | 396 | 869 | 2.91E-67 | 250 |
| **SR-VP_0-2_scaffold_141_5191495_67|SR-VP_0-2cm_Phage_38_25|SR-VP_0-2cm** | P0_An_GD2017L_S7_170907_scaffold_359857_26|E_GD2017-1_P0_An_GD2017L_S7_Biohub_170907_UNK|E_GD2017-1_P0_An_GD2017L_S7_Biohub_170907 | 36.508 | 252 | 135 | 6 | 1 | 238 | 1 | 241 | 2.75E-31 | 144 |
| **SR-VP_0-2_scaffold_141_5191495_67|SR-VP_0-2cm_Phage_38_25|SR-VP_0-2cm** | S2_GD2017_2_manure_scaffold_3_prodigal-single_484|GD_PHAGE_COMPLETE_48_40|GD2017-2_manure_QB3_180125 | 34.068 | 499 | 289 | 14 | 277 | 760 | 396 | 869 | 2.91E-67 | 250 |
| **SR-VP_0-2_scaffold_141_5191495_67|SR-VP_0-2cm_Phage_38_25|SR-VP_0-2cm** | S2_GD2017_2_manure_scaffold_3_prodigal-single_484|GD_PHAGE_COMPLETE_48_40|GD2017-2_manure_QB3_180125 | 36.508 | 252 | 135 | 6 | 1 | 238 | 1 | 241 | 2.75E-31 | 144 |
| **SR-VP_0-2_scaffold_141_5191495_67|SR-VP_0-2cm_Phage_38_25|SR-VP_0-2cm** | S2_GD2017_2_manure_scaffold_3_411|GD2017-2_manure_QB3_180125_Potentially_complete_Phage_48_40|GD2017-2_manure_QB3_180125 | 34.068 | 499 | 289 | 14 | 277 | 760 | 396 | 869 | 2.91E-67 | 250 |
| **SR-VP_0-2_scaffold_141_5191495_67|SR-VP_0-2cm_Phage_38_25|SR-VP_0-2cm** | S2_GD2017_2_manure_scaffold_3_411|GD2017-2_manure_QB3_180125_Potentially_complete_Phage_48_40|GD2017-2_manure_QB3_180125 | 36.508 | 252 | 135 | 6 | 1 | 238 | 1 | 241 | 2.75E-31 | 144 |
| **SR-VP_0-2_scaffold_141_5191495_68|SR-VP_0-2cm_Phage_38_25|SR-VP_0-2cm** | SR-VP_4-6_scaffold_141_3583822_2|SR-VP_4-6cm_Biohub_180515_UNK|SR-VP_4-6cm_Biohub_180515 | 100.000 | 946 | 0 | 0 | 1 | 946 | 1 | 946 | 0.0 | 1932 |
| **SR-VP_0-2_scaffold_141_5191495_68|SR-VP_0-2cm_Phage_38_25|SR-VP_0-2cm** | SR-VP_0-2_scaffold_141_5191495_prodigal-single_75|SR-VP_PHAGE_38_25|SR-VP_0-2cm | 100.000 | 946 | 0 | 0 | 1 | 946 | 1 | 946 | 0.0 | 1932 |
| **SR-VP_0-2_scaffold_141_5191495_68|SR-VP_0-2cm_Phage_38_25|SR-VP_0-2cm** | SR-VP_0-2_scaffold_141_5191495_68|SR-VP_0-2cm_Phage_38_25|SR-VP_0-2cm | 100.000 | 946 | 0 | 0 | 1 | 946 | 1 | 946 | 0.0 | 1932 |
| **SR-VP_0-2_scaffold_141_5191495_68|SR-VP_0-2cm_Phage_38_25|SR-VP_0-2cm** | P0_An_pond3_S8_coassembly_k141_1182798_2|P0_An_pond3_S8_coassembly_UNK|E_GD2017-1_P0_An_pond3_S8_Biohub_coassembly | 69.790 | 523 | 108 | 3 | 1 | 502 | 3 | 496 | 0.0 | 729 |
| **SR-VP_0-2_scaffold_141_5191495_68|SR-VP_0-2cm_Phage_38_25|SR-VP_0-2cm** | P0_An_pond3_S8_coassembly_k141_1182798_2|P0_An_pond3_S8_coassembly_UNK|E_GD2017-1_P0_An_pond3_S8_Biohub_coassembly | 66.667 | 255 | 85 | 0 | 675 | 929 | 485 | 739 | 1.72E-112 | 374 |
| **SR-VP_0-2_scaffold_141_5191495_68|SR-VP_0-2cm_Phage_38_25|SR-VP_0-2cm** | S15_GE15_scaffold_109408_1|E_GD2017-2_urea-2_S15_GE15_Biohub_170515_UNK|E_GD2017-2_urea-2_S15_GE15_Biohub_180515 | 69.790 | 523 | 108 | 3 | 1 | 502 | 3 | 496 | 0.0 | 728 |
| **SR-VP_0-2_scaffold_141_5191495_68|SR-VP_0-2cm_Phage_38_25|SR-VP_0-2cm** | S15_GE15_scaffold_109408_1|E_GD2017-2_urea-2_S15_GE15_Biohub_170515_UNK|E_GD2017-2_urea-2_S15_GE15_Biohub_180515 | 64.957 | 117 | 41 | 0 | 675 | 791 | 485 | 601 | 1.7E-42 | 177 |
| **SR-VP_0-2_scaffold_141_5191495_68|SR-VP_0-2cm_Phage_38_25|SR-VP_0-2cm** | S16_GE16_scaffold_592262_1|E_GD2017-2_urea-3_S16_GE16_Biohub_170515_UNK|E_GD2017-2_urea-3_S16_GE16_Biohub_180515 | 71.858 | 366 | 75 | 1 | 137 | 502 | 1 | 338 | 5.95E-177 | 535 |
| **SR-VP_0-2_scaffold_141_5191495_68|SR-VP_0-2cm_Phage_38_25|SR-VP_0-2cm** | S16_GE16_scaffold_592262_1|E_GD2017-2_urea-3_S16_GE16_Biohub_170515_UNK|E_GD2017-2_urea-3_S16_GE16_Biohub_180515 | 67.431 | 218 | 71 | 0 | 675 | 892 | 327 | 544 | 8.52E-96 | 323 |
| **SR-VP_0-2_scaffold_141_5191495_68|SR-VP_0-2cm_Phage_38_25|SR-VP_0-2cm** | P0_An_GD2017L_S7_coassembly_k141_704205_1|P0_An_GD2017L_S7_coassembly_UNK|E_P0_An_GD2017L_S7_coassembly | 69.184 | 331 | 74 | 1 | 172 | 502 | 1 | 303 | 1.56E-149 | 462 |
| **SR-VP_0-2_scaffold_141_5191495_68|SR-VP_0-2cm_Phage_38_25|SR-VP_0-2cm** | P0_An_GD2017L_S7_coassembly_k141_704205_1|P0_An_GD2017L_S7_coassembly_UNK|E_P0_An_GD2017L_S7_coassembly | 67.647 | 204 | 66 | 0 | 675 | 878 | 292 | 495 | 2.35E-89 | 305 |
| **SR-VP_0-2_scaffold_141_5191495_68|SR-VP_0-2cm_Phage_38_25|SR-VP_0-2cm** | ERMZT366_2_scaffold_2856_2|ERMZT366_2_UNK|ERMZT366_2 | 53.911 | 358 | 163 | 1 | 4 | 359 | 261 | 618 | 5.56E-119 | 387 |
| **SR-VP_0-2_scaffold_141_5191495_68|SR-VP_0-2cm_Phage_38_25|SR-VP_0-2cm** | BC_09192017_0_5m_scaffold_24519_2|BC_09192017_0_5m_UNK|BC_09192017_0_5m | 44.175 | 412 | 223 | 5 | 20 | 428 | 20 | 427 | 6.22E-107 | 348 |
| **SR-VP_0-2_scaffold_141_5191495_68|SR-VP_0-2cm_Phage_38_25|SR-VP_0-2cm** | BC_09192017_0_5m_scaffold_24526_2|BC_09192017_0_5m_UNK|BC_09192017_0_5m | 44.417 | 412 | 222 | 5 | 20 | 428 | 20 | 427 | 4.06E-106 | 347 |
| **SR-VP_0-2_scaffold_141_5191495_68|SR-VP_0-2cm_Phage_38_25|SR-VP_0-2cm** | ERMZT517_2_scaffold_27826_2|ERMZT517_2_UNK|ERMZT517_2 | 55.019 | 269 | 121 | 0 | 121 | 389 | 1 | 269 | 4.61E-86 | 294 |
| **SR-VP_0-2_scaffold_141_5191495_68|SR-VP_0-2cm_Phage_38_25|SR-VP_0-2cm** | FFC_04162018_0_1um_scaffold_562_1|FFC_04162018_0_1um_UNK|FFC_04162018_0_1um | 42.382 | 361 | 196 | 5 | 5 | 364 | 8 | 357 | 6.61E-86 | 290 |
| **SR-VP_0-2_scaffold_141_5191495_68|SR-VP_0-2cm_Phage_38_25|SR-VP_0-2cm** | RifSed_csp1_16ft_1_scaffold_50415_4|RifSed_csp1_16ft_1_UNK|RifSed_csp1_16ft_1 | 37.269 | 432 | 256 | 7 | 5 | 428 | 474 | 898 | 1.47E-82 | 297 |
| **SR-VP_0-2_scaffold_141_5191495_68|SR-VP_0-2cm_Phage_38_25|SR-VP_0-2cm** | RifSed_csp1_19ft_3_scaffold_29188_5|RifSed_csp1_19ft_3_Maxbin2_249|RifSed_csp1_19ft_3 | 37.269 | 432 | 256 | 7 | 5 | 428 | 495 | 919 | 2.43E-82 | 297 |
| **SR-VP_0-2_scaffold_141_5191495_68|SR-VP_0-2cm_Phage_38_25|SR-VP_0-2cm** | RifSed_csp1_19ft_1_scaffold_28051_2|RifSed_csp1_19ft_1_UNK|RifSed_csp1_19ft_1 | 37.269 | 432 | 256 | 7 | 5 | 428 | 495 | 919 | 2.43E-82 | 297 |
| **SR-VP_0-2_scaffold_141_5191495_68|SR-VP_0-2cm_Phage_38_25|SR-VP_0-2cm** | RifSed_csp1_16ft_4_scaffold_19582_5|RifSed_csp1_16ft_4_Maxbin2_189|RifSed_csp1_16ft_4 | 37.269 | 432 | 256 | 7 | 5 | 428 | 495 | 919 | 2.43E-82 | 297 |
| **SR-VP_0-2_scaffold_141_5191495_68|SR-VP_0-2cm_Phage_38_25|SR-VP_0-2cm** | RifSed_csp1_16ft_2_scaffold_10403_23|RifSed_csp1_16ft_2_UNK|RifSed_csp1_16ft_2 | 37.269 | 432 | 256 | 7 | 5 | 428 | 495 | 919 | 2.43E-82 | 297 |
| **SR-VP_0-2_scaffold_141_5191495_68|SR-VP_0-2cm_Phage_38_25|SR-VP_0-2cm** | RifSed_csp1_19ft_2_scaffold_3679_9|RifSed_csp1_19ft_2_UNK|RifSed_csp1_19ft_2 | 37.269 | 432 | 256 | 7 | 5 | 428 | 495 | 919 | 2.43E-82 | 297 |
| **SR-VP_0-2_scaffold_141_5191495_68|SR-VP_0-2cm_Phage_38_25|SR-VP_0-2cm** | rifcsp2_19ft_1_scaffold_24192_2|RifCSP19_1_full_UNK|RIFCSP19_1_FULL | 37.269 | 432 | 256 | 7 | 5 | 428 | 495 | 919 | 2.43E-82 | 297 |
| **SR-VP_0-2_scaffold_141_5191495_68|SR-VP_0-2cm_Phage_38_25|SR-VP_0-2cm** | rifcsplowo2_12_scaffold_2007_23|RifCSPlowO2_12_FULL_UNK|RIFCSPLOWO2_12_FULL | 37.269 | 432 | 256 | 7 | 5 | 428 | 495 | 919 | 2.43E-82 | 297 |
| **SR-VP_0-2_scaffold_141_5191495_68|SR-VP_0-2cm_Phage_38_25|SR-VP_0-2cm** | rifcsphigho2_12_scaffold_1835_33|RifCSPhighO2_12_full_UNK|RIFCSPHIGHO2_12_FULL | 37.269 | 432 | 256 | 7 | 5 | 428 | 495 | 919 | 2.43E-82 | 297 |
| **SR-VP_0-2_scaffold_141_5191495_68|SR-VP_0-2cm_Phage_38_25|SR-VP_0-2cm** | Rifle_16ft_4_minimus_14884_9|16ft_4_MIN_UNK|16FT_4_MIN | 37.269 | 432 | 256 | 7 | 5 | 428 | 495 | 919 | 2.43E-82 | 297 |
| **SR-VP_0-2_scaffold_141_5191495_68|SR-VP_0-2cm_Phage_38_25|SR-VP_0-2cm** | 19ft_2_nophage_noknown_scaffold_2724_23|19ft_2_UNK|19FT_2_THINNED | 37.269 | 432 | 256 | 7 | 5 | 428 | 495 | 919 | 2.43E-82 | 297 |
| **SR-VP_0-2_scaffold_141_5191495_68|SR-VP_0-2cm_Phage_38_25|SR-VP_0-2cm** | 16ft_4_scaffold_18225_9|16ft_4_UNK|16ft_4 | 37.269 | 432 | 256 | 7 | 5 | 428 | 495 | 919 | 2.43E-82 | 297 |
| **SR-VP_0-2_scaffold_141_5191495_68|SR-VP_0-2cm_Phage_38_25|SR-VP_0-2cm** | gwc1_scaffold_679_2|GWC1 | 37.269 | 432 | 256 | 7 | 5 | 428 | 495 | 919 | 2.43E-82 | 297 |
| **SR-VP_0-2_scaffold_141_5191495_68|SR-VP_0-2cm_Phage_38_25|SR-VP_0-2cm** | GWB1_scaffold_872_11|GWB1 | 37.269 | 432 | 256 | 7 | 5 | 428 | 495 | 919 | 2.43E-82 | 297 |
| **SR-VP_0-2_scaffold_141_5191495_68|SR-VP_0-2cm_Phage_38_25|SR-VP_0-2cm** | gwa1_scaffold_544_2|GWA1 | 37.269 | 432 | 256 | 7 | 5 | 428 | 495 | 919 | 2.43E-82 | 297 |
| **SR-VP_0-2_scaffold_141_5191495_68|SR-VP_0-2cm_Phage_38_25|SR-VP_0-2cm** | SP6_scaffold_43725_1|sea_ice_sp6_UNK|sea_ice_sp6 | 36.967 | 422 | 254 | 5 | 12 | 426 | 162 | 578 | 2.72E-82 | 288 |
| **SR-VP_0-2_scaffold_141_5191495_68|SR-VP_0-2cm_Phage_38_25|SR-VP_0-2cm** | P0_An_GD2017L_S7_coassembly_k141_2601098_2|P0_An_GD2017L_S7_coassembly_UNK|E_P0_An_GD2017L_S7_coassembly | 69.948 | 193 | 36 | 2 | 1 | 172 | 3 | 194 | 1.07E-79 | 268 |
| **SR-VP_0-2_scaffold_141_5191495_68|SR-VP_0-2cm_Phage_38_25|SR-VP_0-2cm** | RifSed_csp1_19ft_4_scaffold_6883_2|RifSed_csp1_19ft_4_UNK|RifSed_csp1_19ft_4 | 37.406 | 401 | 238 | 6 | 36 | 428 | 1 | 396 | 1.17E-79 | 275 |
| **SR-VP_0-2_scaffold_141_5191495_68|SR-VP_0-2cm_Phage_38_25|SR-VP_0-2cm** | anamox4_scaffold_5988_1|anamox4_UNK|anamox4 | 37.288 | 413 | 244 | 6 | 20 | 426 | 271 | 674 | 3.09E-79 | 283 |
| **SR-VP_0-2_scaffold_141_5191495_68|SR-VP_0-2cm_Phage_38_25|SR-VP_0-2cm** | LAC_NA08_scaffold_2624_6|LAC_NA08_UNK|LAC_NA08 | 37.288 | 413 | 244 | 6 | 20 | 426 | 515 | 918 | 2.8E-77 | 283 |
| **SR-VP_0-2_scaffold_141_5191495_68|SR-VP_0-2cm_Phage_38_25|SR-VP_0-2cm** | LacPavin_0818_WC45_scaffold_260245_1|LacPavin_0818_WC45_UNK|LacPavin_0818_WC45 | 46.758 | 293 | 141 | 4 | 658 | 935 | 164 | 456 | 5.5E-77 | 270 |
| **SR-VP_0-2_scaffold_141_5191495_68|SR-VP_0-2cm_Phage_38_25|SR-VP_0-2cm** | LAC_NA04_scaffold_2971_6|NA04_UNK|NA04 | 37.288 | 413 | 244 | 6 | 20 | 426 | 563 | 966 | 1.32E-76 | 281 |
| **SR-VP_0-2_scaffold_141_5191495_68|SR-VP_0-2cm_Phage_38_25|SR-VP_0-2cm** | SRVP18_trench_3_45cm_scaffold_37267_2|SRVP18_trench_3_45cm_UNK|SRVP18_trench_3_45cm | 37.831 | 415 | 243 | 7 | 20 | 428 | 13 | 418 | 2.28E-76 | 267 |
| **SR-VP_0-2_scaffold_141_5191495_68|SR-VP_0-2cm_Phage_38_25|SR-VP_0-2cm** | FFC_04162018_0_1um_scaffold_776_1|FFC_04162018_0_1um_UNK|FFC_04162018_0_1um | 48.123 | 293 | 137 | 4 | 658 | 935 | 415 | 707 | 3.21E-76 | 275 |
| **SR-VP_0-2_scaffold_141_5191495_68|SR-VP_0-2cm_Phage_38_25|SR-VP_0-2cm** | LAC_NA05_scaffold_109_82|NA05_UNK|NA05 | 37.288 | 413 | 244 | 6 | 20 | 426 | 735 | 1138 | 4.98E-76 | 282 |
| **SR-VP_0-2_scaffold_141_5191495_68|SR-VP_0-2cm_Phage_38_25|SR-VP_0-2cm** | LAC_NA03_scaffold_201_12|NA03_UNK|NA03 | 37.288 | 413 | 244 | 6 | 20 | 426 | 735 | 1138 | 4.98E-76 | 282 |
| **SR-VP_0-2_scaffold_141_5191495_68|SR-VP_0-2cm_Phage_38_25|SR-VP_0-2cm** | LAC_NA01_scaffold_417_69|NA01_UNK|NA01 | 37.288 | 413 | 244 | 6 | 20 | 426 | 735 | 1138 | 4.98E-76 | 282 |
| **SR-VP_0-2_scaffold_141_5191495_68|SR-VP_0-2cm_Phage_38_25|SR-VP_0-2cm** | LAC_NA06_scaffold_75_291|LAC_NA06_UNK|LAC_NA06 | 37.288 | 413 | 244 | 6 | 20 | 426 | 735 | 1138 | 4.98E-76 | 282 |
| **SR-VP_0-2_scaffold_141_5191495_68|SR-VP_0-2cm_Phage_38_25|SR-VP_0-2cm** | LAC_NA10_scaffold_1061_13|LAC_NA10_UNK|LAC_NA10 | 37.288 | 413 | 244 | 6 | 20 | 426 | 735 | 1138 | 4.98E-76 | 282 |
| **SR-VP_0-2_scaffold_141_5191495_68|SR-VP_0-2cm_Phage_38_25|SR-VP_0-2cm** | LAC_NA09_scaffold_171_12|LAC_NA09_UNK|LAC_NA09 | 37.288 | 413 | 244 | 6 | 20 | 426 | 735 | 1138 | 4.98E-76 | 282 |
| **SR-VP_0-2_scaffold_141_5191495_68|SR-VP_0-2cm_Phage_38_25|SR-VP_0-2cm** | Salt_Pond_R2_restored_H2O_MG_scaffold_682_5|JGI_Salt_Pond_R2_restored_H2O_MG_UNK|JGI_Salt_Pond_R2_restored_H2O_MG | 36.028 | 433 | 262 | 7 | 4 | 426 | 754 | 1181 | 9.64E-75 | 279 |
| **SR-VP_0-2_scaffold_141_5191495_68|SR-VP_0-2cm_Phage_38_25|SR-VP_0-2cm** | BC_09192017_0_5m_scaffold_1685_2|BC_09192017_0_5m_UNK|BC_09192017_0_5m | 48.630 | 292 | 135 | 4 | 658 | 934 | 692 | 983 | 1.03E-74 | 276 |
| **SR-VP_0-2_scaffold_141_5191495_68|SR-VP_0-2cm_Phage_38_25|SR-VP_0-2cm** | SP8F_scaffold_41569_1|sea_ice_sp8_UNK|sea_ice_sp8 | 35.663 | 415 | 254 | 6 | 20 | 426 | 49 | 458 | 1.2E-74 | 264 |
| **SR-VP_0-2_scaffold_141_5191495_68|SR-VP_0-2cm_Phage_38_25|SR-VP_0-2cm** | P0_An_pond3_S8_coassembly_k141_1893190_4|P0_An_pond3_S8_coassembly_UNK|E_GD2017-1_P0_An_pond3_S8_Biohub_coassembly | 35.142 | 424 | 258 | 8 | 13 | 428 | 54 | 468 | 3.2E-74 | 263 |
| **SR-VP_0-2_scaffold_141_5191495_68|SR-VP_0-2cm_Phage_38_25|SR-VP_0-2cm** | AB_092018_0_1um_scaffold_3731_5|AB_092018_0_1um_UNK|AB_092018_0_1um | 48.123 | 293 | 137 | 4 | 658 | 935 | 661 | 953 | 4.98E-74 | 274 |
| **SR-VP_0-2_scaffold_141_5191495_68|SR-VP_0-2cm_Phage_38_25|SR-VP_0-2cm** | BC_09192017_0_5m_scaffold_48618_1|BC_09192017_0_5m_UNK|BC_09192017_0_5m | 36.957 | 414 | 249 | 6 | 20 | 426 | 143 | 551 | 5.77E-74 | 265 |
| **SR-VP_0-2_scaffold_141_5191495_68|SR-VP_0-2cm_Phage_38_25|SR-VP_0-2cm** | AB_072018_0_1um_scaffold_711_8|AB_072018_0_1um_UNK|AB_072018_0_1um | 48.123 | 293 | 137 | 4 | 658 | 935 | 692 | 984 | 8.92E-74 | 274 |
| **SR-VP_0-2_scaffold_141_5191495_68|SR-VP_0-2cm_Phage_38_25|SR-VP_0-2cm** | Salt_Pond_SF2_A_H2O_MG_scaffold_593_1|JGI_Salt_Pond_SF2_A_H2O_MG_UNK|JGI_Salt_Pond_SF2_A_H2O_MG | 34.699 | 415 | 258 | 6 | 20 | 426 | 206 | 615 | 3.9E-73 | 264 |
| **SR-VP_0-2_scaffold_141_5191495_68|SR-VP_0-2cm_Phage_38_25|SR-VP_0-2cm** | GS605_0p1_scaffold_738_27|lsdeep_GS605_0p1_UNK|lsdeep_GS605_0p1 | 36.256 | 422 | 257 | 5 | 12 | 426 | 1028 | 1444 | 4.93E-73 | 276 |
| **SR-VP_0-2_scaffold_141_5191495_68|SR-VP_0-2cm_Phage_38_25|SR-VP_0-2cm** | Salt_Pond_R2A_C_H2O_MG_scaffold_36848_1|JGI_Salt_Pond_R2A_C_H2O_MG_UNK|JGI_Salt_Pond_R2A_C_H2O_MG | 33.645 | 428 | 270 | 5 | 9 | 426 | 87 | 510 | 5.26E-73 | 261 |
| **SR-VP_0-2_scaffold_141_5191495_68|SR-VP_0-2cm_Phage_38_25|SR-VP_0-2cm** | RHP_09252018_0_1um_scaffold_618_37|RHP_09252018_0_1um_UNK|RHP_09252018_0_1um | 37.560 | 418 | 245 | 6 | 20 | 426 | 230 | 642 | 5.97E-73 | 265 |
| **SR-VP_0-2_scaffold_141_5191495_68|SR-VP_0-2cm_Phage_38_25|SR-VP_0-2cm** | GS843_0p1_scaffold_644_32|lsdeep_GS843_0p1_UNK|lsdeep_GS843_0p1 | 35.181 | 415 | 256 | 6 | 20 | 426 | 638 | 1047 | 1.93E-72 | 271 |
| **SR-VP_0-2_scaffold_141_5191495_68|SR-VP_0-2cm_Phage_38_25|SR-VP_0-2cm** | Salt_Pond_SF2_C_H2O_MG_scaffold_20010_4|JGI_Salt_Pond_SF2_C_H2O_MG_UNK|JGI_Salt_Pond_SF2_C_H2O_MG | 35.904 | 415 | 253 | 6 | 20 | 426 | 413 | 822 | 3.4E-72 | 266 |
| **SR-VP_0-2_scaffold_141_5191495_68|SR-VP_0-2cm_Phage_38_25|SR-VP_0-2cm** | LacPavin_0818_WC40_scaffold_532593_4|LacPavin_0818_WC40_UNK|LacPavin_0818_WC40 | 46.758 | 293 | 141 | 4 | 658 | 935 | 692 | 984 | 4.45E-72 | 269 |
| **SR-VP_0-2_scaffold_141_5191495_68|SR-VP_0-2cm_Phage_38_25|SR-VP_0-2cm** | LacPavin_0419_WC53_scaffold_76481_2|LacPavin_0419_WC53_UNK|LacPavin_0419_WC53 | 49.813 | 267 | 120 | 3 | 683 | 935 | 718 | 984 | 6.13E-72 | 269 |
| **SR-VP_0-2_scaffold_141_5191495_68|SR-VP_0-2cm_Phage_38_25|SR-VP_0-2cm** | Salt_Pond_SF2_B_H2O_MG_scaffold_1443_17|JGI_Salt_Pond_SF2_B_H2O_MG_UNK|JGI_Salt_Pond_SF2_B_H2O_MG | 34.515 | 423 | 264 | 6 | 12 | 426 | 393 | 810 | 1.48E-71 | 265 |
| **SR-VP_0-2_scaffold_141_5191495_68|SR-VP_0-2cm_Phage_38_25|SR-VP_0-2cm** | GS605_0p1_scaffold_1309_5|lsdeep_GS605_0p1_UNK|lsdeep_GS605_0p1 | 36.627 | 415 | 250 | 6 | 20 | 426 | 687 | 1096 | 2.22E-71 | 268 |
| **SR-VP_0-2_scaffold_141_5191495_68|SR-VP_0-2cm_Phage_38_25|SR-VP_0-2cm** | AB_082018_0_1um_scaffold_15827_3|AB_082018_0_1um_UNK|AB_082018_0_1um | 34.346 | 428 | 258 | 9 | 7 | 424 | 3 | 417 | 4.83E-71 | 253 |
| **SR-VP_0-2_scaffold_141_5191495_68|SR-VP_0-2cm_Phage_38_25|SR-VP_0-2cm** | ALT_082018_0_1um_scaffold_8173_1|ALT_082018_0_1um_UNK|ALT_082018_0_1um | 35.535 | 439 | 258 | 8 | 11 | 428 | 171 | 605 | 1.76E-70 | 257 |
| **SR-VP_0-2_scaffold_141_5191495_68|SR-VP_0-2cm_Phage_38_25|SR-VP_0-2cm** | LacPavin_0419_WC70S_scaffold_883483_2|LacPavin_0419_WC70S_UNK|LacPavin_0419_WC70S | 33.816 | 414 | 258 | 6 | 22 | 428 | 8 | 412 | 1.81E-70 | 251 |
| **SR-VP_0-2_scaffold_141_5191495_68|SR-VP_0-2cm_Phage_38_25|SR-VP_0-2cm** | RHP_09252018_0_1um_scaffold_2419_25|RHP_09252018_0_1um_UNK|RHP_09252018_0_1um | 34.644 | 407 | 250 | 6 | 29 | 428 | 1 | 398 | 4.59E-70 | 249 |
| **SR-VP_0-2_scaffold_141_5191495_68|SR-VP_0-2cm_Phage_38_25|SR-VP_0-2cm** | Salt_Pond_R2A_B_H2O_MG_scaffold_9641_4|JGI_Salt_Pond_R2A_B_H2O_MG_UNK|JGI_Salt_Pond_R2A_B_H2O_MG | 34.670 | 424 | 254 | 7 | 17 | 426 | 205 | 619 | 4.72E-70 | 256 |
| **SR-VP_0-2_scaffold_141_5191495_68|SR-VP_0-2cm_Phage_38_25|SR-VP_0-2cm** | LacPavin_0818_WC45_scaffold_246313_1|LacPavin_0818_WC45_UNK|LacPavin_0818_WC45 | 34.615 | 416 | 252 | 8 | 16 | 424 | 95 | 497 | 6.37E-70 | 252 |
| **SR-VP_0-2_scaffold_141_5191495_68|SR-VP_0-2cm_Phage_38_25|SR-VP_0-2cm** | GS605_0p1_scaffold_1118_1|lsdeep_GS605_0p1_UNK|lsdeep_GS605_0p1 | 34.058 | 414 | 257 | 6 | 20 | 426 | 1057 | 1461 | 1.92E-69 | 265 |
| **SR-VP_0-2_scaffold_141_5191495_68|SR-VP_0-2cm_Phage_38_25|SR-VP_0-2cm** | Salt_Pond_SF2_C_H2O_MG_scaffold_1705_4|JGI_Salt_Pond_SF2_C_H2O_MG_UNK|JGI_Salt_Pond_SF2_C_H2O_MG | 34.699 | 415 | 258 | 6 | 20 | 426 | 914 | 1323 | 3.47E-69 | 264 |
| **SR-VP_0-2_scaffold_141_5191495_68|SR-VP_0-2cm_Phage_38_25|SR-VP_0-2cm** | qh_9_scaffold_7944_5|QH_9_UNK|QH_9 | 34.286 | 420 | 259 | 7 | 18 | 426 | 40 | 453 | 3.82E-69 | 248 |
| **SR-VP_0-2_scaffold_141_5191495_68|SR-VP_0-2cm_Phage_38_25|SR-VP_0-2cm** | sw_7_scaffold_1_prodigal-single_98|SW-7_PHAGE_46_16|SW_7 | 34.862 | 436 | 267 | 7 | 4 | 428 | 530 | 959 | 4.91E-69 | 260 |
| **SR-VP_0-2_scaffold_141_5191495_68|SR-VP_0-2cm_Phage_38_25|SR-VP_0-2cm** | sw_7_scaffold_1_98|SW_7_Phage_46_16|SW_7 | 34.862 | 436 | 267 | 7 | 4 | 428 | 530 | 959 | 4.91E-69 | 260 |
| **SR-VP_0-2_scaffold_141_5191495_68|SR-VP_0-2cm_Phage_38_25|SR-VP_0-2cm** | Salt_Pond_SF2_B_H2O_MG_scaffold_11776_5|JGI_Salt_Pond_SF2_B_H2O_MG_UNK|JGI_Salt_Pond_SF2_B_H2O_MG | 34.524 | 420 | 260 | 7 | 17 | 426 | 147 | 561 | 1.02E-68 | 251 |
| **SR-VP_0-2_scaffold_141_5191495_68|SR-VP_0-2cm_Phage_38_25|SR-VP_0-2cm** | Salt_Pond_SF2_B_H2O_MG_scaffold_67_3|JGI_Salt_Pond_SF2_B_H2O_MG_UNK|JGI_Salt_Pond_SF2_B_H2O_MG | 33.645 | 428 | 270 | 5 | 9 | 426 | 859 | 1282 | 1.14E-68 | 262 |
| **SR-VP_0-2_scaffold_141_5191495_68|SR-VP_0-2cm_Phage_38_25|SR-VP_0-2cm** | Salt_Pond_SF2_A_H2O_MG_scaffold_295_3|JGI_Salt_Pond_SF2_A_H2O_MG_UNK|JGI_Salt_Pond_SF2_A_H2O_MG | 33.645 | 428 | 270 | 5 | 9 | 426 | 859 | 1282 | 1.14E-68 | 262 |
| **SR-VP_0-2_scaffold_141_5191495_68|SR-VP_0-2cm_Phage_38_25|SR-VP_0-2cm** | Salt_Pond_SF2_C_H2O_MG_scaffold_12_47|JGI_Salt_Pond_SF2_C_H2O_MG_UNK|JGI_Salt_Pond_SF2_C_H2O_MG | 33.645 | 428 | 270 | 5 | 9 | 426 | 859 | 1282 | 1.14E-68 | 262 |
| **SR-VP_0-2_scaffold_141_5191495_68|SR-VP_0-2cm_Phage_38_25|SR-VP_0-2cm** | RHP_09252018_0_1um_scaffold_851_7|RHP_09252018_0_1um_UNK|RHP_09252018_0_1um | 34.895 | 427 | 255 | 9 | 8 | 424 | 386 | 799 | 1.83E-68 | 256 |
| **SR-VP_0-2_scaffold_141_5191495_68|SR-VP_0-2cm_Phage_38_25|SR-VP_0-2cm** | GS605_0p1_scaffold_98_20|lsdeep_GS605_0p1_UNK|lsdeep_GS605_0p1 | 35.238 | 420 | 249 | 7 | 20 | 426 | 782 | 1191 | 5.44E-68 | 259 |
| **SR-VP_0-2_scaffold_141_5191495_68|SR-VP_0-2cm_Phage_38_25|SR-VP_0-2cm** | qh_8_scaffold_10_68|QH_8_UNK|QH_8 | 35.000 | 420 | 256 | 7 | 20 | 428 | 540 | 953 | 7.24E-68 | 256 |
| **SR-VP_0-2_scaffold_141_5191495_68|SR-VP_0-2cm_Phage_38_25|SR-VP_0-2cm** | LacPavin_0818_WC40_scaffold_81873_14|LacPavin_0818_WC40_UNK|LacPavin_0818_WC40 | 33.575 | 414 | 259 | 6 | 22 | 428 | 270 | 674 | 8.43E-68 | 251 |
| **SR-VP_0-2_scaffold_141_5191495_68|SR-VP_0-2cm_Phage_38_25|SR-VP_0-2cm** | qh_6_scaffold_504_4|QH_6_UNK|QH_6 | 35.000 | 420 | 256 | 7 | 20 | 428 | 540 | 953 | 9.3E-68 | 256 |
| **SR-VP_0-2_scaffold_141_5191495_68|SR-VP_0-2cm_Phage_38_25|SR-VP_0-2cm** | SW_scaffold_197533_1|LAC_SW_UNK|lac_sw | 34.466 | 412 | 247 | 8 | 22 | 424 | 110 | 507 | 1.37E-67 | 246 |
| **SR-VP_0-2_scaffold_141_5191495_68|SR-VP_0-2cm_Phage_38_25|SR-VP_0-2cm** | PH2017_35_SFW_U_B_scaffold_4543_2|PH2017_35_SFW_U_B_full_UNK|PH2017_35_SFW_U_B_full | 35.572 | 402 | 238 | 8 | 29 | 424 | 1 | 387 | 1.39E-67 | 242 |
| **SR-VP_0-2_scaffold_141_5191495_68|SR-VP_0-2cm_Phage_38_25|SR-VP_0-2cm** | PH2017_35_SFW_U_B_scaffold_3944_2|PH2017_35_SFW_U_B_filtered_BT2def_UNK|PH2017_35_SFW_U_B_filtered_BT2def | 35.572 | 402 | 238 | 8 | 29 | 424 | 1 | 387 | 1.39E-67 | 242 |
| **SR-VP_0-2_scaffold_141_5191495_68|SR-VP_0-2cm_Phage_38_25|SR-VP_0-2cm** | PH2017_35_SFW_U_B_scaffold_4563_2|PH2017_35_SFW_U_B_filtered_UNK|PH2017_35_SFW_U_B_filtered | 35.572 | 402 | 238 | 8 | 29 | 424 | 1 | 387 | 1.39E-67 | 242 |
| **SR-VP_0-2_scaffold_141_5191495_68|SR-VP_0-2cm_Phage_38_25|SR-VP_0-2cm** | qh_11_scaffold_73_17|QH_11_UNK|QH_11 | 35.071 | 422 | 257 | 7 | 18 | 428 | 546 | 961 | 1.59E-67 | 256 |
| **SR-VP_0-2_scaffold_141_5191495_68|SR-VP_0-2cm_Phage_38_25|SR-VP_0-2cm** | qh_10_scaffold_74_17|QH_10_UNK|QH_10 | 35.071 | 422 | 257 | 7 | 18 | 428 | 546 | 961 | 1.59E-67 | 256 |
| **SR-VP_0-2_scaffold_141_5191495_68|SR-VP_0-2cm_Phage_38_25|SR-VP_0-2cm** | LacPavin_0818_WC40_scaffold_1310565_2|LacPavin_0818_WC40_UNK|LacPavin_0818_WC40 | 34.634 | 410 | 250 | 7 | 20 | 424 | 401 | 797 | 1.68E-67 | 253 |
| **SR-VP_0-2_scaffold_141_5191495_68|SR-VP_0-2cm_Phage_38_25|SR-VP_0-2cm** | AB_092018_0_1um_scaffold_25296_1|AB_092018_0_1um_UNK|AB_092018_0_1um | 34.346 | 428 | 262 | 7 | 11 | 428 | 274 | 692 | 1.93E-67 | 250 |
| **SR-VP_0-2_scaffold_141_5191495_68|SR-VP_0-2cm_Phage_38_25|SR-VP_0-2cm** | Salt_Pond_SF2_A_H2O_MG_scaffold_3756_1|JGI_Salt_Pond_SF2_A_H2O_MG_UNK|JGI_Salt_Pond_SF2_A_H2O_MG | 34.906 | 424 | 253 | 7 | 17 | 426 | 707 | 1121 | 3.37E-67 | 256 |
| **SR-VP_0-2_scaffold_141_5191495_68|SR-VP_0-2cm_Phage_38_25|SR-VP_0-2cm** | SP8F_scaffold_1167_25|sea_ice_sp8_UNK|sea_ice_sp8 | 35.629 | 421 | 252 | 7 | 18 | 428 | 307 | 718 | 3.76E-67 | 250 |
| **SR-VP_0-2_scaffold_141_5191495_68|SR-VP_0-2cm_Phage_38_25|SR-VP_0-2cm** | FFC_082018_0_1um_scaffold_412_7|FFC_082018_0_1um_UNK|FFC_082018_0_1um | 34.192 | 427 | 258 | 9 | 8 | 424 | 469 | 882 | 5.44E-67 | 253 |
| **SR-VP_0-2_scaffold_141_5191495_68|SR-VP_0-2cm_Phage_38_25|SR-VP_0-2cm** | sw_4_scaffold_1_47|SW_4_UNK|SW_4 | 35.084 | 419 | 256 | 6 | 18 | 426 | 781 | 1193 | 1.29E-66 | 255 |
| **SR-VP_0-2_scaffold_141_5191495_68|SR-VP_0-2cm_Phage_38_25|SR-VP_0-2cm** | BML_coassembly_scaffold_87733_4|BML_coassembly_UNK|BML_coassembly | 36.180 | 445 | 254 | 13 | 3 | 426 | 72 | 507 | 1.32E-66 | 243 |
| **SR-VP_0-2_scaffold_141_5191495_68|SR-VP_0-2cm_Phage_38_25|SR-VP_0-2cm** | k87_7858187_2|zodeltone_water_may_2017_UNK|zodeltone_water_may_2017 | 33.898 | 413 | 261 | 6 | 20 | 428 | 19 | 423 | 1.73E-66 | 240 |
| **SR-VP_0-2_scaffold_141_5191495_68|SR-VP_0-2cm_Phage_38_25|SR-VP_0-2cm** | sw_12_scaffold_5_48|SW_12_Viruses_57_8|SW_12 | 35.154 | 421 | 255 | 8 | 18 | 426 | 783 | 1197 | 1.91E-66 | 255 |
| **SR-VP_0-2_scaffold_141_5191495_68|SR-VP_0-2cm_Phage_38_25|SR-VP_0-2cm** | SW_scaffold_180664_1|LAC_SW_UNK|lac_sw | 33.894 | 416 | 260 | 6 | 20 | 426 | 123 | 532 | 6.27E-66 | 242 |
| **SR-VP_0-2_scaffold_141_5191495_68|SR-VP_0-2cm_Phage_38_25|SR-VP_0-2cm** | GWB1_scaffold_5870_7|GWB1 | 37.666 | 377 | 220 | 6 | 62 | 428 | 37 | 408 | 8.41E-66 | 238 |
| **SR-VP_0-2_scaffold_141_5191495_68|SR-VP_0-2cm_Phage_38_25|SR-VP_0-2cm** | LacPavin_0419_WC53_scaffold_165427_31|LacPavin_0419_WC53_UNK|LacPavin_0419_WC53 | 32.184 | 435 | 277 | 6 | 3 | 428 | 4 | 429 | 1.38E-65 | 238 |
| **SR-VP_0-2_scaffold_141_5191495_68|SR-VP_0-2cm_Phage_38_25|SR-VP_0-2cm** | LacPavin_0818_WC40_scaffold_288012_36|LacPavin_0818_WC40_UNK|LacPavin_0818_WC40 | 32.184 | 435 | 277 | 6 | 3 | 428 | 4 | 429 | 1.38E-65 | 238 |
| **SR-VP_0-2_scaffold_141_5191495_68|SR-VP_0-2cm_Phage_38_25|SR-VP_0-2cm** | gwc1_scaffold_10315_4|GWC1 | 37.401 | 377 | 221 | 6 | 62 | 428 | 37 | 408 | 1.98E-65 | 237 |
| **SR-VP_0-2_scaffold_141_5191495_68|SR-VP_0-2cm_Phage_38_25|SR-VP_0-2cm** | LacPavin_0818_WC45_scaffold_975382_2|LacPavin_0818_WC45_UNK|LacPavin_0818_WC45 | 33.915 | 401 | 247 | 7 | 29 | 424 | 1 | 388 | 2.15E-65 | 236 |
| **SR-VP_0-2_scaffold_141_5191495_69|SR-VP_0-2cm_Phage_38_25|SR-VP_0-2cm** | SR-VP_0-2_scaffold_141_5191495_prodigal-single_76|SR-VP_PHAGE_38_25|SR-VP_0-2cm | 100.000 | 204 | 0 | 0 | 1 | 204 | 1 | 204 | 8.89E-148 | 419 |
| **SR-VP_0-2_scaffold_141_5191495_69|SR-VP_0-2cm_Phage_38_25|SR-VP_0-2cm** | SR-VP_0-2_scaffold_141_5191495_69|SR-VP_0-2cm_Phage_38_25|SR-VP_0-2cm | 100.000 | 204 | 0 | 0 | 1 | 204 | 1 | 204 | 8.89E-148 | 419 |
| **SR-VP_0-2_scaffold_141_5191495_69|SR-VP_0-2cm_Phage_38_25|SR-VP_0-2cm** | SR-VP_0-2_scaffold_141_5755040_2|SR-VP_0-2cm_UNK|SR-VP_0-2cm | 74.874 | 199 | 50 | 0 | 1 | 199 | 1 | 199 | 2.42E-110 | 324 |
| **SR-VP_0-2_scaffold_141_5191495_69|SR-VP_0-2cm_Phage_38_25|SR-VP_0-2cm** | S15_GE15_scaffold_572745_2|E_GD2017-2_urea-2_S15_GE15_Biohub_170515_UNK|E_GD2017-2_urea-2_S15_GE15_Biohub_180515 | 70.149 | 201 | 60 | 0 | 1 | 201 | 1 | 201 | 6.54E-106 | 313 |
| **SR-VP_0-2_scaffold_141_5191495_69|SR-VP_0-2cm_Phage_38_25|SR-VP_0-2cm** | SR-VP_4-6_scaffold_141_3583822_3|SR-VP_4-6cm_Biohub_180515_UNK|SR-VP_4-6cm_Biohub_180515 | 100.000 | 120 | 0 | 0 | 1 | 120 | 1 | 120 | 1.36E-83 | 253 |
| **SR-VP_0-2_scaffold_141_5191495_69|SR-VP_0-2cm_Phage_38_25|SR-VP_0-2cm** | P0_An_pond3_S8_coassembly_k141_1308441_2|P0_An_pond3_S8_coassembly_UNK|E_GD2017-1_P0_An_pond3_S8_Biohub_coassembly | 74.675 | 154 | 39 | 0 | 48 | 201 | 1 | 154 | 4.87E-82 | 251 |
| **SR-VP_0-2_scaffold_141_5191495_69|SR-VP_0-2cm_Phage_38_25|SR-VP_0-2cm** | SR-VP_0-2_scaffold_141_6448357_7|SR-VP_0-2cm_UNK|SR-VP_0-2cm | 55.556 | 198 | 80 | 2 | 1 | 191 | 1 | 197 | 3.14E-77 | 240 |
| **SR-VP_0-2_scaffold_141_5191495_69|SR-VP_0-2cm_Phage_38_25|SR-VP_0-2cm** | PAFVLPS_2018_scaffold_536846_2|viral-cat_UNK|viral-cat | 57.368 | 190 | 81 | 0 | 1 | 190 | 1 | 190 | 6.4E-77 | 239 |
| **SR-VP_0-2_scaffold_141_5191495_69|SR-VP_0-2cm_Phage_38_25|SR-VP_0-2cm** | PAFVLPS_2018_scaffold_1087129_2|viral-cat_UNK|viral-cat | 57.653 | 196 | 82 | 1 | 1 | 196 | 1 | 195 | 6.53E-77 | 239 |
| **SR-VP_0-2_scaffold_141_5191495_69|SR-VP_0-2cm_Phage_38_25|SR-VP_0-2cm** | ERMZT133_2_scaffold_7473_1|ERMZT133_2_UNK|ERMZT133_2 | 58.947 | 190 | 78 | 0 | 1 | 190 | 1 | 190 | 1.97E-76 | 238 |
| **SR-VP_0-2_scaffold_141_5191495_69|SR-VP_0-2cm_Phage_38_25|SR-VP_0-2cm** | SR-VP_4-6_scaffold_141_7120738_2|SR-VP_4-6cm_Biohub_180515_UNK|SR-VP_4-6cm_Biohub_180515 | 54.545 | 198 | 82 | 2 | 1 | 191 | 11 | 207 | 1.33E-74 | 234 |
| **SR-VP_0-2_scaffold_141_5191495_69|SR-VP_0-2cm_Phage_38_25|SR-VP_0-2cm** | SR-VP_4-6_scaffold_141_6470619_2|SR-VP_4-6cm_Biohub_180515_UNK|SR-VP_4-6cm_Biohub_180515 | 55.959 | 193 | 85 | 0 | 1 | 193 | 1 | 193 | 4.01E-74 | 232 |
| **SR-VP_0-2_scaffold_141_5191495_69|SR-VP_0-2cm_Phage_38_25|SR-VP_0-2cm** | S16_GE16_scaffold_164849_2|E_GD2017-2_urea-3_S16_GE16_Biohub_170515_UNK|E_GD2017-2_urea-3_S16_GE16_Biohub_180515 | 74.074 | 135 | 35 | 0 | 67 | 201 | 1 | 135 | 1.48E-69 | 218 |
| **SR-VP_0-2_scaffold_141_5191495_69|SR-VP_0-2cm_Phage_38_25|SR-VP_0-2cm** | ERMLT800_2_scaffold_19362_2|ERMLT800_2_UNK|ERMLT800_2 | 51.832 | 191 | 92 | 0 | 1 | 191 | 1 | 191 | 4.45E-69 | 219 |
| **SR-VP_0-2_scaffold_141_5191495_69|SR-VP_0-2cm_Phage_38_25|SR-VP_0-2cm** | SRVP18_trench_1_20cm_scaffold_10130_3|SRVP18_trench_1_20cm_UNK|SRVP18_trench_1_20cm | 50.526 | 190 | 93 | 1 | 1 | 189 | 1 | 190 | 7.7E-68 | 216 |
| **SR-VP_0-2_scaffold_141_5191495_69|SR-VP_0-2cm_Phage_38_25|SR-VP_0-2cm** | LacPavin_0419_WC70S_scaffold_1090708_2|LacPavin_0419_WC70S_UNK|LacPavin_0419_WC70S | 50.251 | 199 | 98 | 1 | 1 | 199 | 1 | 198 | 1.79E-67 | 216 |
| **SR-VP_0-2_scaffold_141_5191495_69|SR-VP_0-2cm_Phage_38_25|SR-VP_0-2cm** | LacPavin_0419_WC70S_scaffold_436490_12|LacPavin_0419_WC70S_UNK|LacPavin_0419_WC70S | 50.000 | 196 | 98 | 0 | 1 | 196 | 1 | 196 | 1.36E-66 | 213 |
| **SR-VP_0-2_scaffold_141_5191495_69|SR-VP_0-2cm_Phage_38_25|SR-VP_0-2cm** | SR-VP_2-4_scaffold_141_6357959_2|SR-VP_2-4cm_UNK|SR-VP_2-4cm | 50.000 | 190 | 95 | 0 | 1 | 190 | 1 | 190 | 3.09E-65 | 210 |
| **SR-VP_0-2_scaffold_141_5191495_69|SR-VP_0-2cm_Phage_38_25|SR-VP_0-2cm** | SRVP18_trench_5_20cm_scaffold_64130_2|SRVP18_trench_5_20cm_UNK|SRVP18_trench_5_20cm | 49.474 | 190 | 95 | 1 | 1 | 189 | 1 | 190 | 3.76E-65 | 210 |
| **SR-VP_0-2_scaffold_141_5191495_69|SR-VP_0-2cm_Phage_38_25|SR-VP_0-2cm** | U1s2_full_scaffold_39371_2|U1s2_UNK|U1s2 | 53.368 | 193 | 86 | 3 | 1 | 190 | 1 | 192 | 1.12E-63 | 206 |
| **SR-VP_0-2_scaffold_141_5191495_69|SR-VP_0-2cm_Phage_38_25|SR-VP_0-2cm** | SR-VP_0-2_scaffold_141_6969966_2|SR-VP_0-2cm_UNK|SR-VP_0-2cm | 48.947 | 190 | 97 | 0 | 1 | 190 | 1 | 190 | 1.84E-63 | 205 |
| **SR-VP_0-2_scaffold_141_5191495_69|SR-VP_0-2cm_Phage_38_25|SR-VP_0-2cm** | PAFVLPS_2018_scaffold_46833_7|viral-cat_UNK|viral-cat | 51.531 | 196 | 92 | 3 | 1 | 195 | 1 | 194 | 2.14E-63 | 205 |
| **SR-VP_0-2_scaffold_141_5191495_69|SR-VP_0-2cm_Phage_38_25|SR-VP_0-2cm** | ALT_03122018_0_1um_scaffold_29131_3|ALT_03122018_0_1um_UNK|ALT_03122018_0_1um | 47.917 | 192 | 98 | 2 | 1 | 190 | 1 | 192 | 2.25E-62 | 203 |
| **SR-VP_0-2_scaffold_141_5191495_69|SR-VP_0-2cm_Phage_38_25|SR-VP_0-2cm** | PAFVLPS_2018_scaffold_386_4|viral-cat_UNK|viral-cat | 51.256 | 199 | 94 | 3 | 1 | 198 | 1 | 197 | 1.04E-61 | 201 |
| **SR-VP_0-2_scaffold_141_5191495_69|SR-VP_0-2cm_Phage_38_25|SR-VP_0-2cm** | PAFVLPS_2018_J28_2_scaffold_23939_2|soil-virus-P12-J28-2018_UNK|soil-virus-P12-J28-2018 | 51.256 | 199 | 94 | 3 | 1 | 198 | 1 | 197 | 1.04E-61 | 201 |
| **SR-VP_0-2_scaffold_141_5191495_69|SR-VP_0-2cm_Phage_38_25|SR-VP_0-2cm** | L3m2_full_idba_ud_scaffold_11238_3|L3m2_concoct_70|L3m2 | 45.550 | 191 | 103 | 1 | 1 | 190 | 1 | 191 | 1.37E-55 | 186 |
| **SR-VP_0-2_scaffold_141_5191495_69|SR-VP_0-2cm_Phage_38_25|SR-VP_0-2cm** | L3m1_full_idba_ud_scaffold_1665_3|L3m1_UNK|L3m1 | 45.550 | 191 | 103 | 1 | 1 | 190 | 1 | 191 | 1.37E-55 | 186 |
| **SR-VP_0-2_scaffold_141_5191495_69|SR-VP_0-2cm_Phage_38_25|SR-VP_0-2cm** | Mad1_40_16_scaffold_318590_2|Mad1_40_16_UNK|Mad1_40_16 | 46.073 | 191 | 101 | 2 | 1 | 189 | 1 | 191 | 9.07E-55 | 183 |
| **SR-VP_0-2_scaffold_141_5191495_69|SR-VP_0-2cm_Phage_38_25|SR-VP_0-2cm** | H2c1_full_idba_ud_scaffold_224665_2|H2c1_UNK|H2c1 | 46.073 | 191 | 101 | 2 | 1 | 189 | 1 | 191 | 2.68E-53 | 179 |
| **SR-VP_0-2_scaffold_141_5191495_69|SR-VP_0-2cm_Phage_38_25|SR-VP_0-2cm** | H1a2_full_idba_ud_scaffold_87695_2|H1a2_UNK|H1a2 | 47.027 | 185 | 96 | 1 | 1 | 183 | 1 | 185 | 1.63E-52 | 177 |
| **SR-VP_0-2_scaffold_141_5191495_69|SR-VP_0-2cm_Phage_38_25|SR-VP_0-2cm** | PLM4_65_b1_redo_sep16_scaffold_449876_1|PLM4_65cm_b1_redo_sep2016_UNK|PLM4_65cm_b1_redo_sep2016 | 52.318 | 151 | 71 | 1 | 47 | 196 | 3 | 153 | 2.66E-49 | 168 |
| **SR-VP_0-2_scaffold_141_5191495_69|SR-VP_0-2cm_Phage_38_25|SR-VP_0-2cm** | SR-VP_4-6_scaffold_141_2744295_1|SR-VP_4-6cm_Biohub_180515_UNK|SR-VP_4-6cm_Biohub_180515 | 100.000 | 80 | 0 | 0 | 125 | 204 | 1 | 80 | 1.95E-47 | 160 |
| **SR-VP_0-2_scaffold_141_5191495_69|SR-VP_0-2cm_Phage_38_25|SR-VP_0-2cm** | ERMGT418_2_scaffold_19603_1|ERMGT418_2_UNK|ERMGT418_2 | 44.643 | 168 | 93 | 0 | 17 | 184 | 210 | 377 | 1.33E-45 | 166 |
| **SR-VP_0-2_scaffold_141_5191495_69|SR-VP_0-2cm_Phage_38_25|SR-VP_0-2cm** | ERMZT517_2_scaffold_27826_2|ERMZT517_2_UNK|ERMZT517_2 | 47.879 | 165 | 86 | 0 | 26 | 190 | 274 | 438 | 2.6E-45 | 166 |
| **SR-VP_0-2_scaffold_141_5191495_69|SR-VP_0-2cm_Phage_38_25|SR-VP_0-2cm** | S16_GE16_scaffold_5545_prodigal-single_31|GD_PHAGE_46_9|E_GD2017-2_urea-3_S16_GE16_Biohub_180515 | 42.932 | 191 | 106 | 3 | 1 | 190 | 1 | 189 | 1.29E-43 | 155 |
| **SR-VP_0-2_scaffold_141_5191495_69|SR-VP_0-2cm_Phage_38_25|SR-VP_0-2cm** | S16_GE16_scaffold_5545_30|E_GD2017-2_urea-3_S16_GE16_Biohub_170515_Phage-like_46_9|E_GD2017-2_urea-3_S16_GE16_Biohub_180515 | 42.932 | 191 | 106 | 3 | 1 | 190 | 1 | 189 | 1.29E-43 | 155 |
| **SR-VP_0-2_scaffold_141_5191495_69|SR-VP_0-2cm_Phage_38_25|SR-VP_0-2cm** | ERMZT366_2_scaffold_2856_3|ERMZT366_2_UNK|ERMZT366_2 | 52.985 | 134 | 63 | 0 | 1 | 134 | 1 | 134 | 2.09E-43 | 152 |
| **SR-VP_0-2_scaffold_141_5191495_69|SR-VP_0-2cm_Phage_38_25|SR-VP_0-2cm** | PAFVLPS_2018_scaffold_33_44|circular_33|viral-cat | 44.737 | 190 | 102 | 3 | 1 | 189 | 1 | 188 | 1.37E-42 | 153 |
| **SR-VP_0-2_scaffold_141_5191495_69|SR-VP_0-2cm_Phage_38_25|SR-VP_0-2cm** | S15_GE15_scaffold_39268_54|E_GD2017-2_urea-2_S15_GE15_Biohub_170515_UNK|E_GD2017-2_urea-2_S15_GE15_Biohub_180515 | 42.781 | 187 | 104 | 3 | 1 | 186 | 1 | 185 | 3.77E-42 | 152 |
| **SR-VP_0-2_scaffold_141_5191495_69|SR-VP_0-2cm_Phage_38_25|SR-VP_0-2cm** | PAFVLPS_2018_J30_2_scaffold_29892_2|soil-virus-P14-J30-2018_UNK|soil-virus-P14-J30-2018 | 44.737 | 190 | 102 | 3 | 1 | 189 | 1 | 188 | 1.07E-41 | 152 |
| **SR-VP_0-2_scaffold_141_5191495_69|SR-VP_0-2cm_Phage_38_25|SR-VP_0-2cm** | BC_09192017_0_5m_scaffold_15350_3|BC_09192017_0_5m_UNK|BC_09192017_0_5m | 42.614 | 176 | 96 | 3 | 7 | 178 | 4 | 178 | 4.7E-39 | 143 |
| **SR-VP_0-2_scaffold_141_5191495_69|SR-VP_0-2cm_Phage_38_25|SR-VP_0-2cm** | PAFVLPS_2018_scaffold_477083_1|viral-cat_UNK|viral-cat | 39.011 | 182 | 108 | 3 | 9 | 189 | 3 | 182 | 7.41E-36 | 135 |
| **SR-VP_0-2_scaffold_141_5191495_69|SR-VP_0-2cm_Phage_38_25|SR-VP_0-2cm** | PAFVLPS_2018_scaffold_18_354|viral-cat_UNK|viral-cat | 37.234 | 188 | 115 | 3 | 3 | 189 | 6 | 191 | 3.85E-35 | 134 |
| **SR-VP_0-2_scaffold_141_5191495_69|SR-VP_0-2cm_Phage_38_25|SR-VP_0-2cm** | PAFVLPS_2018_J30_2_scaffold_1899_5|soil-virus-P14-J30-2018_UNK|soil-virus-P14-J30-2018 | 37.234 | 188 | 115 | 3 | 3 | 189 | 6 | 191 | 3.85E-35 | 134 |
| **SR-VP_0-2_scaffold_141_5191495_69|SR-VP_0-2cm_Phage_38_25|SR-VP_0-2cm** | P0_An_pond3_S8_coassembly_k141_276347_5|P0_An_pond3_S8_coassembly_UNK|E_GD2017-1_P0_An_pond3_S8_Biohub_coassembly | 37.968 | 187 | 113 | 3 | 5 | 190 | 2 | 186 | 4.63E-33 | 128 |
| **SR-VP_0-2_scaffold_141_5191495_69|SR-VP_0-2cm_Phage_38_25|SR-VP_0-2cm** | P0_An_GD2017L_S7_coassembly_k141_961924_10|P0_An_GD2017L_S7_coassembly_UNK|E_P0_An_GD2017L_S7_coassembly | 37.968 | 187 | 113 | 3 | 5 | 190 | 2 | 186 | 4.63E-33 | 128 |
| **SR-VP_0-2_scaffold_141_5191495_69|SR-VP_0-2cm_Phage_38_25|SR-VP_0-2cm** | P0_An_pond3_S8_170907_scaffold_875447_5|E_GD2017-1_P0_AN_POND3_S8_BIOHUB_170907_UNK|E_GD2017-1_P0_An_pond3_S8_Biohub_170907 | 37.968 | 187 | 113 | 3 | 5 | 190 | 2 | 186 | 4.63E-33 | 128 |
| **SR-VP_0-2_scaffold_141_5191495_69|SR-VP_0-2cm_Phage_38_25|SR-VP_0-2cm** | P0_An_GD2017L_S7_170907_scaffold_2243732_5|E_GD2017-1_P0_An_GD2017L_S7_Biohub_170907_UNK|E_GD2017-1_P0_An_GD2017L_S7_Biohub_170907 | 37.968 | 187 | 113 | 3 | 5 | 190 | 2 | 186 | 1.38E-32 | 127 |
| **SR-VP_0-2_scaffold_141_5191495_69|SR-VP_0-2cm_Phage_38_25|SR-VP_0-2cm** | FFC_04162018_0_1um_scaffold_383_3|FFC_04162018_0_1um_UNK|FFC_04162018_0_1um | 36.702 | 188 | 114 | 3 | 7 | 190 | 9 | 195 | 4.6E-32 | 125 |
| **SR-VP_0-2_scaffold_141_5191495_69|SR-VP_0-2cm_Phage_38_25|SR-VP_0-2cm** | SR-VP_4-6_scaffold_141_2760307_3|SR-VP_4-6cm_Biohub_180515_UNK|SR-VP_4-6cm_Biohub_180515 | 36.683 | 199 | 124 | 2 | 1 | 198 | 1 | 198 | 9.39E-32 | 125 |
| **SR-VP_0-2_scaffold_141_5191495_69|SR-VP_0-2cm_Phage_38_25|SR-VP_0-2cm** | SR-VP_0-2_scaffold_141_955116_3|SR-VP_0-2cm_UNK|SR-VP_0-2cm | 36.683 | 199 | 124 | 2 | 1 | 198 | 1 | 198 | 9.39E-32 | 125 |
| **SR-VP_0-2_scaffold_141_5191495_69|SR-VP_0-2cm_Phage_38_25|SR-VP_0-2cm** | GWB1_scaffold_1082_3|GWB1 | 36.264 | 182 | 113 | 2 | 4 | 184 | 2 | 181 | 1.79E-31 | 124 |
| **SR-VP_0-2_scaffold_141_5191495_69|SR-VP_0-2cm_Phage_38_25|SR-VP_0-2cm** | BC_09192017_0_5m_scaffold_2201_5|BC_09192017_0_5m_UNK|BC_09192017_0_5m | 37.824 | 193 | 115 | 3 | 2 | 190 | 4 | 195 | 5.03E-31 | 123 |

| **ALT_09252017_20_scaffold_16_257|ALT_09252017_20_Phage_39_18|ALT_09252017_20** | ALT_03122018_0_1um_scaffold_3_102|ALT_03122018_0_1um_partial_phage_39_31|ALT_03122018_0_1um | 100.000 | 757 | 0 | 0 | 1 | 757 | 1 | 757 | 0.0 | 1543 |
| --- | --- | --- | --- | --- | --- | --- | --- | --- | --- | --- | --- |
| **ALT_09252017_20_scaffold_16_257|ALT_09252017_20_Phage_39_18|ALT_09252017_20** | ALT_082018_0_1um_scaffold_4834_11|ALT_082018_0_1um_UNK|ALT_082018_0_1um | 100.000 | 757 | 0 | 0 | 1 | 757 | 1 | 757 | 0.0 | 1543 |
| **ALT_09252017_20_scaffold_16_257|ALT_09252017_20_Phage_39_18|ALT_09252017_20** | ALT_09252017_20_scaffold_16_prodigal-single_259|ALT_PHAGE_39_18|ALT_09252017_20 | 100.000 | 757 | 0 | 0 | 1 | 757 | 1 | 757 | 0.0 | 1543 |
| **ALT_09252017_20_scaffold_16_257|ALT_09252017_20_Phage_39_18|ALT_09252017_20** | ALT_09252017_20_scaffold_16_257|ALT_09252017_20_Phage_39_18|ALT_09252017_20 | 100.000 | 757 | 0 | 0 | 1 | 757 | 1 | 757 | 0.0 | 1543 |
| **ALT_09252017_20_scaffold_16_257|ALT_09252017_20_Phage_39_18|ALT_09252017_20** | RHP_09252018_0_1um_scaffold_54_48|RHP_09252018_0_1um_UNK|RHP_09252018_0_1um | 93.527 | 757 | 49 | 0 | 1 | 757 | 1 | 757 | 0.0 | 1466 |
| **ALT_09252017_20_scaffold_16_257|ALT_09252017_20_Phage_39_18|ALT_09252017_20** | RHP_09252018_0_1um_scaffold_7414_5|RHP_09252018_0_1um_UNK|RHP_09252018_0_1um | 81.579 | 760 | 134 | 3 | 1 | 756 | 1 | 758 | 0.0 | 1297 |
| **ALT_09252017_20_scaffold_16_257|ALT_09252017_20_Phage_39_18|ALT_09252017_20** | RHP_09252018_0_1um_scaffold_1251_53|RHP_09252018_0_1um_UNK|RHP_09252018_0_1um | 76.882 | 757 | 173 | 2 | 1 | 755 | 1 | 757 | 0.0 | 1183 |
| **ALT_09252017_20_scaffold_16_257|ALT_09252017_20_Phage_39_18|ALT_09252017_20** | AB_092018_0_1um_scaffold_2615_2|AB_092018_0_1um_UNK|AB_092018_0_1um | 75.941 | 744 | 176 | 3 | 1 | 742 | 1 | 743 | 0.0 | 1170 |
| **ALT_09252017_20_scaffold_16_257|ALT_09252017_20_Phage_39_18|ALT_09252017_20** | FFC_07242016_10_scaffold_1071_4|FFC_07242016_10_UNK|FFC_07242016_10 | 74.177 | 759 | 189 | 4 | 1 | 755 | 1 | 756 | 0.0 | 1164 |
| **ALT_09252017_20_scaffold_16_257|ALT_09252017_20_Phage_39_18|ALT_09252017_20** | AB_072018_0_1um_scaffold_817_8|AB_072018_0_1um_UNK|AB_072018_0_1um | 75.066 | 758 | 180 | 6 | 1 | 754 | 1 | 753 | 0.0 | 1155 |
| **ALT_09252017_20_scaffold_16_257|ALT_09252017_20_Phage_39_18|ALT_09252017_20** | ALT_072018_0_1um_scaffold_58_97|ALT_072018_0_1um_UNK|ALT_072018_0_1um | 75.729 | 754 | 181 | 2 | 1 | 752 | 1 | 754 | 0.0 | 1151 |
| **ALT_09252017_20_scaffold_16_257|ALT_09252017_20_Phage_39_18|ALT_09252017_20** | ALT_03122018_0_1um_scaffold_1_163|ALT_03122018_0_1um_potentially_complete_phage_41_17|ALT_03122018_0_1um | 75.729 | 754 | 181 | 2 | 1 | 752 | 1 | 754 | 0.0 | 1151 |
| **ALT_09252017_20_scaffold_16_257|ALT_09252017_20_Phage_39_18|ALT_09252017_20** | AB_072018_0_1um_scaffold_33_39|AB_072018_0_1um_UNK|AB_072018_0_1um | 75.729 | 754 | 181 | 2 | 1 | 752 | 1 | 754 | 0.0 | 1151 |
| **ALT_09252017_20_scaffold_16_257|ALT_09252017_20_Phage_39_18|ALT_09252017_20** | ALT_082018_0_1um_scaffold_29_7|ALT_082018_0_1um_Huge_Phage_41_11|ALT_082018_0_1um | 75.729 | 754 | 181 | 2 | 1 | 752 | 1 | 754 | 0.0 | 1151 |
| **ALT_09252017_20_scaffold_16_257|ALT_09252017_20_Phage_39_18|ALT_09252017_20** | FFC_07242016_10_scaffold_945_6|FFC_07242016_10_UNK|FFC_07242016_10 | 71.846 | 753 | 207 | 3 | 1 | 750 | 1 | 751 | 0.0 | 1128 |
| **ALT_09252017_20_scaffold_16_257|ALT_09252017_20_Phage_39_18|ALT_09252017_20** | BC_09192017_0_5m_scaffold_41_68|BC_09192017_0_5m_UNK|BC_09192017_0_5m | 69.169 | 746 | 229 | 1 | 1 | 745 | 1 | 746 | 0.0 | 1105 |
| **ALT_09252017_20_scaffold_16_257|ALT_09252017_20_Phage_39_18|ALT_09252017_20** | LacPavin_0818_WC50_scaffold_160460_3|LacPavin_0818_WC50_UNK|LacPavin_0818_WC50 | 72.840 | 648 | 174 | 1 | 1 | 646 | 1 | 648 | 0.0 | 1005 |
| **ALT_09252017_20_scaffold_16_257|ALT_09252017_20_Phage_39_18|ALT_09252017_20** | Salt_Pond_SF2_C_H2O_MG_scaffold_5179_4|JGI_Salt_Pond_SF2_C_H2O_MG_UNK|JGI_Salt_Pond_SF2_C_H2O_MG | 67.308 | 728 | 235 | 3 | 1 | 726 | 1 | 727 | 0.0 | 991 |
| **ALT_09252017_20_scaffold_16_257|ALT_09252017_20_Phage_39_18|ALT_09252017_20** | ALT_03122018_0_1um_scaffold_5949_4|ALT_03122018_0_1um_UNK|ALT_03122018_0_1um | 61.364 | 748 | 280 | 4 | 1 | 739 | 1 | 748 | 0.0 | 976 |
| **ALT_09252017_20_scaffold_16_257|ALT_09252017_20_Phage_39_18|ALT_09252017_20** | RHP_09252018_0_1um_scaffold_2296_12|RHP_09252018_0_1um_UNK|RHP_09252018_0_1um | 60.561 | 748 | 286 | 4 | 1 | 739 | 1 | 748 | 0.0 | 974 |
| **ALT_09252017_20_scaffold_16_257|ALT_09252017_20_Phage_39_18|ALT_09252017_20** | ALT_04162018_0_2um_scaffold_986_25|ALT_04162018_0_2um_UNK|ALT_04162018_0_2um | 60.963 | 748 | 283 | 4 | 1 | 739 | 1 | 748 | 0.0 | 972 |
| **ALT_09252017_20_scaffold_16_257|ALT_09252017_20_Phage_39_18|ALT_09252017_20** | ALT_03122018_0_1um_scaffold_2951_8|ALT_03122018_0_1um_UNK|ALT_03122018_0_1um | 60.963 | 748 | 283 | 4 | 1 | 739 | 1 | 748 | 0.0 | 972 |
| **ALT_09252017_20_scaffold_16_257|ALT_09252017_20_Phage_39_18|ALT_09252017_20** | ALT_09252017_20_scaffold_265_237|ALT_09252017_20_Phage_42_25|ALT_09252017_20 | 60.963 | 748 | 283 | 4 | 1 | 739 | 1 | 748 | 0.0 | 971 |
| **ALT_09252017_20_scaffold_16_257|ALT_09252017_20_Phage_39_18|ALT_09252017_20** | LacPavin_0818_WC50_scaffold_931777_24|LacPavin_0818_WC50_UNK|LacPavin_0818_WC50 | 60.829 | 748 | 284 | 4 | 1 | 739 | 1 | 748 | 0.0 | 970 |
| **ALT_09252017_20_scaffold_16_257|ALT_09252017_20_Phage_39_18|ALT_09252017_20** | LacPavin_0818_WC40_scaffold_456509_5|LacPavin_0818_WC40_UNK|LacPavin_0818_WC40 | 60.829 | 748 | 284 | 4 | 1 | 739 | 1 | 748 | 0.0 | 969 |
| **ALT_09252017_20_scaffold_16_257|ALT_09252017_20_Phage_39_18|ALT_09252017_20** | BC_09192017_0_5m_scaffold_16751_4|BC_09192017_0_5m_UNK|BC_09192017_0_5m | 61.497 | 748 | 279 | 6 | 1 | 739 | 1 | 748 | 0.0 | 967 |
| **ALT_09252017_20_scaffold_16_257|ALT_09252017_20_Phage_39_18|ALT_09252017_20** | ALT_072018_0_1um_scaffold_6488_12|ALT_072018_0_1um_UNK|ALT_072018_0_1um | 60.133 | 750 | 289 | 5 | 1 | 740 | 1 | 750 | 0.0 | 954 |
| **ALT_09252017_20_scaffold_16_257|ALT_09252017_20_Phage_39_18|ALT_09252017_20** | ALT_03122018_0_1um_scaffold_3035_5|ALT_03122018_0_1um_UNK|ALT_03122018_0_1um | 60.586 | 751 | 284 | 4 | 1 | 739 | 1 | 751 | 0.0 | 952 |
| **ALT_09252017_20_scaffold_16_257|ALT_09252017_20_Phage_39_18|ALT_09252017_20** | ALT_072018_0_1um_scaffold_6894_4|ALT_072018_0_1um_UNK|ALT_072018_0_1um | 61.119 | 751 | 279 | 5 | 1 | 739 | 1 | 750 | 0.0 | 949 |
| **ALT_09252017_20_scaffold_16_257|ALT_09252017_20_Phage_39_18|ALT_09252017_20** | ALT_082018_0_1um_scaffold_5715_7|ALT_082018_0_1um_UNK|ALT_082018_0_1um | 61.119 | 751 | 279 | 5 | 1 | 739 | 1 | 750 | 0.0 | 949 |
| **ALT_09252017_20_scaffold_16_257|ALT_09252017_20_Phage_39_18|ALT_09252017_20** | BML_08022017_1_5m_scaffold_117_26|BML_08022017_1_5m_UNK|BML_08022017_1_5m | 62.148 | 745 | 269 | 7 | 1 | 734 | 1 | 743 | 0.0 | 949 |
| **ALT_09252017_20_scaffold_16_257|ALT_09252017_20_Phage_39_18|ALT_09252017_20** | ALT_03122018_0_1um_scaffold_33_100|ALT_03122018_0_1um_UNK|ALT_03122018_0_1um | 59.192 | 767 | 299 | 6 | 1 | 755 | 1 | 765 | 0.0 | 946 |
| **ALT_09252017_20_scaffold_16_257|ALT_09252017_20_Phage_39_18|ALT_09252017_20** | BML_08022017_1_5m_scaffold_40_235|BML_08022017_1_5m_UNK|BML_08022017_1_5m | 61.194 | 737 | 275 | 5 | 1 | 727 | 1 | 736 | 0.0 | 946 |
| **ALT_09252017_20_scaffold_16_257|ALT_09252017_20_Phage_39_18|ALT_09252017_20** | BML_coassembly_scaffold_3077_61|BML_coassembly_UNK|BML_coassembly | 61.194 | 737 | 275 | 5 | 1 | 727 | 1 | 736 | 0.0 | 946 |
| **ALT_09252017_20_scaffold_16_257|ALT_09252017_20_Phage_39_18|ALT_09252017_20** | BML_08022017_6_5m_scaffold_11_68|BML_08022017_6_5m_UNK|BML_08022017_6_5m | 61.058 | 737 | 276 | 5 | 1 | 727 | 1 | 736 | 0.0 | 944 |
| **ALT_09252017_20_scaffold_16_257|ALT_09252017_20_Phage_39_18|ALT_09252017_20** | BML_08022017_1_5m_scaffold_145_37|BML_08022017_1_5m_UNK|BML_08022017_1_5m | 59.001 | 761 | 301 | 4 | 1 | 752 | 1 | 759 | 0.0 | 937 |
| **ALT_09252017_20_scaffold_16_257|ALT_09252017_20_Phage_39_18|ALT_09252017_20** | BML_coassembly_scaffold_7136_14|BML_coassembly_UNK|BML_coassembly | 59.001 | 761 | 301 | 4 | 1 | 752 | 1 | 759 | 0.0 | 937 |
| **ALT_09252017_20_scaffold_16_257|ALT_09252017_20_Phage_39_18|ALT_09252017_20** | Salt_Pond_SF2_A_H2O_MG_scaffold_5044_3|JGI_Salt_Pond_SF2_A_H2O_MG_UNK|JGI_Salt_Pond_SF2_A_H2O_MG | 63.599 | 728 | 260 | 3 | 1 | 726 | 1 | 725 | 0.0 | 934 |
| **ALT_09252017_20_scaffold_16_257|ALT_09252017_20_Phage_39_18|ALT_09252017_20** | Salt_Pond_R2_restored_H2O_MG_scaffold_286_26|JGI_Salt_Pond_R2_restored_H2O_MG_UNK|JGI_Salt_Pond_R2_restored_H2O_MG | 59.839 | 747 | 285 | 7 | 1 | 734 | 1 | 745 | 0.0 | 934 |
| **ALT_09252017_20_scaffold_16_257|ALT_09252017_20_Phage_39_18|ALT_09252017_20** | Salt_Pond_R2_restored_H2O_MG_scaffold_17_98|JGI_Salt_Pond_R2_restored_H2O_MG_UNK|JGI_Salt_Pond_R2_restored_H2O_MG | 58.257 | 769 | 304 | 7 | 1 | 757 | 1 | 764 | 0.0 | 933 |
| **ALT_09252017_20_scaffold_16_257|ALT_09252017_20_Phage_39_18|ALT_09252017_20** | ALT_072018_0_1um_scaffold_94_90|ALT_072018_0_1um_UNK|ALT_072018_0_1um | 58.902 | 747 | 299 | 3 | 1 | 739 | 1 | 747 | 0.0 | 931 |
| **ALT_09252017_20_scaffold_16_257|ALT_09252017_20_Phage_39_18|ALT_09252017_20** | ALT_03122018_0_1um_scaffold_1175_21|ALT_03122018_0_1um_UNK|ALT_03122018_0_1um | 58.902 | 747 | 299 | 3 | 1 | 739 | 1 | 747 | 0.0 | 931 |
| **ALT_09252017_20_scaffold_16_257|ALT_09252017_20_Phage_39_18|ALT_09252017_20** | ALT_082018_0_1um_scaffold_114_144|ALT_082018_0_1um_UNK|ALT_082018_0_1um | 58.902 | 747 | 299 | 3 | 1 | 739 | 1 | 747 | 0.0 | 931 |
| **ALT_09252017_20_scaffold_16_257|ALT_09252017_20_Phage_39_18|ALT_09252017_20** | BML_05172017_7_5m_scaffold_95_33|BML_05172017_7_5m_UNK|BML_05172017_7_5m | 58.589 | 751 | 302 | 3 | 1 | 742 | 1 | 751 | 0.0 | 930 |
| **ALT_09252017_20_scaffold_16_257|ALT_09252017_20_Phage_39_18|ALT_09252017_20** | LacPavin_0818_WC50_scaffold_712805_32|LacPavin_0818_WC50_UNK|LacPavin_0818_WC50 | 60.627 | 734 | 281 | 5 | 1 | 727 | 1 | 733 | 0.0 | 928 |
| **ALT_09252017_20_scaffold_16_257|ALT_09252017_20_Phage_39_18|ALT_09252017_20** | LacPavin_0818_WC40_scaffold_705268_32|LacPavin_0818_WC40_UNK|LacPavin_0818_WC40 | 60.627 | 734 | 281 | 5 | 1 | 727 | 1 | 733 | 0.0 | 928 |
| **ALT_09252017_20_scaffold_16_257|ALT_09252017_20_Phage_39_18|ALT_09252017_20** | AB_072018_0_1um_scaffold_499_27|AB_072018_0_1um_UNK|AB_072018_0_1um | 58.411 | 755 | 298 | 4 | 1 | 739 | 1 | 755 | 0.0 | 926 |
| **ALT_09252017_20_scaffold_16_257|ALT_09252017_20_Phage_39_18|ALT_09252017_20** | Salt_Pond_SF2_C_H2O_MG_scaffold_14149_2|JGI_Salt_Pond_SF2_C_H2O_MG_UNK|JGI_Salt_Pond_SF2_C_H2O_MG | 60.594 | 741 | 281 | 5 | 1 | 730 | 1 | 741 | 0.0 | 926 |
| **ALT_09252017_20_scaffold_16_257|ALT_09252017_20_Phage_39_18|ALT_09252017_20** | BML_06132017_6_25m_scaffold_5771_3|BML_06132017_6_25m_UNK|BML_06132017_6_25m | 58.511 | 752 | 302 | 3 | 1 | 742 | 1 | 752 | 0.0 | 925 |
| **ALT_09252017_20_scaffold_16_257|ALT_09252017_20_Phage_39_18|ALT_09252017_20** | BML_08022017_6_5m_scaffold_525_17|BML_08022017_6_5m_UNK|BML_08022017_6_5m | 58.511 | 752 | 302 | 3 | 1 | 742 | 1 | 752 | 0.0 | 925 |
| **ALT_09252017_20_scaffold_16_257|ALT_09252017_20_Phage_39_18|ALT_09252017_20** | BML_08022017_1_5m_scaffold_73_39|BML_08022017_1_5m_Fragmented_Jumbo_Phage_39_24|BML_08022017_1_5m | 58.511 | 752 | 302 | 3 | 1 | 742 | 1 | 752 | 0.0 | 925 |
| **ALT_09252017_20_scaffold_16_257|ALT_09252017_20_Phage_39_18|ALT_09252017_20** | BML_08042016_6_5m_scaffold_6_49|BML_08042016_6_5m_Fragmented_Jumbo_Phage_39_11|BML_08042016_6_5m | 58.511 | 752 | 302 | 3 | 1 | 742 | 1 | 752 | 0.0 | 925 |
| **ALT_09252017_20_scaffold_16_257|ALT_09252017_20_Phage_39_18|ALT_09252017_20** | BML_02172017_6_5m_scaffold_2082_4|BML_02172017_6_5m_UNK|BML_02172017_6_5m | 58.511 | 752 | 302 | 3 | 1 | 742 | 1 | 752 | 0.0 | 925 |
| **ALT_09252017_20_scaffold_16_257|ALT_09252017_20_Phage_39_18|ALT_09252017_20** | BML_08182015_8_5m_scaffold_0_prodigal-single_38|BML_PHAGE_39_34|BML_08182015_8_5m | 58.511 | 752 | 302 | 3 | 1 | 742 | 1 | 752 | 0.0 | 925 |
| **ALT_09252017_20_scaffold_16_257|ALT_09252017_20_Phage_39_18|ALT_09252017_20** | BML_08182015_8_5m_scaffold_0_38|BML_08182015_8_5m_Fragmented_Jumbo_Phage_39_34|BML_08182015_8_5m | 58.511 | 752 | 302 | 3 | 1 | 742 | 1 | 752 | 0.0 | 925 |
| **ALT_09252017_20_scaffold_16_257|ALT_09252017_20_Phage_39_18|ALT_09252017_20** | BML_08182015_6_5m_scaffold_6_prodigal-single_43|BML_PHAGE_39_25|BML_08182015_6_5m | 58.511 | 752 | 302 | 3 | 1 | 742 | 1 | 752 | 0.0 | 925 |
| **ALT_09252017_20_scaffold_16_257|ALT_09252017_20_Phage_39_18|ALT_09252017_20** | BML_08182015_6_5m_scaffold_6_43|BML_08182015_6_5m_Phage_39_25|BML_08182015_6_5m | 58.511 | 752 | 302 | 3 | 1 | 742 | 1 | 752 | 0.0 | 925 |
| **ALT_09252017_20_scaffold_16_257|ALT_09252017_20_Phage_39_18|ALT_09252017_20** | BML_08182015_1_5m_scaffold_5_prodigal-single_210|BML_PHAGE_39_22|BML_08182015_1_5m | 58.511 | 752 | 302 | 3 | 1 | 742 | 1 | 752 | 0.0 | 925 |
| **ALT_09252017_20_scaffold_16_257|ALT_09252017_20_Phage_39_18|ALT_09252017_20** | BML_08182015_1_5m_scaffold_5_207|BML_08182015_1_5m_Incomplete_Jumbo_Phage_39_22|BML_08182015_1_5m | 58.511 | 752 | 302 | 3 | 1 | 742 | 1 | 752 | 0.0 | 925 |
| **ALT_09252017_20_scaffold_16_257|ALT_09252017_20_Phage_39_18|ALT_09252017_20** | BML_coassembly_scaffold_6354_13|BML_coassembly_UNK|BML_coassembly | 58.511 | 752 | 302 | 3 | 1 | 742 | 1 | 752 | 0.0 | 925 |
| **ALT_09252017_20_scaffold_16_257|ALT_09252017_20_Phage_39_18|ALT_09252017_20** | Salt_Pond_R1_B_H2O_MG_scaffold_1876_3|JGI_Salt_Pond_R1_B_H2O_MG_UNK|JGI_Salt_Pond_R1_B_H2O_MG | 59.264 | 734 | 295 | 2 | 1 | 730 | 1 | 734 | 0.0 | 924 |
| **ALT_09252017_20_scaffold_16_257|ALT_09252017_20_Phage_39_18|ALT_09252017_20** | BML_02132018_6_5m_scaffold_4518_3|BML_02132018_6_5m_UNK|BML_02132018_6_5m | 58.910 | 752 | 296 | 6 | 1 | 740 | 1 | 751 | 0.0 | 923 |
| **ALT_09252017_20_scaffold_16_257|ALT_09252017_20_Phage_39_18|ALT_09252017_20** | Salt_Pond_R2_restored_H2O_MG_scaffold_6014_2|JGI_Salt_Pond_R2_restored_H2O_MG_UNK|JGI_Salt_Pond_R2_restored_H2O_MG | 57.847 | 771 | 304 | 8 | 1 | 757 | 1 | 764 | 0.0 | 923 |
| **ALT_09252017_20_scaffold_16_257|ALT_09252017_20_Phage_39_18|ALT_09252017_20** | BML_coassembly_scaffold_1849_31|BML_coassembly_UNK|BML_coassembly | 58.910 | 752 | 296 | 6 | 1 | 740 | 1 | 751 | 0.0 | 923 |
| **ALT_09252017_20_scaffold_16_257|ALT_09252017_20_Phage_39_18|ALT_09252017_20** | Salt_Pond_R2_restored_H2O_MG_scaffold_262_10|JGI_Salt_Pond_R2_restored_H2O_MG_UNK|JGI_Salt_Pond_R2_restored_H2O_MG | 59.128 | 734 | 296 | 2 | 1 | 730 | 1 | 734 | 0.0 | 922 |
| **ALT_09252017_20_scaffold_16_257|ALT_09252017_20_Phage_39_18|ALT_09252017_20** | BML_coassembly_scaffold_1045_29|BML_coassembly_UNK|BML_coassembly | 59.426 | 732 | 289 | 4 | 1 | 724 | 1 | 732 | 0.0 | 920 |
| **ALT_09252017_20_scaffold_16_257|ALT_09252017_20_Phage_39_18|ALT_09252017_20** | Salt_Pond_SF2_B_H2O_MG_scaffold_11288_2|JGI_Salt_Pond_SF2_B_H2O_MG_UNK|JGI_Salt_Pond_SF2_B_H2O_MG | 60.185 | 756 | 288 | 6 | 1 | 749 | 1 | 750 | 0.0 | 915 |
| **ALT_09252017_20_scaffold_16_257|ALT_09252017_20_Phage_39_18|ALT_09252017_20** | BML_08182015_8_5m_scaffold_2742_5|BML_08182015_8_5m_UNK|BML_08182015_8_5m | 57.831 | 747 | 307 | 5 | 1 | 739 | 1 | 747 | 0.0 | 913 |
| **ALT_09252017_20_scaffold_16_257|ALT_09252017_20_Phage_39_18|ALT_09252017_20** | BML_coassembly_scaffold_373_110|BML_coassembly_UNK|BML_coassembly | 57.831 | 747 | 307 | 5 | 1 | 739 | 1 | 747 | 0.0 | 913 |
| **ALT_09252017_20_scaffold_16_257|ALT_09252017_20_Phage_39_18|ALT_09252017_20** | RHP_09252018_0_1um_scaffold_1856_28|RHP_09252018_0_1um_UNK|RHP_09252018_0_1um | 60.490 | 734 | 282 | 4 | 1 | 730 | 1 | 730 | 0.0 | 912 |
| **ALT_09252017_20_scaffold_16_257|ALT_09252017_20_Phage_39_18|ALT_09252017_20** | BML_06132017_6_25m_scaffold_4342_2|BML_06132017_6_25m_UNK|BML_06132017_6_25m | 58.791 | 728 | 299 | 1 | 1 | 727 | 1 | 728 | 0.0 | 912 |
| **ALT_09252017_20_scaffold_16_257|ALT_09252017_20_Phage_39_18|ALT_09252017_20** | BC_09192017_0_5m_scaffold_45030_2|BC_09192017_0_5m_UNK|BC_09192017_0_5m | 58.611 | 749 | 278 | 5 | 1 | 739 | 1 | 727 | 0.0 | 911 |
| **ALT_09252017_20_scaffold_16_257|ALT_09252017_20_Phage_39_18|ALT_09252017_20** | BML_02132018_6_5m_scaffold_175_60|BML_02132018_6_5m_UNK|BML_02132018_6_5m | 59.085 | 743 | 290 | 5 | 1 | 731 | 1 | 741 | 0.0 | 907 |
| **ALT_09252017_20_scaffold_16_257|ALT_09252017_20_Phage_39_18|ALT_09252017_20** | BML_08022017_1_5m_scaffold_839_15|BML_08022017_1_5m_UNK|BML_08022017_1_5m | 59.085 | 743 | 290 | 5 | 1 | 731 | 1 | 741 | 0.0 | 907 |
| **ALT_09252017_20_scaffold_16_257|ALT_09252017_20_Phage_39_18|ALT_09252017_20** | BML_coassembly_scaffold_4017_31|BML_coassembly_UNK|BML_coassembly | 59.085 | 743 | 290 | 5 | 1 | 731 | 1 | 741 | 0.0 | 907 |
| **ALT_09252017_20_scaffold_16_257|ALT_09252017_20_Phage_39_18|ALT_09252017_20** | BML_02132018_6_5m_scaffold_16491_1|BML_02132018_6_5m_UNK|BML_02132018_6_5m | 62.270 | 705 | 254 | 6 | 1 | 695 | 1 | 703 | 0.0 | 905 |
| **ALT_09252017_20_scaffold_16_257|ALT_09252017_20_Phage_39_18|ALT_09252017_20** | BC_09192017_0_5m_scaffold_18980_1|BC_09192017_0_5m_UNK|BC_09192017_0_5m | 62.300 | 687 | 250 | 5 | 63 | 741 | 1 | 686 | 0.0 | 904 |
| **ALT_09252017_20_scaffold_16_257|ALT_09252017_20_Phage_39_18|ALT_09252017_20** | LacPavin_0818_WC50_scaffold_136821_15|LacPavin_0818_WC50_UNK|LacPavin_0818_WC50 | 60.168 | 713 | 272 | 5 | 39 | 739 | 3 | 715 | 0.0 | 904 |
| **ALT_09252017_20_scaffold_16_257|ALT_09252017_20_Phage_39_18|ALT_09252017_20** | RHP_09252018_0_1um_scaffold_5773_12|RHP_09252018_0_1um_UNK|RHP_09252018_0_1um | 59.946 | 734 | 286 | 4 | 1 | 730 | 1 | 730 | 0.0 | 901 |
| **ALT_09252017_20_scaffold_16_257|ALT_09252017_20_Phage_39_18|ALT_09252017_20** | BML_05172017_7_5m_scaffold_762_7|BML_05172017_7_5m_UNK|BML_05172017_7_5m | 57.200 | 750 | 318 | 3 | 1 | 749 | 1 | 748 | 0.0 | 899 |
| **ALT_09252017_20_scaffold_16_257|ALT_09252017_20_Phage_39_18|ALT_09252017_20** | BML_coassembly_scaffold_6745_8|BML_coassembly_UNK|BML_coassembly | 57.200 | 750 | 318 | 3 | 1 | 749 | 1 | 748 | 0.0 | 899 |
| **ALT_09252017_20_scaffold_16_257|ALT_09252017_20_Phage_39_18|ALT_09252017_20** | Salt_Pond_R2_A_D1_MG_scaffold_4964_14|JGI_Salt_Pond_R2_A_D1_MG_UNK|JGI_Salt_Pond_R2_A_D1_MG | 59.350 | 738 | 287 | 8 | 1 | 731 | 1 | 732 | 0.0 | 896 |
| **ALT_09252017_20_scaffold_16_257|ALT_09252017_20_Phage_39_18|ALT_09252017_20** | Salt_Pond_R1_C_H2O_MG_scaffold_143_12|JGI_Salt_Pond_R1_C_H2O_MG_UNK|JGI_Salt_Pond_R1_C_H2O_MG | 58.016 | 736 | 299 | 3 | 1 | 726 | 1 | 736 | 0.0 | 895 |
| **ALT_09252017_20_scaffold_16_257|ALT_09252017_20_Phage_39_18|ALT_09252017_20** | Salt_Pond_R1_B_H2O_MG_scaffold_150_43|JGI_Salt_Pond_R1_B_H2O_MG_UNK|JGI_Salt_Pond_R1_B_H2O_MG | 58.016 | 736 | 299 | 3 | 1 | 726 | 1 | 736 | 0.0 | 895 |
| **ALT_09252017_20_scaffold_16_257|ALT_09252017_20_Phage_39_18|ALT_09252017_20** | Salt_Pond_R1_A_H2O_MG_scaffold_88_46|JGI_Salt_Pond_R1_A_H2O_MG_UNK|JGI_Salt_Pond_R1_A_H2O_MG | 58.016 | 736 | 299 | 3 | 1 | 726 | 1 | 736 | 0.0 | 895 |
| **ALT_09252017_20_scaffold_16_257|ALT_09252017_20_Phage_39_18|ALT_09252017_20** | Salt_Pond_R1_C_H2O_MG_scaffold_445_30|JGI_Salt_Pond_R1_C_H2O_MG_UNK|JGI_Salt_Pond_R1_C_H2O_MG | 59.214 | 738 | 288 | 8 | 1 | 731 | 1 | 732 | 0.0 | 894 |
| **ALT_09252017_20_scaffold_16_257|ALT_09252017_20_Phage_39_18|ALT_09252017_20** | BML_08022017_1_5m_scaffold_58_39|BML_08022017_1_5m_UNK|BML_08022017_1_5m | 60.272 | 735 | 282 | 5 | 1 | 730 | 1 | 730 | 0.0 | 894 |
| **ALT_09252017_20_scaffold_16_257|ALT_09252017_20_Phage_39_18|ALT_09252017_20** | BML_05172017_7_5m_scaffold_12_67|BML_05172017_7_5m_UNK|BML_05172017_7_5m | 60.272 | 735 | 282 | 5 | 1 | 730 | 1 | 730 | 0.0 | 894 |
| **ALT_09252017_20_scaffold_16_257|ALT_09252017_20_Phage_39_18|ALT_09252017_20** | BML_02172017_6_5m_scaffold_25_70|BML_02172017_6_5m_UNK|BML_02172017_6_5m | 60.272 | 735 | 282 | 5 | 1 | 730 | 1 | 730 | 0.0 | 894 |
| **ALT_09252017_20_scaffold_16_257|ALT_09252017_20_Phage_39_18|ALT_09252017_20** | BML_02172017_0m_scaffold_60_96|BML_02172017_0m_UNK|BML_02172017_0m | 60.272 | 735 | 282 | 5 | 1 | 730 | 1 | 730 | 0.0 | 894 |
| **ALT_09252017_20_scaffold_16_257|ALT_09252017_20_Phage_39_18|ALT_09252017_20** | Salt_Pond_R2_restored_H2O_MG_scaffold_241_9|JGI_Salt_Pond_R2_restored_H2O_MG_UNK|JGI_Salt_Pond_R2_restored_H2O_MG | 58.583 | 734 | 296 | 3 | 1 | 726 | 1 | 734 | 0.0 | 894 |
| **ALT_09252017_20_scaffold_16_257|ALT_09252017_20_Phage_39_18|ALT_09252017_20** | Salt_Pond_R2_restored_H2O_MG_scaffold_10_4|JGI_Salt_Pond_R2_restored_H2O_MG_UNK|JGI_Salt_Pond_R2_restored_H2O_MG | 57.296 | 747 | 316 | 3 | 1 | 746 | 1 | 745 | 0.0 | 894 |
| **ALT_09252017_20_scaffold_16_257|ALT_09252017_20_Phage_39_18|ALT_09252017_20** | BML_coassembly_scaffold_344_91|BML_coassembly_UNK|BML_coassembly | 60.272 | 735 | 282 | 5 | 1 | 730 | 1 | 730 | 0.0 | 894 |
| **ALT_09252017_20_scaffold_16_257|ALT_09252017_20_Phage_39_18|ALT_09252017_20** | BC_09192017_0_5m_scaffold_605_26|BC_09192017_0_5m_UNK|BC_09192017_0_5m | 57.616 | 755 | 312 | 5 | 1 | 754 | 1 | 748 | 0.0 | 889 |
| **ALT_09252017_20_scaffold_16_257|ALT_09252017_20_Phage_39_18|ALT_09252017_20** | AB_072018_0_1um_scaffold_1787_5|AB_072018_0_1um_UNK|AB_072018_0_1um | 58.516 | 728 | 297 | 4 | 1 | 727 | 1 | 724 | 0.0 | 888 |
| **ALT_09252017_20_scaffold_16_257|ALT_09252017_20_Phage_39_18|ALT_09252017_20** | ALT_07252016_14_scaffold_200_26|ALT_07252016_14_UNK|ALT_07252016_14 | 57.319 | 731 | 307 | 3 | 1 | 730 | 1 | 727 | 0.0 | 887 |
| **ALT_09252017_20_scaffold_16_257|ALT_09252017_20_Phage_39_18|ALT_09252017_20** | ALT_072018_0_1um_scaffold_481_67|ALT_072018_0_1um_UNK|ALT_072018_0_1um | 57.319 | 731 | 307 | 3 | 1 | 730 | 1 | 727 | 0.0 | 887 |
| **ALT_09252017_20_scaffold_16_257|ALT_09252017_20_Phage_39_18|ALT_09252017_20** | ALT_04162018_0_2um_scaffold_851_12|ALT_04162018_0_2um_UNK|ALT_04162018_0_2um | 56.283 | 748 | 317 | 4 | 1 | 747 | 1 | 739 | 0.0 | 887 |
| **ALT_09252017_20_scaffold_16_257|ALT_09252017_20_Phage_39_18|ALT_09252017_20** | ALT_04162018_0_2um_scaffold_238_21|ALT_04162018_0_2um_UNK|ALT_04162018_0_2um | 57.319 | 731 | 307 | 3 | 1 | 730 | 1 | 727 | 0.0 | 887 |
| **ALT_09252017_20_scaffold_16_257|ALT_09252017_20_Phage_39_18|ALT_09252017_20** | ALT_03122018_0_2um_scaffold_722_26|ALT_03122018_0_2um_UNK|ALT_03122018_0_2um | 57.319 | 731 | 307 | 3 | 1 | 730 | 1 | 727 | 0.0 | 887 |
| **ALT_09252017_20_scaffold_16_256|ALT_09252017_20_Phage_39_18|ALT_09252017_20** | ALT_072018_0_1um_scaffold_39118_2|ALT_072018_0_1um_UNK|ALT_072018_0_1um | 100.000 | 170 | 0 | 0 | 1 | 170 | 2 | 171 | 3.14E-124 | 357 |
| **ALT_09252017_20_scaffold_16_256|ALT_09252017_20_Phage_39_18|ALT_09252017_20** | ALT_03122018_0_1um_scaffold_3_101|ALT_03122018_0_1um_partial_phage_39_31|ALT_03122018_0_1um | 100.000 | 170 | 0 | 0 | 1 | 170 | 1 | 170 | 4.6E-124 | 356 |
| **ALT_09252017_20_scaffold_16_256|ALT_09252017_20_Phage_39_18|ALT_09252017_20** | ALT_082018_0_1um_scaffold_4834_12|ALT_082018_0_1um_UNK|ALT_082018_0_1um | 100.000 | 170 | 0 | 0 | 1 | 170 | 1 | 170 | 4.6E-124 | 356 |
| **ALT_09252017_20_scaffold_16_256|ALT_09252017_20_Phage_39_18|ALT_09252017_20** | ALT_09252017_20_scaffold_16_prodigal-single_258|ALT_PHAGE_39_18|ALT_09252017_20 | 100.000 | 170 | 0 | 0 | 1 | 170 | 1 | 170 | 4.6E-124 | 356 |
| **ALT_09252017_20_scaffold_16_256|ALT_09252017_20_Phage_39_18|ALT_09252017_20** | ALT_09252017_20_scaffold_16_256|ALT_09252017_20_Phage_39_18|ALT_09252017_20 | 100.000 | 170 | 0 | 0 | 1 | 170 | 1 | 170 | 4.6E-124 | 356 |
| **ALT_09252017_20_scaffold_16_256|ALT_09252017_20_Phage_39_18|ALT_09252017_20** | AB_072018_0_1um_scaffold_4667_5|AB_072018_0_1um_UNK|AB_072018_0_1um | 99.412 | 170 | 1 | 0 | 1 | 170 | 1 | 170 | 3.96E-123 | 353 |
| **ALT_09252017_20_scaffold_16_256|ALT_09252017_20_Phage_39_18|ALT_09252017_20** | ALT_04162018_0_2um_scaffold_60435_2|ALT_04162018_0_2um_UNK|ALT_04162018_0_2um | 100.000 | 152 | 0 | 0 | 19 | 170 | 1 | 152 | 9.55E-110 | 319 |
| **ALT_09252017_20_scaffold_16_256|ALT_09252017_20_Phage_39_18|ALT_09252017_20** | RHP_09252018_0_1um_scaffold_54_47|RHP_09252018_0_1um_UNK|RHP_09252018_0_1um | 87.647 | 170 | 21 | 0 | 1 | 170 | 1 | 170 | 8.57E-109 | 317 |
| **ALT_09252017_20_scaffold_16_256|ALT_09252017_20_Phage_39_18|ALT_09252017_20** | ALT_03122018_0_1um_scaffold_14613_4|ALT_03122018_0_1um_UNK|ALT_03122018_0_1um | 82.249 | 169 | 30 | 0 | 2 | 170 | 3 | 171 | 7.67E-102 | 300 |
| **ALT_09252017_20_scaffold_16_256|ALT_09252017_20_Phage_39_18|ALT_09252017_20** | RHP_09252018_0_1um_scaffold_7414_6|RHP_09252018_0_1um_UNK|RHP_09252018_0_1um | 70.588 | 170 | 50 | 0 | 1 | 170 | 2 | 171 | 6.3E-85 | 257 |
| **ALT_09252017_20_scaffold_16_256|ALT_09252017_20_Phage_39_18|ALT_09252017_20** | ALT_082018_0_1um_scaffold_141163_2|ALT_082018_0_1um_UNK|ALT_082018_0_1um | 83.099 | 142 | 24 | 0 | 29 | 170 | 1 | 142 | 2.64E-83 | 252 |
| **ALT_09252017_20_scaffold_16_256|ALT_09252017_20_Phage_39_18|ALT_09252017_20** | LacPavin_0818_WC50_scaffold_759589_2|LacPavin_0818_WC50_UNK|LacPavin_0818_WC50 | 69.277 | 166 | 50 | 1 | 1 | 166 | 1 | 165 | 5.53E-80 | 244 |
| **ALT_09252017_20_scaffold_16_256|ALT_09252017_20_Phage_39_18|ALT_09252017_20** | RHP_09252018_0_1um_scaffold_1251_52|RHP_09252018_0_1um_UNK|RHP_09252018_0_1um | 65.217 | 161 | 56 | 0 | 3 | 163 | 2 | 162 | 8.2E-75 | 231 |
| **ALT_09252017_20_scaffold_16_256|ALT_09252017_20_Phage_39_18|ALT_09252017_20** | FFC_07242016_10_scaffold_945_5|FFC_07242016_10_UNK|FFC_07242016_10 | 62.874 | 167 | 60 | 1 | 1 | 167 | 1 | 165 | 1.49E-71 | 223 |
| **ALT_09252017_20_scaffold_16_256|ALT_09252017_20_Phage_39_18|ALT_09252017_20** | ALT_072018_0_1um_scaffold_58_96|ALT_072018_0_1um_UNK|ALT_072018_0_1um | 62.577 | 163 | 61 | 0 | 3 | 165 | 2 | 164 | 3.82E-71 | 222 |
| **ALT_09252017_20_scaffold_16_256|ALT_09252017_20_Phage_39_18|ALT_09252017_20** | ALT_03122018_0_1um_scaffold_1_164|ALT_03122018_0_1um_potentially_complete_phage_41_17|ALT_03122018_0_1um | 62.577 | 163 | 61 | 0 | 3 | 165 | 2 | 164 | 3.82E-71 | 222 |
| **ALT_09252017_20_scaffold_16_256|ALT_09252017_20_Phage_39_18|ALT_09252017_20** | AB_072018_0_1um_scaffold_33_38|AB_072018_0_1um_UNK|AB_072018_0_1um | 62.577 | 163 | 61 | 0 | 3 | 165 | 2 | 164 | 3.82E-71 | 222 |
| **ALT_09252017_20_scaffold_16_256|ALT_09252017_20_Phage_39_18|ALT_09252017_20** | ALT_082018_0_1um_scaffold_29_6|ALT_082018_0_1um_Huge_Phage_41_11|ALT_082018_0_1um | 62.577 | 163 | 61 | 0 | 3 | 165 | 2 | 164 | 3.82E-71 | 222 |
| **ALT_09252017_20_scaffold_16_256|ALT_09252017_20_Phage_39_18|ALT_09252017_20** | ALT_03122018_0_1um_scaffold_7081_5|ALT_03122018_0_1um_UNK|ALT_03122018_0_1um | 61.350 | 163 | 63 | 0 | 1 | 163 | 1 | 163 | 4.31E-71 | 222 |
| **ALT_09252017_20_scaffold_16_256|ALT_09252017_20_Phage_39_18|ALT_09252017_20** | BC_09192017_0_5m_scaffold_41_69|BC_09192017_0_5m_UNK|BC_09192017_0_5m | 62.733 | 161 | 60 | 0 | 1 | 161 | 1 | 161 | 3.15E-69 | 218 |
| **ALT_09252017_20_scaffold_16_256|ALT_09252017_20_Phage_39_18|ALT_09252017_20** | FFC_092018_0_1um_scaffold_7730_2|FFC_092018_0_1um_UNK|FFC_092018_0_1um | 61.585 | 164 | 63 | 0 | 1 | 164 | 1 | 164 | 1.09E-68 | 216 |
| **ALT_09252017_20_scaffold_16_256|ALT_09252017_20_Phage_39_18|ALT_09252017_20** | BC_09192017_0_5m_scaffold_24005_3|BC_09192017_0_5m_UNK|BC_09192017_0_5m | 60.870 | 161 | 63 | 0 | 4 | 164 | 3 | 163 | 2.61E-68 | 215 |
| **ALT_09252017_20_scaffold_16_256|ALT_09252017_20_Phage_39_18|ALT_09252017_20** | AB_092018_0_1um_scaffold_57011_2|AB_092018_0_1um_UNK|AB_092018_0_1um | 61.963 | 163 | 60 | 1 | 4 | 166 | 3 | 163 | 5.73E-68 | 214 |
| **ALT_09252017_20_scaffold_16_256|ALT_09252017_20_Phage_39_18|ALT_09252017_20** | AB_072018_0_1um_scaffold_817_9|AB_072018_0_1um_UNK|AB_072018_0_1um | 60.366 | 164 | 64 | 1 | 4 | 167 | 3 | 165 | 6.19E-68 | 214 |
| **ALT_09252017_20_scaffold_16_256|ALT_09252017_20_Phage_39_18|ALT_09252017_20** | BML_coassembly_scaffold_88333_3|BML_coassembly_UNK|BML_coassembly | 58.537 | 164 | 68 | 0 | 1 | 164 | 1 | 164 | 5.18E-67 | 212 |
| **ALT_09252017_20_scaffold_16_256|ALT_09252017_20_Phage_39_18|ALT_09252017_20** | BC_09192017_0_5m_scaffold_62112_2|BC_09192017_0_5m_UNK|BC_09192017_0_5m | 60.119 | 168 | 64 | 2 | 1 | 167 | 1 | 166 | 1.46E-65 | 208 |
| **ALT_09252017_20_scaffold_16_256|ALT_09252017_20_Phage_39_18|ALT_09252017_20** | AB_092018_0_1um_scaffold_10923_9|AB_092018_0_1um_UNK|AB_092018_0_1um | 56.688 | 157 | 68 | 0 | 1 | 157 | 1 | 157 | 2.47E-62 | 199 |
| **ALT_09252017_20_scaffold_16_256|ALT_09252017_20_Phage_39_18|ALT_09252017_20** | FFC_07242016_10_scaffold_1071_5|FFC_07242016_10_UNK|FFC_07242016_10 | 56.051 | 157 | 69 | 0 | 1 | 157 | 1 | 157 | 2.34E-61 | 197 |
| **ALT_09252017_20_scaffold_16_256|ALT_09252017_20_Phage_39_18|ALT_09252017_20** | BC_09192017_0_5m_scaffold_61331_1|BC_09192017_0_5m_UNK|BC_09192017_0_5m | 66.187 | 139 | 47 | 0 | 29 | 167 | 1 | 139 | 4.97E-61 | 196 |
| **ALT_09252017_20_scaffold_16_256|ALT_09252017_20_Phage_39_18|ALT_09252017_20** | SR-VP_2-4_scaffold_141_4999782_2|SR-VP_2-4cm_UNK|SR-VP_2-4cm | 56.024 | 166 | 71 | 1 | 3 | 168 | 2 | 165 | 2.87E-60 | 194 |
| **ALT_09252017_20_scaffold_16_256|ALT_09252017_20_Phage_39_18|ALT_09252017_20** | FFC_092018_0_1um_scaffold_16039_1|FFC_092018_0_1um_UNK|FFC_092018_0_1um | 65.185 | 135 | 47 | 0 | 1 | 135 | 1 | 135 | 5.92E-60 | 192 |
| **ALT_09252017_20_scaffold_16_256|ALT_09252017_20_Phage_39_18|ALT_09252017_20** | BML_coassembly_scaffold_304109_3|BML_coassembly_UNK|BML_coassembly | 55.195 | 154 | 69 | 0 | 13 | 166 | 1 | 154 | 5.35E-59 | 191 |
| **ALT_09252017_20_scaffold_16_256|ALT_09252017_20_Phage_39_18|ALT_09252017_20** | AB_092018_0_1um_scaffold_95407_2|AB_092018_0_1um_UNK|AB_092018_0_1um | 57.143 | 147 | 63 | 0 | 11 | 157 | 217 | 363 | 5.71E-54 | 185 |
| **ALT_09252017_20_scaffold_16_256|ALT_09252017_20_Phage_39_18|ALT_09252017_20** | Salt_Pond_SF2_A_H2O_MG_scaffold_13457_2|JGI_Salt_Pond_SF2_A_H2O_MG_UNK|JGI_Salt_Pond_SF2_A_H2O_MG | 51.205 | 166 | 81 | 0 | 1 | 166 | 1 | 166 | 2E-53 | 177 |
| **ALT_09252017_20_scaffold_16_256|ALT_09252017_20_Phage_39_18|ALT_09252017_20** | Salt_Pond_SF2_B_H2O_MG_scaffold_729_2|JGI_Salt_Pond_SF2_B_H2O_MG_UNK|JGI_Salt_Pond_SF2_B_H2O_MG | 50.602 | 166 | 82 | 0 | 1 | 166 | 1 | 166 | 7.57E-53 | 176 |
| **ALT_09252017_20_scaffold_16_256|ALT_09252017_20_Phage_39_18|ALT_09252017_20** | Salt_Pond_R2A_C_H2O_MG_scaffold_24845_2|JGI_Salt_Pond_R2A_C_H2O_MG_UNK|JGI_Salt_Pond_R2A_C_H2O_MG | 50.602 | 166 | 82 | 0 | 1 | 166 | 1 | 166 | 7.57E-53 | 176 |
| **ALT_09252017_20_scaffold_16_256|ALT_09252017_20_Phage_39_18|ALT_09252017_20** | Salt_Pond_SF2_C_H2O_MG_scaffold_5179_5|JGI_Salt_Pond_SF2_C_H2O_MG_UNK|JGI_Salt_Pond_SF2_C_H2O_MG | 50.602 | 166 | 82 | 0 | 1 | 166 | 1 | 166 | 7.57E-53 | 176 |
| **ALT_09252017_20_scaffold_16_256|ALT_09252017_20_Phage_39_18|ALT_09252017_20** | Salt_Pond_SF2_C_H2O_MG_scaffold_50247_2|JGI_Salt_Pond_SF2_C_H2O_MG_UNK|JGI_Salt_Pond_SF2_C_H2O_MG | 49.398 | 166 | 84 | 0 | 1 | 166 | 1 | 166 | 7.91E-53 | 176 |
| **ALT_09252017_20_scaffold_16_256|ALT_09252017_20_Phage_39_18|ALT_09252017_20** | Salt_Pond_SF2_B_H2O_MG_scaffold_60217_2|JGI_Salt_Pond_SF2_B_H2O_MG_UNK|JGI_Salt_Pond_SF2_B_H2O_MG | 50.617 | 162 | 78 | 2 | 3 | 163 | 2 | 162 | 2.45E-52 | 175 |
| **ALT_09252017_20_scaffold_16_256|ALT_09252017_20_Phage_39_18|ALT_09252017_20** | Salt_Pond_SF2_B_H2O_MG_scaffold_15417_2|JGI_Salt_Pond_SF2_B_H2O_MG_UNK|JGI_Salt_Pond_SF2_B_H2O_MG | 45.783 | 166 | 90 | 0 | 1 | 166 | 1 | 166 | 2.66E-51 | 172 |
| **ALT_09252017_20_scaffold_16_256|ALT_09252017_20_Phage_39_18|ALT_09252017_20** | Salt_Pond_SF2_C_H2O_MG_scaffold_6646_2|JGI_Salt_Pond_SF2_C_H2O_MG_UNK|JGI_Salt_Pond_SF2_C_H2O_MG | 45.783 | 166 | 90 | 0 | 1 | 166 | 1 | 166 | 2.66E-51 | 172 |
| **ALT_09252017_20_scaffold_16_256|ALT_09252017_20_Phage_39_18|ALT_09252017_20** | Salt_Pond_SF2_B_H2O_MG_scaffold_7972_2|JGI_Salt_Pond_SF2_B_H2O_MG_UNK|JGI_Salt_Pond_SF2_B_H2O_MG | 49.057 | 159 | 81 | 0 | 1 | 159 | 1 | 159 | 1.2E-49 | 168 |
| **ALT_09252017_20_scaffold_16_256|ALT_09252017_20_Phage_39_18|ALT_09252017_20** | Salt_Pond_SF2_C_H2O_MG_scaffold_48011_2|JGI_Salt_Pond_SF2_C_H2O_MG_UNK|JGI_Salt_Pond_SF2_C_H2O_MG | 49.057 | 159 | 81 | 0 | 1 | 159 | 1 | 159 | 1.2E-49 | 168 |
| **ALT_09252017_20_scaffold_16_256|ALT_09252017_20_Phage_39_18|ALT_09252017_20** | Salt_Pond_SF2_B_H2O_MG_scaffold_24002_5|JGI_Salt_Pond_SF2_B_H2O_MG_UNK|JGI_Salt_Pond_SF2_B_H2O_MG | 48.765 | 162 | 81 | 1 | 4 | 165 | 3 | 162 | 7.21E-47 | 160 |
| **ALT_09252017_20_scaffold_16_256|ALT_09252017_20_Phage_39_18|ALT_09252017_20** | Salt_Pond_SF2_A_H2O_MG_scaffold_7906_10|JGI_Salt_Pond_SF2_A_H2O_MG_UNK|JGI_Salt_Pond_SF2_A_H2O_MG | 48.684 | 152 | 78 | 0 | 1 | 152 | 1 | 152 | 2.17E-46 | 159 |
| **ALT_09252017_20_scaffold_16_256|ALT_09252017_20_Phage_39_18|ALT_09252017_20** | AB_092018_0_1um_scaffold_3365_9|AB_092018_0_1um_UNK|AB_092018_0_1um | 44.444 | 171 | 89 | 2 | 1 | 169 | 1 | 167 | 3.83E-46 | 159 |
| **ALT_09252017_20_scaffold_16_256|ALT_09252017_20_Phage_39_18|ALT_09252017_20** | Salt_Pond_R2A_B_H2O_MG_scaffold_26941_4|JGI_Salt_Pond_R2A_B_H2O_MG_UNK|JGI_Salt_Pond_R2A_B_H2O_MG | 50.676 | 148 | 73 | 0 | 1 | 148 | 1 | 148 | 4.74E-46 | 158 |
| **ALT_09252017_20_scaffold_16_256|ALT_09252017_20_Phage_39_18|ALT_09252017_20** | Salt_Pond_R2A_B_H2O_MG_scaffold_48791_1|JGI_Salt_Pond_R2A_B_H2O_MG_UNK|JGI_Salt_Pond_R2A_B_H2O_MG | 48.718 | 156 | 80 | 0 | 1 | 156 | 1 | 156 | 9.8E-46 | 158 |
| **ALT_09252017_20_scaffold_16_256|ALT_09252017_20_Phage_39_18|ALT_09252017_20** | BML_coassembly_scaffold_11403_10|BML_coassembly_UNK|BML_coassembly | 44.444 | 171 | 89 | 2 | 1 | 169 | 1 | 167 | 2.36E-45 | 157 |
| **ALT_09252017_20_scaffold_16_256|ALT_09252017_20_Phage_39_18|ALT_09252017_20** | Salt_Pond_SF2_B_H2O_MG_scaffold_74011_2|JGI_Salt_Pond_SF2_B_H2O_MG_UNK|JGI_Salt_Pond_SF2_B_H2O_MG | 46.061 | 165 | 87 | 1 | 2 | 166 | 1 | 163 | 2.69E-45 | 157 |
| **ALT_09252017_20_scaffold_16_256|ALT_09252017_20_Phage_39_18|ALT_09252017_20** | water-treatment_AWTP-2_BAC_bulk_4_scaffold_4188_16|AWTP-2_BAC_bulk_4_UNK|AWTP-2_BAC_bulk_4 | 47.826 | 161 | 84 | 0 | 4 | 164 | 2 | 162 | 7.46E-45 | 156 |
| **ALT_09252017_20_scaffold_16_256|ALT_09252017_20_Phage_39_18|ALT_09252017_20** | Salt_Pond_SF2_B_H2O_MG_scaffold_1866_10|JGI_Salt_Pond_SF2_B_H2O_MG_UNK|JGI_Salt_Pond_SF2_B_H2O_MG | 45.399 | 163 | 87 | 1 | 4 | 166 | 3 | 163 | 2.36E-44 | 154 |
| **ALT_09252017_20_scaffold_16_256|ALT_09252017_20_Phage_39_18|ALT_09252017_20** | Salt_Pond_SF2_A_H2O_MG_scaffold_36385_2|JGI_Salt_Pond_SF2_A_H2O_MG_UNK|JGI_Salt_Pond_SF2_A_H2O_MG | 45.399 | 163 | 87 | 1 | 4 | 166 | 3 | 163 | 2.36E-44 | 154 |
| **ALT_09252017_20_scaffold_16_256|ALT_09252017_20_Phage_39_18|ALT_09252017_20** | Salt_Pond_R2A_B_H2O_MG_scaffold_11565_3|JGI_Salt_Pond_R2A_B_H2O_MG_UNK|JGI_Salt_Pond_R2A_B_H2O_MG | 45.399 | 163 | 87 | 1 | 4 | 166 | 3 | 163 | 2.36E-44 | 154 |
| **ALT_09252017_20_scaffold_16_256|ALT_09252017_20_Phage_39_18|ALT_09252017_20** | Salt_Pond_SF2_C_H2O_MG_scaffold_1138_14|JGI_Salt_Pond_SF2_C_H2O_MG_UNK|JGI_Salt_Pond_SF2_C_H2O_MG | 45.399 | 163 | 87 | 1 | 4 | 166 | 3 | 163 | 2.36E-44 | 154 |
| **ALT_09252017_20_scaffold_16_256|ALT_09252017_20_Phage_39_18|ALT_09252017_20** | AB_092018_0_1um_scaffold_38162_2|AB_092018_0_1um_UNK|AB_092018_0_1um | 43.478 | 161 | 89 | 1 | 1 | 159 | 1 | 161 | 2.39E-44 | 155 |
| **ALT_09252017_20_scaffold_16_256|ALT_09252017_20_Phage_39_18|ALT_09252017_20** | AB_072018_0_1um_scaffold_15237_2|AB_072018_0_1um_UNK|AB_072018_0_1um | 42.442 | 172 | 91 | 3 | 1 | 169 | 1 | 167 | 1.85E-43 | 152 |
| **ALT_09252017_20_scaffold_16_256|ALT_09252017_20_Phage_39_18|ALT_09252017_20** | Salt_Pond_R2_restored_H2O_MG_scaffold_10_162|JGI_Salt_Pond_R2_restored_H2O_MG_UNK|JGI_Salt_Pond_R2_restored_H2O_MG | 47.531 | 162 | 83 | 1 | 4 | 165 | 3 | 162 | 2.71E-43 | 152 |
| **ALT_09252017_20_scaffold_16_256|ALT_09252017_20_Phage_39_18|ALT_09252017_20** | Salt_Pond_SF2_B_H2O_MG_scaffold_52379_2|JGI_Salt_Pond_SF2_B_H2O_MG_UNK|JGI_Salt_Pond_SF2_B_H2O_MG | 44.242 | 165 | 90 | 1 | 2 | 166 | 1 | 163 | 4.1E-43 | 151 |
| **ALT_09252017_20_scaffold_16_256|ALT_09252017_20_Phage_39_18|ALT_09252017_20** | Salt_Pond_SF2_B_H2O_MG_scaffold_44909_2|JGI_Salt_Pond_SF2_B_H2O_MG_UNK|JGI_Salt_Pond_SF2_B_H2O_MG | 44.242 | 165 | 90 | 1 | 3 | 167 | 2 | 164 | 4.48E-43 | 151 |
| **ALT_09252017_20_scaffold_16_256|ALT_09252017_20_Phage_39_18|ALT_09252017_20** | Salt_Pond_SF2_C_H2O_MG_scaffold_113547_2|JGI_Salt_Pond_SF2_C_H2O_MG_UNK|JGI_Salt_Pond_SF2_C_H2O_MG | 44.242 | 165 | 90 | 1 | 3 | 167 | 2 | 164 | 4.48E-43 | 151 |
| **ALT_09252017_20_scaffold_16_256|ALT_09252017_20_Phage_39_18|ALT_09252017_20** | BC_09192017_0_5m_scaffold_127562_2|BC_09192017_0_5m_UNK|BC_09192017_0_5m | 56.911 | 123 | 51 | 1 | 45 | 167 | 1 | 121 | 4.87E-43 | 149 |
| **ALT_09252017_20_scaffold_16_256|ALT_09252017_20_Phage_39_18|ALT_09252017_20** | BC_09192017_0_5m_scaffold_101410_1|BC_09192017_0_5m_UNK|BC_09192017_0_5m | 42.675 | 157 | 88 | 1 | 1 | 155 | 1 | 157 | 8.32E-43 | 150 |
| **ALT_09252017_20_scaffold_16_256|ALT_09252017_20_Phage_39_18|ALT_09252017_20** | Salt_Pond_R2_restored_H2O_MG_scaffold_4358_12|JGI_Salt_Pond_R2_restored_H2O_MG_UNK|JGI_Salt_Pond_R2_restored_H2O_MG | 46.914 | 162 | 84 | 1 | 4 | 165 | 3 | 162 | 9.4E-43 | 150 |
| **ALT_09252017_20_scaffold_16_256|ALT_09252017_20_Phage_39_18|ALT_09252017_20** | Salt_Pond_R2_restored_H2O_MG_scaffold_6115_3|JGI_Salt_Pond_R2_restored_H2O_MG_UNK|JGI_Salt_Pond_R2_restored_H2O_MG | 44.785 | 163 | 88 | 1 | 4 | 166 | 3 | 163 | 1.38E-42 | 150 |
| **ALT_09252017_20_scaffold_16_256|ALT_09252017_20_Phage_39_18|ALT_09252017_20** | Salt_Pond_R2A_C_H2O_MG_scaffold_32817_4|JGI_Salt_Pond_R2A_C_H2O_MG_UNK|JGI_Salt_Pond_R2A_C_H2O_MG | 44.512 | 164 | 89 | 1 | 2 | 165 | 1 | 162 | 1.51E-42 | 150 |
| **ALT_09252017_20_scaffold_16_256|ALT_09252017_20_Phage_39_18|ALT_09252017_20** | Salt_Pond_R2A_B_H2O_MG_scaffold_31828_4|JGI_Salt_Pond_R2A_B_H2O_MG_UNK|JGI_Salt_Pond_R2A_B_H2O_MG | 44.512 | 164 | 89 | 1 | 2 | 165 | 1 | 162 | 1.51E-42 | 150 |
| **ALT_09252017_20_scaffold_16_256|ALT_09252017_20_Phage_39_18|ALT_09252017_20** | Salt_Pond_R2A_C_H2O_MG_scaffold_420_13|JGI_Salt_Pond_R2A_C_H2O_MG_UNK|JGI_Salt_Pond_R2A_C_H2O_MG | 43.902 | 164 | 90 | 1 | 2 | 165 | 1 | 162 | 4.19E-42 | 149 |
| **ALT_09252017_20_scaffold_16_256|ALT_09252017_20_Phage_39_18|ALT_09252017_20** | Salt_Pond_R2A_B_H2O_MG_scaffold_1098_19|JGI_Salt_Pond_R2A_B_H2O_MG_UNK|JGI_Salt_Pond_R2A_B_H2O_MG | 43.902 | 164 | 90 | 1 | 2 | 165 | 1 | 162 | 4.57E-42 | 148 |
| **ALT_09252017_20_scaffold_16_256|ALT_09252017_20_Phage_39_18|ALT_09252017_20** | Salt_Pond_R2_A_D1_MG_scaffold_36585_5|JGI_Salt_Pond_R2_A_D1_MG_UNK|JGI_Salt_Pond_R2_A_D1_MG | 48.684 | 152 | 76 | 1 | 8 | 159 | 7 | 156 | 5.47E-42 | 148 |
| **ALT_09252017_20_scaffold_16_256|ALT_09252017_20_Phage_39_18|ALT_09252017_20** | Salt_Pond_R2A_C_H2O_MG_scaffold_32816_4|JGI_Salt_Pond_R2A_C_H2O_MG_UNK|JGI_Salt_Pond_R2A_C_H2O_MG | 44.512 | 164 | 89 | 1 | 2 | 165 | 1 | 162 | 6.92E-42 | 148 |
| **ALT_09252017_20_scaffold_16_256|ALT_09252017_20_Phage_39_18|ALT_09252017_20** | Salt_Pond_R2A_B_H2O_MG_scaffold_5557_9|JGI_Salt_Pond_R2A_B_H2O_MG_UNK|JGI_Salt_Pond_R2A_B_H2O_MG | 44.512 | 164 | 89 | 1 | 2 | 165 | 1 | 162 | 6.92E-42 | 148 |
| **ALT_09252017_20_scaffold_16_256|ALT_09252017_20_Phage_39_18|ALT_09252017_20** | Salt_Pond_R2_restored_H2O_MG_scaffold_666_7|JGI_Salt_Pond_R2_restored_H2O_MG_UNK|JGI_Salt_Pond_R2_restored_H2O_MG | 48.684 | 152 | 76 | 1 | 8 | 159 | 7 | 156 | 8.18E-42 | 148 |
| **ALT_09252017_20_scaffold_16_256|ALT_09252017_20_Phage_39_18|ALT_09252017_20** | Salt_Pond_R2_restored_H2O_MG_scaffold_771_29|JGI_Salt_Pond_R2_restored_H2O_MG_UNK|JGI_Salt_Pond_R2_restored_H2O_MG | 44.512 | 164 | 89 | 1 | 2 | 165 | 1 | 162 | 8.31E-42 | 148 |
| **ALT_09252017_20_scaffold_16_256|ALT_09252017_20_Phage_39_18|ALT_09252017_20** | Salt_Pond_SF2_B_H2O_MG_scaffold_31037_2|JGI_Salt_Pond_SF2_B_H2O_MG_UNK|JGI_Salt_Pond_SF2_B_H2O_MG | 41.818 | 165 | 94 | 1 | 4 | 168 | 3 | 165 | 2.16E-41 | 147 |
| **ALT_09252017_20_scaffold_16_256|ALT_09252017_20_Phage_39_18|ALT_09252017_20** | Salt_Pond_R2_restored_H2O_MG_scaffold_9153_6|JGI_Salt_Pond_R2_restored_H2O_MG_UNK|JGI_Salt_Pond_R2_restored_H2O_MG | 43.030 | 165 | 92 | 1 | 2 | 166 | 1 | 163 | 2.73E-41 | 146 |
| **ALT_09252017_20_scaffold_16_256|ALT_09252017_20_Phage_39_18|ALT_09252017_20** | Salt_Pond_R2_restored_H2O_MG_scaffold_5544_7|JGI_Salt_Pond_R2_restored_H2O_MG_UNK|JGI_Salt_Pond_R2_restored_H2O_MG | 43.293 | 164 | 91 | 1 | 2 | 165 | 1 | 162 | 2.95E-41 | 146 |
| **ALT_09252017_20_scaffold_16_256|ALT_09252017_20_Phage_39_18|ALT_09252017_20** | ALT_03122018_0_1um_scaffold_23997_2|ALT_03122018_0_1um_UNK|ALT_03122018_0_1um | 47.651 | 149 | 78 | 0 | 11 | 159 | 11 | 159 | 3.52E-41 | 146 |
| **ALT_09252017_20_scaffold_16_256|ALT_09252017_20_Phage_39_18|ALT_09252017_20** | Salt_Pond_SF2_B_H2O_MG_scaffold_82888_2|JGI_Salt_Pond_SF2_B_H2O_MG_UNK|JGI_Salt_Pond_SF2_B_H2O_MG | 44.516 | 155 | 84 | 1 | 2 | 156 | 1 | 153 | 4.07E-41 | 146 |
| **ALT_09252017_20_scaffold_16_256|ALT_09252017_20_Phage_39_18|ALT_09252017_20** | AB_072018_0_1um_scaffold_1787_4|AB_072018_0_1um_UNK|AB_072018_0_1um | 42.614 | 176 | 85 | 5 | 1 | 169 | 1 | 167 | 7.41E-41 | 145 |
| **ALT_09252017_20_scaffold_16_256|ALT_09252017_20_Phage_39_18|ALT_09252017_20** | SW_scaffold_794_62|LAC_SW_UNK|lac_sw | 44.720 | 161 | 86 | 2 | 9 | 167 | 8 | 167 | 9.23E-41 | 146 |
| **ALT_09252017_20_scaffold_16_256|ALT_09252017_20_Phage_39_18|ALT_09252017_20** | Salt_Pond_SF2_C_H2O_MG_scaffold_28389_2|JGI_Salt_Pond_SF2_C_H2O_MG_UNK|JGI_Salt_Pond_SF2_C_H2O_MG | 43.030 | 165 | 92 | 1 | 2 | 166 | 1 | 163 | 9.75E-41 | 145 |
| **ALT_09252017_20_scaffold_16_256|ALT_09252017_20_Phage_39_18|ALT_09252017_20** | Salt_Pond_SF2_A_H2O_MG_scaffold_23332_3|JGI_Salt_Pond_SF2_A_H2O_MG_UNK|JGI_Salt_Pond_SF2_A_H2O_MG | 44.737 | 152 | 84 | 0 | 7 | 158 | 2 | 153 | 1.32E-40 | 145 |
| **ALT_09252017_20_scaffold_16_256|ALT_09252017_20_Phage_39_18|ALT_09252017_20** | Salt_Pond_SF2_C_H2O_MG_scaffold_829_4|JGI_Salt_Pond_SF2_C_H2O_MG_UNK|JGI_Salt_Pond_SF2_C_H2O_MG | 44.737 | 152 | 84 | 0 | 7 | 158 | 2 | 153 | 1.32E-40 | 145 |
| **ALT_09252017_20_scaffold_16_256|ALT_09252017_20_Phage_39_18|ALT_09252017_20** | Salt_Pond_R2_restored_C_black_MG_scaffold_15105_2|JGI_Salt_Pond_R2_restored_C_black_MG_UNK|JGI_Salt_Pond_R2_restored_C_black_MG | 46.452 | 155 | 78 | 3 | 4 | 156 | 2 | 153 | 3.88E-40 | 144 |
| **ALT_09252017_20_scaffold_16_256|ALT_09252017_20_Phage_39_18|ALT_09252017_20** | Salt_Pond_R2_B_D2_MG_scaffold_55753_2|JGI_Salt_Pond_R2_B_D2_MG_UNK|JGI_Salt_Pond_R2_B_D2_MG | 46.452 | 155 | 78 | 3 | 4 | 156 | 2 | 153 | 3.88E-40 | 144 |
| **ALT_09252017_20_scaffold_16_256|ALT_09252017_20_Phage_39_18|ALT_09252017_20** | Salt_Pond_R2_B_D1_MG_scaffold_59493_2|JGI_Salt_Pond_R2_B_D1_MG_UNK|JGI_Salt_Pond_R2_B_D1_MG | 46.452 | 155 | 78 | 3 | 4 | 156 | 2 | 153 | 3.88E-40 | 144 |
| **ALT_09252017_20_scaffold_16_256|ALT_09252017_20_Phage_39_18|ALT_09252017_20** | Salt_Pond_R1_B_D2_MG_scaffold_7099_16|JGI_Salt_Pond_R1_B_D2_MG_UNK|JGI_Salt_Pond_R1_B_D2_MG | 46.452 | 155 | 78 | 3 | 4 | 156 | 2 | 153 | 3.88E-40 | 144 |
| **ALT_09252017_20_scaffold_16_256|ALT_09252017_20_Phage_39_18|ALT_09252017_20** | Salt_Pond_R1_A_D2_MG_scaffold_1291_16|JGI_Salt_Pond_R1_A_D2_MG_UNK|JGI_Salt_Pond_R1_A_D2_MG | 46.452 | 155 | 78 | 3 | 4 | 156 | 2 | 153 | 3.88E-40 | 144 |
| **ALT_09252017_20_scaffold_16_256|ALT_09252017_20_Phage_39_18|ALT_09252017_20** | Salt_Pond_R1_A_D1_MG_scaffold_3868_22|Salt_Pond_R1_A_D1_MG_UNK|JGI_Salt_Pond_R1_A_D1_MG | 46.452 | 155 | 78 | 3 | 4 | 156 | 2 | 153 | 3.88E-40 | 144 |
| **ALT_09252017_20_scaffold_16_256|ALT_09252017_20_Phage_39_18|ALT_09252017_20** | Salt_Pond_R2_restored_H2O_MG_scaffold_16603_2|JGI_Salt_Pond_R2_restored_H2O_MG_UNK|JGI_Salt_Pond_R2_restored_H2O_MG | 42.945 | 163 | 91 | 1 | 3 | 165 | 2 | 162 | 4.51E-40 | 143 |
| **ALT_09252017_20_scaffold_16_256|ALT_09252017_20_Phage_39_18|ALT_09252017_20** | PH2015_18_scaffold_11383_3|PH2015_18_UNK|PH2015_18 | 42.012 | 169 | 92 | 2 | 1 | 166 | 29 | 194 | 6.43E-40 | 144 |
| **ALT_09252017_20_scaffold_16_256|ALT_09252017_20_Phage_39_18|ALT_09252017_20** | LacPavin_0818_WC50_scaffold_739678_22|LacPavin_0818_WC50_UNK|LacPavin_0818_WC50 | 42.012 | 169 | 93 | 2 | 1 | 167 | 1 | 166 | 8.77E-40 | 143 |
| **ALT_09252017_20_scaffold_16_256|ALT_09252017_20_Phage_39_18|ALT_09252017_20** | LacPavin_0818_WC40_scaffold_777749_19|LacPavin_0818_WC40_UNK|LacPavin_0818_WC40 | 42.012 | 169 | 93 | 2 | 1 | 167 | 1 | 166 | 8.77E-40 | 143 |
| **ALT_09252017_20_scaffold_16_256|ALT_09252017_20_Phage_39_18|ALT_09252017_20** | FFC_04162018_0_1um_scaffold_18828_1|FFC_04162018_0_1um_UNK|FFC_04162018_0_1um | 44.025 | 159 | 86 | 1 | 1 | 159 | 9 | 164 | 8.93E-40 | 143 |
| **ALT_09252017_20_scaffold_16_256|ALT_09252017_20_Phage_39_18|ALT_09252017_20** | Salt_Pond_SF2_B_H2O_MG_scaffold_689_18|JGI_Salt_Pond_SF2_B_H2O_MG_UNK|JGI_Salt_Pond_SF2_B_H2O_MG | 41.718 | 163 | 93 | 1 | 4 | 166 | 3 | 163 | 1E-39 | 142 |
| **ALT_09252017_20_scaffold_16_256|ALT_09252017_20_Phage_39_18|ALT_09252017_20** | Salt_Pond_SF2_A_H2O_MG_scaffold_515_23|JGI_Salt_Pond_SF2_A_H2O_MG_UNK|JGI_Salt_Pond_SF2_A_H2O_MG | 41.718 | 163 | 93 | 1 | 4 | 166 | 3 | 163 | 1E-39 | 142 |
| **ALT_09252017_20_scaffold_16_256|ALT_09252017_20_Phage_39_18|ALT_09252017_20** | Salt_Pond_R2A_C_H2O_MG_scaffold_11591_3|JGI_Salt_Pond_R2A_C_H2O_MG_UNK|JGI_Salt_Pond_R2A_C_H2O_MG | 41.718 | 163 | 93 | 1 | 4 | 166 | 3 | 163 | 1E-39 | 142 |
| **ALT_09252017_20_scaffold_16_256|ALT_09252017_20_Phage_39_18|ALT_09252017_20** | Salt_Pond_R2A_B_H2O_MG_scaffold_2655_2|JGI_Salt_Pond_R2A_B_H2O_MG_UNK|JGI_Salt_Pond_R2A_B_H2O_MG | 41.718 | 163 | 93 | 1 | 4 | 166 | 3 | 163 | 1E-39 | 142 |
| **ALT_09252017_20_scaffold_16_256|ALT_09252017_20_Phage_39_18|ALT_09252017_20** | Salt_Pond_SF2_C_H2O_MG_scaffold_41_92|JGI_Salt_Pond_SF2_C_H2O_MG_UNK|JGI_Salt_Pond_SF2_C_H2O_MG | 41.718 | 163 | 93 | 1 | 4 | 166 | 3 | 163 | 1E-39 | 142 |
| **ALT_09252017_20_scaffold_16_255|ALT_09252017_20_Phage_39_18|ALT_09252017_20** | ALT_04162018_0_2um_scaffold_64309_3|ALT_04162018_0_2um_UNK|ALT_04162018_0_2um | 100.000 | 273 | 0 | 0 | 1 | 273 | 1 | 273 | 0.0 | 562 |
| **ALT_09252017_20_scaffold_16_255|ALT_09252017_20_Phage_39_18|ALT_09252017_20** | ALT_03122018_0_1um_scaffold_3_100|ALT_03122018_0_1um_partial_phage_39_31|ALT_03122018_0_1um | 100.000 | 273 | 0 | 0 | 1 | 273 | 1 | 273 | 0.0 | 562 |
| **ALT_09252017_20_scaffold_16_255|ALT_09252017_20_Phage_39_18|ALT_09252017_20** | ALT_09252017_20_scaffold_16_prodigal-single_257|ALT_PHAGE_39_18|ALT_09252017_20 | 100.000 | 273 | 0 | 0 | 1 | 273 | 1 | 273 | 0.0 | 562 |
| **ALT_09252017_20_scaffold_16_255|ALT_09252017_20_Phage_39_18|ALT_09252017_20** | ALT_09252017_20_scaffold_16_255|ALT_09252017_20_Phage_39_18|ALT_09252017_20 | 100.000 | 273 | 0 | 0 | 1 | 273 | 1 | 273 | 0.0 | 562 |
| **ALT_09252017_20_scaffold_16_255|ALT_09252017_20_Phage_39_18|ALT_09252017_20** | AB_072018_0_1um_scaffold_4667_4|AB_072018_0_1um_UNK|AB_072018_0_1um | 99.634 | 273 | 1 | 0 | 1 | 273 | 1 | 273 | 0.0 | 559 |
| **ALT_09252017_20_scaffold_16_255|ALT_09252017_20_Phage_39_18|ALT_09252017_20** | ALT_072018_0_1um_scaffold_5724_11|ALT_072018_0_1um_UNK|ALT_072018_0_1um | 100.000 | 252 | 0 | 0 | 1 | 252 | 1 | 252 | 0.0 | 521 |
| **ALT_09252017_20_scaffold_16_255|ALT_09252017_20_Phage_39_18|ALT_09252017_20** | RHP_09252018_0_1um_scaffold_54_46|RHP_09252018_0_1um_UNK|RHP_09252018_0_1um | 87.546 | 273 | 33 | 1 | 1 | 273 | 1 | 272 | 9.42E-178 | 500 |
| **ALT_09252017_20_scaffold_16_255|ALT_09252017_20_Phage_39_18|ALT_09252017_20** | ALT_03122018_0_1um_scaffold_14613_3|ALT_03122018_0_1um_UNK|ALT_03122018_0_1um | 75.465 | 269 | 65 | 1 | 1 | 269 | 1 | 268 | 8.26E-148 | 424 |
| **ALT_09252017_20_scaffold_16_255|ALT_09252017_20_Phage_39_18|ALT_09252017_20** | RHP_09252018_0_1um_scaffold_7414_7|RHP_09252018_0_1um_UNK|RHP_09252018_0_1um | 62.082 | 269 | 99 | 1 | 1 | 269 | 1 | 266 | 3.53E-119 | 352 |
| **ALT_09252017_20_scaffold_16_255|ALT_09252017_20_Phage_39_18|ALT_09252017_20** | RHP_09252018_0_1um_scaffold_1251_51|RHP_09252018_0_1um_UNK|RHP_09252018_0_1um | 51.292 | 271 | 124 | 3 | 1 | 269 | 1 | 265 | 1.6E-90 | 279 |
| **ALT_09252017_20_scaffold_16_255|ALT_09252017_20_Phage_39_18|ALT_09252017_20** | ALT_03122018_0_1um_scaffold_7081_4|ALT_03122018_0_1um_UNK|ALT_03122018_0_1um | 51.799 | 278 | 119 | 4 | 1 | 269 | 1 | 272 | 1.36E-86 | 270 |
| **ALT_09252017_20_scaffold_16_255|ALT_09252017_20_Phage_39_18|ALT_09252017_20** | ALT_072018_0_1um_scaffold_58_95|ALT_072018_0_1um_UNK|ALT_072018_0_1um | 51.264 | 277 | 121 | 5 | 1 | 269 | 1 | 271 | 1.78E-86 | 269 |
| **ALT_09252017_20_scaffold_16_255|ALT_09252017_20_Phage_39_18|ALT_09252017_20** | ALT_03122018_0_1um_scaffold_1_165|ALT_03122018_0_1um_potentially_complete_phage_41_17|ALT_03122018_0_1um | 51.264 | 277 | 121 | 5 | 1 | 269 | 1 | 271 | 1.78E-86 | 269 |
| **ALT_09252017_20_scaffold_16_255|ALT_09252017_20_Phage_39_18|ALT_09252017_20** | AB_072018_0_1um_scaffold_33_37|AB_072018_0_1um_UNK|AB_072018_0_1um | 51.264 | 277 | 121 | 5 | 1 | 269 | 1 | 271 | 1.78E-86 | 269 |
| **ALT_09252017_20_scaffold_16_255|ALT_09252017_20_Phage_39_18|ALT_09252017_20** | ALT_082018_0_1um_scaffold_29_5|ALT_082018_0_1um_Huge_Phage_41_11|ALT_082018_0_1um | 51.264 | 277 | 121 | 5 | 1 | 269 | 1 | 271 | 1.78E-86 | 269 |
| **ALT_09252017_20_scaffold_16_255|ALT_09252017_20_Phage_39_18|ALT_09252017_20** | LacPavin_0818_WC50_scaffold_759589_1|LacPavin_0818_WC50_UNK|LacPavin_0818_WC50 | 47.292 | 277 | 137 | 4 | 1 | 271 | 2 | 275 | 7.57E-85 | 265 |
| **ALT_09252017_20_scaffold_16_255|ALT_09252017_20_Phage_39_18|ALT_09252017_20** | ALT_082018_0_1um_scaffold_4834_13|ALT_082018_0_1um_UNK|ALT_082018_0_1um | 100.000 | 126 | 0 | 0 | 148 | 273 | 1 | 126 | 4.64E-83 | 255 |
| **ALT_09252017_20_scaffold_16_255|ALT_09252017_20_Phage_39_18|ALT_09252017_20** | AB_072018_0_1um_scaffold_817_10|AB_072018_0_1um_UNK|AB_072018_0_1um | 45.387 | 271 | 144 | 3 | 1 | 269 | 1 | 269 | 7.05E-83 | 260 |
| **ALT_09252017_20_scaffold_16_255|ALT_09252017_20_Phage_39_18|ALT_09252017_20** | BC_09192017_0_5m_scaffold_41_70|BC_09192017_0_5m_UNK|BC_09192017_0_5m | 48.175 | 274 | 135 | 4 | 2 | 270 | 3 | 274 | 2.69E-82 | 259 |
| **ALT_09252017_20_scaffold_16_255|ALT_09252017_20_Phage_39_18|ALT_09252017_20** | BC_09192017_0_5m_scaffold_28021_5|BC_09192017_0_5m_UNK|BC_09192017_0_5m | 47.101 | 276 | 136 | 5 | 2 | 269 | 3 | 276 | 5.88E-82 | 258 |
| **ALT_09252017_20_scaffold_16_255|ALT_09252017_20_Phage_39_18|ALT_09252017_20** | BC_09192017_0_5m_scaffold_24005_2|BC_09192017_0_5m_UNK|BC_09192017_0_5m | 46.886 | 273 | 142 | 3 | 2 | 271 | 3 | 275 | 1.94E-80 | 254 |
| **ALT_09252017_20_scaffold_16_255|ALT_09252017_20_Phage_39_18|ALT_09252017_20** | LacPavin_0818_WC50_scaffold_160460_2|LacPavin_0818_WC50_UNK|LacPavin_0818_WC50 | 46.715 | 274 | 140 | 3 | 2 | 269 | 3 | 276 | 6.06E-79 | 250 |
| **ALT_09252017_20_scaffold_16_255|ALT_09252017_20_Phage_39_18|ALT_09252017_20** | FFC_092018_0_1um_scaffold_14882_1|FFC_092018_0_1um_UNK|FFC_092018_0_1um | 49.805 | 257 | 125 | 2 | 1 | 253 | 1 | 257 | 1.11E-77 | 246 |
| **ALT_09252017_20_scaffold_16_255|ALT_09252017_20_Phage_39_18|ALT_09252017_20** | BML_coassembly_scaffold_88333_2|BML_coassembly_UNK|BML_coassembly | 46.545 | 275 | 139 | 4 | 2 | 268 | 3 | 277 | 8.77E-71 | 229 |
| **ALT_09252017_20_scaffold_16_255|ALT_09252017_20_Phage_39_18|ALT_09252017_20** | AB_072018_0_1um_scaffold_499_25|AB_072018_0_1um_UNK|AB_072018_0_1um | 42.606 | 284 | 143 | 5 | 1 | 271 | 1 | 277 | 6.84E-70 | 227 |
| **ALT_09252017_20_scaffold_16_255|ALT_09252017_20_Phage_39_18|ALT_09252017_20** | ALT_082018_0_1um_scaffold_4834_14|ALT_082018_0_1um_UNK|ALT_082018_0_1um | 100.000 | 104 | 0 | 0 | 1 | 104 | 1 | 104 | 9.82E-70 | 221 |
| **ALT_09252017_20_scaffold_16_255|ALT_09252017_20_Phage_39_18|ALT_09252017_20** | Salt_Pond_R2_restored_H2O_MG_scaffold_8499_3|JGI_Salt_Pond_R2_restored_H2O_MG_UNK|JGI_Salt_Pond_R2_restored_H2O_MG | 43.416 | 281 | 138 | 6 | 1 | 271 | 1 | 270 | 2.78E-69 | 225 |
| **ALT_09252017_20_scaffold_16_255|ALT_09252017_20_Phage_39_18|ALT_09252017_20** | BML_05172017_1_5m_scaffold_5955_3|BML_05172017_1_5m_UNK|BML_05172017_1_5m | 43.206 | 287 | 137 | 6 | 1 | 271 | 1 | 277 | 5.38E-69 | 225 |
| **ALT_09252017_20_scaffold_16_255|ALT_09252017_20_Phage_39_18|ALT_09252017_20** | RHP_09252018_0_1um_scaffold_2296_13|RHP_09252018_0_1um_UNK|RHP_09252018_0_1um | 45.000 | 280 | 134 | 10 | 1 | 269 | 1 | 271 | 1.29E-68 | 224 |
| **ALT_09252017_20_scaffold_16_255|ALT_09252017_20_Phage_39_18|ALT_09252017_20** | BML_08022017_1_5m_scaffold_73_37|BML_08022017_1_5m_Fragmented_Jumbo_Phage_39_24|BML_08022017_1_5m | 43.206 | 287 | 137 | 6 | 1 | 271 | 1 | 277 | 5.93E-68 | 222 |
| **ALT_09252017_20_scaffold_16_255|ALT_09252017_20_Phage_39_18|ALT_09252017_20** | BML_05172017_7_5m_scaffold_95_31|BML_05172017_7_5m_UNK|BML_05172017_7_5m | 43.206 | 287 | 137 | 6 | 1 | 271 | 1 | 277 | 5.93E-68 | 222 |
| **ALT_09252017_20_scaffold_16_255|ALT_09252017_20_Phage_39_18|ALT_09252017_20** | BML_08042016_9_3m_scaffold_16106_2|BML_08042016_9_3m_UNK|BML_08042016_9_3m | 43.206 | 287 | 137 | 6 | 1 | 271 | 1 | 277 | 5.93E-68 | 222 |
| **ALT_09252017_20_scaffold_16_255|ALT_09252017_20_Phage_39_18|ALT_09252017_20** | BML_08042016_6_5m_scaffold_6_47|BML_08042016_6_5m_Fragmented_Jumbo_Phage_39_11|BML_08042016_6_5m | 43.206 | 287 | 137 | 6 | 1 | 271 | 1 | 277 | 5.93E-68 | 222 |
| **ALT_09252017_20_scaffold_16_255|ALT_09252017_20_Phage_39_18|ALT_09252017_20** | BML_08042016_1_5m_scaffold_705_8|BML_08042016_1_5m_UNK|BML_08042016_1_5m | 43.206 | 287 | 137 | 6 | 1 | 271 | 1 | 277 | 5.93E-68 | 222 |
| **ALT_09252017_20_scaffold_16_255|ALT_09252017_20_Phage_39_18|ALT_09252017_20** | BML_02172017_6_5m_scaffold_2082_6|BML_02172017_6_5m_UNK|BML_02172017_6_5m | 43.206 | 287 | 137 | 6 | 1 | 271 | 1 | 277 | 5.93E-68 | 222 |
| **ALT_09252017_20_scaffold_16_255|ALT_09252017_20_Phage_39_18|ALT_09252017_20** | BML_02172017_0m_scaffold_1012_12|BML_02172017_0m_UNK|BML_02172017_0m | 43.206 | 287 | 137 | 6 | 1 | 271 | 1 | 277 | 5.93E-68 | 222 |
| **ALT_09252017_20_scaffold_16_255|ALT_09252017_20_Phage_39_18|ALT_09252017_20** | BML_08182015_8_5m_scaffold_0_prodigal-single_36|BML_PHAGE_39_34|BML_08182015_8_5m | 43.206 | 287 | 137 | 6 | 1 | 271 | 1 | 277 | 5.93E-68 | 222 |
| **ALT_09252017_20_scaffold_16_255|ALT_09252017_20_Phage_39_18|ALT_09252017_20** | BML_08182015_8_5m_scaffold_0_36|BML_08182015_8_5m_Fragmented_Jumbo_Phage_39_34|BML_08182015_8_5m | 43.206 | 287 | 137 | 6 | 1 | 271 | 1 | 277 | 5.93E-68 | 222 |
| **ALT_09252017_20_scaffold_16_255|ALT_09252017_20_Phage_39_18|ALT_09252017_20** | BML_08182015_6_5m_scaffold_6_prodigal-single_41|BML_PHAGE_39_25|BML_08182015_6_5m | 43.206 | 287 | 137 | 6 | 1 | 271 | 1 | 277 | 5.93E-68 | 222 |
| **ALT_09252017_20_scaffold_16_255|ALT_09252017_20_Phage_39_18|ALT_09252017_20** | BML_08182015_6_5m_scaffold_6_41|BML_08182015_6_5m_Phage_39_25|BML_08182015_6_5m | 43.206 | 287 | 137 | 6 | 1 | 271 | 1 | 277 | 5.93E-68 | 222 |
| **ALT_09252017_20_scaffold_16_255|ALT_09252017_20_Phage_39_18|ALT_09252017_20** | BML_08182015_1_5m_scaffold_5_prodigal-single_212|BML_PHAGE_39_22|BML_08182015_1_5m | 43.206 | 287 | 137 | 6 | 1 | 271 | 1 | 277 | 5.93E-68 | 222 |
| **ALT_09252017_20_scaffold_16_255|ALT_09252017_20_Phage_39_18|ALT_09252017_20** | BML_08182015_1_5m_scaffold_5_209|BML_08182015_1_5m_Incomplete_Jumbo_Phage_39_22|BML_08182015_1_5m | 43.206 | 287 | 137 | 6 | 1 | 271 | 1 | 277 | 5.93E-68 | 222 |
| **ALT_09252017_20_scaffold_16_255|ALT_09252017_20_Phage_39_18|ALT_09252017_20** | BML_coassembly_scaffold_6354_11|BML_coassembly_UNK|BML_coassembly | 43.206 | 287 | 137 | 6 | 1 | 271 | 1 | 277 | 5.93E-68 | 222 |
| **ALT_09252017_20_scaffold_16_255|ALT_09252017_20_Phage_39_18|ALT_09252017_20** | Salt_Pond_R2_restored_H2O_MG_scaffold_10_3|JGI_Salt_Pond_R2_restored_H2O_MG_UNK|JGI_Salt_Pond_R2_restored_H2O_MG | 42.066 | 271 | 141 | 5 | 1 | 263 | 1 | 263 | 1.42E-67 | 221 |
| **ALT_09252017_20_scaffold_16_255|ALT_09252017_20_Phage_39_18|ALT_09252017_20** | Salt_Pond_R1_C_H2O_MG_scaffold_93_69|JGI_Salt_Pond_R1_C_H2O_MG_UNK|JGI_Salt_Pond_R1_C_H2O_MG | 42.086 | 278 | 150 | 4 | 1 | 271 | 1 | 274 | 1.6E-67 | 221 |
| **ALT_09252017_20_scaffold_16_255|ALT_09252017_20_Phage_39_18|ALT_09252017_20** | Salt_Pond_R1_B_H2O_MG_scaffold_482_25|JGI_Salt_Pond_R1_B_H2O_MG_UNK|JGI_Salt_Pond_R1_B_H2O_MG | 42.086 | 278 | 150 | 4 | 1 | 271 | 1 | 274 | 1.6E-67 | 221 |
| **ALT_09252017_20_scaffold_16_255|ALT_09252017_20_Phage_39_18|ALT_09252017_20** | Salt_Pond_R1_A_H2O_MG_scaffold_879_14|JGI_Salt_Pond_R1_A_H2O_MG_UNK|JGI_Salt_Pond_R1_A_H2O_MG | 42.086 | 278 | 150 | 4 | 1 | 271 | 1 | 274 | 1.6E-67 | 221 |
| **ALT_09252017_20_scaffold_16_255|ALT_09252017_20_Phage_39_18|ALT_09252017_20** | FFC_07242016_10_scaffold_945_4|FFC_07242016_10_UNK|FFC_07242016_10 | 44.891 | 274 | 122 | 4 | 1 | 270 | 1 | 249 | 1.74E-67 | 220 |
| **ALT_09252017_20_scaffold_16_255|ALT_09252017_20_Phage_39_18|ALT_09252017_20** | ALT_072018_0_1um_scaffold_94_88|ALT_072018_0_1um_UNK|ALT_072018_0_1um | 41.993 | 281 | 149 | 5 | 1 | 271 | 1 | 277 | 2.49E-67 | 221 |
| **ALT_09252017_20_scaffold_16_255|ALT_09252017_20_Phage_39_18|ALT_09252017_20** | ALT_03122018_0_1um_scaffold_1175_19|ALT_03122018_0_1um_UNK|ALT_03122018_0_1um | 41.993 | 281 | 149 | 5 | 1 | 271 | 1 | 277 | 2.49E-67 | 221 |
| **ALT_09252017_20_scaffold_16_255|ALT_09252017_20_Phage_39_18|ALT_09252017_20** | ALT_082018_0_1um_scaffold_114_142|ALT_082018_0_1um_UNK|ALT_082018_0_1um | 41.993 | 281 | 149 | 5 | 1 | 271 | 1 | 277 | 2.49E-67 | 221 |
| **ALT_09252017_20_scaffold_16_255|ALT_09252017_20_Phage_39_18|ALT_09252017_20** | BML_08022017_1_5m_scaffold_145_35|BML_08022017_1_5m_UNK|BML_08022017_1_5m | 42.160 | 287 | 140 | 6 | 1 | 271 | 1 | 277 | 1.98E-66 | 218 |
| **ALT_09252017_20_scaffold_16_255|ALT_09252017_20_Phage_39_18|ALT_09252017_20** | BML_coassembly_scaffold_7136_12|BML_coassembly_UNK|BML_coassembly | 42.160 | 287 | 140 | 6 | 1 | 271 | 1 | 277 | 1.98E-66 | 218 |
| **ALT_09252017_20_scaffold_16_255|ALT_09252017_20_Phage_39_18|ALT_09252017_20** | ALT_072018_0_1um_scaffold_13618_3|ALT_072018_0_1um_UNK|ALT_072018_0_1um | 43.772 | 281 | 130 | 5 | 1 | 262 | 1 | 272 | 2.63E-66 | 218 |
| **ALT_09252017_20_scaffold_16_255|ALT_09252017_20_Phage_39_18|ALT_09252017_20** | ALT_03122018_0_1um_scaffold_33_98|ALT_03122018_0_1um_UNK|ALT_03122018_0_1um | 43.772 | 281 | 130 | 5 | 1 | 262 | 1 | 272 | 3.2E-66 | 218 |
| **ALT_09252017_20_scaffold_16_255|ALT_09252017_20_Phage_39_18|ALT_09252017_20** | ALT_082018_0_1um_scaffold_34542_2|ALT_082018_0_1um_UNK|ALT_082018_0_1um | 43.772 | 281 | 130 | 5 | 1 | 262 | 1 | 272 | 3.2E-66 | 218 |
| **ALT_09252017_20_scaffold_16_255|ALT_09252017_20_Phage_39_18|ALT_09252017_20** | ALT_082018_0_1um_scaffold_80872_2|ALT_082018_0_1um_UNK|ALT_082018_0_1um | 43.060 | 281 | 146 | 5 | 1 | 271 | 1 | 277 | 6.88E-66 | 217 |
| **ALT_09252017_20_scaffold_16_255|ALT_09252017_20_Phage_39_18|ALT_09252017_20** | AB_072018_0_1um_scaffold_460_28|AB_072018_0_1um_UNK|AB_072018_0_1um | 40.647 | 278 | 157 | 3 | 1 | 273 | 1 | 275 | 7.59E-66 | 217 |
| **ALT_09252017_20_scaffold_16_255|ALT_09252017_20_Phage_39_18|ALT_09252017_20** | FFC_092018_0_1um_scaffold_8647_2|FFC_092018_0_1um_UNK|FFC_092018_0_1um | 43.110 | 283 | 141 | 8 | 1 | 271 | 1 | 275 | 1.03E-65 | 216 |
| **ALT_09252017_20_scaffold_16_255|ALT_09252017_20_Phage_39_18|ALT_09252017_20** | ALT_082018_0_1um_scaffold_25643_7|ALT_082018_0_1um_UNK|ALT_082018_0_1um | 40.357 | 280 | 154 | 3 | 1 | 273 | 1 | 274 | 2.77E-65 | 215 |
| **ALT_09252017_20_scaffold_16_255|ALT_09252017_20_Phage_39_18|ALT_09252017_20** | BC_09192017_0_5m_scaffold_26874_3|BC_09192017_0_5m_UNK|BC_09192017_0_5m | 42.955 | 291 | 136 | 7 | 1 | 271 | 1 | 281 | 3.4E-65 | 215 |
| **ALT_09252017_20_scaffold_16_255|ALT_09252017_20_Phage_39_18|ALT_09252017_20** | AB_072018_0_1um_scaffold_481_7|AB_072018_0_1um_UNK|AB_072018_0_1um | 42.545 | 275 | 149 | 5 | 1 | 269 | 1 | 272 | 3.61E-65 | 215 |
| **ALT_09252017_20_scaffold_16_255|ALT_09252017_20_Phage_39_18|ALT_09252017_20** | ALT_082018_0_1um_scaffold_5715_9|ALT_082018_0_1um_UNK|ALT_082018_0_1um | 42.545 | 275 | 149 | 5 | 1 | 269 | 1 | 272 | 3.61E-65 | 215 |
| **ALT_09252017_20_scaffold_16_255|ALT_09252017_20_Phage_39_18|ALT_09252017_20** | RHP_09252018_0_1um_scaffold_9565_7|RHP_09252018_0_1um_UNK|RHP_09252018_0_1um | 42.647 | 272 | 131 | 6 | 1 | 258 | 1 | 261 | 1.02E-64 | 214 |
| **ALT_09252017_20_scaffold_16_255|ALT_09252017_20_Phage_39_18|ALT_09252017_20** | Salt_Pond_R2_restored_H2O_MG_scaffold_262_12|JGI_Salt_Pond_R2_restored_H2O_MG_UNK|JGI_Salt_Pond_R2_restored_H2O_MG | 42.599 | 277 | 147 | 5 | 1 | 271 | 1 | 271 | 1.29E-64 | 214 |
| **ALT_09252017_20_scaffold_16_255|ALT_09252017_20_Phage_39_18|ALT_09252017_20** | Salt_Pond_R1_C_H2O_MG_scaffold_143_14|JGI_Salt_Pond_R1_C_H2O_MG_UNK|JGI_Salt_Pond_R1_C_H2O_MG | 40.000 | 275 | 157 | 5 | 1 | 269 | 1 | 273 | 2.02E-64 | 213 |
| **ALT_09252017_20_scaffold_16_255|ALT_09252017_20_Phage_39_18|ALT_09252017_20** | Salt_Pond_R1_B_H2O_MG_scaffold_150_41|JGI_Salt_Pond_R1_B_H2O_MG_UNK|JGI_Salt_Pond_R1_B_H2O_MG | 40.000 | 275 | 157 | 5 | 1 | 269 | 1 | 273 | 2.02E-64 | 213 |
| **ALT_09252017_20_scaffold_16_255|ALT_09252017_20_Phage_39_18|ALT_09252017_20** | Salt_Pond_R1_A_H2O_MG_scaffold_88_44|JGI_Salt_Pond_R1_A_H2O_MG_UNK|JGI_Salt_Pond_R1_A_H2O_MG | 40.000 | 275 | 157 | 5 | 1 | 269 | 1 | 273 | 2.02E-64 | 213 |
| **ALT_09252017_20_scaffold_16_255|ALT_09252017_20_Phage_39_18|ALT_09252017_20** | ALT_03122018_0_1um_scaffold_5949_3|ALT_03122018_0_1um_UNK|ALT_03122018_0_1um | 43.015 | 272 | 130 | 6 | 1 | 258 | 1 | 261 | 2.47E-64 | 213 |
| **ALT_09252017_20_scaffold_16_255|ALT_09252017_20_Phage_39_18|ALT_09252017_20** | ALT_082018_0_1um_scaffold_15359_2|ALT_082018_0_1um_UNK|ALT_082018_0_1um | 43.015 | 272 | 130 | 6 | 1 | 258 | 1 | 261 | 2.47E-64 | 213 |
| **ALT_09252017_20_scaffold_16_255|ALT_09252017_20_Phage_39_18|ALT_09252017_20** | BML_08182015_8_5m_scaffold_2742_4|BML_08182015_8_5m_UNK|BML_08182015_8_5m | 42.756 | 283 | 139 | 6 | 1 | 269 | 1 | 274 | 3.44E-64 | 213 |
| **ALT_09252017_20_scaffold_16_255|ALT_09252017_20_Phage_39_18|ALT_09252017_20** | BML_08182015_1_5m_scaffold_3178_6|BML_08182015_1_5m_UNK|BML_08182015_1_5m | 42.756 | 283 | 139 | 6 | 1 | 269 | 1 | 274 | 3.44E-64 | 213 |
| **ALT_09252017_20_scaffold_16_255|ALT_09252017_20_Phage_39_18|ALT_09252017_20** | BML_coassembly_scaffold_373_109|BML_coassembly_UNK|BML_coassembly | 42.756 | 283 | 139 | 6 | 1 | 269 | 1 | 274 | 3.44E-64 | 213 |
| **ALT_09252017_20_scaffold_16_255|ALT_09252017_20_Phage_39_18|ALT_09252017_20** | Salt_Pond_SF2_B_H2O_MG_scaffold_94820_2|JGI_Salt_Pond_SF2_B_H2O_MG_UNK|JGI_Salt_Pond_SF2_B_H2O_MG | 40.426 | 282 | 156 | 5 | 1 | 271 | 1 | 281 | 5.12E-64 | 212 |
| **ALT_09252017_20_scaffold_16_255|ALT_09252017_20_Phage_39_18|ALT_09252017_20** | BML_08022017_1_5m_scaffold_1076_14|BML_08022017_1_5m_UNK|BML_08022017_1_5m | 42.606 | 284 | 140 | 6 | 1 | 271 | 1 | 274 | 7.26E-64 | 212 |
| **ALT_09252017_20_scaffold_16_255|ALT_09252017_20_Phage_39_18|ALT_09252017_20** | BML_coassembly_scaffold_1849_29|BML_coassembly_UNK|BML_coassembly | 42.606 | 284 | 140 | 6 | 1 | 271 | 1 | 274 | 7.26E-64 | 212 |
| **ALT_09252017_20_scaffold_16_255|ALT_09252017_20_Phage_39_18|ALT_09252017_20** | LacPavin_0818_WC50_scaffold_136821_13|LacPavin_0818_WC50_UNK|LacPavin_0818_WC50 | 41.818 | 275 | 151 | 5 | 1 | 269 | 1 | 272 | 8.47E-64 | 211 |
| **ALT_09252017_20_scaffold_16_255|ALT_09252017_20_Phage_39_18|ALT_09252017_20** | Salt_Pond_R2_restored_H2O_MG_scaffold_241_13|JGI_Salt_Pond_R2_restored_H2O_MG_UNK|JGI_Salt_Pond_R2_restored_H2O_MG | 37.906 | 277 | 160 | 5 | 1 | 271 | 1 | 271 | 2.9E-63 | 210 |
| **ALT_09252017_20_scaffold_16_255|ALT_09252017_20_Phage_39_18|ALT_09252017_20** | LacPavin_0818_WC50_scaffold_931777_23|LacPavin_0818_WC50_UNK|LacPavin_0818_WC50 | 42.254 | 284 | 138 | 7 | 1 | 269 | 1 | 273 | 4.51E-63 | 210 |
| **ALT_09252017_20_scaffold_16_255|ALT_09252017_20_Phage_39_18|ALT_09252017_20** | LacPavin_0818_WC40_scaffold_456509_4|LacPavin_0818_WC40_UNK|LacPavin_0818_WC40 | 42.254 | 284 | 138 | 7 | 1 | 269 | 1 | 273 | 4.51E-63 | 210 |
| **ALT_09252017_20_scaffold_16_255|ALT_09252017_20_Phage_39_18|ALT_09252017_20** | ALT_072018_0_1um_scaffold_29443_2|ALT_072018_0_1um_UNK|ALT_072018_0_1um | 42.279 | 272 | 132 | 6 | 1 | 258 | 1 | 261 | 6.8E-63 | 209 |
| **ALT_09252017_20_scaffold_16_255|ALT_09252017_20_Phage_39_18|ALT_09252017_20** | BC_09192017_0_5m_scaffold_16751_3|BC_09192017_0_5m_UNK|BC_09192017_0_5m | 40.569 | 281 | 152 | 6 | 1 | 272 | 1 | 275 | 7.41E-63 | 209 |
| **ALT_09252017_20_scaffold_16_255|ALT_09252017_20_Phage_39_18|ALT_09252017_20** | ALT_04162018_0_2um_scaffold_986_24|ALT_04162018_0_2um_UNK|ALT_04162018_0_2um | 41.197 | 284 | 141 | 7 | 1 | 269 | 1 | 273 | 9.31E-63 | 209 |
| **ALT_09252017_20_scaffold_16_255|ALT_09252017_20_Phage_39_18|ALT_09252017_20** | ALT_03122018_0_1um_scaffold_2951_7|ALT_03122018_0_1um_UNK|ALT_03122018_0_1um | 41.197 | 284 | 141 | 7 | 1 | 269 | 1 | 273 | 9.31E-63 | 209 |
| **ALT_09252017_20_scaffold_16_255|ALT_09252017_20_Phage_39_18|ALT_09252017_20** | ALT_09252017_20_scaffold_265_236|ALT_09252017_20_Phage_42_25|ALT_09252017_20 | 41.197 | 284 | 141 | 7 | 1 | 269 | 1 | 273 | 9.31E-63 | 209 |
| **ALT_09252017_20_scaffold_16_255|ALT_09252017_20_Phage_39_18|ALT_09252017_20** | Salt_Pond_R2_restored_H2O_MG_scaffold_3659_9|JGI_Salt_Pond_R2_restored_H2O_MG_UNK|JGI_Salt_Pond_R2_restored_H2O_MG | 41.085 | 258 | 148 | 3 | 1 | 258 | 1 | 254 | 1.7E-62 | 208 |
| **ALT_09252017_20_scaffold_16_255|ALT_09252017_20_Phage_39_18|ALT_09252017_20** | BML_02132018_6_5m_scaffold_175_62|BML_02132018_6_5m_UNK|BML_02132018_6_5m | 42.456 | 285 | 136 | 6 | 1 | 269 | 1 | 273 | 1.74E-62 | 208 |
| **ALT_09252017_20_scaffold_16_255|ALT_09252017_20_Phage_39_18|ALT_09252017_20** | BML_08022017_1_5m_scaffold_839_13|BML_08022017_1_5m_UNK|BML_08022017_1_5m | 42.456 | 285 | 136 | 6 | 1 | 269 | 1 | 273 | 1.74E-62 | 208 |
| **ALT_09252017_20_scaffold_16_255|ALT_09252017_20_Phage_39_18|ALT_09252017_20** | BML_coassembly_scaffold_4017_29|BML_coassembly_UNK|BML_coassembly | 42.456 | 285 | 136 | 6 | 1 | 269 | 1 | 273 | 1.74E-62 | 208 |
| **ALT_09252017_20_scaffold_16_255|ALT_09252017_20_Phage_39_18|ALT_09252017_20** | Salt_Pond_R2A_C_H2O_MG_scaffold_3884_10|JGI_Salt_Pond_R2A_C_H2O_MG_UNK|JGI_Salt_Pond_R2A_C_H2O_MG | 38.662 | 269 | 145 | 3 | 1 | 269 | 1 | 249 | 1.81E-62 | 207 |
| **ALT_09252017_20_scaffold_16_255|ALT_09252017_20_Phage_39_18|ALT_09252017_20** | Salt_Pond_R2A_B_H2O_MG_scaffold_1824_12|JGI_Salt_Pond_R2A_B_H2O_MG_UNK|JGI_Salt_Pond_R2A_B_H2O_MG | 38.662 | 269 | 145 | 3 | 1 | 269 | 1 | 249 | 1.81E-62 | 207 |
| **ALT_09252017_20_scaffold_16_255|ALT_09252017_20_Phage_39_18|ALT_09252017_20** | ALT_03122018_0_1um_scaffold_3035_7|ALT_03122018_0_1um_UNK|ALT_03122018_0_1um | 43.321 | 277 | 141 | 8 | 1 | 268 | 1 | 270 | 3.1E-62 | 207 |
| **ALT_09252017_20_scaffold_16_255|ALT_09252017_20_Phage_39_18|ALT_09252017_20** | Salt_Pond_R1_C_H2O_MG_scaffold_13906_2|JGI_Salt_Pond_R1_C_H2O_MG_UNK|JGI_Salt_Pond_R1_C_H2O_MG | 43.023 | 258 | 136 | 4 | 1 | 253 | 1 | 252 | 7.59E-62 | 206 |
| **ALT_09252017_20_scaffold_16_255|ALT_09252017_20_Phage_39_18|ALT_09252017_20** | Salt_Pond_R1_C_H2O_MG_scaffold_2789_8|JGI_Salt_Pond_R1_C_H2O_MG_UNK|JGI_Salt_Pond_R1_C_H2O_MG | 40.876 | 274 | 150 | 6 | 1 | 272 | 1 | 264 | 1.11E-61 | 206 |
| **ALT_09252017_20_scaffold_16_255|ALT_09252017_20_Phage_39_18|ALT_09252017_20** | Salt_Pond_R1_B_H2O_MG_scaffold_873_23|JGI_Salt_Pond_R1_B_H2O_MG_UNK|JGI_Salt_Pond_R1_B_H2O_MG | 40.876 | 274 | 150 | 6 | 1 | 272 | 1 | 264 | 1.11E-61 | 206 |
| **ALT_09252017_20_scaffold_16_255|ALT_09252017_20_Phage_39_18|ALT_09252017_20** | Salt_Pond_R1_B_H2O_MG_scaffold_9977_3|JGI_Salt_Pond_R1_B_H2O_MG_UNK|JGI_Salt_Pond_R1_B_H2O_MG | 39.273 | 275 | 161 | 4 | 1 | 271 | 1 | 273 | 2.05E-61 | 205 |
| **ALT_09252017_20_scaffold_16_255|ALT_09252017_20_Phage_39_18|ALT_09252017_20** | ALT_03122018_0_1um_scaffold_16424_4|ALT_03122018_0_1um_UNK|ALT_03122018_0_1um | 44.541 | 229 | 123 | 2 | 1 | 228 | 1 | 226 | 4.38E-61 | 203 |
| **ALT_09252017_20_scaffold_16_255|ALT_09252017_20_Phage_39_18|ALT_09252017_20** | BML_08012017_8_0m_scaffold_12472_1|BML_08012017_8_0m_UNK|BML_08012017_8_0m | 40.000 | 280 | 151 | 5 | 1 | 269 | 1 | 274 | 4.42E-61 | 205 |
| **ALT_09252017_20_scaffold_16_255|ALT_09252017_20_Phage_39_18|ALT_09252017_20** | BML_08022017_6_5m_scaffold_11_70|BML_08022017_6_5m_UNK|BML_08022017_6_5m | 40.000 | 280 | 151 | 5 | 1 | 269 | 1 | 274 | 4.42E-61 | 205 |
| **ALT_09252017_20_scaffold_16_255|ALT_09252017_20_Phage_39_18|ALT_09252017_20** | BML_08022017_1_5m_scaffold_40_233|BML_08022017_1_5m_UNK|BML_08022017_1_5m | 40.000 | 280 | 151 | 5 | 1 | 269 | 1 | 274 | 4.42E-61 | 205 |
| **ALT_09252017_20_scaffold_16_254|ALT_09252017_20_Phage_39_18|ALT_09252017_20** | ALT_072018_0_1um_scaffold_5724_10|ALT_072018_0_1um_UNK|ALT_072018_0_1um | 100.000 | 69 | 0 | 0 | 1 | 69 | 1 | 69 | 2.05E-37 | 129 |
| **ALT_09252017_20_scaffold_16_254|ALT_09252017_20_Phage_39_18|ALT_09252017_20** | ALT_04162018_0_2um_scaffold_64309_2|ALT_04162018_0_2um_UNK|ALT_04162018_0_2um | 100.000 | 69 | 0 | 0 | 1 | 69 | 1 | 69 | 2.05E-37 | 129 |
| **ALT_09252017_20_scaffold_16_254|ALT_09252017_20_Phage_39_18|ALT_09252017_20** | ALT_03122018_0_1um_scaffold_3_99|ALT_03122018_0_1um_partial_phage_39_31|ALT_03122018_0_1um | 100.000 | 69 | 0 | 0 | 1 | 69 | 1 | 69 | 2.05E-37 | 129 |
| **ALT_09252017_20_scaffold_16_254|ALT_09252017_20_Phage_39_18|ALT_09252017_20** | AB_072018_0_1um_scaffold_4667_3|AB_072018_0_1um_UNK|AB_072018_0_1um | 100.000 | 69 | 0 | 0 | 1 | 69 | 1 | 69 | 2.05E-37 | 129 |
| **ALT_09252017_20_scaffold_16_254|ALT_09252017_20_Phage_39_18|ALT_09252017_20** | ALT_082018_0_1um_scaffold_4834_15|ALT_082018_0_1um_UNK|ALT_082018_0_1um | 100.000 | 69 | 0 | 0 | 1 | 69 | 1 | 69 | 2.05E-37 | 129 |
| **ALT_09252017_20_scaffold_16_254|ALT_09252017_20_Phage_39_18|ALT_09252017_20** | ALT_09252017_20_scaffold_16_prodigal-single_256|ALT_PHAGE_39_18|ALT_09252017_20 | 100.000 | 69 | 0 | 0 | 1 | 69 | 1 | 69 | 2.05E-37 | 129 |
| **ALT_09252017_20_scaffold_16_254|ALT_09252017_20_Phage_39_18|ALT_09252017_20** | ALT_09252017_20_scaffold_16_254|ALT_09252017_20_Phage_39_18|ALT_09252017_20 | 100.000 | 69 | 0 | 0 | 1 | 69 | 1 | 69 | 2.05E-37 | 129 |
| **ALT_09252017_20_scaffold_16_254|ALT_09252017_20_Phage_39_18|ALT_09252017_20** | AB_092018_0_1um_scaffold_47720_2|AB_092018_0_1um_UNK|AB_092018_0_1um | 88.060 | 67 | 8 | 0 | 1 | 67 | 1 | 67 | 7.73E-33 | 117 |
| **ALT_09252017_20_scaffold_16_254|ALT_09252017_20_Phage_39_18|ALT_09252017_20** | BC_09192017_0_5m_scaffold_3193_16|BC_09192017_0_5m_UNK|BC_09192017_0_5m | 85.507 | 69 | 10 | 0 | 1 | 69 | 1 | 69 | 2.18E-32 | 116 |
| **ALT_09252017_20_scaffold_16_258|ALT_09252017_20_Phage_39_18|ALT_09252017_20** | ALT_072018_0_1um_scaffold_31513_2|ALT_072018_0_1um_UNK|ALT_072018_0_1um | 100.000 | 228 | 0 | 0 | 1 | 228 | 1 | 228 | 3.83E-169 | 475 |
| **ALT_09252017_20_scaffold_16_258|ALT_09252017_20_Phage_39_18|ALT_09252017_20** | ALT_04162018_0_2um_scaffold_5662_10|ALT_04162018_0_2um_UNK|ALT_04162018_0_2um | 100.000 | 228 | 0 | 0 | 1 | 228 | 1 | 228 | 3.83E-169 | 475 |
| **ALT_09252017_20_scaffold_16_258|ALT_09252017_20_Phage_39_18|ALT_09252017_20** | ALT_03122018_0_1um_scaffold_3_103|ALT_03122018_0_1um_partial_phage_39_31|ALT_03122018_0_1um | 100.000 | 228 | 0 | 0 | 1 | 228 | 1 | 228 | 3.83E-169 | 475 |
| **ALT_09252017_20_scaffold_16_258|ALT_09252017_20_Phage_39_18|ALT_09252017_20** | ALT_082018_0_1um_scaffold_4834_10|ALT_082018_0_1um_UNK|ALT_082018_0_1um | 100.000 | 228 | 0 | 0 | 1 | 228 | 1 | 228 | 3.83E-169 | 475 |
| **ALT_09252017_20_scaffold_16_258|ALT_09252017_20_Phage_39_18|ALT_09252017_20** | ALT_09252017_20_scaffold_16_prodigal-single_260|ALT_PHAGE_39_18|ALT_09252017_20 | 100.000 | 228 | 0 | 0 | 1 | 228 | 1 | 228 | 3.83E-169 | 475 |
| **ALT_09252017_20_scaffold_16_258|ALT_09252017_20_Phage_39_18|ALT_09252017_20** | ALT_09252017_20_scaffold_16_258|ALT_09252017_20_Phage_39_18|ALT_09252017_20 | 100.000 | 228 | 0 | 0 | 1 | 228 | 1 | 228 | 3.83E-169 | 475 |
| **ALT_09252017_20_scaffold_16_258|ALT_09252017_20_Phage_39_18|ALT_09252017_20** | AB_072018_0_1um_scaffold_10951_1|AB_072018_0_1um_UNK|AB_072018_0_1um | 100.000 | 212 | 0 | 0 | 17 | 228 | 1 | 212 | 1.9E-156 | 442 |
| **ALT_09252017_20_scaffold_16_258|ALT_09252017_20_Phage_39_18|ALT_09252017_20** | ALT_03122018_0_1um_scaffold_6730_2|ALT_03122018_0_1um_UNK|ALT_03122018_0_1um | 58.952 | 229 | 93 | 1 | 1 | 228 | 1 | 229 | 7.35E-92 | 279 |
| **ALT_09252017_20_scaffold_16_258|ALT_09252017_20_Phage_39_18|ALT_09252017_20** | RHP_09252018_0_1um_scaffold_54_49|RHP_09252018_0_1um_UNK|RHP_09252018_0_1um | 58.079 | 229 | 95 | 1 | 1 | 228 | 1 | 229 | 2.39E-89 | 273 |
| **ALT_09252017_20_scaffold_16_258|ALT_09252017_20_Phage_39_18|ALT_09252017_20** | RHP_09252018_0_1um_scaffold_7414_4|RHP_09252018_0_1um_UNK|RHP_09252018_0_1um | 35.965 | 228 | 146 | 0 | 1 | 228 | 7 | 234 | 7.18E-41 | 150 |
| **ALT_09252017_20_scaffold_16_258|ALT_09252017_20_Phage_39_18|ALT_09252017_20** | ALT_072018_0_1um_scaffold_58_98|ALT_072018_0_1um_UNK|ALT_072018_0_1um | 34.091 | 220 | 139 | 2 | 6 | 225 | 6 | 219 | 1.88E-36 | 138 |
| **ALT_09252017_20_scaffold_16_258|ALT_09252017_20_Phage_39_18|ALT_09252017_20** | ALT_03122018_0_1um_scaffold_1_162|ALT_03122018_0_1um_potentially_complete_phage_41_17|ALT_03122018_0_1um | 34.091 | 220 | 139 | 2 | 6 | 225 | 6 | 219 | 1.88E-36 | 138 |
| **ALT_09252017_20_scaffold_16_258|ALT_09252017_20_Phage_39_18|ALT_09252017_20** | AB_072018_0_1um_scaffold_33_40|AB_072018_0_1um_UNK|AB_072018_0_1um | 34.091 | 220 | 139 | 2 | 6 | 225 | 6 | 219 | 1.88E-36 | 138 |
| **ALT_09252017_20_scaffold_16_258|ALT_09252017_20_Phage_39_18|ALT_09252017_20** | ALT_082018_0_2um_scaffold_24253_2|ALT_082018_0_2um_UNK|ALT_082018_0_2um | 34.091 | 220 | 139 | 2 | 6 | 225 | 6 | 219 | 1.88E-36 | 138 |
| **ALT_09252017_20_scaffold_16_258|ALT_09252017_20_Phage_39_18|ALT_09252017_20** | ALT_082018_0_1um_scaffold_29_8|ALT_082018_0_1um_Huge_Phage_41_11|ALT_082018_0_1um | 34.091 | 220 | 139 | 2 | 6 | 225 | 6 | 219 | 1.88E-36 | 138 |
| **ALT_09252017_20_scaffold_16_258|ALT_09252017_20_Phage_39_18|ALT_09252017_20** | BC_09192017_0_5m_scaffold_37828_2|BC_09192017_0_5m_UNK|BC_09192017_0_5m | 33.184 | 223 | 143 | 4 | 6 | 226 | 10 | 228 | 1.05E-35 | 136 |
| **ALT_09252017_20_scaffold_16_258|ALT_09252017_20_Phage_39_18|ALT_09252017_20** | AB_072018_0_1um_scaffold_817_7|AB_072018_0_1um_UNK|AB_072018_0_1um | 34.061 | 229 | 144 | 3 | 1 | 226 | 3 | 227 | 3.92E-34 | 132 |
| **ALT_09252017_20_scaffold_16_258|ALT_09252017_20_Phage_39_18|ALT_09252017_20** | BC_09192017_0_5m_scaffold_38632_3|BC_09192017_0_5m_UNK|BC_09192017_0_5m | 31.250 | 224 | 147 | 4 | 1 | 222 | 5 | 223 | 1.6E-31 | 125 |
| **ALT_09252017_20_scaffold_16_258|ALT_09252017_20_Phage_39_18|ALT_09252017_20** | FFC_082018_0_1um_scaffold_15254_2|FFC_082018_0_1um_UNK|FFC_082018_0_1um | 31.604 | 212 | 138 | 3 | 12 | 222 | 1 | 206 | 6E-31 | 124 |
| **ALT_09252017_20_scaffold_16_259|ALT_09252017_20_Phage_39_18|ALT_09252017_20** | ALT_04162018_0_2um_scaffold_5662_9|ALT_04162018_0_2um_UNK|ALT_04162018_0_2um | 100.000 | 148 | 0 | 0 | 1 | 148 | 1 | 148 | 4.59E-100 | 293 |
| **ALT_09252017_20_scaffold_16_259|ALT_09252017_20_Phage_39_18|ALT_09252017_20** | ALT_03122018_0_1um_scaffold_3_104|ALT_03122018_0_1um_partial_phage_39_31|ALT_03122018_0_1um | 100.000 | 148 | 0 | 0 | 1 | 148 | 1 | 148 | 4.59E-100 | 293 |
| **ALT_09252017_20_scaffold_16_259|ALT_09252017_20_Phage_39_18|ALT_09252017_20** | ALT_082018_0_1um_scaffold_4834_9|ALT_082018_0_1um_UNK|ALT_082018_0_1um | 100.000 | 148 | 0 | 0 | 1 | 148 | 1 | 148 | 4.59E-100 | 293 |
| **ALT_09252017_20_scaffold_16_259|ALT_09252017_20_Phage_39_18|ALT_09252017_20** | ALT_09252017_20_scaffold_16_prodigal-single_261|ALT_PHAGE_39_18|ALT_09252017_20 | 100.000 | 148 | 0 | 0 | 1 | 148 | 1 | 148 | 4.59E-100 | 293 |
| **ALT_09252017_20_scaffold_16_259|ALT_09252017_20_Phage_39_18|ALT_09252017_20** | ALT_09252017_20_scaffold_16_259|ALT_09252017_20_Phage_39_18|ALT_09252017_20 | 100.000 | 148 | 0 | 0 | 1 | 148 | 1 | 148 | 4.59E-100 | 293 |
| **ALT_09252017_20_scaffold_16_259|ALT_09252017_20_Phage_39_18|ALT_09252017_20** | RHP_09252018_0_1um_scaffold_54_50|RHP_09252018_0_1um_UNK|RHP_09252018_0_1um | 94.595 | 148 | 8 | 0 | 1 | 148 | 1 | 148 | 7.52E-88 | 263 |
| **ALT_09252017_20_scaffold_16_259|ALT_09252017_20_Phage_39_18|ALT_09252017_20** | RHP_09252018_0_1um_scaffold_7414_3|RHP_09252018_0_1um_UNK|RHP_09252018_0_1um | 69.799 | 149 | 44 | 1 | 1 | 148 | 1 | 149 | 5.08E-57 | 185 |
| **ALT_09252017_20_scaffold_16_259|ALT_09252017_20_Phage_39_18|ALT_09252017_20** | ALT_03122018_0_1um_scaffold_6730_3|ALT_03122018_0_1um_UNK|ALT_03122018_0_1um | 79.333 | 150 | 29 | 1 | 1 | 148 | 1 | 150 | 3.7E-52 | 172 |
| **ALT_09252017_20_scaffold_16_259|ALT_09252017_20_Phage_39_18|ALT_09252017_20** | AB_072018_0_1um_scaffold_23907_1|AB_072018_0_1um_UNK|AB_072018_0_1um | 77.885 | 104 | 21 | 1 | 47 | 148 | 1 | 104 | 7.18E-37 | 132 |
| **ALT_09252017_20_scaffold_16_259|ALT_09252017_20_Phage_39_18|ALT_09252017_20** | ALT_072018_0_1um_scaffold_31513_1|ALT_072018_0_1um_UNK|ALT_072018_0_1um | 100.000 | 57 | 0 | 0 | 1 | 57 | 1 | 57 | 8.3E-31 | 115 |
| **ALT_09252017_20_scaffold_16_260|ALT_09252017_20_Phage_39_18|ALT_09252017_20** | ALT_04162018_0_2um_scaffold_5662_8|ALT_04162018_0_2um_UNK|ALT_04162018_0_2um | 100.000 | 125 | 0 | 0 | 1 | 125 | 1 | 125 | 1.06E-84 | 253 |
| **ALT_09252017_20_scaffold_16_260|ALT_09252017_20_Phage_39_18|ALT_09252017_20** | ALT_03122018_0_1um_scaffold_3_105|ALT_03122018_0_1um_partial_phage_39_31|ALT_03122018_0_1um | 100.000 | 125 | 0 | 0 | 1 | 125 | 1 | 125 | 1.06E-84 | 253 |
| **ALT_09252017_20_scaffold_16_260|ALT_09252017_20_Phage_39_18|ALT_09252017_20** | AB_072018_0_1um_scaffold_10951_2|AB_072018_0_1um_UNK|AB_072018_0_1um | 100.000 | 125 | 0 | 0 | 1 | 125 | 1 | 125 | 1.06E-84 | 253 |
| **ALT_09252017_20_scaffold_16_260|ALT_09252017_20_Phage_39_18|ALT_09252017_20** | ALT_082018_0_1um_scaffold_4834_8|ALT_082018_0_1um_UNK|ALT_082018_0_1um | 100.000 | 125 | 0 | 0 | 1 | 125 | 1 | 125 | 1.06E-84 | 253 |
| **ALT_09252017_20_scaffold_16_260|ALT_09252017_20_Phage_39_18|ALT_09252017_20** | ALT_09252017_20_scaffold_16_prodigal-single_262|ALT_PHAGE_39_18|ALT_09252017_20 | 100.000 | 125 | 0 | 0 | 1 | 125 | 1 | 125 | 1.06E-84 | 253 |
| **ALT_09252017_20_scaffold_16_260|ALT_09252017_20_Phage_39_18|ALT_09252017_20** | ALT_09252017_20_scaffold_16_260|ALT_09252017_20_Phage_39_18|ALT_09252017_20 | 100.000 | 125 | 0 | 0 | 1 | 125 | 1 | 125 | 1.06E-84 | 253 |
| **ALT_09252017_20_scaffold_16_260|ALT_09252017_20_Phage_39_18|ALT_09252017_20** | RHP_09252018_0_1um_scaffold_54_51|RHP_09252018_0_1um_UNK|RHP_09252018_0_1um | 86.179 | 123 | 17 | 0 | 1 | 123 | 1 | 123 | 1.09E-72 | 223 |
| **ALT_09252017_20_scaffold_16_260|ALT_09252017_20_Phage_39_18|ALT_09252017_20** | ALT_03122018_0_1um_scaffold_6730_4|ALT_03122018_0_1um_UNK|ALT_03122018_0_1um | 59.200 | 125 | 48 | 1 | 1 | 122 | 1 | 125 | 8.18E-47 | 157 |
| **ALT_09252017_20_scaffold_16_260|ALT_09252017_20_Phage_39_18|ALT_09252017_20** | AB_072018_0_1um_scaffold_23907_2|AB_072018_0_1um_UNK|AB_072018_0_1um | 59.200 | 125 | 48 | 1 | 1 | 122 | 1 | 125 | 8.18E-47 | 157 |
| **ALT_09252017_20_scaffold_16_260|ALT_09252017_20_Phage_39_18|ALT_09252017_20** | RHP_09252018_0_1um_scaffold_7414_2|RHP_09252018_0_1um_UNK|RHP_09252018_0_1um | 52.033 | 123 | 53 | 3 | 1 | 119 | 1 | 121 | 2.74E-34 | 125 |

| **BML_05172017_7_5m_scaffold_944_8|BML_05172017_7_5m_Fragmented_Jumbo_Phage_47_9|BML_05172017_7_5m** | BML_06132017_6_25m_scaffold_1104_9|BML_06132017_6_25m_UNK|BML_06132017_6_25m | 100.000 | 553 | 0 | 0 | 1 | 553 | 200 | 752 | 0.0 | 1140 |
| --- | --- | --- | --- | --- | --- | --- | --- | --- | --- | --- | --- |
| **BML_05172017_7_5m_scaffold_944_8|BML_05172017_7_5m_Fragmented_Jumbo_Phage_47_9|BML_05172017_7_5m** | BML_09012016_9m_scaffold_519_9|BML_09012016_9m_UNK|BML_09012016_9m | 100.000 | 553 | 0 | 0 | 1 | 553 | 200 | 752 | 0.0 | 1140 |
| **BML_05172017_7_5m_scaffold_944_8|BML_05172017_7_5m_Fragmented_Jumbo_Phage_47_9|BML_05172017_7_5m** | BML_09012016_5m_scaffold_1459_5|BML_09012016_5m_UNK|BML_09012016_5m | 100.000 | 553 | 0 | 0 | 1 | 553 | 200 | 752 | 0.0 | 1140 |
| **BML_05172017_7_5m_scaffold_944_8|BML_05172017_7_5m_Fragmented_Jumbo_Phage_47_9|BML_05172017_7_5m** | BML_05172017_8_0m_scaffold_189_76|BML_05172017_8_0m_Fragmented_Jumbo_Phage_47_10|BML_05172017_8_0m | 100.000 | 553 | 0 | 0 | 1 | 553 | 200 | 752 | 0.0 | 1140 |
| **BML_05172017_7_5m_scaffold_944_8|BML_05172017_7_5m_Fragmented_Jumbo_Phage_47_9|BML_05172017_7_5m** | BML_08042016_6_5m_scaffold_1_prodigal-single_330|BML_PHAGE_COMPLETE_47_13|BML_08042016_6_5m | 100.000 | 553 | 0 | 0 | 1 | 553 | 200 | 752 | 0.0 | 1140 |
| **BML_05172017_7_5m_scaffold_944_8|BML_05172017_7_5m_Fragmented_Jumbo_Phage_47_9|BML_05172017_7_5m** | BML_08042016_6_5m_scaffold_1_322|BML_08042016_6_5m_Potentially_Complete_Phage_47_13|BML_08042016_6_5m | 100.000 | 553 | 0 | 0 | 1 | 553 | 200 | 752 | 0.0 | 1140 |
| **BML_05172017_7_5m_scaffold_944_8|BML_05172017_7_5m_Fragmented_Jumbo_Phage_47_9|BML_05172017_7_5m** | BML_08042016_1_5m_scaffold_0_curated_closed_complete_prodigal-single_83|BML_PHAGE_COMPLETE_CU-CL_47_17|BML_08042016_1_5m | 100.000 | 553 | 0 | 0 | 1 | 553 | 200 | 752 | 0.0 | 1140 |
| **BML_05172017_7_5m_scaffold_944_8|BML_05172017_7_5m_Fragmented_Jumbo_Phage_47_9|BML_05172017_7_5m** | BML_08042016_1_5m_scaffold_0_75|BML_08042016_1_5m_Complete_Phage_47_17|BML_08042016_1_5m | 100.000 | 553 | 0 | 0 | 1 | 553 | 200 | 752 | 0.0 | 1140 |
| **BML_05172017_7_5m_scaffold_944_8|BML_05172017_7_5m_Fragmented_Jumbo_Phage_47_9|BML_05172017_7_5m** | BML_02172017_6_5m_scaffold_119_18|BML_02172017_6_5m_Fragmented_Jumbo_Phage_47_10|BML_02172017_6_5m | 100.000 | 553 | 0 | 0 | 1 | 553 | 200 | 752 | 0.0 | 1140 |
| **BML_05172017_7_5m_scaffold_944_8|BML_05172017_7_5m_Fragmented_Jumbo_Phage_47_9|BML_05172017_7_5m** | BML_02172017_0m_scaffold_162_72|BML_02172017_0m_Fragmented_Jumbo_Phage_47_9|BML_02172017_0m | 100.000 | 553 | 0 | 0 | 1 | 553 | 200 | 752 | 0.0 | 1140 |
| **BML_05172017_7_5m_scaffold_944_8|BML_05172017_7_5m_Fragmented_Jumbo_Phage_47_9|BML_05172017_7_5m** | BML_08182015_6_5m_scaffold_153_prodigal-single_10|BML_PHAGE_47_8|BML_08182015_6_5m | 100.000 | 553 | 0 | 0 | 1 | 553 | 200 | 752 | 0.0 | 1140 |
| **BML_05172017_7_5m_scaffold_944_8|BML_05172017_7_5m_Fragmented_Jumbo_Phage_47_9|BML_05172017_7_5m** | BML_08182015_6_5m_scaffold_153_9|BML_08182015_6_5m_Fragmented_Jumbo_Phage_47_8|BML_08182015_6_5m | 100.000 | 553 | 0 | 0 | 1 | 553 | 200 | 752 | 0.0 | 1140 |
| **BML_05172017_7_5m_scaffold_944_8|BML_05172017_7_5m_Fragmented_Jumbo_Phage_47_9|BML_05172017_7_5m** | BML_08182015_1_5m_scaffold_15_prodigal-single_110|BML_PHAGE_47_7|BML_08182015_1_5m | 100.000 | 553 | 0 | 0 | 1 | 553 | 200 | 752 | 0.0 | 1140 |
| **BML_05172017_7_5m_scaffold_944_8|BML_05172017_7_5m_Fragmented_Jumbo_Phage_47_9|BML_05172017_7_5m** | BML_08182015_1_5m_scaffold_15_97|BML_08182015_1_5m_Incomplete_Jumbo_Phage_47_7|BML_08182015_1_5m | 100.000 | 553 | 0 | 0 | 1 | 553 | 200 | 752 | 0.0 | 1140 |
| **BML_05172017_7_5m_scaffold_944_8|BML_05172017_7_5m_Fragmented_Jumbo_Phage_47_9|BML_05172017_7_5m** | BML_coassembly_scaffold_253_67|BML_coassembly_UNK|BML_coassembly | 100.000 | 553 | 0 | 0 | 1 | 553 | 200 | 752 | 0.0 | 1140 |
| **BML_05172017_7_5m_scaffold_944_8|BML_05172017_7_5m_Fragmented_Jumbo_Phage_47_9|BML_05172017_7_5m** | BML_05172017_7_5m_scaffold_944_8|BML_05172017_7_5m_Fragmented_Jumbo_Phage_47_9|BML_05172017_7_5m | 100.000 | 553 | 0 | 0 | 1 | 553 | 1 | 553 | 0.0 | 1137 |
| **BML_05172017_7_5m_scaffold_944_8|BML_05172017_7_5m_Fragmented_Jumbo_Phage_47_9|BML_05172017_7_5m** | BML_08042016_7_5m_scaffold_1523_1|BML_08042016_7_5m_UNK|BML_08042016_7_5m | 99.817 | 547 | 1 | 0 | 7 | 553 | 1 | 547 | 0.0 | 1120 |
| **BML_05172017_7_5m_scaffold_944_8|BML_05172017_7_5m_Fragmented_Jumbo_Phage_47_9|BML_05172017_7_5m** | BML_coassembly_scaffold_1632_67|BML_coassembly_UNK|BML_coassembly | 90.381 | 551 | 53 | 0 | 1 | 551 | 200 | 750 | 0.0 | 1050 |
| **BML_05172017_7_5m_scaffold_944_8|BML_05172017_7_5m_Fragmented_Jumbo_Phage_47_9|BML_05172017_7_5m** | BML_08042016_9_3m_scaffold_1472_1|BML_08042016_9_3m_Fragemented_Jumbo_Phage_47_6|BML_08042016_9_3m | 100.000 | 505 | 0 | 0 | 1 | 505 | 200 | 704 | 0.0 | 1041 |
| **BML_05172017_7_5m_scaffold_944_8|BML_05172017_7_5m_Fragmented_Jumbo_Phage_47_9|BML_05172017_7_5m** | BML_05172017_9_9m_scaffold_1617_1|BML_05172017_9_9m_UNK|BML_05172017_9_9m | 100.000 | 483 | 0 | 0 | 71 | 553 | 1 | 483 | 0.0 | 994 |
| **BML_05172017_7_5m_scaffold_944_8|BML_05172017_7_5m_Fragmented_Jumbo_Phage_47_9|BML_05172017_7_5m** | Salt_Pond_R2_restored_H2O_MG_scaffold_2210_7|JGI_Salt_Pond_R2_restored_H2O_MG_UNK|JGI_Salt_Pond_R2_restored_H2O_MG | 76.396 | 555 | 127 | 1 | 1 | 551 | 201 | 755 | 0.0 | 900 |
| **BML_05172017_7_5m_scaffold_944_8|BML_05172017_7_5m_Fragmented_Jumbo_Phage_47_9|BML_05172017_7_5m** | Salt_Pond_R2_restored_H2O_MG_scaffold_3519_7|JGI_Salt_Pond_R2_restored_H2O_MG_UNK|JGI_Salt_Pond_R2_restored_H2O_MG | 75.495 | 555 | 132 | 1 | 1 | 551 | 201 | 755 | 0.0 | 890 |
| **BML_05172017_7_5m_scaffold_944_8|BML_05172017_7_5m_Fragmented_Jumbo_Phage_47_9|BML_05172017_7_5m** | Salt_Pond_R2_restored_H2O_MG_scaffold_1583_5|JGI_Salt_Pond_R2_restored_H2O_MG_UNK|JGI_Salt_Pond_R2_restored_H2O_MG | 74.775 | 555 | 135 | 2 | 1 | 551 | 201 | 754 | 0.0 | 883 |
| **BML_05172017_7_5m_scaffold_944_8|BML_05172017_7_5m_Fragmented_Jumbo_Phage_47_9|BML_05172017_7_5m** | amazon_plume_scaffold_4132_9|amazon-plume_UNK|amazon-plume | 74.775 | 555 | 136 | 1 | 1 | 551 | 202 | 756 | 0.0 | 877 |
| **BML_05172017_7_5m_scaffold_944_8|BML_05172017_7_5m_Fragmented_Jumbo_Phage_47_9|BML_05172017_7_5m** | Salt_Pond_SF2_C_H2O_MG_scaffold_5447_4|JGI_Salt_Pond_SF2_C_H2O_MG_UNK|JGI_Salt_Pond_SF2_C_H2O_MG | 71.864 | 558 | 150 | 3 | 1 | 551 | 202 | 759 | 0.0 | 829 |
| **BML_05172017_7_5m_scaffold_944_8|BML_05172017_7_5m_Fragmented_Jumbo_Phage_47_9|BML_05172017_7_5m** | ALT_04162018_0_2um_scaffold_28758_1|ALT_04162018_0_2um_UNK|ALT_04162018_0_2um | 70.109 | 552 | 164 | 1 | 1 | 551 | 171 | 722 | 0.0 | 826 |
| **BML_05172017_7_5m_scaffold_944_8|BML_05172017_7_5m_Fragmented_Jumbo_Phage_47_9|BML_05172017_7_5m** | ALT_03122018_0_1um_scaffold_13766_1|ALT_03122018_0_1um_UNK|ALT_03122018_0_1um | 70.109 | 552 | 164 | 1 | 1 | 551 | 171 | 722 | 0.0 | 826 |
| **BML_05172017_7_5m_scaffold_944_8|BML_05172017_7_5m_Fragmented_Jumbo_Phage_47_9|BML_05172017_7_5m** | ALT_09252017_20_scaffold_24488_1|ALT_09252017_20_UNK|ALT_09252017_20 | 70.109 | 552 | 164 | 1 | 1 | 551 | 171 | 722 | 0.0 | 826 |
| **BML_05172017_7_5m_scaffold_944_8|BML_05172017_7_5m_Fragmented_Jumbo_Phage_47_9|BML_05172017_7_5m** | ALT_03122018_0_2um_scaffold_21282_1|ALT_03122018_0_2um_UNK|ALT_03122018_0_2um | 70.109 | 552 | 164 | 1 | 1 | 551 | 113 | 664 | 0.0 | 825 |
| **BML_05172017_7_5m_scaffold_944_8|BML_05172017_7_5m_Fragmented_Jumbo_Phage_47_9|BML_05172017_7_5m** | BML_08182015_6_5m_scaffold_392_30|BML_08182015_6_5m_UNK|BML_08182015_6_5m | 69.565 | 552 | 167 | 1 | 1 | 551 | 200 | 751 | 0.0 | 825 |
| **BML_05172017_7_5m_scaffold_944_8|BML_05172017_7_5m_Fragmented_Jumbo_Phage_47_9|BML_05172017_7_5m** | BML_08182015_1_5m_scaffold_19_prodigal-single_69|BML_PHAGE_COMPLETE_42_5|BML_08182015_1_5m | 69.565 | 552 | 167 | 1 | 1 | 551 | 200 | 751 | 0.0 | 825 |
| **BML_05172017_7_5m_scaffold_944_8|BML_05172017_7_5m_Fragmented_Jumbo_Phage_47_9|BML_05172017_7_5m** | BML_08182015_1_5m_scaffold_19_68|BML_08182015_1_5m_Complete_Jumbo_Phage_42_5|BML_08182015_1_5m | 69.565 | 552 | 167 | 1 | 1 | 551 | 200 | 751 | 0.0 | 825 |
| **BML_05172017_7_5m_scaffold_944_8|BML_05172017_7_5m_Fragmented_Jumbo_Phage_47_9|BML_05172017_7_5m** | LacPavin_0419_WC70S_scaffold_1363466_24|LacPavin_0419_WC70S_UNK|LacPavin_0419_WC70S | 72.285 | 534 | 148 | 0 | 1 | 534 | 209 | 742 | 0.0 | 825 |
| **BML_05172017_7_5m_scaffold_944_8|BML_05172017_7_5m_Fragmented_Jumbo_Phage_47_9|BML_05172017_7_5m** | LacPavin_0419_WC53_scaffold_75329_267|LacPavin_0419_WC53_Potentially_Complete_Phage_42_65|LacPavin_0419_WC53 | 72.285 | 534 | 148 | 0 | 1 | 534 | 209 | 742 | 0.0 | 825 |
| **BML_05172017_7_5m_scaffold_944_8|BML_05172017_7_5m_Fragmented_Jumbo_Phage_47_9|BML_05172017_7_5m** | BML_06132017_6_25m_scaffold_2644_5|BML_06132017_6_25m_UNK|BML_06132017_6_25m | 70.072 | 558 | 162 | 1 | 1 | 553 | 201 | 758 | 0.0 | 825 |
| **BML_05172017_7_5m_scaffold_944_8|BML_05172017_7_5m_Fragmented_Jumbo_Phage_47_9|BML_05172017_7_5m** | BML_09012016_9m_scaffold_954_8|BML_09012016_9m_UNK|BML_09012016_9m | 70.072 | 558 | 162 | 1 | 1 | 553 | 201 | 758 | 0.0 | 825 |
| **BML_05172017_7_5m_scaffold_944_8|BML_05172017_7_5m_Fragmented_Jumbo_Phage_47_9|BML_05172017_7_5m** | BML_08042016_6_5m_scaffold_954_8|BML_08042016_6_5m_UNK|BML_08042016_6_5m | 70.072 | 558 | 162 | 1 | 1 | 553 | 201 | 758 | 0.0 | 825 |
| **BML_05172017_7_5m_scaffold_944_8|BML_05172017_7_5m_Fragmented_Jumbo_Phage_47_9|BML_05172017_7_5m** | BML_08042016_1_5m_scaffold_666_20|BML_08042016_1_5m_UNK|BML_08042016_1_5m | 70.072 | 558 | 162 | 1 | 1 | 553 | 201 | 758 | 0.0 | 825 |
| **BML_05172017_7_5m_scaffold_944_8|BML_05172017_7_5m_Fragmented_Jumbo_Phage_47_9|BML_05172017_7_5m** | BML_02172017_0m_scaffold_1637_3|BML_02172017_0m_UNK|BML_02172017_0m | 70.072 | 558 | 162 | 1 | 1 | 553 | 201 | 758 | 0.0 | 825 |
| **BML_05172017_7_5m_scaffold_944_8|BML_05172017_7_5m_Fragmented_Jumbo_Phage_47_9|BML_05172017_7_5m** | BML_coassembly_scaffold_181_63|BML_coassembly_UNK|BML_coassembly | 70.072 | 558 | 162 | 1 | 1 | 553 | 201 | 758 | 0.0 | 825 |
| **BML_05172017_7_5m_scaffold_944_8|BML_05172017_7_5m_Fragmented_Jumbo_Phage_47_9|BML_05172017_7_5m** | ALT_04162018_0_2um_scaffold_28756_1|ALT_04162018_0_2um_Alphaproteobacteria_45_26|ALT_04162018_0_2um | 69.928 | 552 | 165 | 1 | 1 | 551 | 171 | 722 | 0.0 | 824 |
| **BML_05172017_7_5m_scaffold_944_8|BML_05172017_7_5m_Fragmented_Jumbo_Phage_47_9|BML_05172017_7_5m** | ALT_03122018_0_2um_scaffold_21294_1|ALT_03122018_0_2um_UNK|ALT_03122018_0_2um | 69.928 | 552 | 165 | 1 | 1 | 551 | 113 | 664 | 0.0 | 824 |
| **BML_05172017_7_5m_scaffold_944_8|BML_05172017_7_5m_Fragmented_Jumbo_Phage_47_9|BML_05172017_7_5m** | ALT_09252017_20_scaffold_24482_1|ALT_09252017_20_UNK|ALT_09252017_20 | 69.928 | 552 | 165 | 1 | 1 | 551 | 171 | 722 | 0.0 | 824 |
| **BML_05172017_7_5m_scaffold_944_8|BML_05172017_7_5m_Fragmented_Jumbo_Phage_47_9|BML_05172017_7_5m** | RHP_09252018_0_1um_scaffold_7449_9|RHP_09252018_0_1um_UNK|RHP_09252018_0_1um | 71.403 | 549 | 156 | 1 | 1 | 549 | 203 | 750 | 0.0 | 824 |
| **BML_05172017_7_5m_scaffold_944_8|BML_05172017_7_5m_Fragmented_Jumbo_Phage_47_9|BML_05172017_7_5m** | AB_092018_0_1um_scaffold_25652_2|AB_092018_0_1um_UNK|AB_092018_0_1um | 72.575 | 536 | 147 | 0 | 1 | 536 | 212 | 747 | 0.0 | 823 |
| **BML_05172017_7_5m_scaffold_944_8|BML_05172017_7_5m_Fragmented_Jumbo_Phage_47_9|BML_05172017_7_5m** | BML_05172017_7_5m_scaffold_11879_1|BML_05172017_7_5m_Alphaproteobacteria_39_6|BML_05172017_7_5m | 71.482 | 533 | 152 | 0 | 1 | 533 | 11 | 543 | 0.0 | 821 |
| **BML_05172017_7_5m_scaffold_944_8|BML_05172017_7_5m_Fragmented_Jumbo_Phage_47_9|BML_05172017_7_5m** | BML_coassembly_scaffold_214_prodigal-single_9|BML_PHAGE_48_21|BML_coassembly | 70.221 | 544 | 162 | 0 | 1 | 544 | 201 | 744 | 0.0 | 820 |
| **BML_05172017_7_5m_scaffold_944_8|BML_05172017_7_5m_Fragmented_Jumbo_Phage_47_9|BML_05172017_7_5m** | BML_coassembly_scaffold_214_9|BML_coassembly_Incompleted_Jumbo_Phage_48_21|BML_coassembly | 70.221 | 544 | 162 | 0 | 1 | 544 | 201 | 744 | 0.0 | 820 |
| **BML_05172017_7_5m_scaffold_944_8|BML_05172017_7_5m_Fragmented_Jumbo_Phage_47_9|BML_05172017_7_5m** | BC_09192017_0_5m_scaffold_15799_2|BC_09192017_0_5m_UNK|BC_09192017_0_5m | 69.565 | 552 | 167 | 1 | 1 | 551 | 200 | 751 | 0.0 | 820 |
| **BML_05172017_7_5m_scaffold_944_8|BML_05172017_7_5m_Fragmented_Jumbo_Phage_47_9|BML_05172017_7_5m** | ALT_03122018_0_1um_scaffold_4809_1|ALT_03122018_0_1um_UNK|ALT_03122018_0_1um | 69.203 | 552 | 169 | 1 | 1 | 551 | 200 | 751 | 0.0 | 819 |
| **BML_05172017_7_5m_scaffold_944_8|BML_05172017_7_5m_Fragmented_Jumbo_Phage_47_9|BML_05172017_7_5m** | BML_02172017_6_5m_scaffold_879_10|BML_02172017_6_5m_UNK|BML_02172017_6_5m | 70.902 | 543 | 158 | 0 | 1 | 543 | 201 | 743 | 0.0 | 819 |
| **BML_05172017_7_5m_scaffold_944_8|BML_05172017_7_5m_Fragmented_Jumbo_Phage_47_9|BML_05172017_7_5m** | ALT_03122018_0_1um_scaffold_1120_16|ALT_03122018_0_1um_UNK|ALT_03122018_0_1um | 69.384 | 552 | 168 | 1 | 1 | 551 | 200 | 751 | 0.0 | 819 |
| **BML_05172017_7_5m_scaffold_944_8|BML_05172017_7_5m_Fragmented_Jumbo_Phage_47_9|BML_05172017_7_5m** | ALT_09252017_20_scaffold_10174_7|ALT_09252017_20_UNK|ALT_09252017_20 | 69.384 | 552 | 168 | 1 | 1 | 551 | 200 | 751 | 0.0 | 819 |
| **BML_05172017_7_5m_scaffold_944_8|BML_05172017_7_5m_Fragmented_Jumbo_Phage_47_9|BML_05172017_7_5m** | FFC_04162018_0_1um_scaffold_1287_6|FFC_04162018_0_1um_UNK|FFC_04162018_0_1um | 69.636 | 550 | 166 | 1 | 1 | 550 | 211 | 759 | 0.0 | 818 |
| **BML_05172017_7_5m_scaffold_944_8|BML_05172017_7_5m_Fragmented_Jumbo_Phage_47_9|BML_05172017_7_5m** | ALT_082018_0_1um_scaffold_43660_1|ALT_082018_0_1um_UNK|ALT_082018_0_1um | 71.482 | 533 | 152 | 0 | 1 | 533 | 200 | 732 | 0.0 | 818 |
| **BML_05172017_7_5m_scaffold_944_8|BML_05172017_7_5m_Fragmented_Jumbo_Phage_47_9|BML_05172017_7_5m** | LacPavin_0818_WC50_scaffold_610821_4|LacPavin_0818_WC50_UNK|LacPavin_0818_WC50 | 69.147 | 551 | 169 | 1 | 1 | 551 | 180 | 729 | 0.0 | 818 |
| **BML_05172017_7_5m_scaffold_944_8|BML_05172017_7_5m_Fragmented_Jumbo_Phage_47_9|BML_05172017_7_5m** | ALT_07252016_14_scaffold_2744_2|ALT_07252016_14_UNK|ALT_07252016_14 | 69.203 | 552 | 169 | 1 | 1 | 551 | 200 | 751 | 0.0 | 817 |
| **BML_05172017_7_5m_scaffold_944_8|BML_05172017_7_5m_Fragmented_Jumbo_Phage_47_9|BML_05172017_7_5m** | ALT_04162018_0_2um_scaffold_312_55|ALT_04162018_0_2um_UNK|ALT_04162018_0_2um | 69.203 | 552 | 169 | 1 | 1 | 551 | 200 | 751 | 0.0 | 817 |
| **BML_05172017_7_5m_scaffold_944_8|BML_05172017_7_5m_Fragmented_Jumbo_Phage_47_9|BML_05172017_7_5m** | ALT_03122018_0_1um_scaffold_415_40|ALT_03122018_0_1um_UNK|ALT_03122018_0_1um | 70.364 | 550 | 161 | 2 | 1 | 549 | 203 | 751 | 0.0 | 817 |
| **BML_05172017_7_5m_scaffold_944_8|BML_05172017_7_5m_Fragmented_Jumbo_Phage_47_9|BML_05172017_7_5m** | ALT_03122018_0_1um_scaffold_220_31|ALT_03122018_0_1um_UNK|ALT_03122018_0_1um | 69.203 | 552 | 169 | 1 | 1 | 551 | 200 | 751 | 0.0 | 817 |
| **BML_05172017_7_5m_scaffold_944_8|BML_05172017_7_5m_Fragmented_Jumbo_Phage_47_9|BML_05172017_7_5m** | ALT_09252017_20_scaffold_362_54|ALT_09252017_20_UNK|ALT_09252017_20 | 69.203 | 552 | 169 | 1 | 1 | 551 | 200 | 751 | 0.0 | 817 |
| **BML_05172017_7_5m_scaffold_944_8|BML_05172017_7_5m_Fragmented_Jumbo_Phage_47_9|BML_05172017_7_5m** | FFC_04162018_0_1um_scaffold_645_8|FFC_04162018_0_1um_UNK|FFC_04162018_0_1um | 69.982 | 553 | 163 | 1 | 1 | 550 | 201 | 753 | 0.0 | 817 |
| **BML_05172017_7_5m_scaffold_944_8|BML_05172017_7_5m_Fragmented_Jumbo_Phage_47_9|BML_05172017_7_5m** | ALT_072018_0_1um_scaffold_493_48|ALT_072018_0_1um_UNK|ALT_072018_0_1um | 69.022 | 552 | 170 | 1 | 1 | 551 | 200 | 751 | 0.0 | 817 |
| **BML_05172017_7_5m_scaffold_944_8|BML_05172017_7_5m_Fragmented_Jumbo_Phage_47_9|BML_05172017_7_5m** | AB_072018_0_1um_scaffold_354_23|AB_072018_0_1um_UNK|AB_072018_0_1um | 70.599 | 551 | 160 | 2 | 1 | 551 | 203 | 751 | 0.0 | 817 |
[truncated: 536,264 more chars]
